# Unveiling the Peptidase Network Orchestrating Hemoglobin Catabolism in *Rhodnius prolixus*

**DOI:** 10.1016/j.mcpro.2024.100775

**Published:** 2024-04-23

**Authors:** Radouane Ouali, Sabrina Bousbata

**Affiliations:** Laboratory of Vector-Pathogen Biology, Proteomic Platform, Department of Molecular Biology, Université Libre de Bruxelles, Gosselies, Belgium

**Keywords:** protein digestion, disease vectors, hematophagy, ectoparasites, *Trypanosoma cruzi*

## Abstract

Chagas disease is transmitted to humans by obligatory hematophagous insects of Triatominae subfamily, which feeds on various hosts to acquire their nutritional sustenance derived from blood proteins. Hemoglobin (Hb) digestion is a pivotal metabolic feature of triatomines, representing a key juncture in their competence toward *Trypanosoma cruzi*; however, it remains poorly understood. To explore the Hb digestion pathway in *Rhodnius prolixus*, a major Chagas disease vector, we employed an array of approaches for activity profiling of various midgut-associated peptidases using specific substrates and inhibitors. Dissecting the individual contribution of each peptidase family in Hb digestion has unveiled a predominant role played by aspartic proteases and cathepsin B-like peptidases. Determination of peptidase-specific cleavage sites of these key hemoglobinases, in conjunction with mass spectrometry–based identification of *in vivo* Hb-derived fragments, has revealed the intricate network of peptidases involved in the Hb digestion pathway. This network is initiated by aspartic proteases and subsequently sustained by cysteine proteases belonging to the C1 family. The process is continued simultaneously by amino and carboxypeptidases. The comprehensive profiling of midgut-associated aspartic proteases by quantitative proteomics has enabled the accurate revision of gene annotations within the A1 family of the *R. prolixus* genome. Significantly, this study also serves to illuminate a potentially important role of the anterior midgut in blood digestion. The expanded repertoire of midgut-associated proteases presented in this study holds promise for the identification of novel targets aimed at controlling the transmission of Chagas disease.

## Highlights

• Enzymatic activity profiling of *Rhodnius prolixus* midgut-associated peptidases.
• Characterization of the intricate network of peptidases involved in hemoglobinolysis.
• Accurate revision of gene annotations within the A1 family of the *R. prolixus* genome.
• Illumination of the obscured role of the anterior midgut in blood digestion.

## Graphical abstract

**Figure:**
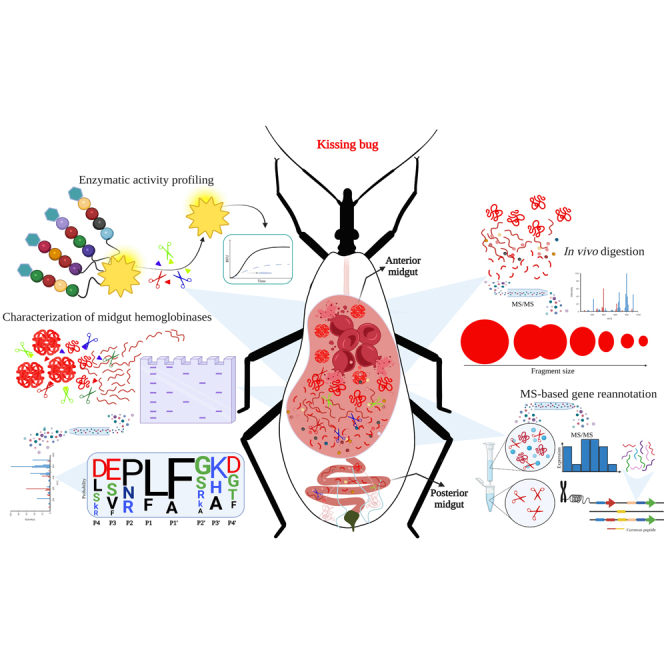

## In Brief

Through this study, we meticulously uncovered the complex network of peptidases orchestrating hemoglobin digestion in *Rhodnius prolixus*, a major Chagas disease vector. Our thorough dissection pinpointed the substantial contributions of aspartic proteases and cathepsin B-like peptidases in this intricate metabolic cascade. Furthermore, we illuminated the anterior midgut's pivotal role in blood digestion. This discovery offers promising opportunities to identify new targets for controlling Chagas disease transmission.

Triatomines or kissing bugs are hematophagous hemipterans of the Reduviidae family. This subfamily includes 157 species distributed over 18 genera (1). Triatomines are temporary ectoparasites, which feed across unlimited number of warm vertebrate hosts. They are notable for their remarkable ability to ingest an astonishing amount of blood in a single meal, often exceeding twelve times their own body weight (2). All triatomine species are proven or suspected vectors of *Trypanosoma cruzi*, the causative agent of Chagas disease; one of the most important neglected tropical disease worldwide (https://www.who.int/news-room/fact-sheets/detail/chagas-disease-(american-trypanosomiasis)). In fact, it affects about 7 million people, causing more than 14,000 deaths annually. Triatomines midgut represents an important interface for triatomine–trypanosome interactions. Indeed, vectorial competence toward *T. cruzi* is closely linked to the blood feeding and digestion; thus, an understanding of their physiology is essential for the development of new strategies to control Chagas disease transmission. The blood meal that consists mostly of hemoglobin (Hb), provides the essential nutritional components for the insect to fulfill various metabolic processes (3). Hb is released by erythrocytes lysis (4) in the anterior midgut (AM). This segment has long been regarded as a storage site devoid of digestive involvement. This perception was rooted in investigations of tissue ultrastructure (5, 6, 7), morphometric analyses of organelles (8), and isolated localization of peptidases (9, 10). However, the application of cutting-edge technologies has cast doubt upon these assumptions (11, 12, 13). Indeed, several digestive enzymes have been validated for their expression and activity within this tissue (11, 14, 15, 16). The subsequent segment of the digestive tract is the posterior midgut (PM) considered as digestive and absorptive organ of the midgut (5, 17, 18). Finally, the rectum that ensures excretion processes, constitutes also the place where parasite metacyclogenesis occurs (19, 20). The midgut is lined by a lipoprotein structure, known as the perimicrovillar membranes, which is produced in response to blood ingestion (21, 22). These membranes serve to shield the digestive epithelium from various aggressions, facilitates detoxification of heme by promoting hemozoin formation (23), aids in the digestive process by compartmentalizing different classes of digestive enzymes, and enhances nutrient absorption (24, 25). Our understanding of blood proteins digestion in triatomines is fragmented and limited to the study of individual proteases of different species, lacking a comprehensive overview of this process (26, 27, 28, 29, 30, 31, 32). From previous investigations, we discern that digestion is facilitated by lysosomal cathepsin-like enzymes (33). A pronounced dearth of molecular insights into the digestive physiology of triatomines is evident. Thus, the present study endeavors to elucidate the intricate process of Hb digestion, within *R. prolixus*, which despite its status as a model organism for insect physiology, enigmatic facets of its digestion persist. Employing an array of approaches encompassing chemical biology tools, proteomics, and enzymology will allow us to unravel the intricate peptidase network governing hemoglobinolysis. This multifaceted approach aims to delineate the distinctive contribution of each protease family to the digestive process. Additionally, this endeavor aims to illuminate the heretofore obscure role of the AM in blood digestion, potentially heralding novel strategies for triatomine control.

## Experimental procedures

### Chemicals

The following reagents, substrates, and protease inhibitors were utilized in this research: Bz-Arg-Gly-Phe-Phe-Pro-4M2NA, Leu-AMC, pepstatin A, bestatin, RKLLW-NH2, human Hb, and fluorescamine from Sigma-Aldrich. Z-Phe-Arg-AMC and Z-Phe-Leu-OH from Bachem, Z-Ala-Ala-Asn-AMC, CA-074, and legumain inhibitor1 from MedChemExpress. E-64 and EDTA from Merck. PMSF and EDTA-free protease inhibitor cocktail from Roche (Merck). The stock solutions of these products were prepared, following the instructions provided by the respective providers.

### Insects and Midgut Tissues Preparation

*R. prolixus* bugs were reared at a stable temperature of 28 °C and 60 to 80% humidity. The rearing conditions included a photoperiod of 12 h of light, followed by 12 h of darkness. The insects were maintained and fed on rabbit blood (12). Midguts were dissected (Supplemental Fig. S1) from both unfed and fully engorged virgin females at various time points: 6 h, 1, 2, 7, and 14 days postfeeding. The AM and PM were separated and then carefully incised to recover their contents separately into Eppendorf tubes. The samples were then centrifuged at 5000*g* for 5 min, and the supernatants were filtered using 0.2 μm filters (Merck) and stored at −80 °C until further analysis. Simultaneously, the tissues were rigorously washed with cold PBS and stored at −80 °C until use. Each biological replicate in this study represents five pooled midgut tissues or contents. In order to obtain the midgut tissue extract, the tissues underwent a process of freeze-thaw at −80 °C, followed by three cycles of sonication. Subsequently, the samples were centrifuged at 13,000*g* for 15 min at 4 °C. The supernatant was collected, and the extracted proteins were quantified using Pierce 660 nm Protein Assay (Thermo Fisher Scientific Inc, Rockford). The protein extracts were then subjected to analysis of their patterns by SDS-PAGE electrophoresis on a Tris-Tricine 12% gel.

### Experimental Design and Statistical Rationale

For the mass spectrometry (MS)-based determination of hemoglobinase cleavage preferences (detailed below), four conditions were analyzed as follows: A: undigested Hb, B: Hb digested with midgut content treated with an inhibitor cocktail, C: Hb digested with midgut content treated with an inhibitor cocktail excluding pepstatin A, D: Hb digested with midgut content treated with an inhibitor cocktail excluding E-64, CA-074, and RKLLW-NH2. A and B serve as the control conditions, while C and D represent the experimental conditions. Each reaction was performed in triplicate for the experimental conditions, using three samples of midgut content, each corresponding to a pool of five insects. For the control conditions, the reactions were carried out in duplicate. The peptides identified in samples (C and D) and present in at least one control were considered to result from nonspecific digestion and were therefore excluded. Unique peptides specific to conditions C and D, respectively, and identified in at least two replicates, were used to determine the preferential cleavage sites of the aspartic and cysteine proteases, respectively.

For mapping the *in vivo* proteolytic cleavage of Hb by tandem mass spectrometry (MS/MS) (detailed below), the analysis was conducted across three biological replicates, each corresponding to the digestive content of an independent insect. Peptides identified in all three biological replicates and identified at least five times per replicate were considered for establishing the cleavage map of Hb.

To monitor the temporal dynamics of aspartic protease expression through proteomics, the purification of expressed aspartic proteases at various postfeeding times was carried out using 20 pooled AM from independent insects for each time point. The relative abundance of each identified protein corresponds to the intensity ratio of that protein compared to the total intensity of all identified proteins in the same condition.

### Enzymatic Activity Profiling of *R. prolixus* Midgut Peptidases

In pursuit of assessment of the activity exhibited by diverse families of *R. prolixus* digestive peptidases and monitoring their activity profile at different postprandial times, bespoke substrates and inhibitors were employed. Briefly, 40 μM Bz-Arg-Gly-Phe-Phe-Pro-4M2NA were used to assess the activity of aspartic peptidases, 25 μM Z-Phe-Arg-AMC for C1 cysteine proteases, 30 μM Leu-AMC for aminopeptidases, 1 mM Z-Phe-Leu-OH for carboxypeptidases, and 30 μM Z-Ala-Ala-Asn-AMC for asparagine endopeptidases. Enzymatic activities were measured in midgut tissue extracts and luminal contents at 30 °C in 0.1 M citrate-phosphate buffer in a range of pH 2 to 8. For cysteine peptidases, 2.5 mM of DTT were added to the reaction buffer. The assessment of enzymatic activity in the presence of the inhibitor was executed by preincubating the sample with the inhibitor for 15 min at 30 °C before adding the substrate. The inhibitors were employed at the following concentrations: 10 μM pepstatin A, E−64, and RKLLW-NH2; 10 nM CA-074 and legumain inhibitor 1; and 1 mM PMSF, 0.1 μM bestatin, and 10 mM EDTA.

Proteolytic activity was continuously assessed for 90 min at 30 °C by measuring fluorescence every 5 min, using a microplate fluorescence reader (SpectraMax i3, Molecular Devices) at 340 nm excitation and 425 nm emission wavelengths for aspartic peptidases substrate and at 360 nm excitation and 465 nm emission wavelengths for the other AMC-containing substrates. Carboxypeptidases activity was monitored after derivatization of liberated product with 0.1% fluorescamine at 390 nm excitation and 465 nm emission wavelengths. All measurements were performed in triplicate. Assays of asparagine endopeptidases and cathepsins L-like cysteine proteases were measured in the presence of 10 nM of CA-074 (34).

### *In Vitro* Digestion of Hb Assay

Human Hb digestion was quantified in the presence of fluorescamine, allowing to quantify neo-N-termini extremities formed following proteolysis. One microgram tissue extracts or 5 μl intestinal content extracts were added to the reaction mixture containing 20 μg Hb, 0.1% fluorescamine in 0.1 M citrate-phosphate buffer in a pH range from 2 to 8, supplemented with 2 mM DTT. The evaluation of Hb digestion rate in the presence of an inhibitor was executed at the determined optimum pH by preincubating the sample with the inhibitor for 15 min at the concentration mentioned above. Hemoglobinolytic activity was assessed for 90 min at 30 °C by measuring fluorescence every 5 min at 390 nm excitation and 465 nm emission wavelengths. All measurements were performed in triplicate. Concurrently, Hb digestion by *R. prolixus* samples was performed under different pH conditions as well as at its optimal pH, in the presence of an inhibitor, all within a total volume of 50 μl. Subsequently, the samples were incubated for 16 h at 30 °C. The enzymatic reaction was stopped by adding 4X Laemmli buffer (0.125 M Tris–HCl, 4% SDS, 20% glycerol, 10% 2-mercaptoethanol, 0.004% bromophenol blue), followed by denaturation at 99 °C for 5 min. The samples were then subjected to analysis by SDS-PAGE on Tris-Tricine 4 to 20% gradient gel under reducing conditions. Gel was stained with Coomassie Blue G-250 (Thermo Fisher Scientific Inc).

### MSBased Determination of *R. prolixus* Hemoglobinases Cleavage Preferences

Twenty micrograms human Hb were digested with 5 μl of the luminal content in the presence and absence of a cocktail of protease inhibitors. Control experiments included undigested Hb or Hb digested with the insect sample previously exposed for 15 min to a cocktail of inhibitors containing E-64, pepstatin A, RKLLW-NH2, CA-074, legumain inhibitor 1, PMSF, bestatin, and EDTA at the concentration indicated above. For determining the cleavage sites of aspartic proteases, pepstatin A was excluded, while for identifying C1 cysteine proteases cleavage sites, E-64, CA-074, and RKLLW-NH2 were omitted. The digestion was conducted at 30 °C for 16 h in citrate-phosphate buffer pH 5 supplemented with 2.5 mM DTT in a total volume of 100 μl. The reactions were performed in triplicate. Subsequently, the samples were ultrafiltrated using 10 kDa molecular weight cut-off (MWCO) column (Merck). The filtrate, containing peptides, resulting from Hb digestion was analyzed using nano-liquid chromatography (LC)-ESI-MS/MS timsTOF Pro (Bruker) coupled with a nanoElute ultra-high performance liquid chromatography. Peptides were separated by nanoElute on a 75 μm ID, 25 cm C18 column with integrated CaptiveSpray insert (Aurora, IonOpticks) at a flow rate of 250 nl/min, at 50 °C. Liquid chromatography mobile phases were A (water with 0.1% formic acid (v/v)) and B (acetonitrile with 0.1% formic acid (v/v)). Samples were loaded directly onto the analytical column at a constant pressure of 800 bar. The peptides were injected and the organic content of the mobile phase was increased linearly from 2% B to 15% in 45 min, from 15% B to 22% in 25 min, from 22% B to 35% in 15 min, and from 35% to 85% in 8 min. Data acquisition on the timsTOF Pro was performed using Compass Hystar version 5.1 (Bruker) (https://www.bruker.com/en/products-and-solutions/mass-spectrometry/lc-ms/compass-hystar.html) and timsControl version 3.0 (Bruker) (https://www.bruker.com/en/products-and-solutions/mass-spectrometry/timstof/timstof-scp.html). Data were acquired using 100 ms trapped ion mobility spectrometry accumulation time and mobility (1/K0) range from 0.6 to 1.6 Vs/cm^2^. Mass spectrometric analyses were carried out using the parallel accumulation serial fragmentation (PASEF) acquisition method (35). One MS spectrum followed by ten PASEF MS/MS spectra per total cycle of 1.1 s. The capillary voltage was set to 1400 V, and both MS and MS/MS spectra were acquired in the range of 100 to 1700 *m/z*. Collision energy was linearly ramped as a function of mobility, starting at 59 eV at 1/K0 = 1.6 Vs/cm^2^ and gradually decreasing to 20 eV at 1/K0 = 0.6 Vs/cm^2^. The target value was set to 20,000 arbitrary units. Tandem mass spectra were extracted, charge state deconvoluted, and deisotoped by Data analysis version 5.3 (Bruker). All MS/MS samples were analyzed using Mascot version 2.8.1 (Matrix Science). Mascot was set up to search the Human Proteome_220705 database (79,684 entries) and *R. Prolixus_*230123 database (18,471 entries) with nonspecific enzyme digestion parameter. Mascot was searched with a fragment ion mass tolerance of 0.050 Da and a parent ion tolerance of 15 ppm. Oxidation of methionine was specified in Mascot as a variable modification. Scaffold version 5.2.2 (Proteome Software Inc) (https://www.proteomesoftware.com/products/scaffold-5) was used to validate MS/MS-based peptide and protein identifications. Peptide identifications were accepted with >91% probability to achieve an false discovery rate <1% by the percolator posterior error probability calculation (Matrix Science). Protein identifications were accepted if they could be established at >34% probability to achieve an false discovery rate <1% and contained at least three identified peptides. Protein probabilities were assigned by the Prophet algorithm (36).

### Mapping the *In Vivo* Proteolytic Cleavage of Hb Using MS

The AM and PM contents from unfed, 6 h, and 1, 2, 7, and 14 days postfed insects, were treated with EDTA-free protease inhibitor cocktail. Subsequently, the samples were denatured by adding Laemmli 4X buffer and heated at 99 °C for 5 min in order to be analyzed by SDS-PAGE electrophoresis on a 10 to 20% Tris-Tricine gradient gel under reducing conditions. Gel was stained with Coomassie Blue G-250 (Thermo Fisher Scientific Inc), or silver (Serva). Concurrently, the digestive content of AM, specifically at 14 days postfeeding, underwent filtration using a 10 kDa MWCO ultrafiltration column. The run-through, containing small molecules, was then subjected to MS/MS analysis as described previously (Mascot was set up to search the *R*. *prolixus*_230123 (18,471 entries) and *Oryctolagus cuniculus*_230418 (59,928 entries) databases, with nonspecific enzyme digestion parameter). The identified peptides will allow to reconstitute the *in vivo* cleavage map of Hb by *R. prolixus* peptidases.

### Aspartic Proteases Purification by Pepstatin A Affinity Chromatography

Aspartic proteases were purified as described previously by Balczun *et al.* (28) using immobilized pepstatin A with some modifications. 2 ml pepstatin A resin (G-Biosciences) were loaded onto empty gravity column (Bio-Rad) and subsequently equilibrated with 5 ml of binding buffer (0.1 M citrate, 0.5 M sodium chloride, pH 5). The resin was then incubated with 200 μg AM tissue extract (from unfed, 6 h, 1, 2, 7, and 14 days postfed insects) for 2 h on a rotary wheel at 300 rpm at 4 °C. The mixture was reloaded onto the column and allowed to flow through under gravity. The resin was washed four times with four resin bed volumes of binding buffer, followed by an additional four washes with four resin bed volumes of wash buffer (0.5 M sodium chloride). The bound proteins were eluted with six washes using four resin bed volumes of elution buffer (0.1 M sodium bicarbonate, 0.5 M sodium chloride, pH 8.7), and 4 ml size fractions were collected. The different recovered fractions were concentrated using 3 kDa MWCO columns (Merck) and analyzed by SDS-PAGE electrophoresis on 12% Tris-Tricine gel under reducing conditions. Gel was stained with silver staining kit (Serva).

### Monitoring the Temporal Dynamics of Aspartic Proteases Expression by Proteomics

The eluted fractions obtained from pepstatin A affinity chromatography purification were pooled and concentrated using 3 kDa MWCO columns. The concentrated samples were denatured by adding 24 mg of urea, and the volume was completed to 50 μl by adding Hepes 20 mM pH 8. After homogenization, 15 mM DTT were added and the mixture was incubated for 30 min at 55 °C. Subsequently, 30 mM iodoacetamide was added, and the sample was incubated in darkness for 15 min. One hundred fifty microliters 20 mM Hepes pH 8 was added to the sample, followed by the addition of 1 μg trypsin (Promega) and incubated overnight at 37 °C, with continuous shaking at 750 rpm. The digestion process was stopped by adding 2 μl 100% TFA. The tryptic peptides were purified using C18 zip-tips (Proxeon) and subsequently eluted with 60% acetonitrile. The buffer was evaporated using a speed-vac system, and the peptides were reconstituted in 10 μl 0.1% TFA. Resulting peptides were analyzed using nano-LC-ESI-MS/MS timsTOF Pro (Bruker) coupled with a nanoElute ultra-high performance liquid chromatography. Data were acquired using 100 ms trapped ion mobility spectrometry accumulation time and mobility (1/K0) range from 0.6 to 1.6 Vs/cm^2^. Mass spectrometric analyses were carried out using PASEF acquisition method (35). One MS spectrum followed by ten PASEF MS/MS spectra per total cycle of 1.1 s. The capillary voltage was set to 1400 V, and both MS and MS/MS spectra were acquired in the range of 100 to 1700 *m/z*. Collision energy was linearly ramped as a function of mobility, starting at 59 eV at 1/K0 = 1.6 Vs/cm^2^ and gradually decreasing to 20 eV at 1/K0 = 0.6 Vs/cm^2^. The target value was set to 20,000 arbitrary units. Tandem mass spectra were extracted, charge state deconvoluted, and deisotoped by Data analysis version 5.3 (Bruker).

Protein identification from the MS data was realized with the Andromeda peptide database search engine integrated into the computational proteomics platform MaxQuant version 1.6.3.4 (Max Planck Institute of Biochemistry) (https://maxquant.net/maxquant/) (37) with default search settings including a false discovery rate set at 1% on both the peptide and the protein level. Spectra were searched against *R. prolixus* proteins (UniProt Tax ID: 13249) in the UniProt/Swiss-Prot reference database (UniProt Proteome ID: UP000015103) (14,966 entries) and the decoy database. Andromeda search parameters for protein identification specified a first search mass tolerance of 20 ppm and a main search tolerance of 4.5 ppm for the parental peptide. Enzyme specificity was set to C terminal to arginine and lysine with a maximum of two missed cleavages. Carbamidomethyl of cysteine was specified as a fixed modification. Oxidation of methionine and acetyl of the N terminus were specified in Mascot as variable modifications.

Variable modifications were set to oxidation of methionine and acetylation of protein N termini. A minimum of one unique peptide was required for protein identification. Proteins were quantified by the MaxLFQ algorithm integrated in the MaxQuant software. A minimum ratio count of two unique or razor peptides was required for quantification. Further data analysis was performed with the Perseus software version 1.6.2.1 (Max Planck Institute of Biochemistry) (https://maxquant.net/perseus/). After loading the protein groups file obtained previously by MaxQuant software. First, proteins identified by site and reverse database hits were removed and label-free quantification values were log2-transformed to achieve normal data distribution. Missing values were imputed with zero. The relative expression of aspartic proteases was calculated by dividing their expression intensity by the total intensities of proteins identified in the sample. This allowed comparisons with relative intensities of aspartic proteases identified previously (11).

### Statistical Analysis

Data were analyzed by one way ANOVA, followed by a subsequent Dunnett’s test for post hoc comparisons between treatments and the control group. In all cases, differences were considered significant when *p* value ≤0.05.

## Results

### Profiling of *R. prolixus* Digestive Peptidases Activity

**Fig. 1:**
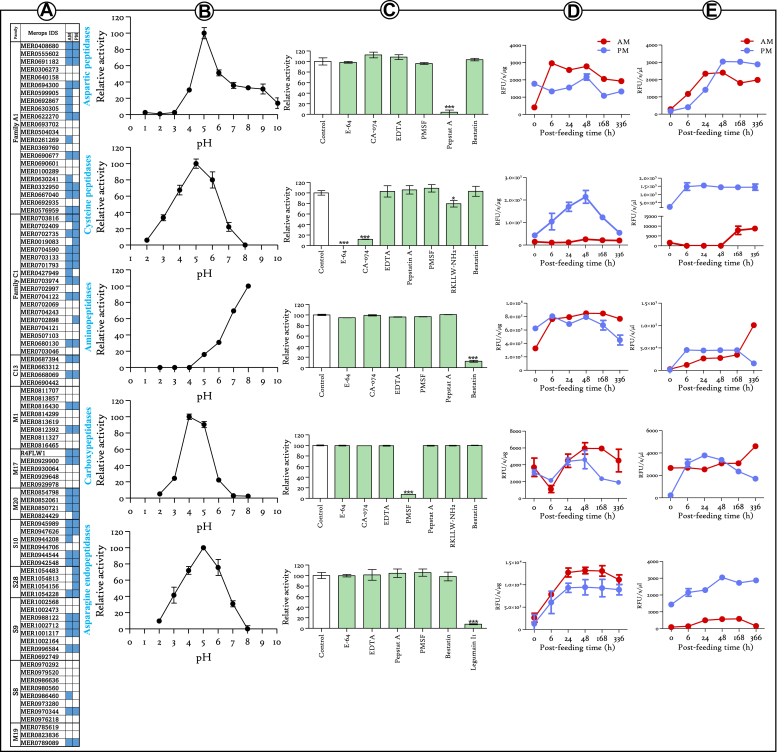
**Enzymatic activity profiling of *Rhodnius prolixus* midgut peptidases.***A*, list of peptidases previously validated for their expression in the insect's digestive tract (AM and PM) through proteomic analysis. *B*, the pH profiles of the identified peptidase families. The activity was measured using specific substrates and represented as relative activity in relation to the maximum detected activity. *C*, the inhibitory profiles of each protease family were obtained by assessing their activity at their respective optimal pH in the presence of selective inhibitors. The obtained values were then represented as a percentage of activity relative to the control. *D*, activity patterns of the peptidase families in the tissue extracts of AM and PM from unfed insects (0), as well as at different time points after feeding. The enzymatic activity was measured using specific substrates at their respective optimum pH and quantified as relative fluorescence units (RFU) per second per microgram of tissue extract. *E*, activity patterns of the peptidase families within the digestive luminal contents of the AM and PM of unfed (0) and different time points postfeeding. The measured activity is expressed in RFU per second per microliter of luminal content. The results are expressed as the mean ± SEM (n = 3 for all experiments), and statistical significance is shown by ∗*p* ≤ 0.05, ∗∗*p* ≤ 0.01, and ∗∗∗*p* ≤ 0.001. AM, anterior midgut; PM, posterior midgut.

The genome analysis of *R. prolixus* has unveiled the existence of 378 putative protease-coding genes, distributed across 65 distinct families (available on MEROPS, accessed on June, 2023). Given the substantial amount of blood (predominantly comprised of proteins) ingested by the insect in a single meal, it is conceivable that the primary role of these proteases is the digestion of blood proteins. Indeed, investigation of the insect's digestive tract proteome has successfully corroborated the expression of 52 proteases (11) belonging to eleven families within the digestive tract tissues (Fig. 1*A*). The investigation of these putative digestive proteases entailed assessment encompassing the determination of their pH activity profiles (Fig. 1*B* and Supplemental Table S1), their responsiveness to protease inhibitors (Fig. 1*C* and Supplemental Table S2), and the temporal dynamics of their activity in AM and PM tissues (Fig. 1*D* and Supplemental Table S3) and luminal contents (Fig. 1*E* and Supplemental Table S3) using specific substrates. Regarding the aspartic proteases, the proteolytic cleavage of Arg-Gly-Phe-Phe-Pro-4M2N is accomplished over a broad pH range, with an optimal catalytic activity at pH 5 (Fig. 1*B*). The degradation of the substrate is significantly and specifically inhibited after treatment of the midgut extract with pepstatin A (Fig. 1*C*). Following blood ingestion in the AM, the tissular activity of these proteases undergoes a substantial induction at 6 h postfeeding and maintaining stability up to 48 h (Fig. 1*D*). Thereafter, a gradual decline of activity is observed at 14 days, although it remained notably higher than unfed insects. In contrast, the tissular activity in the PM is comparatively less pronounced than that observed in the AM (Fig. 1*D*). However, a subsequent increase is noted at 48 h postfeeding, followed by a decline at day 7 (168 h). The luminal activity of these proteases exhibits an evident increase following blood ingestion, both in the AM and PM (Fig. 1*E*). In the AM, the activity stabilizes between 24 and 48 h, subsequently declining at day 7, and then remains stable until day 14 (336 h). Conversely, the activity of these proteases in the PM continues to increase until 48 h postfeeding, subsequently stabilizing at a higher level than that detected in the AM, throughout the kinetic period (Fig. 1*E*). The peptidases from the C1 family stand out as widely expressed enzymes within the digestive tissues (Fig. 1*A*). This family encompasses cathepsins B- and L-like cysteine proteases. Evaluating their pH-dependent activity on the fluorogenic substrate Z-Phe-Arg-AMC revealed a remarkable stability across a broad pH range from 3 to 7, with an optimal activity at pH 5 (Fig. 1*B*). The degradation of Z-Phe-Arg-AMC is meaningfully inhibited by E-64 (Fig. 1*C*). Moreover, the predominant hydrolysis of the substrate was ascribed to cathepsins B-like cysteine proteases, based on their substantial sensitivity to CA-074 (Fig. 1*C*). The remaining activity, which is insensitive to CA-074, was effectively inhibited by RKLLW-NH2, a bespoke inhibitor of cathepsins L-like cysteine proteases. The kinetic evaluation of the activity of these proteases, both in the tissues (Fig. 1*D*) and luminal contents (Fig. 1*E*) of the AM and PM, revealed a notable disparity between the two tissues. Indeed, a consistent and unchanged basic activity was detected in the AM tissue extract over time, while in the PM, their activity increased after feeding and continued to rise until 48 h, after which it declined and returned to its initial level at day 14 (Fig. 1*D*). Concurrently, their activity in the digestive content remained basic in the AM until 48 h and then slightly increased at day 7, remaining stable until day 14 (Fig. 1*E*). In contrast, the activity in the PM content underwent induction, following feeding and remained stable throughout the kinetic period (Fig. 1*E*). Among the other cysteine proteases expressed in the midgut, we have identified two asparagine endopeptidases belonging to the C13 family (Fig. 1*A*). They exhibit a pH profile similar to that of C1 family (Fig. 1*B*). However, they are not sensitive to E-64 but their ability to hydrolyze Z-Ala-Ala-Asn-AMC is significantly affected by legumain I1 inhibitor confirming the specificity of the substrate (Fig. 1*C*). Remarkably, the activity profile detected in both AM and PM is highly similar, with an induction of activity following blood ingestion that culminate at 24 h remaining steady until day 14 (Fig. 1*D*). In the lumen, the activity is substantially higher in the PM than in the AM, where a basic activity was detected over the studied kinetic time (Fig. 1*E*).

Several aminopeptidases of diverse families are expressed in the digestive tissues (Fig. 1*A*). Monitored using Leu-AMC substrate, they exhibited an optimum activity at pH 8 (Fig. 1*B*) with significant inhibition by bestatin (Fig. 1*C*). Their temporal activity profile in the AM revealed induction by blood ingestion in tissue (Fig. 1*D*) and content samples (Fig. 1*E*). Additionally, a sharp increase in luminal activity occurred at day 14 (Fig. 1*E*). Conversely, in the PM, the activity gradually declined over time in the tissue, returning to prefeeding detected activity (Fig. 1*D*). In the lumen, their activity remained stable until day 7, followed by a decrease at day 14 (Fig. 1*E*).

In the same context, various carboxypeptidases have been identified (Fig. 1*A*). Their activity on Z-Phe-Leu-OH remained stable over a wide range of pHs (3–6) and significantly affected by PMSF (Fig. 1, *B* and *C*). Examination of their temporal dynamics showed a notable decline in tissular activity in the AM tissue at 6 h postfeeding, followed by a subsequent increase up to 48 h, and eventually a decline at day 7 (Fig. 1*D*). Conversely, in the PM, tissular activity exhibited an initial surge postfeeding, reaching its peak at 48 h, and subsequently returning to its prefeeding level (Fig. 1*D*). Regarding luminal activity, the AM demonstrated a sustained level until day 7 postfeeding similar to unfed condition, followed by an upsurge at day 14. In contrast, the luminal activity in the PM increased immediately postfeeding, remaining high up to 48 h, and then underwent a continuous decrease (Fig. 1*E*).

### Hb Digestion is Orchestrated by Cross-Functional Peptidases

**Fig. 2:**
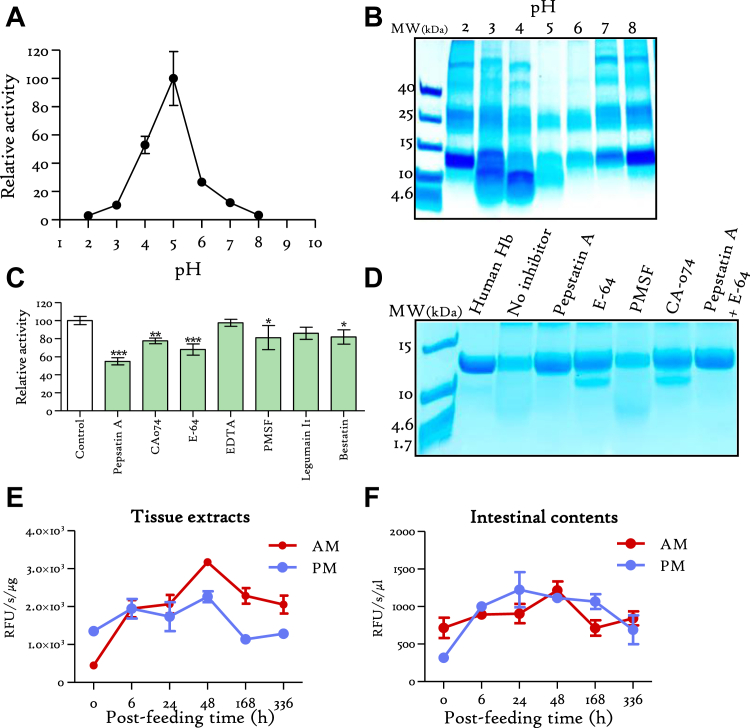
**Human Hb digestion by *Rhodnius prolixus* midgut-associated peptidases.***A*, the pH profile of Hb digestion by midgut luminal content peptidases was quantified by derivatization of fluorescamine. *B*, SDS-PAGE visualization of the effect of the pH on *in vitro* hemoglobinolysis by midgut luminal contents. *C*, effect of midgut extracts on Hb hydrolysis in the presence of different protease inhibitors. *D*, Hb digestion products by midgut luminal content treated or not with individual protease inhibitors was visualized by SDS-PAGE after Coomassie blue staining. *E*, Hb digestion by AM and PM tissue extracts. *F*, Hb digestion by AM and PM luminal contents from unfed (0) and different postfeeding time points. The results are expressed as the mean ± SEM (n = 3 for all experiments) and statistical significance is shown by ∗*p* ≤ 0.05, ∗∗*p* ≤ 0.01, and ∗∗∗*p* ≤ 0.001. AM, anterior midgut; Hb, hemoglobin; PM, posterior midgut.

Human Hb digestion by *R. prolixus* digestive proteases was measured in various experimental contexts. This was achieved through quantification of the hydrolysis rate using fluorescamine derivatization, as well as visualization of Hb digested fragments on SDS-PAGE. Interestingly, the assessment of the pH effect on Hb degradation reveals that its digestion occurs between pH 3 and 7, with the most effective hemoglobinolysis occurring at pH 5. (Fig. 2*A* and Supplemental Table S4). The electrophoretic profile exhibiting the pH effect after 16 h digestion of Hb (α and β subunits: 15.2 and 15.9 kDa, respectively) shows a slight degradation at pH 3 and 4, resulting in the formation of large fragments predominantly above 10 kDa. Hb is almost completely digested at pH 5 and subsequently decreases progressively as the pH is raised. At pH 8, the digestion is entirely inhibited, with a pattern similar to that observed at pH 2 (Fig. 2*B*). Then, the implication of specific peptidase on Hb digestion was assessed by employing selective peptidase inhibitors to scrutinize their distinct roles in this process. The fluorescamine derivatization experiment revealed that the addition of pepstatin A and E-64 separately, drastically alters the rate of Hb digestion (*p* ≤ 0.001) by around 54% and 32%, respectively (Fig. 2*C* and Supplemental Table S5). Since, the selective inhibition of cathepsin B-like proteinases by CA-074 is about 23% (*p* ≤ 0.01), it suggests that cathepsin L-like proteases are responsible for the remaining 10% of hemoglobinolysis. Moreover, inhibition of aminopeptidases and carboxypeptidases by bestatin and PMSF, respectively, significantly affected Hb digestion by around 20% (*p* ≤ 0.05). The addition of legumain I1, a specific inhibitor of asparagine endopeptidases, and EDTA did not show a significant impact on Hb hydrolysis (Fig. 2*C* and Supplemental Table S5). The visualization of Hb digest on gel confirms that it is mainly digested by pepstatin A and E-64 sensitive proteases (Fig. 2*D*). Indeed, the combination of these two inhibitors blocks completely the digestion. Moreover, the electrophoretic profiles observed for the samples treated with E−64 and CA-074 are highly similar, confirming the weak contribution of cathepsin L-like proteases in Hb hydrolysis. Similarly, the pattern obtained for PMSF is almost indistinguishable from the control with no inhibitor, confirming the minimal contribution of carboxypeptidases to the digestive process (Fig. 2*D*).

Hb degradation by tissue extracts (Fig. 2*E*) and luminal content (Fig. 2*F*) from both AM and PM at various time points postfeeding was assessed at the optimal pH 5 (Supplemental Table S6). Tissular proteolysis of Hb by the AM shows a two peaks increase at 6 h to reach a maximum at 48 h postfeeding before gradually declining until day 14. A similar, but less pronounced, profile is observed for PM extracts (Fig. 2*E*). The dynamic of Hb digestion by luminal contents exhibits a comparative pattern. Indeed, a continuous increment of the digestion rate by AM content is observed after feeding, peaking at 48 h, and then dropping at day 7. The PM manifests a more pronounced induction of hemoglobinolytic activity at 6 h postfeeding, remaining higher than unfed condition along the studied feeding kinetic (Fig. 2*F*).

### Cleavage Site-Specificity of Major *R. prolixus* Hemoglobinases

**Fig. 3:**
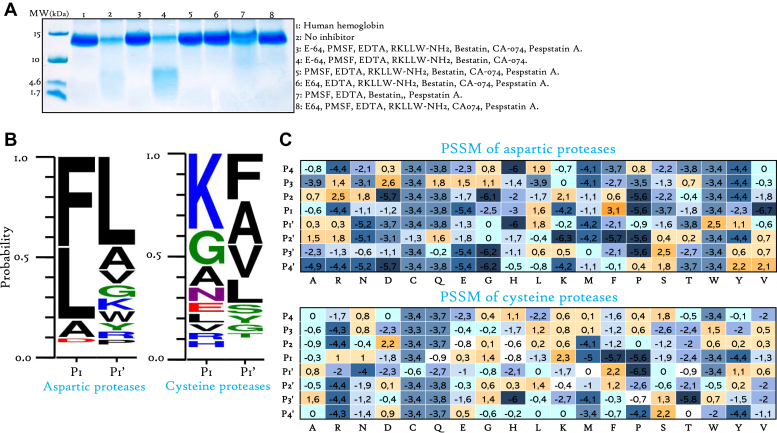
**Hb cleavage site-specificity of major midgut-associated peptidases.***A*, contribution of individual peptidase in Hb digestion was assessed by treating luminal digestive content with a cocktail of inhibitors, allowing to concurrently preserve the activity of a specific protease family. The digestion was conducted for 16 h, and the degradation pattern of Hb was visualized on a 10 to 20% Tris-Tricine gel. *B*, peptidase-specific digests of Hb by aspartic and cysteine proteases were subjected to mass spectrometry analysis. The examination of these peptide sequences allowed the establishment of sequence logo representing P1-P1′ subsite specificities of these proteases. *C*, the PSSM representing cleavage site preferences of aspartic and cysteine proteases generated according to the alignment of sequenced peptides. The scores presented within the PSSM matrix correspond to the log-odds, which represents the log 2 of the frequency of occurrence of an amino acid in a specific position normalized by the background frequency of each amino acid. The background frequency corresponds to the relative abundance of the amino acid within the hemoglobin molecule (Supplemental Table S8). PSSM, position-specific scoring matrix.

Inhibition of Hb digestion by adding individual selective protease inhibitors demonstrate that both aspartic and cysteine proteases play a major role in Hb cleavage (Fig. 2, *C* and *D*). A detailed investigation of their individual action on Hb was conducted by treating digestive content from 48 h postfed insects (where the highest activity of these proteases as well as hemoglobinolysis was detected) (Fig. 2*F*) with a cocktail of inhibitors: E-64, PMSF, EDTA, RKLLW-NH2, bestatin, CA-074, and pepstatin A. This allowed to concurrently preserve the activity of a specific protease family. Hb digestion was conducted for 16 h, as described previously, and the degradation pattern of Hb was observed on a 10 to 20% Tris-Tricine gel. Interestingly, exclusion of pepstatin A from the cocktail, led to a significant and almost complete enzymatic proteolysis, with generation of bands between 10 and 1.7 kDa (Fig. 3*A*, lane 4). The electrophoretic profile is similar to the control where no inhibitor was added. On the other hand, keeping cysteine-like proteases active by excluding E-64 promotes Hb degradation but with a less pronounced effect than aspartic proteases (Fig. 3*A*). Subsequently, a complete inhibition of digestion is observed when pepstatin A and E-64 are added simultaneously (Fig. 3*A*, lane 3). Moreover, the exclusion of PMSF and bestatin individually does not appear to promote Hb degradation.

In order to illustrate the cleavage site preferences of these key hemoglobinases, we employed functional proteomics. The combination of a selective cocktail of inhibitors facilitated the generation of peptidase-specific digests of Hb by aspartic and cysteine proteases. Subsequently, the fragments resulting from the specific digestion were subjected to MS analysis. The examination of these peptide sequences (Supplemental Table S7) allowed us to establish the graphical representation illustrating the occurrence of cleavage events at specific amino acid positions (Fig. 3*B*). The results demonstrate that aspartic proteases preferentially cleave between hydrophobic amino acids at the P1-P1′ position, with a notable affinity for F, L, and A at the P1 site, and a pronounced preference for L at the P1′ site (approximately 50%) (Fig. 3*B*). Undoubtedly, cleavage by aspartic proteases could also occurs at A, V, G, K, W, Y, R, and P in P1′ position.

In order to provide a comprehensive representation of human Hb degradation by these peptidases, position-specific scoring matrix was created based on sequenced peptides, along with the distribution of amino acids within the protein (Supplemental Table S8). Indeed, the experimentally obtained occurrence frequencies of each amino acid were normalized by dividing them by their relative abundance within the Hb molecule. This normalization process allowed for a more accurate assessment, ensuring that the occurrence scores considered the relative prevalence of each amino acid within the Hb sequence. This facilitated the determination of P4-P4′ specificity across the calculation of occurrence scores for different amino acids at each position (Fig. 3*C*). Taking into consideration, this matrix has allowed to conclude that the probability of Hb cleavage by aspartic proteases at W and Y in P1′ position is significantly considerable giving the low abundance of these residues within the protein. Outside the P1P1′ site, aspartic proteases tolerate different residues but display a preference for L at P4, D at P3, R at P2 and P2′, S at P3′, and Y/V at P4′.

Cysteine proteases sensitive to E-64 demonstrate less specificity, leading to a broader range of cleavage sites across different amino acids at positions P1, despite K and G being privileged (Fig. 3*B*). However, they exhibit a clear preference for hydrophobic amino acids (F, A, V, and L) at P1′ (Fig. 3*B*).

### Quantitative Proteomics Enables Deep Profiling of Midgut-Associated Aspartic Proteases

**Fig. 4:**
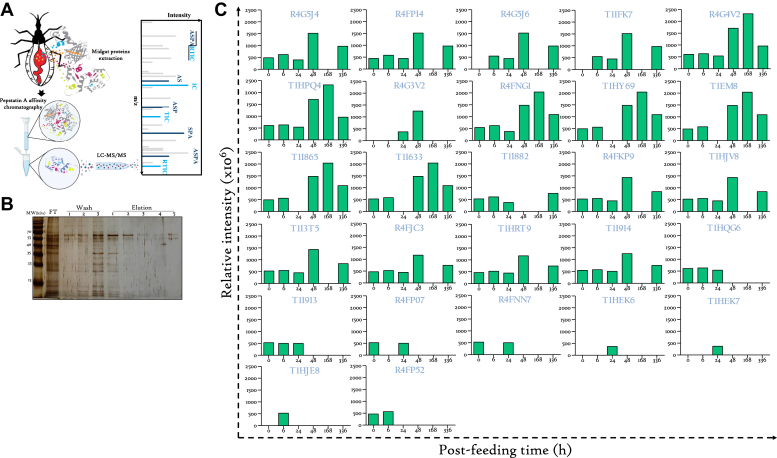
**Deep profiling of midgut-associated aspartic proteases.***A*, workflow of aspartic proteases purification on pepstatin A affinity column prior to their analysis by LC-MS/MS. *B*, SDS-PAGE analysis of the purification fractions on Tris-Tricine 12% gel (flowthrough fraction (FT) from pepstatin A column, wash fractions, and elution fractions). *C*, monitoring of the temporal dynamics of aspartic proteases (identified by their respective accession numbers) expression by proteomics. The relative expression of each isoform was calculated by normalizing its intensity to the intensity of the total identified proteins.

Given the pivotal importance of aspartic proteases in the digestive process, particularly in Hb processing, special attention has been dedicated to them. Interestingly, these genes appear to encode for 32 protein isoforms (available on UniProt database). Upon exploration of transcript expression within the digestive tissues, a total of 14 aspartic proteases were identified, exhibiting a predominant expression in the AM (13). Among them, ten have been validated at the protein level as midgut-associated peptidases (11), and intriguingly, the expression of seven isoforms in the AM was induced postprandially (12). In the pursuit of gaining comprehensive insights into the involvement of these proteases in digestive physiology of *R. prolixus*, as well as the significance of their isoforms diversity in the digestion process, they have been purified from the AM of unfed as well as engorged insects at various postprandial time points using pepstatin A affinity chromatography (Fig. 4*A*).

The temporal expression pattern of different isoforms was monitored by quantitative proteomics. This allowed the identification of 27 isoforms (Fig. 4*C*). Strikingly, many isoforms exhibited similar expression profiles. This is the case of R4G5J4 with R4FPI4, R4G5J6 with T1IEK7, R4G4V2 with T1HPQ, T1HY69 with T1IEM8, T1I865 with T1I633, R4FKP9 with T1HJV8, R4FJC3 with T1HRT9, R4FP07 with R4FNN7, and T1HEK6 with T1HEK7 (Fig. 4*C* and Supplemental Table S9). The expression pattern and the significant difference in the lengths of some isoforms strongly imply that they may represent fragmented portions of the same protein, likely originating from a common gene.

### *In Vivo* Mapping of Hb Proteolysis

**Fig. 5:**
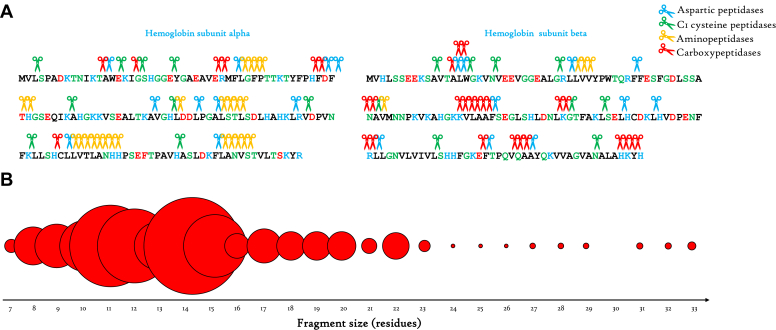
***In vivo* mapping of hemoglobin proteolytic cleavage.***A*, depiction of cleavage sites by various peptidases based on the sequences of rabbit Hb-derived fragments identified through mass spectrometry within the digestive content of the AM after 14 days postfeeding. *B*, the size distribution of Hb fragments in the AM at 14 days postfeeding. The fragments are represented by *circles*. The relative size of the *circles* corresponds to the abundance of assigned peptides of the same length. AM, anterior midgut; PM, posterior midgut.

In order to delve into the intricacies of *in vivo* Hb digestion within the AM, we employed MS to identify Hb-derived peptides generated through the action of various peptidases at 14 days postfeeding. To accomplish this, the extracted luminal digestive content from the AM was subjected to treatment with a protease inhibitor cocktail to prevent any further proteolysis postextraction, as detailed in the methods section. The digestion products were subsequently identified using MS (Supplemental Table S10). Drawing upon the sequences of the identified peptides, as well as the previously characterized cleavage sites for aspartic and cysteinyl proteases, we were able to construct a cleavage map of α- and β-subunits of Hb molecule (Fig. 5*A*). The findings elucidate a sequential cascade wherein Hb fragments undergo an initial cleavage by aspartic proteases, marking the inception of Hb fragmentation. These precursor fragments, once generated, undergo truncation by cysteine proteases before being concomitantly processed by amino and carboxypeptidases from both ends. At 14 days of the digestive process, aspartic proteases–generated fragments emerge as a minor fraction (Fig. 5*B*). These fragments are predominantly cleaved into smaller constituents, typically ranging between 10 and 16 residues. This subsequent array of intermediate fragments undergoes further cleavage, primarily orchestrated by aminopeptidases (Fig. 5*A*), culminating in the formation of even smaller fragments. It is important to note that the MS detector constraints imposed a lower mass threshold for detection, precluding the identification of fragments below than seven residues. Nonetheless, Hb cleavage map empowers us to extrapolate and assert a substantial release of amino acids within the AM.

## Discussion

Notwithstanding the significant medical importance of triatomines, and all investigations that have been conducted on *R. prolixus* to comprehend various facets of insect physiology (38, 39), little is definitively known about their digestion (40). Indeed, it was conventionally accepted that blood proteins digestion occurs exclusively within the PM, relegating the AM to a mere storage organ (41). These assertions were founded on observations of tissues structure and ultrastructure along the digestive tract (5, 6, 7, 8). However, the availability of *R. prolixus* genome (42) and the application of postgenomic technologies (11, 13, 43) have provided a comprehensive view of the insect's digestive tissues physiology, revealing minimal molecular differences between the AM and the PM (11). Furthermore, the comprehensive analysis of protein expression has substantiated the presence of diverse protease families, notably within the AM (12), challenging the prior notion of its limited functionality. The detection of enzymatic activities of various peptidases within the AM (12, 15) underscores the dynamic nature of this organ and prompt a reevaluation of its contribution to the overall digestive process.

In this work, we have delineated the involvement of various protease families in Hb digestion pathway, a predominant protein constituent of the blood meal. Initially, we conducted a comprehensive activity profiling of the distinct protease families. This was accomplished utilizing specific substrates and inhibitors. This endeavor not only validated the enzymatic activity of aspartic, cysteine, carboxy, and aminopeptidases but also facilitated the monitoring of their tissue-specific and luminal activity profiles throughout the digestive process (Fig. 1). From our findings, we deduce that the activity of the various secreted proteases is comparable between the AM and the PM, with the exception of cysteine proteases (Fig. 1*E*). Notably, cysteine proteases exhibit a modest activity in the AM, yet they display a discernible escalation after day 7. We have designated cathepsin L- and B-like proteases as cysteine proteases, even though asparagine endopeptidases fall within this family as well. Intriguingly, we are the pioneers in delineating the expression and corroborating the activity of asparagine endopeptidases in triatomines. The latter have demonstrated more pronounced activity in the PM (comparably to cysteine proteases), thereby unveiling a novel facet of protease dynamics in the context of insect digestion. Regarding aminopeptidases, we believe that the luminal aminopeptidase activity measured *via* L-AMC substrate hydrolysis (Fig. 1*E*) is exclusively accomplished by M1 alanyl aminopeptidase. Indeed, these later have demonstrated a broader specificity, displaying a potent affinity for leucine and a diverse range of amino acids (44). Indeed, this assertion arises from the fact that all predicted M17 leucine aminopeptidases are anticipated to be cytosolic. Inhibition by bestatin failed to discriminate between these two families of exopeptidases. Consequently, the luminal activity previously attributed to M17 leucine aminopeptidase (15, 32, 45) is in fact ensured by M1 alanyl aminopeptidases (both inhibited by bestatin), thus reframing our understanding in this context.

Results of *in vitro* digestion of Hb by intestinal content extracts revealed that optimal hemoglobinolysis occurs at pH 5 (Fig. 2*A*). This observation is noteworthy given that the pH of the midgut of unfed triatomines approximates 6.3 and 6.5 in the AM and PM, respectively. However, blood-feeding induces pH drop, which reported to attain 5.2 in both tissues (28), potentially due to hemolysis. The quantification of the contribution of individual peptidase family in this process, indicated that hemoglobinolysis is predominantly orchestrated by the concerted actions of aspartic and C1 cysteine proteases (Fig. 2*C*). Intriguingly, the application of pepstatin A to the luminal digestive content effectively abrogated hemoglobinolysis (Fig. 2*C*). Notably, it has been previously substantiated that the ingestion of pepstatin A inhibits blood digestion within the midgut (46). Furthermore, this inhibition significantly impeded molting across all nymphal instars and concurrently exerted a substantial influence on the intricate process of oogenesis in adult stages (46). This confirms the vital role of aspartic proteases in digestion, indicating that Hb precleavage by these enzymes is a crucial processing step, while amino and carboxyexopeptidases exhibited a discernible activity albeit with comparatively lesser contribution. Their involvement remained significant only if aspartic proteases are active (Figs. 2*C* and 3*B*).

The purification of aspartic proteases by pepstatin A affinity chromatography from AM tissues at various time points, followed by quantitative proteomic analysis, enabled the reconstruction of their expression patterns during the digestive process (Fig. 4*C*).

The utilization of MS for identifying Hb-derived fragments generated in the AM through *in vivo* activity of diverse peptidases has revealed that at 14 days the prevailing fragments predominantly comprise 11 to 14 residues (Fig. 5*B*). Through the discernment of preferential cleavage sites of endopeptidases, we have delineated the pathway of Hb digestion. Specifically, the aspartic proteases undertake the initial cleavage of Hb molecules, acting preferentially between hydrophobic amino acids. This generates relatively larger fragments up to 33 residues. Subsequently, these precursor fragments undergo further truncation through the action of cysteine proteases, notably the cathepsin B-like peptidases, which exhibit heightened activity within this enzyme family. The precursor fragments are then simultaneously processed by amino and carboxypeptidases (Fig. 5*B*). The ultimate products of digestion consist of fragments spanning 7 to 10 residues, accompanied by a substantial release of amino acids primarily due to the concerted action of exopeptidases (Fig. 5*B*). This aligns harmoniously with our previous observations pertaining to the regulation of proteins implicated in amino acid metabolism within the AM (12). Furthermore, RNAi-mediated silencing of genes involved in tyrosine detoxification has been demonstrated to induce the formation of tyrosine crystals within the AM, ultimately leading to insect mortality (47). This lends further support to our observations concerning the release of amino acids within the AM. We postulate that the culmination of digestion is orchestrated by the involvement of additional peptidases, including dipeptidases and monopeptidases.

Our investigation has successfully unveiled the intricate dynamics of Hb digestion orchestrated by a network of gut-associated peptidases, distinctly elucidating the involvement of the AM in the digestive process of this prominent blood protein. This pioneering endeavor marks the foremost investigation to attain a comprehensive and precise overview of the digestive process in triatomines. Conceivably, this revelation holds promise in ushering in new prospects for vector control strategies and disrupting the transmission of *T. cruzi*, which takes advantage of this digestive system to seize its vital nutrients.

## Data Availability

MS data have been deposited into ProteomeXchange Consortium *via* PRIDE repository (48): *In vivo* digestion of rabbit Hb by *R prolixus* midgut-associated peptidases: PXD044590 reviewer_pxd044590@ebi.ac.uk.

MS-based determination of *R. prolixus* hemoglobinases cleavage preferences PXD044620 reviewer_pxd044620@ebi.ac.uk.

Expression profile of *R*. *prolixus* midgut-associated aspartic proteases: PXD044628 reviewer_pxd044628@ebi.ac.uk and MS-viewer search key: 45wcqfzmlt.

All proteins and peptides identified in the MS/MS analyses are available in Supplemental Table S11.

## Supplemental data

This article contains supplemental data.

## Conflict of interest

The authors declare that they have no competing interests.
