# Supplementary material for: Unveiling the Peptidase Network Orchestrating Hemoglobin Catabolism in Rhodnius prolixus
Source: Mol Cell Proteomics. 2024 Apr 23;23(6):100775. doi: 10.1016/j.mcpro.2024.100775 (PMC11135036; doi:10.1016/j.mcpro.2024.100775)
Supplement: Supplemental Table S1 [file mmc1.pdf]

Table S1:The pH profiles of *Rhodnius prolixus* peptidase families

A1 aspartic peptidases

Time (s)	pH	RFU						RFU/S		Relative activity	
		Exp1	Exp2	Exp3	Exp (x̄)	SD	Blank (x̄)	Exp (x̄)	SD	Exp (x̄)	SD
5100	**1**	2138259	1563035	1307203	1669499	425634,18	1160322	99,8386	83,4577	2,630353	2,19878
5100	**2**	797426	675863	1336156	936481,67	351424,35	746950,7	37,1629	68,9067	0,979097	1,8154201
5100	**3**	1143857	1152381	1245701	1180646,3	56499,972	657498,7	102,578	11,0784	2,702524	0,2918727
5100	**4**	6287540	6887196	7013643	6729459,7	387900,7	858785	1151,11	76,059	30,32727	2,003853
5100	**5**	19280726	20736178	21964114	20660339	1343300,6	1302597	3795,64	263,392	100	6,9393451
5100	**6**	10480800	11737785	11641190	11286592	699505,4	1372075	1944,02	137,158	51,21732	3,6135691
5100	**7**	8400863	7974958	9384551	8586790,7	722955,69	1643769	1361,38	141,756	35,8669	3,7347107
5100	**8**	8909921	8800448	8429126	8713165	252001,39	2347949	1248,08	49,412	32,88201	1,3018119
5100	**9**	7228297	7076229	9175971	7826832,3	1170859,7	1745426	1192,43	229,58	31,41589	6,0485345
5100	**10**	5881716	3370526	4727092	4659778	1256947,6	1945737	532,165	246,46	14,02044	6,4932549

C1 cysteine peptidases

Time (s)	pH	RFU						RFU/S		Relative activity	
		Exp1	Exp2	Exp3	Exp (x̄)	SD	Blank (x̄)	Exp (x̄)	SD	Exp (x̄)	SD
3900	**1**	64988196	72001688	71315184	69435023	3866331,9	82307315	-3300,59	991,367	-0,7269	0,2183333
3900	**2**	175928592	190408480	213244304	193193792	18813135	87267483	27160,6	4823,88	5,981701	1,0623852
3900	**3**	620238784	667091200	746558400	677962795	63857694	83124360	152523	16373,8	33,59076	3,6060693
3900	**4**	1160321280	1,34E+09	1348195584	1,283E+09	106160409	86540624	306736	27220,6	67,5539	5,9949205
3900	**5**	1742041216	1,884E+09	1933940864	1,853E+09	99587813	82550467	454061	25535,3	100	5,6237634
3900	**6**	1401776256	1,687E+09	1365436928	1,485E+09	175983888	67053184	363491	45124,1	80,05327	9,9378801
3900	**7**	441561184	568897920	382039744	464166283	95458040	69102253	101298	24476,4	22,30942	5,3905534
3900	**8**	47292904	36880776	33911424	39361701	7027250,1	36569108	716,05	1801,86	0,157699	0,3968316
3900	**9**	57057884	61725784	61426896	60070188	2613008,8	67322511	-1859,57	670,002	-0,40954	0,1475576
3900	**10**	63527452	65051824	65248660	64609312	942073,19	62961216	422,589	241,557	0,093069	0,0531992

Aminopeptidases

Time (s)	pH	RFU						RFU/S		Relative activity	
		Exp1	Exp2	Exp3	Exp (x̄)	SD	Blank (x̄)	Exp (x̄)	SD	Exp (x̄)	SD
2400	**2**	74155192	75431816	74348344	74645117	688111,92	74230133	172,91	286,713	0,015803	0,0262047
2400	**3**	83757448	81475144	81455640	82229411	1323355,1	79706373	1051,27	551,398	0,096082	0,0503961
2400	**4**	83565280	84872648	86080424	84839451	1257900,6	81442555	1415,37	524,125	0,129361	0,0479035

2400	**5**	505706984	506494304	506513568	506238285	460221,26	85449875	175329	191,759	16,02449	0,0175262
2400	**6**	625732352	656444224	647942464	643373013	15857638	93969304	228918	6607,35	20,92243	0,6038917
2400	**7**	1964520320	1,895E+09	1925789696	1,928E+09	34944441	1,02E+08	761070	14560,2	69,5595	1,3307567
2400	**8**	2732412672	2,693E+09	2748841216	2,725E+09	28756379	98808563	1094128	11981,8	100	1,0951025
**Carboxypeptidases**											
Time (s)	**pH**	**RFU**						**RFU/S**		**Relative activity**	
		**Exp1**	**Exp2**	**Exp3**	**Exp (X̅)**	**SD**	**Blank (X̅)**	**Exp (X̅)**	**SD**	**Exp (X̅)**	**SD**
5100	**2**	2690168	2955565	2854862	2833531,7	133978,09	1223519	315,689	26,2702	5,19462	0,4322733
5100	**3**	8699905	8954380	8846387	8833557,3	127721,7	1301290	1476,92	25,0435	24,30246	0,4120874
5100	**4**	32218842	32831064	34238536	33096147	1035612,3	2102307	6077,22	203,061	100	3,3413488
5100	**5**	33394234	33505302	31460682	32786739	1149741,3	4772184	5493,05	225,439	90,38749	3,70958
5100	**6**	8470879	8287436	8434703	8397672,7	97166,186	1491663	1354,12	19,0522	22,28188	0,3135016
5100	**7**	2402111	2255622	2536397	2398043,3	140431,69	1482240	179,569	27,5356	2,95479	0,4530955
5100	**8**	2330016	2249536	2270699	2283417	41720,118	1528117	148,098	8,18042	2,436935	0,1346078
**Asparagine endopeptidases**											
Time (s)	**pH**	**RFU**						**RFU/S**		**Relative activity**	
		**Exp1**	**Exp2**	**Exp3**	**Exp (X̅)**	**SD**	**Blank (X̅)**	**Exp (X̅)**	**SD**	**Exp (X̅)**	**SD**
3600	**2**	65923800	65924552	65991048	65946467	38610,398	65474424	131,123	10,7251	9,705766	0,7938763
3600	**3**	66627140	67194208	66246760	66689369	476779,61	64664724	562,401	132,439	41,62915	9,8031637
3600	**4**	70487728	70488912	70898600	70625080	236876,01	67121776	973,14	65,7989	72,03215	4,8704563
3600	**5**	72151544	72152856	72105808	72136736	26792,466	67273208	1350,98	7,44235	100	0,5508854
3600	**6**	76027376	76698880	75759872	76162043	483771,99	72477368	1023,52	134,381	75,76135	9,9469355
3600	**7**	66908224	66548288	66754064	66736859	180583,77	65230204	418,515	50,1622	30,97864	3,7130201
3600	**8**	68751736	69072312	68748488	68857512	186029,35	68852760	1,32	51,6748	0,097707	3,8249877
