# Supplementary material for: Unveiling the Peptidase Network Orchestrating Hemoglobin Catabolism in Rhodnius prolixus
Source: Mol Cell Proteomics. 2024 Apr 23;23(6):100775. doi: 10.1016/j.mcpro.2024.100775 (PMC11135036; doi:10.1016/j.mcpro.2024.100775)
Supplement: Supplemental Table S2 [file mmc2.pdf]

Table S2: Profiling of peptidases activity in presence of selective inhibitors

**A1 aspartic peptidases**												**One-way ANOVA test: P<0.0001**				
**Time (s)**	**Inhibitor**	**RFU**						**RFU/S**		**Relative activity**		**Dunnett's Multiple**	**Mean Diff.**	**q**	**Significant? P < 0.01?**		**99% CI of diff**
		**Exp1**	**Exp2**	**Exp3**	**Exp (x̄)**	**SD**	**Blank (x̄)**	**Exp (x̄)**	**SD**	**Exp (x̄)**	**SD**						
3000	Control	5358556	5430210	5644456	5477741	148758	3304655	724,36	49,5861	100	6,845						
3000	CA074	5692990	5456368	5597320	5582226	119031	3142363	813,29	39,677	112,276	5,478	Control vs CA-	-266800	3,527	No	*	-551400 to 17850
3000	E64	5391962	5320719	5336975	5349885	37335	3211311	712,86	12,445	98,4119	1,718	**Control vs E64**	34510	0,4562	No	ns	-250100 to 319100
3000	Pepstatin A	3397030	3218783	3272410	3296074	91449	3210986	28,363	30,4831	3,91557	4,208	Control vs Pep	2088000	27,60	Yes	***	1803000 to 2373000
3000	EDTA	5438966	5411602	5597712	5482760	100487	3131242	783,84	33,4957	108,211	4,624	Control vs EDT	-178400	2,359	No	ns	-463100 to 106200
3000	PMSF	5382231	5316398	5315992	5338207	38126	3249254	696,32	12,7088	96,1284	1,754	Control vs PM	84130	1,112	No	ns	-200500 to 368800
3000	Bestatin	5524875	5479943	5425528	5476782	49749	3227332	749,82	16,583	103,514	2,289	Control vs Bes	-76360	1,010	No	ns	-361000 to 208300

**C1 cysteine peptidases**												**One-way ANOVA test: P<0.0001**					
**Time (s)**	**Inhibitor**	**RFU**						**RFU/S**		**Relative activity**		**Dunnett's Multiple**	**Mean Diff.**	**q**	**Significant? P < 0.05?**		**95% CI of diff**
		**Exp1**	**Exp2**	**Exp3**	**Exp (x̄)**	**SD**	**Blank (x̄)**	**Exp (x̄)**	**SD**	**Exp (x̄)**	**SD**						
1500	Control	1,34E+09	1,39E+09	1,45E+09	1,4E+09	5E+07	88119011	868163	36630,4	100	4,219						
1500	CA074	2,41E+08	2,41E+08	2,48E+08	2,4E+08	4E+06	88119011	103304	2789,94	11,8991	0,321	Control vs CA-	1,1E+09	15,74	Yes	***	934200000 to 1360000000
1500	E64	57094582	57975746	63225147	5,9E+07	3E+06	88119011	-19125	2209,68	-2,2029	0,255	Control vs E-6	1,3E+09	18,26	Yes	***	1118000000 to 1544000000
1500	EDTA	1,26E+09	1,51E+09	1,5E+09	1,4E+09	1E+08	88119011	892553	93585,7	102,809	10,78	Control vs EDT	-4E+07	0,5020	No	ns	-249700000 to 176500000
1500	Pepstatin A	1,35E+09	1,55E+09	1,5E+09	1,5E+09	1E+08	88119011	918128	69952,5	105,755	8,058	Control vs Pep	-7E+07	1,028	No	ns	-288000000 to 138100000
1500	PMSF	1,4E+09	1,54E+09	1,57E+09	1,5E+09	9E+07	88119011	942956	60452	108,615	6,963	Control vs PM	-1E+08	1,539	No	ns	-325300000 to 100900000
1500	Cat L inhibit	1,05E+09	1,21E+09	1,11E+09	1,1E+09	8E+07	88119011	689268	54010,3	79,3939	6,221	Control vs RKL	2,7E+08	3,682	Yes	*	55260000 to 481400000
1500	Bestatin	1,31E+09	1,43E+09	1,55E+09	1,4E+09	1E+08	88119011	894261	82133,8	103,006	9,461	Control vs Bes	-4E+07	0,5372	No	ns	-252200000 to 173900000

**Aminopeptidases**												**One-way ANOVA test: P<0.0001**					
**Time (s)**	**Inhibitor**	**RFU**						**RFU/S**		**Relative activity**		**Dunnett's Multiple**	**Mean Diff.**	**q**	**Significant? P < 0.001?**		**99.9% CI of diff**
		**Exp1**	**Exp2**	**Exp3**	**Exp (x̄)**	**SD**	**Blank (x̄)**	**Exp (x̄)**	**SD**	**Exp (x̄)**	**SD**						
3300	Control	3,63E+08	3,7E+08	3,69E+08	3,7E+08	3E+06	77664837	87792	1052,4	100	1,199						
3300	Pepsatin A	3,7E+08	3,69E+08	3,69E+08	3,7E+08	584377	77664837	88333	177,084	100,616	0,202	Control vs Pep	-2E+06	0,7566	No	ns	-14190000 to 10610000
3300	CA074	3,64E+08	3,69E+08	3,62E+08	3,6E+08	3E+06	77664837	87037	1007,84	99,14	1,148	Control vs CA0	2492000	1,056	No	ns	-9909000 to 14890000
3300	E64	3,52E+08	3,52E+08	3,51E+08	3,5E+08	317058	77664837	83042	96,0782	94,5896	0,109	Control vs E64	1,6E+07	6,643	Yes	ns	3275000 to 28070000
3300	EDTA	3,55E+08	3,57E+08	3,56E+08	3,6E+08	1E+06	77664837	84380	326,966	96,1131	0,372	Control vs EDT	1,1E+07	4,773	No	ns	-1139000 to 23660000
3300	PMSF	3,57E+08	3,58E+08	3,58E+08	3,6E+08	882139	77664837	84877	267,315	96,6792	0,304	Control vs PM	9621000	4,078	No	ns	-2779000 to 22020000
3300	Bestat	1,19E+08	1,1E+08	1,1E+08	1,1E+08	5E+06	77664837	10792	1579,33	12,2932	1,799	Control vs Bes	2,5E+08	107,7	Yes	***	241700000 to 266500000

**Carboxypeptidases**												**One-way ANOVA test: P<0.0001**					
**Time (s)**	**Inhibitor**	**RFU**						**RFU/S**		**Relative activity**		**Dunnett's Multiple**	**Mean Diff.**	**q**	**Significant? P < 0.05?**		**95% CI of diff**
		**Exp1**	**Exp2**	**Exp3**	**Exp (x̄)**	**SD**	**Blank (x̄)**	**Exp (x̄)**	**SD**	**Exp (x̄)**	**SD**						
3300	Control	33869192	33476524	33369122	3,4E+07	263247	1046344	9856,1	79,7718	100	0,809						
3300	CA074	33397216	33390536	33383746	3,3E+07	6735,1	1081910	9790,5	2,04093	99,3338	0,021	**ontrol vs CA-07**	181100	1,097	No	ns	-301500 to 663800
3300	E64	33532194	33199974	33671906	3,3E+07	242422	1063854	9819,4	73,4611	99,6277	0,745	Control vs E-6	103600	0,6275	No	ns	-379100 to 586200
3300	EDTA	33485706	33541168	33035270	3,3E+07	277459	1052279	9788,4	84,0785	99,3128	0,853	Control vs EDT	217600	1,318	No	ns	-265100 to 700200
3300	PMSF	3482130	3426444	3414538	3441037	36082	1036046	728,79	10,9339	7,39422	0,111	Control vs PM	3E+07	182,5	Yes	***	29650000 to 30610000
3300	PEPSTAT	33488776	33143796	33583822	3,3E+07	231541	1044464	9806,4	70,164	99,495	0,712	Control vs Pep	166100	1,007	No	ns	-316500 to 648800

3300	RKLLW-NH2	33430904	33648730	33237690	3,3E+07	205643	1077630	9806,5	62,316	99,4964	0,632	Control vs RKL	132500	0,8027	No	ns	-350100 to 615100
3300	Bestatin	33604600	33664712	33367620	3,4E+07	157076	1029916	9853,3	47,5987	99,9707	0,483	Control vs Bes	25970	0,1573	No	ns	-456700 to 508600
**Asparagine endopeptidases**												**One-way ANOVA test: P<0.0001**					
**Time (s)**	**Inhibitor**	**RFU**						**RFU/S**		**Relative activity**		**Dunnett's Multiple**	**Mean Diff.**	**q**	**Significant? P < 0.05?**		**95% CI of diff**
		**Exp1**	**Exp2**	**Exp3**	**Exp (x̄)**	**SD**	**Blank (x̄)**	**Exp (x̄)**	**SD**	**Exp (x̄)**	**SD**						
3300	Control	1,33E+09	1,45E+09	1,48E+09	1,4E+09	8E+07	61315321	411521	23618,4	100	5,739						
3300	E64	1,39E+09	1,4E+09	1,45E+09	1,4E+09	3E+07	61315321	410414	9699,96	99,731	2,357	Control vs E64	3653000	0,04844	No	ns	-280100000 to 287400000
3300	EDTA	1,28E+09	1,53E+09	1,49E+09	1,4E+09	1E+08	61315321	416169	41816,7	101,13	10,16	Control vs ED	-2E+07	0,2035	No	ns	-299000000 to 268400000
3300	PEPSTAT	1,36E+09	1,57E+09	1,51E+09	1,5E+09	1E+08	61315321	429360	32889,6	104,335	7,992	Control vs PE	-6E+07	0,7808	No	ns	-342600000 to 224800000
3300	PMSF	1,39E+09	1,55E+09	1,55E+09	1,5E+09	9E+07	61315321	434121	28553,4	105,492	6,938	Control vs PM	-7E+07	0,9891	No	ns	-358300000 to 209100000
3300	bestatin	1,27E+09	1,41E+09	1,5E+09	1,4E+09	1E+08	61315321	403594	34218,7	98,0737	8,315	Control vs bes	2,6E+07	0,3470	No	ns	-257500000 to 309900000
3300	Legum inh	1,53E+08	1,72E+08	1,75E+08	1,7E+08	1E+07	61315321	31864	3578,52	7,74286	0,87	Control vs Leg	1,3E+09	16,62	Yes	***	969200000 to 1537000000
