# Supplementary material for: Unveiling the Peptidase Network Orchestrating Hemoglobin Catabolism in Rhodnius prolixus
Source: Mol Cell Proteomics. 2024 Apr 23;23(6):100775. doi: 10.1016/j.mcpro.2024.100775 (PMC11135036; doi:10.1016/j.mcpro.2024.100775)
Supplement: Supplemental Table S3 [file mmc3.pdf]

Table S3: Temporal activity patterns of various peptidase families in the tissue extracts of AM and PM before and at various post-feeding time points

		Time (s)	Post-feeding time (h)	Blank (x̄)	Tissue extracts							Digestive contents						
					RFU					RFU/S/µg		RFU					RFU/S/µl	
					Exp1	Exp2	Exp3	Exp (x̄)	SD	Exp (x̄)	SD	Exp1	Exp2	Exp3	Exp (x̄)	SD	Exp (x̄)	SD
A1 aspartic peptidases	Anterior midgut	3000	0	3494202	4722261	4735171	4723981	4727137,77	7009,919426	410,97859	2,3366398	7689738	7806280	7800414	7765477,3	65657,73	284,75169	4,377182
		3000	6	3494202	12211629	12360866	12575635	12382710	182983,5027	2962,836	60,994501	20177292	21906726	21189274	21091097	868886,9	1173,1264	57,925795
		3000	24	3494202	11171711	11155523	11292410	11206547,9	74797,70383	2570,782	24,932568	39317682	38351881	38139565	38603043	627934,4	2340,5894	41,86229
		3000	48	3494202	11844780	11805026	11850930	11833578,7	24917,79897	2779,7922	8,305933	39781662	39450210	39345910	39525927	227529,8	2402,115	15,16865
		3000	168	3494202	9771240	9493590	9718345	9661058,33	147423,5472	2055,6188	49,141182	30818889	30362296	30425651	30535612	247361,8	1802,7607	16,490786
		3000	366	3494202	9136284	9231876	9501852	9290004	189589,3957	1931,934	63,196465	33781662	33450210	32345910	33192594	751744,9	1979,8928	50,116329
	Posterior midgut	3000	0	3494202	8265267	7852925	8366842	8828310	13956,42662	1778,036	4,6521422	6167460	6239386	6185106	6197317,3	37485,66	180,20769	2,4990441
		3000	6	3494202	7221984	7576590	7713069	7503881	253487,807	1336,5597	84,495936	9686721	9555102	9542195	9594672,7	79976,99	406,69804	5,3317995
		3000	24	3494202	8840628	8813152	8831150	8161678	272168,4798	1555,8253	90,722827	24722202	24250240	24893676	24622039	333207	1408,5225	22,213799
		3000	48	3494202	9715563	10604920	9653258	9991247	532368,6612	2165,6817	177,45622	48986502	49323020	49006287	49105270	188836,7	3040,7378	12,589114
		3000	168	3494202	6621039	6821805	6783990	6742278	106684,886	1082,692	35,561629	48944972	49142340	48828067	48971793	158844,1	3031,8394	10,589607
		3000	366	3494202	7023012	7576590	7852925	7484175,67	422604,0743	1329,9912	140,86802	46707112	46623442	47019760	46783438	208892,7	2885,9491	13,926179
C1 cysteine peptidases	Anterior midgut	3600	0	50715875	49735848	86131224	90194888	75353986,7	22278804,12	6843,92	6188,5567	64802440	67592728	63907360	65434176	1922187	2985,0606	106,78816
		3600	6	50715875	49735848	87191032	73548008	70158296	18956273,7	5400,6726	5265,6316	3359221	3490609	4007548	3619126	342738,1	-1307,651	95,205042
		3600	24	50715875	49735848	78072176	78831456	68879826,7	16583517,9	5045,5422	4606,5327	10907317	8048709	7023147	8659724,3	2012883	-957,6097	559,13423
		3600	48	50715875	49735848	123693976	122086296	98505373,3	42243296,62	13274,861	11734,249	23705476	20845574	19544530	21365193	2128584	-75,28546	591,27341
		3600	168	50715875	49735848	111451864	106601208	89262973,3	34317305,31	10707,527	9532,5848	1,33E+08	1,27E+08	1,28E+08	129040437	3196312	7402,162	887,86439
		3600	366	50715875	49735848	110063640	101386648	87062045,3	32615277,14	10096,159	9059,7992	1,39E+08	1,38E+08	1,38E+08	138216283	414485,6	8039,3735	115,13488
	Posterior midgut	3600	0	50715875	2,2E+08	211536112	203835072	211946773	8324632,324	44786,361	2312,3979	3,35E+08	3,43E+08	2,59E+08	312159285	46243676	14524,634	2569,0931
		3600	6	50715875	2,86E+08	532804448	488601824	435909824	131417603,7	106998,32	36504,89	1,47E+09	1,55E+09	1,13E+09	1,382E+09	2,19E+08	73954,998	12160,206
		3600	24	50715875	3,1E+08	686810816	597626240	531553312	196796448,8	133565,95	54665,68	1,52E+09	1,53E+09	1,27E+09	1,444E+09	1,47E+08	77413,51	8146,8456
		3600	48	50715875	6,9E+08	845528896	764278272	766700928	77644991,8	198884,74	21568,053	1,51E+09	1,33E+09	1,19E+09	1,343E+09	1,6E+08	71771,993	8909,5293
		3600	168	50715875	4,94E+08	500008000	446863680	480407445	29187146,65	119358,77	8107,5407	1,44E+09	1,42E+09	1,16E+09	1,336E+09	1,56E+08	71421,131	8662,3061
		3600	366	50715875	2,33E+08	261701888	237915904	244045355	15527346,35	53702,633	4313,1518	1,45E+09	1,43E+09	1,13E+09	1,34E+09	1,79E+08	71619,861	9940,2288
Aminopeptidases	Anterior midgut	3600	0	98126208	1,11E+09	1,316E+09	1,382E+09	1268639659	142825125,2	325142,63	39673,646	1,47E+08	1,43E+08	1,44E+08	144506389	2196529	2576,6767	122,0294
		3600	6	98126208	2,75E+09	2,844E+09	2,924E+09	2839366315	86884368,2	761455,59	24134,547	3,23E+08	3,17E+08	3,08E+08	316036992	7862626	12106,155	436,81257
		3600	24	98126208	2,95E+09	2,987E+09	2,897E+09	2945694464	45336214,43	790991,18	12593,393	5,5E+08	6,18E+08	5,58E+08	575163584	37228798	26502,076	2068,2666
		3600	48	98126208	3,15E+09	3,171E+09	3,126E+09	3149293739	22888762,05	847546,54	6357,9895	4,86E+08	6,25E+08	6,81E+08	597346880	1E+08	27734,482	5581,3729
		3600	168	98126208	3,12E+09	3,182E+09	3,12E+09	3141447680	35544007,04	845367,08	9873,3353	7,23E+08	7,48E+08	6,96E+08	722219093	26316356	34671,827	1462,0198
		3600	366	98126208	2,89E+09	2,88E+09	2,795E+09	2855866027	53056963,66	766038,84	14738,045	1,94E+09	1,88E+09	1,93E+09	1,915E+09	32029252	100942,7	1779,4029
	Posterior midgut	3600	0	98126208	2,34E+09	2,323E+09	2,364E+09	2344049749	20294331,58	623867,65	5637,3143	1,25E+08	1,21E+08	1,2E+08	122060413	2905681	1329,6781	161,4267
		3600	6	98126208	2,99E+09	3E+09	2,985E+09	2992384683	7503721,367	803960,69	2084,367	8,76E+08	9,02E+08	9,67E+08	915186283	46936856	45392,226	2607,6031
		3600	24	98126208	2,59E+09	2,614E+09	2,525E+09	2574858837	45321218,38	687981,29	12589,227	8,91E+08	9,05E+08	9E+08	898712757	7371179	44477,031	409,50997
		3600	48	98126208	3E+09	2,977E+09	2,866E+09	2948129536	72538147,51	791667,59	20149,485	9,11E+08	9,09E+08	9,08E+08	909198933	1373962	45059,596	76,331249
		3600	168	98126208	2,22E+09	2,684E+09	2,64E+09	2513526443	257507721,3	670944,51	71529,923	9,16E+08	9,42E+08	8,69E+08	909020267	37167773	45049,67	2064,8763
		3600	366	98126208	1,42E+09	1,8E+09	1,928E+09	1716185515	263994099,5	449460,92	73331,694	3,74E+08	3,75E+08	3,86E+08	378366709	6656632	15568,917	369,81292
es	midgut	2100	0	1454049,7	6648918	11110763	9917232	9225637,67	2309922,562	3700,7562	1099,9631	28889082	29607382	29714334	29403599	448782,5	2661,8619	42,741192
		2100	6	1454049,7	3553948	4759410	3056508	3789955,33	875638,8533	1112,336	416,97088	29529060	30853044	28494558	29625554	1182200	2683,0004	112,5905
		2100	24	1454049,7	12762633	9773433	10045608	10860558	1652857,159	4479,2897	787,07484	27868148	29291290	27216166	28125201	1061175	2540,1097	101,06428

Carboxypeptidas	Anterior	2100	48	1454049,7	12482503	14020110	15327475	13943362,7	1424037,933	5947,2919	678,1133	34503980	34692316	31859422	33685239	1584006	3069,6371	150,85769
		2100	168	1454049,7	13522841	14278451	13981669	13927653,7	380689,9791	5939,8114	181,28094	35868376	33507430	31941092	33772299	1976994	3077,9285	188,28518
		2100	366	1454049,7	9185477	9382232	14158624	10908777,7	2816168,323	4502,2514	1341,0325	49827972	47766628	51845612	49813404	2039531	4605,6528	194,24105
	Posterior midgut	2100	0	1454049,7	7616786	8692998	7675462	7995082	605124,5951	3114,7773	288,15457	3737716	4208134	3596462	3847437,3	320257,3	227,94168	30,500695
		2100	6	1454049,7	6002620	6434428	5380950	5939332,67	529582,7972	2135,849	252,18228	31932320	31317934	38074440	33774898	3736163	3078,176	355,82504
		2100	24	1454049,7	10095049	11649474	10354920	10699814,3	832630,3778	4402,7451	396,49066	40244200	41922044	41487088	41217777	870738,9	3787,0217	82,927518
		2100	48	1454049,7	9397487	13734156	10315698	11149113,7	2285303,209	4616,6971	1088,2396	36195836	38709996	36111632	37005821	1476459	3385,883	140,61514
		2100	168	1454049,7	5886104	6489562	6733003	6369556,33	436016,6271	2340,7175	207,62697	25321568	26620270	26525856	26155898	724091,4	2352,557	68,961089
		2100	366	1454049,7	4976294	5824807	5579624	5460241,67	436672,2844	1907,7105	207,93918	19078720	18743890	20386136	19402915	867796	1709,4158	82,647241
Asparagine endopeptidases	Anterior midgut	2400	0	287230144	2,92E+08	292873664	296228832	293570837	2387028,132	2641,9556	994,59506	2,89E+08	2,88E+08	2,88E+08	288328875	180937,1	91,56091	15,078093
		2400	6	287230144	3,06E+08	305577920	306556768	306033931	492833,8506	7834,9111	205,34744	2,89E+08	2,89E+08	2,89E+08	288985945	108178,4	146,31674	9,0148638
		2400	24	287230144	3,15E+08	319458400	318913216	317810037	2398445,576	12741,622	999,35232	2,93E+08	2,94E+08	2,93E+08	293261036	312955,4	502,57437	26,079615
		2400	48	287230144	3,17E+08	319734784	320038240	318891979	1729251,584	13192,431	720,52149	2,94E+08	2,94E+08	2,94E+08	294021098	140102	565,91281	11,675166
		2400	168	287230144	3,15E+08	320422400	320160256	318650976	2844194,775	13092,013	1185,0812	2,94E+08	2,95E+08	2,94E+08	294233783	288941,6	583,63656	24,078463
		2400	366	287230144	3,11E+08	315986304	314842144	313956501	2588849,483	11135,982	1078,6873	2,89E+08	2,89E+08	2,89E+08	289188109	372715,4	163,16372	31,059617
	Posterior midgut	2400	0	287230144	2,89E+08	286225024	295823488	290379744	4927353,747	1312,3333	2053,0641	3,03E+08	3,07E+08	3,04E+08	304483435	1779403	1437,7742	148,28362
		2400	6	287230144	2,98E+08	299079744	308672160	301803115	5991124,687	6072,0711	2496,302	3,15E+08	3,14E+08	3,1E+08	313087083	2710308	2154,7449	225,85902
		2400	24	287230144	3,08E+08	308897248	312350912	309608363	2465155,95	9324,2578	1027,1483	3,16E+08	3,14E+08	3,15E+08	314806283	849907,4	2298,0116	70,825613
		2400	48	287230144	3,13E+08	307036160	#SAT	309924320	4084475,042	9455,9067	1701,8646	3,22E+08	3,24E+08	3,25E+08	323825856	1298302	3049,6427	108,19179
		2400	168	287230144	3,06E+08	307451648	314814560	309470923	4673506,032	9266,9911	1947,2942	3,22E+08	3,19E+08	3,19E+08	319930411	1428443	2725,0222	119,03692
		2400	366	287230144	3,08E+08	306476704	311751232	308613173	2776246,981	8909,5956	1156,7696	3,21E+08	3,21E+08	3,23E+08	321684768	1249101	2871,2187	104,09171
