# Supplementary material for: Unveiling the Peptidase Network Orchestrating Hemoglobin Catabolism in Rhodnius prolixus
Source: Mol Cell Proteomics. 2024 Apr 23;23(6):100775. doi: 10.1016/j.mcpro.2024.100775 (PMC11135036; doi:10.1016/j.mcpro.2024.100775)
Supplement: Supplemental Table S4 [file mmc4.pdf]

Table S4: Effect of pH on Hemoglobin digestion

Time (s)	pH	RFU						RFU/S		Relative activity	
		Exp1	Exp2	Exp3	Exp ($\bar{X}$)	SD	Blank ($\bar{X}$)	Exp ($\bar{X}$)	SD	Exp ($\bar{X}$)	SD
3000	**2**	2069098	1983904	1979441	2010814,33	50524,4391	1267982,33	247,610667	16,8414797	2,94043419	0,19999648
3000	**3**	4705090	3883324	4256536	4281650	411458,229	1666476	871,724667	137,152743	10,3519329	1,62872069
3000	**4**	15098926	15491265	17953676	16181289	1547416,95	2806103,33	4458,39522	515,805651	52,944479	6,12531194
3000	**5**	34149460	27813312	24674294	28879022	4826644,39	3616358,33	8420,88789	1608,88146	100	19,1058411
3000	**6**	13391760	12573781	12374360	12779967	539129,103	6049491,33	2243,49189	179,709701	26,6419874	2,13409445
3000	**7**	6307307	5753416	5236635	5765786	535443,177	2725526,67	1013,41978	178,481059	12,0345953	2,11950404
3000	**8**	5030592	4879910	4105106	4671869,33	496580,077	3853269	272,866778	165,526692	3,24035638	1,96566793
