# Supplementary material for: Unveiling the Peptidase Network Orchestrating Hemoglobin Catabolism in Rhodnius prolixus
Source: Mol Cell Proteomics. 2024 Apr 23;23(6):100775. doi: 10.1016/j.mcpro.2024.100775 (PMC11135036; doi:10.1016/j.mcpro.2024.100775)
Supplement: Supplemental Table S5 [file mmc5.pdf]

Table S5: Hemoglobin digestion in presence of protease inhibitors

Time (s)	Inhibitor	RFU						RFU/S		Relative activity		One way ANOVA: P<0.0001					
		Exp1	Exp2	Exp3	Exp ($\bar{x}$)	SD	Blank ($\bar{x}$)	Exp ($\bar{x}$)	SD	Exp ($\bar{x}$)	SD	Dunnett's Multiple	Mean Diff.	q	Significant? P < 0.05?	Summary	95% CI of diff
2340	Control	17621010	18015140	18793094	18143081,3	596424,3	5155074	5550,43	254,882	100	4,59212						
2340	Pepsatin A	11891787	12858251	12094504	12281514	509649,6	5155074	3045,49	217,799	54,8694	3,924	Control vs Pepsat	5862000	7,967	Yes	***	3710000 to 8013000
2340	CA074	14781358	15611842	15279233	15224144,3	417973,7	5155074	4303,02	178,621	77,5259	3,21815	Control vs CA074	2919000	3,967	Yes	**	767600 to 5070000
2340	E64	13566941	13478259	14915524	13986908	805426,5	5155074	3774,29	344,199	67,9999	6,20131	Control vs E64	4156000	5,649	Yes	***	2005000 to 6307000
2340	EDTA	17271214	17931876	18248312	17817134	498552,3	5155074	5411,14	213,057	97,4904	3,83856	Control vs EDTA	325900	0,4430	No	ns	-1825000 to 2477000
2340	PMSF	13710344	16929700	16431641	15690561,7	1732906	5155074	4502,35	740,558	81,117	13,3424	Control vs PMSF	2453000	3,333	Yes	*	301200 to 4604000
2340	Legumain I1	15934111	15719987	17315796	16323298	866170,6	5155074	4772,75	370,158	85,9887	6,669	Control vs Legum	1820000	2,473	No	ns	-331500 to 3971000
2340	Bestat	16305034	14603014	16451154	15786400,7	1027444	5155074	4543,3	439,079	81,8549	7,91071	Control vs Bestat	2357000	3,203	Yes	*	205400 to 4508000
