# Supplementary material for: Unveiling the Peptidase Network Orchestrating Hemoglobin Catabolism in Rhodnius prolixus
Source: Mol Cell Proteomics. 2024 Apr 23;23(6):100775. doi: 10.1016/j.mcpro.2024.100775 (PMC11135036; doi:10.1016/j.mcpro.2024.100775)
Supplement: Supplemental Table S6 [file mmc6.pdf]

Table S6: Hemoglobin digestion by AM and PM tissue extracts and contents before and at various post-feeding times

Time (s)	Post-feeding time (h)	Blank (X̄) Tissue	Blank (X̄) Content AM	Blank (X̄) Content PM	Anterior midgut							Posterior midgut						
					Tissue extracts													
					RFU					RFU/S/µg		RFU					RFU/S/µg	
					Exp1	Exp2	Exp3	Exp (X̄)	SD	Exp (X̄)	SD	Exp1	Exp2	Exp3	Exp (X̄)	SD	Exp (X̄)	SD
3300	0	2715125,33			4001622	4448956	4115812	4188796,67	232426,328	446,567071	70,4322207	7182872	7287939	7094583	7188464,667	96799,2465	1355,55737	29,333105
3300	6	2715125,33			8284088	9647211	9476156	9135818,33	742562,034	1945,66455	225,018798	10035476	9016272	8340763	9130837	853145,294	1944,15505	258,528877
3300	24	2715125,33			8713614	10304930	9565864	9528136	796328,578	2064,54869	241,31169	7031606	9444964	8844644	8440404,667	1256435,95	1734,93313	380,738166
3300	48	2715125,33			13184289	13020640	13323180	13176036,3	151438,743	3169,97303	45,8905281	9765979	10076016	10700278	10180757,67	475874,74	2262,31283	144,204467
3300	168	2715125,33			9478583	10462428	10801699	10247570	687227,733	2282,55899	208,250828	6330878	6487430	6583682	6467330	127594,957	1137,03172	38,6651385
3300	366	2715125,33			8656020	10209092	9591757	9485623	781956,827	2051,66596	236,956614	7049596	7082871	6716624	6949697	202531,661	1283,20354	61,3732308
					Digestive Contents													
					RFU					RFU/S/µl		RFU					RFU/S/µl	
					Exp1	Exp2	Exp3	Exp (X̄)	SD	Exp (X̄)	SD	Exp1	Exp2	Exp3	Exp (X̄)	SD	Exp (X̄)	SD
3300	0		22993284	2524088	32936592	34202936	37288772	34809433,3	2238581,46	716,130263	135,671604	8286739	7724484	7238084	7749769	524784,552	316,707939	31,8051243
3300	6		22679212,7	18975411,3	38123596	37367556	36843696	37444949,3	643450,308	894,893131	38,9969883	35448480	35985816	35079956	35504750,67	455544,044	1001,77814	27,6087299
3300	24		26522193,3	25492041,3	41766112	43422696	39207816	41465541,3	2123454,83	905,657455	128,694232	50134620	43152892	43822384	45703298,67	3852208,65	1224,92469	233,467191
3300	48		37229434,7	20655770	59534564	56282424	56100688	57305892	1932224,41	1216,75499	117,104509	38212884	39633372	39333996	39060084	748810,642	1115,41297	45,3824631
3300	168		35871429,3	13590268,7	49606100	46738384	46623688	47656057,3	1689759,92	714,219879	102,409692	32675788	31411116	29441826	31176243,33	1629724,34	1065,81665	98,7711723
3300	366		64670500	11339815,3	79782576	79095192	76834104	78570624	1542643,99	842,431758	93,493575	19409414	23060178	25734904	22734832	3175270,61	690,607071	192,440643
