# Supplementary material for: Unveiling the Peptidase Network Orchestrating Hemoglobin Catabolism in Rhodnius prolixus
Source: Mol Cell Proteomics. 2024 Apr 23;23(6):100775. doi: 10.1016/j.mcpro.2024.100775 (PMC11135036; doi:10.1016/j.mcpro.2024.100775)
Supplement: Supplemental Table S7 [file mmc7.pdf]

Table S7: Mass spectrometry identified peptides fellowing specific digestion of Human hemoglobin

A1 aspartic proteases

Alpha-subunit

Sequence	Prob	P1-P1'	Mascot Ion score	Mascot Identity score	Mascot Delta Ion Score	NTT	Modifications	Observed	Actual Mass	Charge	Delta Da	Delta PPM	Retention Time	TIC
(M)VLSPADKTNVKAAWGKVGAHAGEYGAEALERm(F)	100 %	MF	160.05	50.83531	156.83	2	Oxidation (+16)	836,4327	3 341,7019	4	0,01897	5,675	1 641,60	176342
(M)VLSPADKTNVKAAWGKVGAHAGEYGAEALERm(F)	100 %	MF	139.9	50.87565	127.76	2	Oxidation (+16)	836,4286	3 341,6855	4	0,002571	0,7692	1 647,45	114972
(M)VLSPADKTNVKAAWGKVGAHAGEYGAEALERm(F)	100 %	MF	109.94	50.86086	99.21	2	Oxidation (+16)	836,4313	3 341,6961	4	0,01325	3,964	1 640,52	84 062,0
(M)VLSPADKTNVKAAWGKVGAHAGEYGAEALERm(F)	99 %	MF	65.87	50.87263	58.78	2	Oxidation (+16)	669,3423	3 341,6751	5	-0,007815	-2,338	1 641,60	192203
(L)SPADKTNVKAAWGKVGAHAGEY(G)	100 %	YG	81.35	49.705093	77.67	2		753,0509	2 256,1309	3	0,002032	0,9003	1 438,14	38 405,0
(L)SPADKTNVKAAWGKVGAHAGEY(G)	97 %	YG	55.86	49.625916	52.34	2		753,0519	2 256,1338	3	0,004942	2,19	1 440,05	31 210,0
(L)SPADKTNVKAAWGKVGAHAGEYGAEA(L)	100 %	AL	162.83	50.02088	154.59	2		862,4331	2 584,2773	3	0,01015	3,927	1 291,93	387567
(L)SPADKTNVKAAWGKVGAHAGEYGAEA(L)	100 %	AL	86.65	49.880997	80.47	2		862,4293	2 584,2660	3	-0,001128	-0,4363	1 289,68	117529
(L)SPADKTNVKAAWGKVGAHAGEYGAEA(L)	100 %	AL	85.55	49.967697	80.1	2		647,0743	2 584,2683	4	0,001086	0,4201	1 290,85	131988
(L)SPADKTNVKAAWGKVGAHAGEYGAEA(L)	100 %	AL	84.05	50.017033	73.82	2		647,0754	2 584,2725	4	0,005286	2,045	1 291,93	81 939,0
(L)SPADKTNVKAAWGKVGAHAGEYGAEAL(E)	100 %	LE	138.69	50.29388	132.85	2		900,1265	2 697,3577	3	0,006432	2,384	1 564,66	164398
(L)SPADKTNVKAAWGKVGAHAGEYGAEAL(E)	100 %	LE	134.37	50.343075	113.79	2		900,1254	2 697,3544	3	0,003102	1,15	1 565,83	363565
(L)SPADKTNVKAAWGKVGAHAGEYGAEAL(E)	100 %	LE	121.49	50.307457	108.98	2		675,3453	2 697,3519	4	0,0006661	0,2468	1 564,66	222911
(L)SPADKTNVKAAWGKVGAHAGEYGAEAL(E)	100 %	LE	80.58	50.297775	59.82	2		900,1239	2 697,3497	3	-0,001548	-0,5737	1 565,08	170821
(D)KTNVKAAWGKVGAHAGEYGAEAL(E)	100 %	LE	96.17	49.533485	83.55	2		776,742	2 327,2042	3	0,001862	0,7998	1 345,60	268292
(D)KTNVKAAWGKVGAHAGEYGAEAL(E)	100 %	LE	89.76	49.529423	78.55	2		776,7416	2 327,2030	3	0,0006021	0,2586	1 344,60	215076
(W)GKVGAHAGEYGAEAL(E)	100 %	LE	74.62	47.971706	54.26	2		715,3552	1 428,6958	2	-0,004082	-2,855	1 584,72	47 049,0
(W)GKVGAHAGEYGAEAL(E)	100 %	LE	72.01	48.199318	53.02	2		715,3582	1 428,7018	2	0,001918	1,342	979,97	638557
(E)YGAEALERMF(L)	100 %	FL	69.82	46.04194	47.07	2		593,7795	1 185,5444	2	-0,004482	-3,777	1 996,25	50 936,0
(E)YGAEALERMF(L)	98 %	FL	57.21	46.25498	31.38	2		593,7784	1 185,5423	2	-0,006542	-5,513	1 998,09	28 966,0
(Y)GAEALERMF(L)	99 %	FL	58.36	47.14623	31.53	2		512,249	1 022,4835	2	-0,002062	-2,015	1 875,54	17 992,0
(Y)GAEALERMF(L)	95 %	FL	49.82	47.148407	17.99	2		512,2492	1 022,4839	2	-0,001642	-1,604	1 876,79	184634
(F)LSFPTTKTYFPHFDLSHG(S)	100 %	GS	113.93	49.3047	100.81	2		699,013	2 094,0172	3	-0,004068	-1,942	1 996,92	61 647,0
(F)LSFPTTKTYFPHFDLSHG(S)	100 %	GS	73.21	49.298325	62.33	2		699,0128	2 094,0167	3	-0,004578	-2,185	1 997,17	29 626,0
(F)LSFPTTKTYFPHFDLSHG(S)	97 %	GS	55.05	49.23845	41.52	2		699,0119	2 094,0140	3	-0,007308	-3,488	1 997,17	40 246,0
(F)PTTKTYFPHFDLSHG(S)	99 %	GS	62.45	48.423843	53.07	2		583,2853	1 746,8340	3	-0,002788	-1,595	1 737,61	61 954,0
(F)PTTKTYFPHFDLSHG(S)	98 %	GS	65.0	54.15641	58.44	2		874,4261	1 746,8377	2	0,0009381	0,5367	1 736,77	119223
(F)PTTKTYFPHFDLSHG(S)	98 %	GS	57.43	48.61325	42.59	2		583,286	1 746,8362	3	-0,0005379	-0,3078	1 735,36	28 488,0
(T)TKTYFPHF(D)	97 %	FD	53.81	46.746223	23.72	2		520,7629	1 039,5112	2	-0,001562	-1,501	1 692,16	33 429,0
(T)TKTYFPHF(D)	93 %	FD	47.39	46.773514	16.2	2		520,7621	1 039,5095	2	-0,003222	-3,096	2 021,95	25 725,0
(F)DLSHGSAQVKGHGKKVADALTNA(V)	100 %	AV	120.35	49.24925	111.47	2		768,7433	2 303,2079	3	0,009452	4,102	939,07	621223
(F)DLSHGSAQVKGHGKKVADALTNA(V)	100 %	AV	116.51	49.524826	105.08	2		768,7389	2 303,1948	3	-0,003718	-1,614	929,835	377700
(F)DLSHGSAQVKGHGKKVADALTNA(V)	100 %	AV	107.56	49.50511	98.48	2		768,7395	2 303,1966	3	-0,001888	-0,8193	957,714	385859
(F)DLSHGSAQVKGHGKKVADALTNA(V)	100 %	AV	107.09	49.379692	104.8	2		768,7418	2 303,2036	3	0,005102	2,214	954,21	412543
(F)DLSHGSAQVKGHGKKVADALTNA(V)	100 %	AV	102.79	49.500484	88.99	2		768,7395	2 303,1966	3	-0,001918	-0,8324	954,293	349938
(F)DLSHGSAQVKGHGKKVADALTNA(V)	100 %	AV	96.46	49.53194	94.22	2		768,7395	2 303,1965	3	-0,001948	-0,8454	928,919	200219
(F)DLSHGSAQVKGHGKKVADALTNA(V)	100 %	AV	91.56	49.48237	78.8	2		768,7393	2 303,1960	3	-0,002488	-1,08	961,217	212677
(F)DLSHGSAQVKGHGKKVADALTNA(V)	100 %	AV	78.12	49.124344	68.77	2		768,7448	2 303,2125	3	0,01404	6,094	928,919	270337
(F)DLSHGSAQVKGHGKKVADALTNA(V)	100 %	AV	72.08	49.589886	69.21	2		768,7376	2 303,1908	3	-0,007648	-3,319	929,753	162502
(F)DLSHGSAQVKGHGKKVADALTNA(V)	100 %	AV	71.76	49.48095	57.72	2		768,7393	2 303,1960	3	-0,002518	-1,093	929,835	287711

(F)DLSHGSAQVKGHGKKVADALTNA(V)	99 %	AV	68.13	51.549957	66.61	2		576,8059	2 303,1943	4	-0,004174	-1,811	929,835	513511
(F)DLSHGSAQVKGHGKKVADALTNA(V)	99 %	AV	63.76	49.56005	61.7	2		768,7389	2 303,1948	3	-0,003688	-1,601	954,293	283826
(F)DLSHGSAQVKGHGKKVADALTNA(V)	96 %	AV	53.47	49.408	45.61	2		576,8073	2 303,2000	4	0,001546	0,671	954,21	320710
(F)DLSHGSAQVKGHGKKVADALTNA(V)	95 %	AV	52.58	49.47414	49.85	2		576,8068	2 303,1979	4	-0,0005339	-0,2317	960,047	212399
(H)GKKVADALTNA(V)	91 %	AV	46.55	47.327713	24.17	2		544,3076	1 086,6006	2	-0,002882	-2,65	727,213	18 535,0
(H)GKKVADALTNA(V)	91 %	AV	46.05	47.358467	31.97	2		544,308	1 086,6013	2	-0,002122	-1,951	729,377	35 321,0
(D)ALTNAVAHVDDmPNALSAL(S)	100 %	LS	71.97	49.14856	52.98	2	Oxidation (+16)	969,9832	1 937,9519	2	0,000103	0,05315	1 957,15	53 635,0
(D)ALTNAVAHVDDMPNALSAL(S)	98 %	LS	59.39	49.32702	46.9	2		961,9862	1 921,9579	2	0,001038	0,5398	2 116,18	81 816,0
(D)ALTNAVAHVDDmPNALSAL(S)	97 %	LS	56.73	49.168854	47.92	2	Oxidation (+16)	969,9852	1 937,9559	2	0,004143	2,137	1 956,15	36 100,0
(D)ALTNAVAHVDDmPNALSALSD(L)	100 %	DL	74.96	48.537407	63.49	2	Oxidation (+16)	1 071,0117	2 140,0088	2	-0,001997	-0,9327	1 912,00	24 106,0
(D)ALTNAVAHVDDMPNALSALSD(L)	99 %	DL	64.9	48.89621	54.98	2		1 063,0170	2 124,0194	2	0,003538	1,665	2 077,54	43 309,0
(A)VAHVDDMPNALSALSD(L)	96 %	DL	52.22	47.399754	36.38	2		827,8895	1 653,7644	2	-0,002522	-1,524	1 935,15	42 426,0
(A)VAHVDDMPNALSALSD(L)	93 %	DL	48.56	47.40773	33.96	2		827,8886	1 653,7626	2	-0,004382	-2,648	1 933,98	44 629,0
(A)VAHVDDmPNALSALSDLH(A)	100 %	HA	110.98	48.307877	95.57	2	Oxidation (+16)	960,9584	1 919,9021	2	-0,002737	-1,425	1 858,03	52 888,0
(A)VAHVDDmPNALSALSDLH(A)	100 %	HA	100.51	48.220245	79.19	2	Oxidation (+16)	960,9568	1 919,8989	2	-0,005937	-3,091	1 857,11	46 516,0
(A)VAHVDDMPNALSALSDLH(A)	99 %	HA	64.63	48.65637	52.18	2		952,9618	1 903,9091	2	-0,0009019	-0,4735	2 029,79	34 660,0
(A)VAHVDDMPNALSALSDLH(A)	98 %	HA	61.62	52.413696	44.44	2		952,9623	1 903,9101	2	0,0001581	0,08297	2 029,88	48 901,0
(A)VAHVDDmPNALSALSDLHAHKL(R)	100 %	LR	97.53	49.760242	89.36	2	Oxidation (+16)	790,7316	2 369,1729	3	-0,007043	-2,971	1 774,10	69 396,0
(A)VAHVDDmPNALSALSDLHAHKL(R)	100 %	LR	91.98	49.77147	88.36	2	Oxidation (+16)	790,7327	2 369,1764	3	-0,003593	-1,516	1 774,10	53 512,0
(A)VAHVDDMPNALSALSDLHAHKL(R)	100 %	LR	76.41	54.518066	74.75	2		785,4036	2 353,1890	3	0,003902	1,657	1 923,82	39 625,0
(A)VAHVDDmPNALSALSDLHAHKLRVD(P)	100 %	DP	77.83	50.310524	62.6	2	Oxidation (+16)	685,8484	2 739,3644	4	-0,01211	-4,419	1 744,64	94 640,0
(A)VAHVDDmPNALSALSDLHAHKLRVD(P)	96 %	DP	55.08	50.292336	42.98	2	Oxidation (+16)	685,8507	2 739,3737	4	-0,002749	-1,003	1 742,38	51 472,0
(A)VAHVDDmPNALSALSDLHAHKLRVD(P)	91 %	DP	49.55	50.344482	42.11	2	Oxidation (+16)	548,8817	2 739,3721	5	-0,004415	-1,611	1 743,64	26 216,0
(A)LSALSDLHAHKL(R)	100 %	LR	95.67	46.25672	76.34	2		652,8689	1 303,7232	2	-0,001722	-1,32	1 349,19	44 580,0
(A)LSALSDLHAHKL(R)	100 %	LR	84.57	46.193024	63.7	2		652,8694	1 303,7241	2	-0,0008219	-0,63	1 350,27	131506
(A)LSALSDLHAHKLRVD(P)	100 %	DP	125.61	47.085487	93.17	2		558,98	1 673,9181	3	-0,003418	-2,041	1 342,18	104965
(A)LSALSDLHAHKLRVD(P)	100 %	DP	101.69	46.785732	84.85	2		837,9705	1 673,9264	2	0,004958	2,96	1 342,18	390524
(L)SALSDLHAHKL(R)	100 %	LR	77.5	47.421513	54.44	2		596,3266	1 190,6386	2	-0,002302	-1,932	1 350,27	51 563,0
(L)SALSDLHAHKL(R)	99 %	LR	64.97	47.234062	43.61	2		596,3276	1 190,6405	2	-0,0003219	-0,2702	1 105,49	33 835,0
(L)SALSDLHAHKL(R)	98 %	LR	55.5	47.425446	37.18	2		596,3269	1 190,6392	2	-0,001682	-1,411	1 774,27	21 837,0
(L)SALSDLHAHKL(R)	96 %	LR	51.61	47.165375	30.56	2		596,3272	1 190,6399	2	-0,0009819	-0,824	1 102,09	14 175,0
(L)SALSDLHAHKL(R)	95 %	LR	49.9	47.157692	28.75	2		596,3283	1 190,6420	2	0,001098	0,9215	1 114,14	11 338,0
(L)SALSDLHAHKL(R)	93 %	LR	48.12	47.329803	38.77	2		596,3257	1 190,6368	2	-0,004062	-3,409	1 923,65	40 042,0
(L)SALSDLHAHKLRVDPVNF(K)	100 %	FK	124.68	48.71199	108.82	2		1 010,0418	2 018,0691	2	-0,0007619	-0,3774	1 722,39	657667
(L)SALSDLHAHKLRVDPVNF(K)	100 %	FK	92.03	48.86406	76.2	2		1 010,0400	2 018,0654	2	-0,004502	-2,23	1 777,44	212402
(L)SALSDLHAHKLRVDPVNF(K)	97 %	FK	55.66	48.868797	44.26	2		673,6958	2 018,0656	3	-0,004268	-2,114	1 722,39	191640
(L)SDLHAHKLRVDPVNF(K)	100 %	FK	100.82	48.496468	84.0	2		874,4686	1 746,9226	2	0,005918	3,386	1 413,47	461565
(L)SDLHAHKLRVDPVNF(K)	100 %	FK	98.09	48.415344	73.2	2		874,4692	1 746,9237	2	0,007078	4,049	1 414,30	738342
(L)SDLHAHKLRVDPVNF(K)	100 %	FK	97.19	48.860054	73.05	2		874,4652	1 746,9159	2	-0,0008019	-0,4588	1 777,52	165596
(L)SDLHAHKLRVDPVNF(K)	100 %	FK	88.75	48.52852	70.63	2		874,4675	1 746,9205	2	0,003798	2,173	1 413,22	491849
(L)SDLHAHKLRVDPVNF(K)	100 %	FK	88.98	48.89957	76.06	2		874,4651	1 746,9157	2	-0,0009419	-0,5389	1 722,47	174142
(L)SDLHAHKLRVDPVNF(K)	100 %	FK	73.27	54.35531	48.35	2		874,4642	1 746,9139	2	-0,002762	-1,58	1 596,12	108940
(L)SDLHAHKLRVDPVNF(K)	99 %	FK	60.76	48.689087	48.16	2		874,4643	1 746,9140	2	-0,002622	-1,5	1 664,15	75 859,0
(S)DLHAHKLRVDPVNF(K)	100 %	FK	71.37	48.331535	63.64	2		830,9504	1 659,8863	2	0,001598	0,9622	1 592,54	71 250,0
(S)DLHAHKLRVDPVNF(K)	100 %	FK	67.36	48.277184	56.46	2		830,9495	1 659,8843	2	-0,0003219	-0,1938	1 722,89	59 503,0
(S)DLHAHKLRVDPVNF(K)	99 %	FK	59.52	48.260685	42.93	2		830,9488	1 659,8829	2	-0,001722	-1,037	1 568,42	22 661,0

(D)LHAHKLRVD(P)	99 %	DP	64.75	47.57206	43.46	2	544,8192	1 087,6237	2	-0,001422	-1,306	793,706	55 389,0
(D)LHAHKLRVD(P)	99 %	DP	59.77	45.35914	36.55	2	544,8194	1 087,6242	2	-0,0009219	-0,8469	1 343,27	52 115,0
(D)LHAHKLRVD(P)	97 %	DP	51.5	45.370377	28.36	2	544,8189	1 087,6232	2	-0,001982	-1,821	790,281	62 471,0
(D)LHAHKLRVD(P)	91 %	DP	44.78	45.553364	23.91	2	544,8181	1 087,6216	2	-0,003582	-3,29	637,423	15 690,0
(D)LHAHKLRVDPVNF(K)	100 %	FK	84.07	46.30377	64.59	2	773,4366	1 544,8585	2	0,0008781	0,568	1 591,37	129126
(D)LHAHKLRVDPVNF(K)	100 %	FK	80.35	46.8135	63.2	2	773,4342	1 544,8538	2	-0,003842	-2,485	1 779,94	51 482,0
(D)LHAHKLRVDPVNF(K)	100 %	FK	76.28	46.602013	62.12	2	773,436	1 544,8573	2	-0,0003219	-0,2083	1 323,43	204966
(D)LHAHKLRVDPVNF(K)	100 %	FK	65.62	46.609226	38.63	2	773,4356	1 544,8567	2	-0,0009819	-0,6352	1 321,18	65 303,0
(D)LHAHKLRVDPVNF(K)	98 %	FK	60.82	50.98986	50.6	2	773,4365	1 544,8584	2	0,0006981	0,4516	1 592,62	56 306,0
(D)LHAHKLRVDPVNF(K)	98 %	FK	55.05	46.53531	45.04	2	773,435	1 544,8555	2	-0,002162	-1,399	1 722,47	76 991,0
(D)LHAHKLRVDPVNF(K)	95 %	FK	49.88	46.350513	39.33	2	773,4363	1 544,8580	2	0,0003781	0,2446	1 414,47	179662
(H)AHKLRVDPVNF(K)	100 %	FK	85.45	45.622574	68.77	2	648,3634	1 294,7123	2	-0,002382	-1,838	1 152,58	112304
(H)AHKLRVDPVNF(K)	100 %	FK	66.82	46.04798	50.02	2	648,3621	1 294,7097	2	-0,004962	-3,829	1 342,10	461720
(H)AHKLRVDPVNF(K)	100 %	FK	66.5	45.766754	56.24	2	648,3641	1 294,7136	2	-0,001042	-0,8041	1 185,92	110567
(H)AHKLRVDPVNF(K)	100 %	FK	66.43	45.779053	49.95	2	648,3645	1 294,7144	2	-0,0002619	-0,2022	1 161,52	133468
(H)AHKLRVDPVNF(K)	97 %	FK	53.2	45.779167	38.44	2	648,3643	1 294,7141	2	-0,0006019	-0,4646	1 341,18	180802
(A)HKLRVDPVNF(K)	99 %	FK	61.06	45.321045	44.0	2	612,8462	1 223,6778	2	0,0002181	0,1781	1 156,76	195805
(A)HKLRVDPVNF(K)	95 %	FK	48.77	45.49555	33.37	2	612,845	1 223,6754	2	-0,002182	-1,782	1 164,94	239269
(A)HKLRVDPVNF(K)	93 %	FK	46.43	45.49543	31.53	2	612,8453	1 223,6761	2	-0,001442	-1,177	1 213,90	51 768,0
(A)HKLRVDPVNF(K)	92 %	FK	45.29	45.30277	27.33	2	612,8457	1 223,6769	2	-0,0006619	-0,5405	1 342,60	93 413,0
(K)LLSHCLLVTLAAHLPAEFTPAVHASLDKF(L)	100 %	FL	102.09	53.557007	101.94	2	779,4212	3 113,6558	4	-0,01809	-5,809	2 016,78	597257
(K)LLSHCLLVTLAAHLPAEFTPAVHASLDKF(L)	100 %	FL	74.53	53.431053	72.44	2	779,4222	3 113,6599	4	-0,01401	-4,499	2 014,44	860740
(L)LVTLAAHLPAEFT(P)	93 %	TP	47.16	45.978817	37.01	2	691,8878	1 381,7610	2	0,0003781	0,2734	2 066,97	42 001,0
(L)LVTLAAHLPAEFT(P)	92 %	TP	45.95	46.19834	32.63	2	691,8887	1 381,7628	2	0,002138	1,546	2 066,14	70 151,0
(L)VTLAAHLPAEFTPAVHASLDKF(L)	100 %	FL	154.2	49.538902	136.52	2	779,0874	2 334,2403	3	0,002882	1,234	2 015,69	1110820
(L)VTLAAHLPAEFTPAVHASLDKF(L)	100 %	FL	154.47	48.676563	141.12	2	779,0882	2 334,2426	3	0,005252	2,249	2 015,61	1030550
(L)VTLAAHLPAEFTPAVHASLDKF(L)	100 %	FL	151.32	48.45222	139.24	2	1 168,1316	2 334,2486	2	0,01122	4,804	2 016,78	564456
(L)VTLAAHLPAEFTPAVHASLDKF(L)	100 %	FL	125.26	48.9244	113.83	2	779,0853	2 334,2341	3	-0,003298	-1,412	2 012,10	172594
(L)VTLAAHLPAEFTPAVHASLDKF(L)	100 %	FL	124.31	48.715088	112.27	2	779,0876	2 334,2410	3	0,003632	1,555	2 013,27	477943
(L)VTLAAHLPAEFTPAVHASLDKF(L)	100 %	FL	122.33	48.709187	111.92	2	779,0878	2 334,2415	3	0,004082	1,748	2 015,61	711971
(L)VTLAAHLPAEFTPAVHASLDKF(L)	100 %	FL	115.55	48.92562	103.1	2	779,0852	2 334,2337	3	-0,003688	-1,579	2 014,44	163143
(L)VTLAAHLPAEFTPAVHASLDKF(L)	100 %	FL	101.31	48.86846	86.72	2	779,0867	2 334,2384	3	0,001022	0,4377	2 037,80	78 040,0
(L)VTLAAHLPAEFTPAVHASLDKF(L)	100 %	FL	71.7	48.57881	66.37	2	1 168,1294	2 334,2443	2	0,006918	2,962	2 014,44	126752
(L)VTLAAHLPAEFTPAVHASLDKF(L)	99 %	FL	66.25	48.76818	51.49	2	779,0871	2 334,2394	3	0,002072	0,8873	2 098,62	24 442,0
(L)VTLAAHLPAEFTPAVHASLDKF(L)	99 %	FL	69.07	52.3568	62.11	2	1 168,1297	2 334,2449	2	0,007498	3,211	2 013,36	76 358,0
(L)VTLAAHLPAEFTPAVHASLDKF(L)	99 %	FL	62.01	49.05715	52.6	2	584,5651	2 334,2315	4	-0,005914	-2,532	2 013,27	52 179,0
(L)VTLAAHLPAEFTPAVHASLDKF(L)	94 %	FL	50.95	49.080505	41.82	2	779,0842	2 334,2308	3	-0,006598	-2,825	2 048,33	24 972,0
(V)TLAAHLPAEFTPAVHASLDKF(L)	100 %	FL	110.01	49.214317	99.93	2	746,0633	2 235,1681	3	-0,0008679	-0,3881	2 097,46	67 265,0
(V)TLAAHLPAEFTPAVHASLDKF(L)	100 %	FL	108.33	49.214157	98.42	2	746,0633	2 235,1682	3	-0,0007779	-0,3479	2 016,86	200077
(V)TLAAHLPAEFTPAVHASLDKF(L)	100 %	FL	104.09	49.031494	90.92	2	746,0642	2 235,1708	3	0,001862	0,8327	2 014,52	126954
(V)TLAAHLPAEFTPAVHASLDKF(L)	100 %	FL	85.0	49.351646	68.7	2	746,061	2 235,1613	3	-0,007678	-3,434	2 013,61	61 223,0
(V)TLAAHLPAEFTPAVHASLDKF(L)	100 %	FL	80.93	49.218163	68.25	2	746,0632	2 235,1677	3	-0,001318	-0,5894	2 015,86	57 104,0
(V)TLAAHLPAEFTPAVHASLDKF(L)	100 %	FL	69.97	49.32692	59.52	2	746,0606	2 235,1599	3	-0,009088	-4,064	1 967,76	52 178,0
(T)LAAHLPAEFTPAVHASLDKFL(A)	100 %	LA	98.38	48.30127	83.16	2	750,0758	2 247,2055	3	0,0001121	0,04984	2 099,63	299906
(T)LAAHLPAEFTPAVHASLDKFL(A)	100 %	LA	90.04	48.2904	74.84	2	750,0758	2 247,2057	3	0,0002921	0,1299	2 097,29	122479
(T)LAAHLPAEFTPAVHASLDKFL(A)	99 %	LA	62.38	48.452778	50.99	2	562,8075	2 247,2007	4	-0,004634	-2,061	2 099,63	101562

(T)LAAHLPAEFTPAVHASLDKFL(A)	96 %	LA	52.94	48.136944	43.43	2	750,0761	2 247,2066	3	0,001192	0,5302	2 096,21	36 015,0
(L)AAHLPAEFT(P)	96 %	TP	51.87	47.001236	31.33	2	956,4805	955,4733	1	-0,003106	-3,247	1 456,29	5 974,00
(L)AAHLPAEFT(P)	95 %	TP	49.39	47.00089	31.02	2	956,4816	955,4743	1	-0,002096	-2,191	1 456,45	12 468,0
(L)AAHLPAEFT(P)	93 %	TP	47.51	46.666954	29.69	2	956,479	955,4717	1	-0,004646	-4,857	1 463,36	5 841,00
(L)AAHLPAEFT(P)	93 %	TP	47.22	46.73353	29.75	2	956,4802	955,4729	1	-0,003446	-3,603	1 458,61	9 863,00
(A)HLPAEFTPAVHASLDKF(L)	100 %	FL	104.41	49.091705	94.02	2	627,3278	1 878,9617	3	-0,001308	-0,6957	1 820,68	144804
(A)HLPAEFTPAVHASLDKF(L)	100 %	FL	92.3	49.066635	77.25	2	940,4863	1 878,9580	2	-0,004922	-2,618	1 846,35	278167
(A)HLPAEFTPAVHASLDKF(L)	100 %	FL	87.78	49.00061	78.05	2	940,4885	1 878,9624	2	-0,0005219	-0,2776	2 015,61	120747
(A)HLPAEFTPAVHASLDKF(L)	100 %	FL	82.93	49.011913	68.91	2	627,3281	1 878,9625	3	-0,0004679	-0,2489	1 824,19	250621
(A)HLPAEFTPAVHASLDKF(L)	100 %	FL	82.15	49.1668	68.45	2	940,4852	1 878,9558	2	-0,007202	-3,831	1 926,82	113234
(A)HLPAEFTPAVHASLDKF(L)	100 %	FL	80.57	49.148453	68.39	2	940,4891	1 878,9637	2	0,0007181	0,382	1 821,85	253114
(A)HLPAEFTPAVHASLDKF(L)	100 %	FL	76.21	49.138985	65.37	2	627,3287	1 878,9642	3	0,001212	0,6447	1 821,85	434204
(A)HLPAEFTPAVHASLDKF(L)	100 %	FL	79.17	53.777905	66.9	2	627,3272	1 878,9597	3	-0,003288	-1,749	1 846,44	359321
(A)HLPAEFTPAVHASLDKF(L)	100 %	FL	71.37	49.037247	58.21	2	627,3279	1 878,9619	3	-0,001098	-0,584	1 820,01	106528
(A)HLPAEFTPAVHASLDKF(L)	100 %	FL	70.96	49.224922	62.84	2	627,3273	1 878,9600	3	-0,002988	-1,589	1 925,90	66 642,0
(A)HLPAEFTPAVHASLDKF(L)	100 %	FL	70.21	49.09155	54.73	2	940,488	1 878,9614	2	-0,001562	-0,8308	1 820,77	54 825,0
(A)HLPAEFTPAVHASLDKF(L)	100 %	FL	74.71	53.777103	60.84	2	627,327	1 878,9593	3	-0,003678	-1,956	1 848,77	256718
(A)HLPAEFTPAVHASLDKF(L)	100 %	FL	69.02	49.187695	61.97	2	940,487	1 878,9594	2	-0,003582	-1,905	2 014,77	80 370,0
(A)HLPAEFTPAVHASLDKF(L)	100 %	FL	68.84	49.064484	55.03	2	627,3267	1 878,9584	3	-0,004578	-2,435	1 930,32	92 244,0
(A)HLPAEFTPAVHASLDKF(L)	100 %	FL	73.09	53.767357	68.43	2	627,3279	1 878,9618	3	-0,001158	-0,6159	1 833,54	64 668,0
(A)HLPAEFTPAVHASLDKF(L)	100 %	FL	68.46	49.21265	55.85	2	627,3265	1 878,9576	3	-0,005418	-2,882	1 822,02	166972
(A)HLPAEFTPAVHASLDKF(L)	99 %	FL	64.08	49.18455	54.4	2	940,4869	1 878,9591	2	-0,003822	-2,033	1 934,98	60 464,0
(A)HLPAEFTPAVHASLDKF(L)	99 %	FL	63.6	49.2165	56.12	2	627,326	1 878,9561	3	-0,006858	-3,648	2 016,86	48 119,0
(A)HLPAEFTPAVHASLDKF(L)	93 %	FL	50.0	49.187485	40.84	2	627,3271	1 878,9595	3	-0,003438	-1,829	1 927,07	52 396,0
(A)HLPAEFTPAVHASLDKFL(A)	100 %	LA	72.75	48.896156	62.93	2	665,0226	1 992,0461	3	-0,0009779	-0,4907	2 033,21	339883
(A)HLPAEFTPAVHASLDKFL(A)	96 %	LA	52.75	48.779354	42.44	2	665,0227	1 992,0464	3	-0,0007079	-0,3552	2 034,38	93 301,0
(H)LPAEFTPAVHASLDKF(L)	100 %	FL	109.25	48.545975	90.25	2	871,96	1 741,9053	2	0,001278	0,7333	1 927,98	643974
(H)LPAEFTPAVHASLDKF(L)	100 %	FL	95.32	48.652344	80.36	2	871,958	1 741,9015	2	-0,002562	-1,47	1 925,65	177852
(H)LPAEFTPAVHASLDKF(L)	100 %	FL	93.6	48.533394	78.32	2	871,9623	1 741,9100	2	0,005918	3,395	1 827,78	567799
(H)LPAEFTPAVHASLDKF(L)	100 %	FL	93.54	48.769794	77.98	2	871,9593	1 741,9040	2	-0,00004194	-0,02406	1 852,18	346359
(H)LPAEFTPAVHASLDKF(L)	100 %	FL	88.96	48.769794	70.43	2	871,9593	1 741,9040	2	-0,00004194	-0,02406	1 824,19	117379
(H)LPAEFTPAVHASLDKF(L)	100 %	FL	84.32	48.68515	66.87	2	871,9587	1 741,9029	2	-0,001202	-0,6896	2 015,69	141828
(H)LPAEFTPAVHASLDKF(L)	99 %	FL	66.05	48.7642	46.41	2	871,9562	1 741,8979	2	-0,006142	-3,524	1 984,22	27 021,0
(H)LPAEFTPAVHASLDKF(L)	98 %	FL	57.41	48.802475	43.22	2	871,9571	1 741,8997	2	-0,004382	-2,514	1 967,76	22 935,0
(H)LPAEFTPAVHASLDKFL(A)	100 %	LA	93.44	48.240738	70.24	2	928,5021	1 854,9897	2	0,001558	0,8395	2 032,29	92 445,0
(H)LPAEFTPAVHASLDKFL(A)	100 %	LA	75.43	48.13768	63.86	2	928,501	1 854,9874	2	-0,0007819	-0,4213	2 033,13	228517
(L)PAEFTPAVHASLDKF(L)	100 %	FL	105.16	48.779816	92.77	2	815,4187	1 628,8229	2	0,002898	1,778	1 926,82	792174
(L)PAEFTPAVHASLDKF(L)	100 %	FL	104.19	48.925953	92.73	2	815,4163	1 628,8179	2	-0,002022	-1,241	1 824,19	887039
(L)PAEFTPAVHASLDKF(L)	100 %	FL	98.65	48.931458	88.45	2	815,4164	1 628,8183	2	-0,001702	-1,044	2 015,61	317965
(L)PAEFTPAVHASLDKF(L)	100 %	FL	96.15	48.861126	72.14	2	543,9466	1 628,8178	3	-0,002148	-1,318	1 799,60	177021
(L)PAEFTPAVHASLDKF(L)	100 %	FL	95.78	48.84478	80.28	2	815,4174	1 628,8203	2	0,0003181	0,1951	1 848,68	945033
(L)PAEFTPAVHASLDKF(L)	100 %	FL	95.7	48.861805	82.66	2	815,4137	1 628,8128	2	-0,007182	-4,407	1 751,68	204969
(L)PAEFTPAVHASLDKF(L)	100 %	FL	95.31	48.718704	82.64	2	815,4172	1 628,8199	2	-0,0001019	-0,06255	2 120,76	302355
(L)PAEFTPAVHASLDKF(L)	100 %	FL	101.11	54.716347	88.54	2	815,4174	1 628,8202	2	0,0001981	0,1215	1 798,43	128428
(L)PAEFTPAVHASLDKF(L)	100 %	FL	94.75	48.728146	84.65	2	815,4171	1 628,8195	2	-0,0004219	-0,2589	1 925,73	281915
(L)PAEFTPAVHASLDKF(L)	100 %	FL	97.69	54.70929	86.14	2	815,4173	1 628,8201	2	0,0001381	0,08471	2 121,02	215299

(L)PAEFTPAVHASLDKF(L)	100 %	FL	90.43	48.931458	77.12	2	815,4164	1 628,8182	2	-0,001742	-1,069	1 797,27	87 402,0
(L)PAEFTPAVHASLDKF(L)	100 %	FL	93.03	54.666866	82.59	2	815,4172	1 628,8198	2	-0,0001219	-0,07482	1 930,32	946618
(L)PAEFTPAVHASLDKF(L)	100 %	FL	86.27	48.84478	73.42	2	815,4174	1 628,8202	2	0,0002781	0,1706	1 827,69	338427
(L)PAEFTPAVHASLDKF(L)	100 %	FL	91.55	54.67848	76.96	2	815,4186	1 628,8226	2	0,002658	1,631	1 853,68	621003
(L)PAEFTPAVHASLDKF(L)	100 %	FL	91.22	54.697945	78.26	2	815,4167	1 628,8188	2	-0,001142	-0,7007	1 828,03	168779
(L)PAEFTPAVHASLDKF(L)	100 %	FL	82.45	48.728085	69.61	2	815,4172	1 628,8198	2	-0,0002019	-0,1239	1 994,66	101499
(L)PAEFTPAVHASLDKF(L)	100 %	FL	88.46	54.749546	79.15	2	815,4165	1 628,8184	2	-0,001542	-0,9461	1 927,32	100235
(L)PAEFTPAVHASLDKF(L)	100 %	FL	82.59	48.892178	72.68	2	815,4177	1 628,8209	2	0,0009381	0,5756	1 799,60	615395
(L)PAEFTPAVHASLDKF(L)	100 %	FL	87.13	54.719395	77.54	2	815,4159	1 628,8172	2	-0,002722	-1,67	1 824,27	499934
(L)PAEFTPAVHASLDKF(L)	100 %	FL	80.13	48.925232	68.55	2	815,4163	1 628,8180	2	-0,001922	-1,179	2 117,37	69 651,0
(L)PAEFTPAVHASLDKF(L)	100 %	FL	85.66	54.749382	65.64	2	815,4165	1 628,8184	2	-0,001522	-0,9338	1 926,15	178686
(L)PAEFTPAVHASLDKF(L)	100 %	FL	79.12	48.850727	59.32	2	543,9473	1 628,8200	3	0,00001206	0,007398	1 799,60	258473
(L)PAEFTPAVHASLDKF(L)	100 %	FL	84.56	54.713974	66.59	2	815,4177	1 628,8208	2	0,0008781	0,5387	1 856,86	248786
(L)PAEFTPAVHASLDKF(L)	100 %	FL	77.23	54.673786	67.07	2	815,4171	1 628,8195	2	-0,0004219	-0,2589	1 929,23	153916
(L)PAEFTPAVHASLDKF(L)	99 %	FL	72.28	54.71268	62.26	2	815,4175	1 628,8204	2	0,0004781	0,2933	1 951,56	85 960,0
(L)PAEFTPAVHASLDKF(L)	99 %	FL	63.76	48.691086	57.41	2	815,4152	1 628,8158	2	-0,004122	-2,529	1 820,68	85 847,0
(L)PAEFTPAVHASLDKF(L)	99 %	FL	61.29	48.8361	48.27	2	543,9462	1 628,8169	3	-0,003078	-1,889	1 796,35	34 160,0
(L)PAEFTPAVHASLDKF(L)	99 %	FL	66.17	54.694157	55.66	2	815,4167	1 628,8188	2	-0,001122	-0,6884	2 015,69	48 264,0
(L)PAEFTPAVHASLDKF(L)	99 %	FL	60.36	48.88786	49.54	2	815,4176	1 628,8207	2	0,0007581	0,4651	1 970,18	34 366,0
(L)PAEFTPAVHASLDKF(L)	98 %	FL	57.87	48.811222	50.84	2	543,9469	1 628,8188	3	-0,001158	-0,7105	2 014,52	27 215,0
(L)PAEFTPAVHASLDKF(L)	98 %	FL	56.68	48.82553	45.2	2	543,9456	1 628,8148	3	-0,005148	-3,159	2 016,78	85 783,0
(L)PAEFTPAVHASLDKF(L)	96 %	FL	53.05	48.882637	44.92	2	815,4159	1 628,8172	2	-0,002742	-1,682	1 912,83	42 421,0
(L)PAEFTPAVHASLDKF(L)	93 %	FL	49.53	48.90091	39.83	2	543,9454	1 628,8143	3	-0,005718	-3,508	1 797,27	40 356,0
(L)PAEFTPAVHASLDKFL(A)	100 %	LA	88.84	48.690086	65.64	2	871,961	1 741,9073	2	0,003278	1,881	2 020,28	420109
(L)PAEFTPAVHASLDKFL(A)	99 %	LA	62.68	48.8488	40.98	2	581,6404	1 741,8995	3	-0,004608	-2,644	2 019,11	174770
(P)AEFTPAVHASLDKF(L)	100 %	FL	89.11	48.78384	70.85	2	766,8899	1 531,7653	2	-0,001882	-1,228	1 835,88	212025
(P)AEFTPAVHASLDKF(L)	100 %	FL	74.17	48.653175	62.53	2	766,8915	1 531,7685	2	0,001338	0,873	2 015,86	51 252,0
(P)AEFTPAVHASLDKF(L)	100 %	FL	71.45	48.768238	56.57	2	766,8893	1 531,7640	2	-0,003122	-2,037	1 765,80	122980
(P)AEFTPAVHASLDKF(L)	98 %	FL	59.24	48.766853	46.49	2	766,8891	1 531,7637	2	-0,003442	-2,246	1 926,15	53 449,0
(P)AEFTPAVHASLDKF(L)	98 %	FL	62.36	52.819466	50.23	2	766,885	1 531,7554	2	-0,01172	-7,648	1 798,93	87 064,0
(P)AEFTPAVHASLDKF(L)	98 %	FL	58.28	48.763966	37.73	2	766,8902	1 531,7658	2	-0,001362	-0,8885	1 799,60	36 452,0
(P)AEFTPAVHASLDKF(L)	98 %	FL	56.97	48.652695	45.53	2	766,8881	1 531,7616	2	-0,005542	-3,616	1 929,32	55 162,0
(P)AEFTPAVHASLDKF(L)	94 %	FL	50.38	48.653885	40.21	2	766,888	1 531,7615	2	-0,005682	-3,707	1 824,35	42 931,0
(P)AEFTPAVHASLDKFL(A)	99 %	LA	65.08	48.727154	47.0	2	823,4343	1 644,8541	2	0,002818	1,712	1 999,50	79 663,0
(P)AEFTPAVHASLDKFL(A)	99 %	LA	63.65	48.96179	45.51	2	823,4301	1 644,8457	2	-0,005582	-3,392	2 020,28	32 545,0
(A)EFTPAVHASLDKF(L)	100 %	FL	81.83	48.6676	64.01	2	731,3686	1 460,7227	2	-0,007342	-5,023	1 800,77	101240
(A)EFTPAVHASLDKF(L)	100 %	FL	80.87	48.708076	67.27	2	731,3701	1 460,7257	2	-0,004362	-2,984	1 925,73	50 530,0
(A)EFTPAVHASLDKF(L)	100 %	FL	75.81	48.67314	57.77	2	731,3704	1 460,7262	2	-0,003882	-2,656	1 828,87	192797
(A)EFTPAVHASLDKF(L)	100 %	FL	72.81	48.730415	51.06	2	731,37	1 460,7254	2	-0,004662	-3,189	1 751,85	120412
(A)EFTPAVHASLDKF(L)	100 %	FL	68.66	48.627277	56.57	2	731,3705	1 460,7263	2	-0,003722	-2,546	1 798,43	29 707,0
(A)EFTPAVHASLDKF(L)	98 %	FL	62.39	54.40448	44.3	2	731,3693	1 460,7241	2	-0,005982	-4,092	1 800,93	42 402,0
(A)EFTPAVHASLDKF(L)	98 %	FL	62.28	54.453083	49.37	2	731,3706	1 460,7266	2	-0,003442	-2,355	1 831,21	106639
(A)EFTPAVHASLDKF(L)	97 %	FL	55.33	48.9264	42.21	2	731,3742	1 460,7339	2	0,003858	2,639	2 015,61	42 483,0
(A)EFTPAVHASLDKFL(A)	100 %	LA	73.06	48.86248	57.39	2	787,9132	1 573,8118	2	-0,002362	-1,5	1 992,48	84 764,0
(A)EFTPAVHASLDKFL(A)	100 %	LA	68.23	48.88679	52.86	2	787,9131	1 573,8116	2	-0,002542	-1,614	1 993,40	133293
(F)TPAVHASLDKF(L)	100 %	FL	80.94	47.474743	60.67	2	593,3148	1 184,6149	2	-0,004122	-3,477	1 828,87	307111

(F)TPAVHASLDKF(L)	100 %	FL	73.94	47.236282	55.22	2	593,3169	1 184,6192	2	0,0001781	0,1502	1 679,10	145586
(F)TPAVHASLDKF(L)	100 %	FL	73.84	47.49582	54.42	2	593,3158	1 184,6170	2	-0,002022	-1,705	1 349,11	570554
(F)TPAVHASLDKF(L)	100 %	FL	73.7	47.501225	58.12	2	593,3162	1 184,6178	2	-0,001302	-1,098	1 924,82	51 394,0
(F)TPAVHASLDKF(L)	100 %	FL	73.53	47.501225	56.38	2	593,3162	1 184,6178	2	-0,001302	-1,098	1 852,18	169093
(F)TPAVHASLDKF(L)	100 %	FL	68.7	47.50362	50.1	2	593,316	1 184,6174	2	-0,001662	-1,402	1 929,15	243831
(F)TPAVHASLDKF(L)	100 %	FL	73.78	53.444942	55.3	2	593,316	1 184,6174	2	-0,001682	-1,419	1 830,03	156940
(F)TPAVHASLDKF(L)	100 %	FL	73.47	53.455715	55.17	2	593,3163	1 184,6180	2	-0,001042	-0,8788	1 678,25	235144
(F)TPAVHASLDKF(L)	99 %	FL	71.74	53.434734	52.24	2	593,3152	1 184,6159	2	-0,003182	-2,684	1 930,48	34 892,0
(F)TPAVHASLDKF(L)	99 %	FL	64.9	47.411198	48.09	2	593,3152	1 184,6159	2	-0,003182	-2,684	1 348,02	204144
(F)TPAVHASLDKF(L)	99 %	FL	61.69	47.333015	43.39	2	593,3148	1 184,6151	2	-0,003982	-3,359	2 015,77	143047
(F)TPAVHASLDKF(L)	99 %	FL	61.77	47.42725	47.88	2	593,3154	1 184,6163	2	-0,002762	-2,33	1 925,65	97 910,0
(F)TPAVHASLDKF(L)	99 %	FL	66.44	53.36292	47.97	2	593,3174	1 184,6202	2	0,001158	0,9767	1 349,27	582894
(F)TPAVHASLDKF(L)	99 %	FL	65.43	53.40807	48.65	2	593,3157	1 184,6168	2	-0,002262	-1,908	1 350,27	133055
(F)TPAVHASLDKF(L)	99 %	FL	65.0	53.438004	48.12	2	593,3149	1 184,6151	2	-0,003922	-3,308	1 834,71	51 758,0
(F)TPAVHASLDKF(L)	98 %	FL	64.28	53.378166	52.07	2	593,3164	1 184,6183	2	-0,0007419	-0,6258	1 932,65	148677
(F)TPAVHASLDKF(L)	98 %	FL	57.54	47.329643	40.7	2	593,3149	1 184,6153	2	-0,003742	-3,156	2 014,61	53 830,0
(F)TPAVHASLDKF(L)	97 %	FL	60.16	53.482018	39.84	2	593,3148	1 184,6149	2	-0,004122	-3,477	1 926,90	26 501,0
(F)TPAVHASLDKF(L)	96 %	FL	51.15	47.366753	35.43	2	593,3152	1 184,6158	2	-0,003282	-2,768	1 799,76	25 522,0
(F)TPAVHASLDKFL(A)	100 %	LA	96.45	46.446156	74.3	2	649,858	1 297,7015	2	-0,001702	-1,31	1 786,77	677340
(F)TPAVHASLDKFL(A)	100 %	LA	74.09	46.94728	58.76	2	649,8605	1 297,7065	2	0,003358	2,586	2 032,13	65 642,0
(F)TPAVHASLDKFL(A)	100 %	LA	70.03	46.605995	39.73	2	649,8563	1 297,6981	2	-0,005082	-3,913	1 785,61	92 621,0
(F)TPAVHASLDKFL(A)	100 %	LA	66.29	46.976475	45.26	2	649,8558	1 297,6971	2	-0,006082	-4,683	1 786,94	41 624,0
(F)TPAVHASLDKFL(A)	99 %	LA	62.89	46.660686	40.91	2	649,8594	1 297,7042	2	0,001018	0,7839	2 033,21	259463
(F)TPAVHASLDKFLASVS(T)	100 %	ST	110.73	48.06289	93.18	2	821,9438	1 641,8730	2	0,0002981	0,1814	1 963,00	294638
(F)TPAVHASLDKFLASVS(T)	100 %	ST	69.2	48.32739	56.68	2	821,9432	1 641,8718	2	-0,0008819	-0,5368	1 961,83	61 728,0
(F)TPAVHASLDKFLASVST(V)	100 %	TV	70.5	48.568253	56.58	2	872,4676	1 742,9206	2	0,0002181	0,125	1 968,84	63 367,0
(F)TPAVHASLDKFLASVST(V)	92 %	TV	48.64	48.566864	41.51	2	872,4675	1 742,9205	2	0,0001181	0,0677	1 966,59	39 550,0
(T)PAVHASLDKF(L)	100 %	FL	73.45	46.872257	57.18	2	542,7921	1 083,5696	2	-0,001742	-1,606	1 934,98	251098
(T)PAVHASLDKF(L)	100 %	FL	68.12	46.807068	47.28	2	542,7918	1 083,5690	2	-0,002342	-2,159	1 925,65	158377
(T)PAVHASLDKF(L)	100 %	FL	66.9	46.827766	49.07	2	542,7922	1 083,5699	2	-0,001442	-1,329	1 258,15	63 947,0
(T)PAVHASLDKF(L)	100 %	FL	70.89	51.066532	54.58	2	542,79	1 083,5655	2	-0,005842	-5,386	1 243,53	95 673,0
(T)PAVHASLDKF(L)	99 %	FL	64.8	46.782818	45.21	2	542,7916	1 083,5687	2	-0,002642	-2,436	1 246,52	422805
(T)PAVHASLDKF(L)	99 %	FL	68.06	50.98045	53.29	2	542,7923	1 083,5700	2	-0,001362	-1,256	1 838,21	246479
(T)PAVHASLDKF(L)	99 %	FL	62.29	46.782818	44.03	2	542,7917	1 083,5688	2	-0,002602	-2,399	1 677,91	238717
(T)PAVHASLDKF(L)	99 %	FL	62.3	46.807068	43.2	2	542,7918	1 083,5690	2	-0,002382	-2,196	1 244,28	158904
(T)PAVHASLDKF(L)	99 %	FL	61.39	46.78928	39.17	2	542,7913	1 083,5680	2	-0,003362	-3,1	1 680,28	91 646,0
(T)PAVHASLDKF(L)	99 %	FL	61.36	46.807068	40.11	2	542,7919	1 083,5692	2	-0,002162	-1,993	1 831,21	80 647,0
(T)PAVHASLDKF(L)	99 %	FL	59.83	46.807068	37.53	2	542,7918	1 083,5691	2	-0,002282	-2,104	1 933,82	44 351,0
(T)PAVHASLDKF(L)	99 %	FL	59.27	46.782818	28.23	2	542,7917	1 083,5688	2	-0,002582	-2,381	1 679,18	44 595,0
(T)PAVHASLDKF(L)	99 %	FL	58.56	46.807068	38.07	2	542,7918	1 083,5691	2	-0,002302	-2,122	2 014,44	72 373,0
(T)PAVHASLDKF(L)	99 %	FL	58.46	46.78928	43.19	2	542,7914	1 083,5682	2	-0,003202	-2,952	2 119,75	50 245,0
(T)PAVHASLDKF(L)	99 %	FL	58.52	46.86645	45.71	2	542,793	1 083,5714	2	0,00005806	0,05353	1 812,54	26 454,0
(T)PAVHASLDKF(L)	98 %	FL	61.98	50.968582	39.84	2	542,792	1 083,5694	2	-0,001922	-1,772	1 932,65	100814
(T)PAVHASLDKF(L)	98 %	FL	56.7	46.782818	40.32	2	542,7917	1 083,5689	2	-0,002462	-2,27	2 017,95	44 353,0
(T)PAVHASLDKF(L)	98 %	FL	56.61	46.827766	37.67	2	542,7924	1 083,5702	2	-0,001182	-1,09	1 564,74	31 857,0
(T)PAVHASLDKF(L)	98 %	FL	59.88	51.024784	44.01	2	542,7918	1 083,5690	2	-0,002382	-2,196	2 015,77	63 228,0

(T)PAVHASLDKF(L)	98 %	FL	59.8	51.022793	43.16	2	542,7918	1 083,5689	2	-0,002422	-2,233	1 930,48	27 542,0
(T)PAVHASLDKF(L)	98 %	FL	55.28	46.782818	34.65	2	542,7917	1 083,5688	2	-0,002522	-2,325	2 016,78	322519
(T)PAVHASLDKF(L)	98 %	FL	55.14	46.782818	35.92	2	542,7916	1 083,5687	2	-0,002642	-2,436	1 349,11	92 474,0
(T)PAVHASLDKF(L)	97 %	FL	54.05	46.827766	37.4	2	542,7924	1 083,5702	2	-0,001142	-1,053	1 844,02	352737
(T)PAVHASLDKF(L)	97 %	FL	53.26	46.78928	32.11	2	542,7913	1 083,5680	2	-0,003342	-3,081	1 847,52	64 866,0
(T)PAVHASLDKF(L)	96 %	FL	52.09	46.78928	30.62	2	542,7912	1 083,5679	2	-0,003502	-3,229	1 923,40	19 713,0
(T)PAVHASLDKF(L)	96 %	FL	51.85	46.569313	40.35	2	542,7897	1 083,5648	2	-0,006522	-6,013	1 296,61	13 807,0
(T)PAVHASLDKF(L)	96 %	FL	51.93	46.807068	30.6	2	542,7918	1 083,5691	2	-0,002282	-2,104	1 827,69	224804
(T)PAVHASLDKF(L)	96 %	FL	51.85	46.827766	36.69	2	542,7922	1 083,5698	2	-0,001522	-1,403	1 845,19	41 295,0
(T)PAVHASLDKF(L)	95 %	FL	54.18	50.966564	32.25	2	542,7919	1 083,5692	2	-0,002182	-2,012	1 248,85	270283
(T)PAVHASLDKF(L)	93 %	FL	52.01	51.024784	31.99	2	542,7918	1 083,5690	2	-0,002402	-2,215	1 245,36	69 612,0
(T)PAVHASLDKF(L)	93 %	FL	47.42	46.827766	24.71	2	542,7923	1 083,5700	2	-0,001342	-1,237	2 097,46	49 073,0
(T)PAVHASLDKF(L)	91 %	FL	45.55	46.782818	26.09	2	542,7916	1 083,5687	2	-0,002642	-2,436	1 350,27	130221
(T)PAVHASLDKFL(A)	100 %	LA	75.17	45.920544	52.98	2	599,3334	1 196,6522	2	-0,003262	-2,724	1 786,77	90 848,0
(T)PAVHASLDKFL(A)	100 %	LA	74.87	46.156975	52.95	2	599,3329	1 196,6512	2	-0,004222	-3,525	1 759,04	165780
(T)PAVHASLDKFL(A)	100 %	LA	70.51	45.85506	47.25	2	599,3331	1 196,6516	2	-0,003822	-3,191	1 759,87	236069
(T)PAVHASLDKFL(A)	100 %	LA	67.42	46.177864	47.88	2	599,3327	1 196,6508	2	-0,004662	-3,893	2 033,13	638917
(T)PAVHASLDKFL(A)	100 %	LA	67.28	46.178177	45.5	2	599,3323	1 196,6500	2	-0,005422	-4,527	2 032,04	253948
(T)PAVHASLDKFL(A)	98 %	LA	62.32	51.51416	48.53	2	599,3343	1 196,6541	2	-0,001382	-1,154	2 033,21	201191
(T)PAVHASLDKFL(A)	98 %	LA	55.36	45.96432	33.38	2	599,334	1 196,6534	2	-0,002102	-1,755	1 928,07	198991
(P)AVHASLDKF(L)	99 %	FL	64.21	48.32739	36.75	2	494,266	986,5173	2	-0,001222	-1,237	1 828,87	36 032,0
(P)AVHASLDKF(L)	99 %	FL	61.17	47.542183	32.89	2	494,2665	986,5183	2	-0,0002219	-0,2247	1 128,76	54 651,0
(P)AVHASLDKF(L)	98 %	FL	58.48	48.32579	31.24	2	494,2654	986,5162	2	-0,002382	-2,412	1 131,09	105334
(P)AVHASLDKF(L)	98 %	FL	56.18	48.32579	28.51	2	494,2652	986,5158	2	-0,002782	-2,817	1 351,44	34 454,0
(P)AVHASLDKF(L)	96 %	FL	53.37	48.127533	20.76	2	987,521	986,5137	1	-0,004856	-4,917	1 839,45	5 576,00
**Beta-subunit**													
(-)MVHLTPEEKSAVTAL(W)	100 %	LW	81.75	48.10031	66.75	2	813,433	1 624,8515	2	0,001938	1,192	1 687,49	139878
(-)MVHLTPEEKSAVTAL(W)	99 %	LW	65.42	48.419098	52.09	2	813,4295	1 624,8443	2	-0,005222	-3,212	1 689,66	256124
(-)MVHLTPEEKSAVTAL(W)	99 %	LW	60.93	48.05786	47.06	2	813,4326	1 624,8506	2	0,0009981	0,6139	1 686,65	109113
(V)HLTPEEKSAVTAL(W)	100 %	LW	78.28	47.551426	57.06	2	698,3773	1 394,7401	2	-0,0005419	-0,3883	1 430,65	105489
(V)HLTPEEKSAVTAL(W)	100 %	LW	72.98	47.53721	48.15	2	698,3768	1 394,7390	2	-0,001642	-1,176	1 512,17	91 386,0
(V)HLTPEEKSAVTAL(W)	99 %	LW	63.05	47.20002	38.72	2	698,3781	1 394,7416	2	0,0009181	0,6578	1 455,29	201007
(V)HLTPEEKSAVTAL(W)	99 %	LW	62.39	47.470932	42.36	2	698,3787	1 394,7428	2	0,002138	1,532	1 463,28	192981
(V)HLTPEEKSAVTAL(W)	98 %	LW	57.87	47.11453	38.39	2	698,3794	1 394,7442	2	0,003518	2,521	1 308,28	429112
(V)HLTPEEKSAVTAL(W)	96 %	LW	52.25	47.047768	30.26	2	698,3796	1 394,7446	2	0,003958	2,836	1 487,72	194851
(V)HLTPEEKSAVTAL(W)	93 %	LW	48.21	47.504005	31.05	2	698,3767	1 394,7388	2	-0,001862	-1,334	1 305,95	114578
(V)HLTPEEKSAVTAL(W)	91 %	LW	46.37	47.551426	28.89	2	698,3774	1 394,7402	2	-0,0005019	-0,3596	1 310,61	76 145,0
(L)TPEEKSAVTAL(W)	100 %	LW	78.04	48.36742	50.91	2	1 145,6031	1 144,5958	1	-0,001826	-1,594	1 508,85	5 162,00
(L)TPEEKSAVTAL(W)	99 %	LW	64.94	48.212646	42.31	2	1 145,6012	1 144,5940	1	-0,003716	-3,244	1 433,07	7 871,00
(L)TPEEKSAVTAL(W)	99 %	LW	62.64	48.373486	31.61	2	573,3037	1 144,5929	2	-0,004742	-4,139	1 219,82	38 277,0
(L)TPEEKSAVTAL(W)	97 %	LW	53.85	48.36742	29.49	2	573,3053	1 144,5961	2	-0,001562	-1,363	1 220,90	140258
(L)TPEEKSAVTAL(W)	96 %	LW	58.04	53.61662	37.01	2	573,3048	1 144,5950	2	-0,002662	-2,324	1 219,98	47 692,0
(L)TPEEKSAVTAL(W)	95 %	LW	51.73	48.473934	29.13	2	1 145,6003	1 144,5930	1	-0,004626	-4,038	1 457,45	4 567,00
(L)TPEEKSAVTALWGKVNVDEVGGEAL(G)	100 %	LG	72.43	50.16858	55.76	2	867,1121	2 598,3143	3	-0,003648	-1,403	2 047,32	24 034,0
(L)TPEEKSAVTALWGKVNVDEVGGEAL(G)	92 %	LG	49.77	50.142433	32.78	2	867,1145	2 598,3218	3	0,003792	1,459	2 046,49	38 439,0
(T)PEEKSAVTAL(W)	99 %	LW	59.49	47.879276	35.55	2	1 044,5559	1 043,5486	1	-0,001356	-1,298	1 481,90	5 241,00

(T)PEEKSAVTAL(W)	98 %	LW	58.6	48.203854	31.68	2	1 044,5545	1 043,5472	1	-0,002736	-2,619	1 456,29	11 506,0
(T)PEEKSAVTAL(W)	98 %	LW	56.71	47.09982	40.83	2	1 044,5521	1 043,5448	1	-0,005176	-4,955	1 492,70	7 158,00
(T)PEEKSAVTAL(W)	98 %	LW	56.94	47.925804	28.95	2	1 044,5552	1 043,5479	1	-0,002096	-2,007	1 431,90	6 982,00
(T)PEEKSAVTAL(W)	98 %	LW	57.11	48.21926	29.04	2	1 044,5578	1 043,5505	1	0,0005641	0,54	1 504,19	6 787,00
(T)PEEKSAVTAL(W)	98 %	LW	56.54	48.144676	34.85	2	522,7812	1 043,5478	2	-0,002202	-2,108	1 550,58	65 248,0
(T)PEEKSAVTAL(W)	97 %	LW	54.76	47.925877	30.33	2	1 044,5556	1 043,5483	1	-0,001696	-1,624	1 432,98	14 631,0
(T)PEEKSAVTAL(W)	97 %	LW	54.04	48.106693	17.57	2	1 044,5539	1 043,5466	1	-0,003396	-3,251	1 434,15	13 410,0
(T)PEEKSAVTAL(W)	96 %	LW	52.28	47.804684	30.55	2	1 044,5589	1 043,5516	1	0,001674	1,603	1 510,34	6 238,00
(T)PEEKSAVTAL(W)	96 %	LW	52.04	47.925877	29.45	2	1 044,5557	1 043,5484	1	-0,001586	-1,518	1 431,82	14 041,0
(T)PEEKSAVTAL(W)	96 %	LW	52.04	48.23631	20.57	2	1 044,5581	1 043,5509	1	0,0008941	0,8559	1 459,78	5 009,00
(T)PEEKSAVTAL(W)	94 %	LW	49.57	48.199123	25.93	2	1 044,5550	1 043,5478	1	-0,002206	-2,112	1 431,82	9 632,00
(T)ALWGKVNVDEVGGEALGRL(L)	100 %	LL	111.2	48.243732	93.3	2	992,0366	1 982,0586	2	-0,0001219	-0,06149	2 033,13	295141
(T)ALWGKVNVDEVGGEALGRL(L)	100 %	LL	84.73	48.060104	71.47	2	992,0372	1 982,0598	2	0,0009981	0,5033	2 031,96	82 152,0
(L)WGKVNVDEVGGEAL(G)	100 %	LG	116.13	48.60697	91.08	2	736,8728	1 471,7310	2	0,00009806	0,06658	1 796,10	194644
(L)WGKVNVDEVGGEAL(G)	100 %	LG	109.5	48.569645	90.58	2	736,8725	1 471,7305	2	-0,0003819	-0,2593	1 797,27	718897
(L)WGKVNVDEVGGEAL(G)	100 %	LG	112.68	52.634064	94.36	2	736,8725	1 471,7304	2	-0,0004419	-0,3001	2 104,38	51 884,0
(L)WGKVNVDEVGGEAL(G)	100 %	LG	106.18	48.36786	78.84	2	736,8711	1 471,7277	2	-0,003182	-2,161	1 787,94	336924
(L)WGKVNVDEVGGEAL(G)	100 %	LG	101.57	48.602425	77.29	2	736,8731	1 471,7316	2	0,0007581	0,5147	1 789,11	714859
(L)WGKVNVDEVGGEAL(G)	100 %	LG	93.57	48.41997	80.41	2	736,8702	1 471,7258	2	-0,005062	-3,437	1 822,02	33 857,0
(L)WGKVNVDEVGGEAL(G)	100 %	LG	92.51	48.421783	77.63	2	736,8703	1 471,7261	2	-0,004742	-3,22	1 919,90	18 324,0
(L)WGKVNVDEVGGEAL(G)	100 %	LG	92.12	48.372665	65.62	2	736,8715	1 471,7283	2	-0,002522	-1,712	1 995,91	57 096,0
(L)WGKVNVDEVGGEAL(G)	100 %	LG	90.63	48.339752	75.17	2	736,8719	1 471,7293	2	-0,001562	-1,061	2 135,09	27 095,0
(L)WGKVNVDEVGGEAL(G)	100 %	LG	88.07	48.45619	66.78	2	736,8695	1 471,7244	2	-0,006422	-4,361	1 787,11	102091
(L)WGKVNVDEVGGEAL(G)	100 %	LG	85.08	48.421722	59.22	2	736,8701	1 471,7255	2	-0,005322	-3,614	1 800,77	60 597,0
(L)WGKVNVDEVGGEAL(G)	100 %	LG	74.66	48.339752	57.15	2	736,872	1 471,7294	2	-0,001442	-0,9791	1 895,53	25 565,0
(L)WGKVNVDEVGGEAL(G)	100 %	LG	71.92	48.38062	55.68	2	1 472,7300	1 471,7228	1	-0,008106	-5,504	1 793,78	6 232,00
(L)WGKVNVDEVGGEAL(G)	100 %	LG	67.25	48.350754	50.89	2	736,8716	1 471,7287	2	-0,002182	-1,482	1 759,96	49 756,0
(L)WGKVNVDEVGGEAL(G)	99 %	LG	70.27	52.528236	48.55	2	736,8699	1 471,7251	2	-0,005722	-3,885	1 816,22	52 752,0
(L)WGKVNVDEVGGEAL(G)	99 %	LG	63.15	48.38484	41.96	2	736,8761	1 471,7376	2	0,006778	4,602	2 363,92	14 350,0
(L)WGKVNVDEVGGEAL(G)	99 %	LG	61.79	48.463802	44.2	2	736,8694	1 471,7242	2	-0,006642	-4,51	2 186,39	15 774,0
(L)WGKVNVDEVGGEAL(G)	99 %	LG	59.9	48.467884	45.46	2	736,8728	1 471,7310	2	0,0001381	0,09374	2 258,72	12 820,0
(L)WGKVNVDEVGGEAL(G)	98 %	LG	59.09	48.421722	45.97	2	1 472,7330	1 471,7257	1	-0,005166	-3,508	1 791,44	6 822,00
(L)WGKVNVDEVGGEAL(G)	98 %	LG	57.79	48.42216	39.45	2	1 472,7326	1 471,7253	1	-0,005526	-3,752	1 792,61	7 402,00
(L)WGKVNVDEVGGEAL(G)	98 %	LG	56.76	48.58639	44.24	2	736,8723	1 471,7301	2	-0,0007819	-0,5309	2 159,90	18 650,0
(L)WGKVNVDEVGGEAL(G)	97 %	LG	55.91	48.56003	36.51	2	736,8755	1 471,7364	2	0,005578	3,788	2 230,64	11 928,0
(L)WGKVNVDEVGGEAL(G)	97 %	LG	55.54	48.457367	38.66	2	736,8693	1 471,7239	2	-0,006922	-4,7	2 338,61	14 955,0
(L)WGKVNVDEVGGEAL(G)	97 %	LG	54.67	48.51845	33.02	2	736,8733	1 471,7321	2	0,001258	0,8542	2 289,94	14 561,0
(L)WGKVNVDEVGGEAL(G)	97 %	LG	54.3	48.422348	34.08	2	736,8699	1 471,7253	2	-0,005582	-3,79	1 946,80	23 344,0
(L)WGKVNVDEVGGEAL(G)	94 %	LG	50.2	48.350754	28.63	2	736,8716	1 471,7287	2	-0,002182	-1,482	2 226,16	11 605,0
(L)WGKVNVDEVGGEAL(G)	94 %	LG	50.07	48.34224	35.13	2	736,8719	1 471,7291	2	-0,001722	-1,169	1 793,78	21 919,0
(L)WGKVNVDEVGGEALGRL(L)	100 %	LL	150.71	48.436813	135.16	2	899,9804	1 797,9463	2	0,008698	4,835	1 879,03	737581
(L)WGKVNVDEVGGEALGRL(L)	100 %	LL	133.78	48.547672	117.16	2	899,9773	1 797,9400	2	0,002478	1,378	1 862,70	879302
(L)WGKVNVDEVGGEALGRL(L)	100 %	LL	123.97	48.711933	109.33	2	899,9755	1 797,9365	2	-0,001102	-0,6125	1 900,02	125476
(L)WGKVNVDEVGGEALGRL(L)	100 %	LL	103.87	48.74064	87.84	2	899,9761	1 797,9377	2	0,0001181	0,06563	1 903,51	108725
(L)WGKVNVDEVGGEALGRL(L)	100 %	LL	98.44	48.741745	81.32	2	600,3177	1 797,9313	3	-0,006298	-3,501	1 868,54	127778
(L)WGKVNVDEVGGEALGRL(L)	100 %	LL	97.43	48.5179	83.42	2	899,975	1 797,9354	2	-0,002202	-1,224	2 125,65	121128
(L)WGKVNVDEVGGEALGRL(L)	100 %	LL	96.22	48.77337	82.87	2	899,9722	1 797,9299	2	-0,007702	-4,281	2 005,27	37 206,0

(L)WGKVNVDEVGGEALGRL(L)	100 %	LL	95.56	48.605774	79.4	2	899,9776	1 797,9407	2	0,003098	1,722	1 952,48	72 107,0
(L)WGKVNVDEVGGEALGRL(L)	100 %	LL	90.3	48.72471	78.73	2	899,9742	1 797,9338	2	-0,003762	-2,091	1 979,36	66 071,0
(L)WGKVNVDEVGGEALGRL(L)	100 %	LL	85.02	48.609962	72.02	2	899,9766	1 797,9386	2	0,001018	0,5659	1 955,99	77 471,0
(L)WGKVNVDEVGGEALGRL(L)	100 %	LL	78.81	48.70246	69.57	2	899,9737	1 797,9329	2	-0,004642	-2,58	2 030,79	50 938,0
(L)WGKVNVDEVGGEALGRL(L)	100 %	LL	76.8	48.77216	65.69	2	899,9724	1 797,9303	2	-0,007302	-4,059	1 999,25	74 339,0
(L)WGKVNVDEVGGEALGRL(L)	100 %	LL	75.88	48.73809	59.38	2	600,3198	1 797,9375	3	-0,0001179	-0,06556	1 868,62	78 392,0
(L)WGKVNVDEVGGEALGRL(L)	100 %	LL	75.81	48.98352	63.31	2	899,9651	1 797,9156	2	-0,02198	-12,22	2 100,79	142213
(L)WGKVNVDEVGGEALGRL(L)	100 %	LL	72.41	48.610264	55.0	2	899,9764	1 797,9382	2	0,0006581	0,3658	1 925,73	84 816,0
(L)WGKVNVDEVGGEALGRL(L)	99 %	LL	66.74	48.695602	50.22	2	600,3196	1 797,9368	3	-0,0007479	-0,4158	2 096,21	18 389,0
(L)WGKVNVDEVGGEALGRL(L)	99 %	LL	65.76	48.71958	53.23	2	899,9746	1 797,9345	2	-0,003022	-1,68	1 931,48	51 324,0
(L)WGKVNVDEVGGEALGRL(L)	99 %	LL	65.01	48.71573	51.66	2	899,9743	1 797,9341	2	-0,003462	-1,924	1 980,79	67 409,0
(L)WGKVNVDEVGGEALGRL(L)	98 %	LL	58.5	48.547916	45.5	2	600,3206	1 797,9401	3	0,002522	1,402	2 102,05	32 517,0
(L)WGKVNVDEVGGEALGRL(L)	97 %	LL	56.04	48.675148	43.39	2	899,9717	1 797,9288	2	-0,008742	-4,86	2 006,35	49 167,0
(L)WGKVNVDEVGGEALGRL(L)	96 %	LL	54.02	48.711468	38.33	2	899,9755	1 797,9365	2	-0,001082	-0,6014	2 034,55	48 747,0
(L)WGKVNVDEVGGEALGRL(L)	96 %	LL	53.04	48.725872	43.42	2	899,9738	1 797,9331	2	-0,004442	-2,469	2 059,97	36 141,0
(L)WGKVNVDEVGGEALGRL(L)	94 %	LL	50.83	48.55598	42.9	2	600,3206	1 797,9399	3	0,002282	1,269	2 077,71	47 944,0
(L)WGKVNVDEVGGEALGRL(L)	92 %	LL	48.68	48.72599	33.0	2	600,3184	1 797,9332	3	-0,004348	-2,417	1 864,04	49 186,0
(L)WGKVNVDEVGGEALGRLL(V)	100 %	LV	152.32	48.03006	135.84	2	956,5182	1 911,0219	2	0,0002181	0,114	2 085,63	890289
(L)WGKVNVDEVGGEALGRLL(V)	100 %	LV	178.28	48.064514	162.38	2	956,5185	1 911,0224	2	0,0007181	0,3755	2 079,87	359367
(L)WGKVNVDEVGGEALGRLL(V)	100 %	LV	152.78	48.074196	139.07	2	956,5186	1 911,0226	2	0,0008981	0,4697	2 079,79	321136
(L)WGKVNVDEVGGEALGRLL(V)	100 %	LV	129.06	47.89482	109.74	2	638,0157	1 911,0251	3	0,003452	1,805	2 087,97	539276
(L)WGKVNVDEVGGEALGRLL(V)	100 %	LV	128.34	48.050316	109.58	2	638,0132	1 911,0178	3	-0,003868	-2,023	2 079,79	332483
(L)WGKVNVDEVGGEALGRLL(V)	100 %	LV	124.95	48.048138	113.5	2	638,0148	1 911,0225	3	0,0008421	0,4404	2 079,79	228351
(L)WGKVNVDEVGGEALGRLL(V)	100 %	LV	118.85	48.392075	105.51	2	638,0114	1 911,0123	3	-0,009358	-4,894	2 037,89	118403
(L)WGKVNVDEVGGEALGRLL(V)	100 %	LV	112.27	48.30255	99.14	2	638,0127	1 911,0163	3	-0,005398	-2,823	2 085,63	210246
(L)WGKVNVDEVGGEALGRLL(V)	100 %	LV	107.68	48.05664	92.22	2	956,5181	1 911,0215	2	-0,0001219	-0,06378	2 037,80	135674
(L)WGKVNVDEVGGEALGRLL(V)	100 %	LV	106.72	48.00545	92.02	2	956,5194	1 911,0242	2	0,002538	1,327	2 116,01	325656
(L)WGKVNVDEVGGEALGRLL(V)	100 %	LV	65.88	47.428978	53.62	2	956,5255	1 911,0363	2	0,01468	7,677	2 115,08	113056
(L)WGKVNVDEVGGEALGRLL(V)	97 %	LV	55.08	48.290462	48.0	2	638,0129	1 911,0169	3	-0,004798	-2,509	2 116,01	29 897,0
(L)WGKVNVDEVGGEALGRLL(V)	95 %	LV	56.18	53.64296	44.66	2	956,5175	1 911,0204	2	-0,001262	-0,66	2 097,29	40 991,0
(L)WGKVNVDEVGGEALGRLL(V)	92 %	LV	48.0	48.165794	44.0	2	638,0134	1 911,0183	3	-0,003358	-1,756	2 087,97	12 818,0
(L)WGKVNVDEVGGEALGRLL(V)	92 %	LV	47.81	48.19386	42.49	2	638,0139	1 911,0199	3	-0,001738	-0,909	2 105,96	12 379,0
(K)VNVDEVGGEALGRL(L)	100 %	LL	87.14	47.98146	62.12	2	714,377	1 426,7394	2	-0,002382	-1,668	2 123,21	68 125,0
(K)VNVDEVGGEALGRL(L)	100 %	LL	86.41	48.050724	64.33	2	714,3771	1 426,7396	2	-0,002162	-1,514	1 873,21	369202
(K)VNVDEVGGEALGRL(L)	100 %	LL	75.46	48.09115	53.2	2	714,3731	1 426,7316	2	-0,01012	-7,089	1 870,87	93 119,0
(D)EVGGEALGRL(L)	99 %	LL	65.52	50.77459	35.72	2	500,7722	999,5298	2	-0,005262	-5,259	2 329,39	8 987,00
(D)EVGGEALGRL(L)	99 %	LL	58.1	46.741165	28.11	2	500,7725	999,5304	2	-0,004702	-4,699	2 259,63	9 735,00
(D)EVGGEALGRL(L)	99 %	LL	58.6	47.376934	28.69	2	500,773	999,5315	2	-0,003542	-3,54	2 247,92	3 433,00
(D)EVGGEALGRL(L)	98 %	LL	56.17	47.033943	31.36	2	500,7738	999,5331	2	-0,001962	-1,961	2 357,86	9 718,00
(D)EVGGEALGRL(L)	98 %	LL	55.67	47.379875	26.62	2	500,7733	999,532	2	-0,003042	-3,04	1 512,17	90 828,0
(D)EVGGEALGRL(L)	98 %	LL	58.93	50.762474	32.01	2	500,7737	999,5328	2	-0,002242	-2,241	1 493,53	82 878,0
(D)EVGGEALGRL(L)	98 %	LL	54.6	46.741165	27.13	2	500,7726	999,5305	2	-0,004522	-4,52	1 517,99	366715
(D)EVGGEALGRL(L)	97 %	LL	54.66	47.348	29.91	2	500,7729	999,5313	2	-0,003802	-3,8	1 492,37	152504
(D)EVGGEALGRL(L)	97 %	LL	53.17	47.033943	30.41	2	500,7739	999,5332	2	-0,001822	-1,821	1 542,59	310400
(D)EVGGEALGRL(L)	97 %	LL	52.64	47.08625	25.83	2	500,7734	999,5323	2	-0,002802	-2,8	1 491,29	69 314,0
(D)EVGGEALGRL(L)	97 %	LL	52.53	47.033943	30.48	2	500,7738	999,533	2	-0,002022	-2,021	1 542,43	1 043,00
(D)EVGGEALGRL(L)	96 %	LL	51.33	47.34776	36.54	2	500,7729	999,5312	2	-0,003902	-3,9	2 385,76	10 187,0

(D)EVGGEALGRL(L)	96 %	LL	54.65	50.79785	29.77	2		500,7736	999,5327	2	-0,002342	-2,341	1 542,51	828491
(D)EVGGEALGRL(L)	95 %	LL	50.73	47.348	26.79	2		500,7729	999,5313	2	-0,003782	-3,78	2 257,56	12 375,0
(D)EVGGEALGRL(L)	92 %	LL	50.64	50.74044	26.35	2		500,7742	999,5338	2	-0,001302	-1,301	1 549,66	56 675,0
(D)EVGGEALGRL(L)	91 %	LL	46.57	47.271423	19.16	2		500,7755	999,5363	2	0,001278	1,277	2 254,06	4 272,00
(D)EVGGEALGRL(L)	91 %	LL	46.04	47.08625	11.78	2		500,7734	999,5323	2	-0,002802	-2,8	2 284,04	5 585,00
(L)GRLLVVYPWTQRF(F)	100 %	FF	72.13	46.065964	63.28	2		817,9623	1 633,9100	2	0,0006581	0,4025	2 042,48	597118
(L)GRLLVVYPWTQRF(F)	99 %	FF	62.06	45.756264	45.08	2		817,9643	1 633,9140	2	0,004658	2,849	2 043,64	566237
(F)FESFGDLSTPDAVMGNPKVK(A)	98 %	KA	57.98	49.30618	48.66	2		1 070,0263	2 138,0380	2	0,002378	1,112	1 860,45	47 463,0
(F)FESFGDLSTPDAVmGNPKVK(A)	91 %	KA	47.32	48.7436	44.94	2	Oxidation (+16)	1 078,0207	2 154,0269	2	-0,003677	-1,706	1 735,27	57 584,0
(F)FESFGDLSTPDAVmGNPKVKAHGKKVL(G)	98 %	LG	58.51	50.186424	53.75	2	Oxidation (+16)	722,8801	2 887,4912	4	0,000631	0,2185	1 545,92	34 043,0
(F)FESFGDLSTPDAVMGNPKVKAHGKKVL(G)	92 %	LG	49.74	50.15867	49.74	2		718,8785	2 871,4849	4	-0,01081	-3,765	1 695,58	34 924,0
(V)LGAFSDGLAHLDNL(K)	99 %	LK	60.64	48.322468	38.21	2		721,8664	1 441,7181	2	-0,002222	-1,54	2 108,97	43 722,0
(V)LGAFSDGLAHLDNL(K)	98 %	LK	56.15	48.449615	37.69	2		721,869	1 441,7234	2	0,003018	2,092	2 107,96	50 064,0
(V)LGAFSDGLAHLDNLKGTF(A)	100 %	FA	69.34	49.32697	56.1	2		938,4844	1 874,9541	2	0,001178	0,628	2 078,71	40 757,0
(V)LGAFSDGLAHLDNLKGTF(A)	99 %	FA	61.07	49.36916	48.25	2		625,9896	1 874,9469	3	-0,006028	-3,213	2 078,62	25 584,0
(V)LGAFSDGLAHLDNLKGTF(A)	97 %	FA	55.33	49.300365	38.28	2		625,9905	1 874,9496	3	-0,003418	-1,822	2 078,62	36 822,0
(G)LAHLDNLKGTF(A)	100 %	FA	84.28	47.408363	69.97	2		614,8365	1 227,6584	2	-0,002942	-2,394	1 636,92	247925
(G)LAHLDNLKGTF(A)	100 %	FA	74.42	47.497902	48.86	2		614,8358	1 227,6571	2	-0,004282	-3,485	1 638,09	80 477,0
(L)AHLDNLKGTF(A)	99 %	FA	62.43	47.92658	44.05	2		558,2947	1 114,5748	2	-0,002442	-2,189	1 786,77	58 043,0
(L)AHLDNLKGTF(A)	99 %	FA	61.03	47.984295	26.18	2		558,2955	1 114,5765	2	-0,0007819	-0,7009	1 302,45	173946
(L)AHLDNLKGTF(A)	98 %	FA	58.87	47.97669	23.42	2		558,2959	1 114,5772	2	-0,0001019	-0,09138	1 304,78	600485
(L)AHLDNLKGTF(A)	98 %	FA	58.16	47.984295	24.69	2		558,2956	1 114,5766	2	-0,0006819	-0,6113	1 715,44	74 013,0
(L)AHLDNLKGTF(A)	96 %	FA	51.86	47.9044	18.0	2		558,2941	1 114,5737	2	-0,003582	-3,211	1 638,09	42 420,0
(L)AHLDNLKGTF(A)	95 %	FA	50.95	48.05786	18.37	2		558,2949	1 114,5752	2	-0,002102	-1,884	2 018,11	28 939,0
(A)HLDNLKGTF(A)	99 %	FA	60.7	48.151794	47.03	2		522,777	1 043,5394	2	-0,0007419	-0,7103	1 982,97	21 434,0
(A)HLDNLKGTF(A)	98 %	FA	56.66	48.09984	37.74	2		522,7757	1 043,5369	2	-0,003262	-3,123	1 637,00	61 251,0
(A)HLDNLKGTF(A)	97 %	FA	54.7	48.20116	30.23	2		522,7773	1 043,5400	2	-0,0001219	-0,1167	1 715,36	57 414,0
(A)HLDNLKGTF(A)	96 %	FA	52.98	47.978077	31.45	2		522,7764	1 043,5382	2	-0,002002	-1,917	1 787,94	37 881,0
(A)HLDNLKGTF(A)	96 %	FA	52.34	48.000774	37.8	2		522,7767	1 043,5388	2	-0,001322	-1,266	1 309,44	70 484,0
(A)HLDNLKGTF(A)	96 %	FA	52.28	48.09856	33.8	2		522,7756	1 043,5366	2	-0,003522	-3,372	1 231,47	76 556,0
(A)HLDNLKGTF(A)	96 %	FA	51.86	48.005386	37.56	2		522,7766	1 043,5385	2	-0,001622	-1,553	1 230,22	105311
(A)HLDNLKGTF(A)	95 %	FA	50.98	48.20116	24.16	2		522,7771	1 043,5397	2	-0,0004419	-0,4231	1 716,53	69 861,0
(A)HLDNLKGTF(A)	93 %	FA	49.1	48.20116	30.01	2		522,7773	1 043,5401	2	-0,00004194	-0,04015	1 304,78	65 894,0
(A)HLDNLKGTF(A)	92 %	FA	48.32	48.170757	26.84	2		522,7771	1 043,5396	2	-0,0005819	-0,5571	1 787,02	42 092,0
(A)HLDNLKGTF(A)	92 %	FA	51.55	51.724773	32.15	2		522,7764	1 043,5383	2	-0,001842	-1,763	1 303,78	63 645,0
(A)TLSELHCDKLHVDPENF(R)	97 %	FR	54.92	48.74093	45.81	2		998,9855	1 995,9564	2	0,02016	10,09	1 721,21	133419
(A)TLSELHCDKLHVDPENF(R)	91 %	FR	47.29	48.209435	39.0	2		998,9751	1 995,9356	2	-0,0006619	-0,3315	1 814,79	53 274,0
(L)SELHCDKLHVDPENF(R)	100 %	FR	101.29	46.540993	86.79	2		891,9082	1 781,8018	2	-0,002662	-1,493	1 694,33	213911
(L)SELHCDKLHVDPENF(R)	100 %	FR	100.5	46.55705	85.46	2		891,9072	1 781,7999	2	-0,004602	-2,581	1 692,08	96 823,0
(L)SELHCDKLHVDPENF(R)	100 %	FR	96.01	46.540993	84.15	2		891,9082	1 781,8018	2	-0,002662	-1,493	1 769,23	116127
(L)SELHCDKLHVDPENF(R)	100 %	FR	93.84	46.887135	81.87	2		891,9121	1 781,8095	2	0,005078	2,848	1 694,33	658925
(L)SELHCDKLHVDPENF(R)	100 %	FR	91.3	46.61576	75.17	2		891,9075	1 781,8004	2	-0,004102	-2,301	1 691,99	238209
(L)SELHCDKLHVDPENF(R)	97 %	FR	53.28	46.77689	43.51	2		891,9095	1 781,8044	2	-0,00002194	-0,01231	1 768,05	30 701,0
(L)SELHCDKLHVDPENF(R)	93 %	FR	47.58	46.606667	38.81	2		891,9075	1 781,8005	2	-0,003982	-2,234	1 767,39	46 991,0
(C)DKLHVDPENFRL(L)	100 %	LL	69.68	47.960953	22.89	2		741,8893	1 481,7640	2	0,001138	0,7675	1 761,04	125822
(C)DKLHVDPENFRL(L)	99 %	LL	60.15	48.29027	40.63	2		494,9264	1 481,7572	3	-0,005648	-3,809	1 761,04	70 401,0

(C)DKLHVDPENFRL(L)	97 %	LL	53.91	48.106693	21.98	2	741,887	1 481,7594	2	-0,003442	-2,321	1 805,44	46 001,0
(C)DKLHVDPENFRL(L)	95 %	LL	50.66	47.986435	23.98	2	741,8878	1 481,7611	2	-0,001782	-1,202	1 810,12	263270
(N)FRLLGNVL(V)	94 %	LV	43.96	41.671104	24.46	2	466,2895	930,5643	2	-0,0008219	-0,8823	2 087,97	246689
(N)FRLLGNVL(V)	92 %	LV	43.0	42.977165	24.93	2	466,2892	930,5637	2	-0,001422	-1,526	2 083,30	24 285,0
(C)VLAHHFGKEFTPPVQAA(Y)	100 %	AY	127.86	52.42586	114.67	2	924,9944	1 847,9743	2	0,005958	3,222	1 418,98	1036970
(C)VLAHHFGKEFTPPVQAA(Y)	100 %	AY	115.23	48.72442	99.93	2	924,9911	1 847,9677	2	-0,0006819	-0,3688	1 425,98	657049
(C)VLAHHFGKEFTPPVQAA(Y)	100 %	AY	115.06	48.54737	102.03	2	924,9954	1 847,9762	2	0,007818	4,228	1 416,64	1016490
(C)VLAHHFGKEFTPPVQAA(Y)	100 %	AY	114.55	48.77268	102.89	2	924,9905	1 847,9664	2	-0,001922	-1,039	1 409,72	266687
(C)VLAHHFGKEFTPPVQAA(Y)	100 %	AY	104.11	48.726166	90.62	2	924,9912	1 847,9678	2	-0,0005219	-0,2823	1 437,64	167865
(C)VLAHHFGKEFTPPVQAA(Y)	100 %	AY	90.14	48.726166	83.01	2	924,9913	1 847,9680	2	-0,0003219	-0,1741	1 424,82	710688
(C)VLAHHFGKEFTPPVQAA(Y)	100 %	AY	89.67	48.84456	78.97	2	924,9897	1 847,9647	2	-0,003622	-1,959	1 659,30	43 128,0
(C)VLAHHFGKEFTPPVQAA(Y)	100 %	AY	76.46	48.726166	65.3	2	924,9913	1 847,9680	2	-0,0003419	-0,1849	1 423,65	131492
(C)VLAHHFGKEFTPPVQAA(Y)	100 %	AY	76.41	48.79222	63.37	2	924,9901	1 847,9656	2	-0,002802	-1,515	1 450,45	78 177,0
(C)VLAHHFGKEFTPPVQAA(Y)	100 %	AY	67.33	48.771698	56.04	2	924,9906	1 847,9667	2	-0,001642	-0,888	1 815,97	67 691,0
(C)VLAHHFGKEFTPPVQAA(Y)	96 %	AY	53.48	48.719173	46.11	2	924,991	1 847,9673	2	-0,001022	-0,5527	1 406,23	15 397,0
(C)VLAHHFGKEFTPPVQAA(Y)	91 %	AY	47.68	48.746445	28.18	2	924,9916	1 847,9687	2	0,0003381	0,1828	1 407,39	45 646,0
(V)LAHHFGKEFTPPVQAA(Y)	100 %	AY	120.97	48.834457	108.02	2	875,4602	1 748,9058	2	0,005838	3,336	1 336,25	1002530
(V)LAHHFGKEFTPPVQAA(Y)	100 %	AY	112.99	54.698868	99.31	2	875,4585	1 748,9024	2	0,002458	1,405	1 336,34	446746
(V)LAHHFGKEFTPPVQAA(Y)	100 %	AY	110.25	54.785202	95.8	2	875,4567	1 748,8989	2	-0,001042	-0,5954	1 332,75	103053
(V)LAHHFGKEFTPPVQAA(Y)	100 %	AY	96.25	49.015293	82.31	2	875,4563	1 748,8981	2	-0,001862	-1,064	1 331,67	294691
(V)LAHHFGKEFTPPVQAA(Y)	100 %	AY	93.63	48.822227	72.53	2	875,4598	1 748,9051	2	0,005138	2,936	1 337,42	386257
(V)LAHHFGKEFTPPVQAA(Y)	100 %	AY	84.42	54.78425	72.03	2	875,4565	1 748,8984	2	-0,001522	-0,8697	1 331,76	165108
(V)LAHHFGKEFTPPVQAA(Y)	100 %	AY	73.59	54.63116	64.48	2	875,4588	1 748,9030	2	0,003038	1,736	1 328,59	72 393,0
(V)LAHHFGKEFTPPVQAA(Y)	97 %	AY	56.37	48.89839	47.73	2	875,4571	1 748,8996	2	-0,0003619	-0,2068	1 326,93	50 444,0
(V)LAHHFGKEFTPPVQAA(Y)	93 %	AY	49.35	48.825813	45.0	2	875,4582	1 748,9018	2	0,001858	1,062	1 335,25	33 562,0
(Q)AAYQKVVAGVANALAHKY(-)	100 %		137.85	47.581474	115.01	2	937,5162	1 873,0178	2	-0,003122	-1,666	1 775,10	173162
(Q)AAYQKVVAGVANALAHKY(-)	100 %		100.79	47.676086	85.74	2	625,3449	1 873,0128	3	-0,008158	-4,353	1 775,19	113426
(Q)AAYQKVVAGVANALAHKY(-)	100 %		88.69	47.774124	76.72	2	937,515	1 873,0155	2	-0,005462	-2,915	1 774,18	119439
(A)YQKVVAGVAN(A)	100 %	NA	67.62	47.101765	45.73	2	524,7924	1 047,5701	2	-0,001122	-1,07	865,668	98 644,0
(A)YQKVVAGVAN(A)	98 %	NA	54.92	47.048363	30.62	2	524,7935	1 047,5724	2	0,001118	1,066	863,34	124704
(A)YQKVVAGVAN(A)	95 %	NA	50.1	47.178535	27.76	2	524,7926	1 047,5706	2	-0,0006219	-0,5931	859,847	33 335,0
(A)YQKVVAGVAN(A)	94 %	NA	49.32	47.17887	26.41	2	524,7926	1 047,5705	2	-0,0007219	-0,6885	796,962	32 072,0
(A)YQKVVAGVAN(A)	93 %	NA	48.03	47.252663	26.85	2	1 048,5755	1 047,5682	1	-0,003036	-2,895	869,49	6 558,00
(A)YQKVVAGVANA(L)	100 %	AL	75.72	47.14271	59.96	2	560,3118	1 118,6089	2	0,0005781	0,5163	999,671	87 524,0
(A)YQKVVAGVANA(L)	100 %	AL	69.3	47.27045	47.54	2	560,3106	1 118,6066	2	-0,001782	-1,592	975,226	132917
(A)YQKVVAGVANA(L)	98 %	AL	57.89	47.27045	43.22	2	560,3106	1 118,6066	2	-0,001762	-1,574	974,146	29 558,0

C1 cysteine peptidases

Alpha-subunit

(V)LSPADKTNVK(A)	100 %	KA	63.52	47.557606	38.61	2	536,8025	1 071,5904	2	-0,002202	-2,053	597,768	41 767,0
(V)LSPADKTNVK(A)	100 %	KA	59.96	47.36914	30.94	2	536,8057	1 071,5968	2	0,004258	3,97	655,006	145453
(V)LSPADKTNVK(A)	100 %	KA	58.39	47.33358	32.26	2	536,8049	1 071,5953	2	0,002758	2,571	658,423	137459
(V)LSPADKTNVK(A)	99 %	KA	55.21	47.138348	29.16	2	536,8047	1 071,5949	2	0,002358	2,198	700,435	162335
(V)LSPADKTNVK(A)	99 %	KA	55.0	47.498287	26.0	2	536,8031	1 071,5916	2	-0,0009219	-0,8595	577,58	46 356,0
(V)LSPADKTNVK(A)	99 %	KA	54.66	49.808937	31.46	2	536,8047	1 071,5948	2	0,002258	2,105	669,192	53 287,0
(V)LSPADKTNVK(A)	97 %	KA	47.17	47.495895	15.25	2	536,8035	1 071,5924	2	-0,0001419	-0,1323	675,942	101602
(V)LSPADKTNVK(A)	94 %	KA	43.56	47.500916	21.86	2	536,8041	1 071,5937	2	0,001158	1,08	630,491	77 960,0

(V)LSPADKTNVK(A)	91 %	KA	40.81	47.249146	14.25	2	536,8027	1 071,5908	2	-0,001762	-1,643	687,766	20 260,0
(V)LSPADKTNVKA(A)	99 %	AA	56.17	47.224533	35.78	2	572,3206	1 142,6265	2	-0,003122	-2,73	790,263	186364
(V)LSPADKTNVKA(A)	99 %	AA	54.52	47.259033	38.87	2	572,3211	1 142,6276	2	-0,002042	-1,785	814,772	176319
(V)LSPADKTNVKA(A)	96 %	AA	46.12	47.3016	28.44	2	572,322	1 142,6295	2	-0,0001819	-0,1591	765,758	90 886,0
(V)LSPADKTNVKAAWGK(V)	100 %	KV	107.41	47.5557	94.44	2	529,2933	1 584,8582	3	-0,004378	-2,761	1 148,23	47 563,0
(V)LSPADKTNVKAAWGK(V)	100 %	KV	91.81	47.3658	74.33	2	793,4389	1 584,8633	2	0,0006981	0,4402	1 147,98	373610
(V)LSPADKTNVKAAWGK(V)	100 %	KV	83.78	47.537437	67.05	2	529,2938	1 584,8596	3	-0,002968	-1,871	1 166,70	138588
(V)LSPADKTNVKAAWGK(V)	100 %	KV	76.81	47.512558	61.79	2	529,2942	1 584,8608	3	-0,001798	-1,134	1 023,36	114793
(V)LSPADKTNVKAAWGK(V)	100 %	KV	76.67	47.381382	62.05	2	793,4381	1 584,8617	2	-0,0009019	-0,5687	1 021,11	67 511,0
(V)LSPADKTNVKAAWGK(V)	100 %	KV	80.79	52.24352	61.42	2	793,4401	1 584,8657	2	0,003158	1,991	1 153,81	293087
(V)LSPADKTNVKAAWGK(V)	100 %	KV	76.39	52.591278	60.42	2	793,4376	1 584,8606	2	-0,001962	-1,237	1 149,15	102716
(V)LSPADKTNVKAAWGK(V)	100 %	KV	70.87	47.879276	53.68	2	793,4336	1 584,8526	2	-0,009922	-6,257	1 146,15	122560
(V)LSPADKTNVKAAWGK(V)	100 %	KV	69.34	47.5795	55.93	2	529,2931	1 584,8574	3	-0,005158	-3,252	1 021,02	97 433,0
(V)LSPADKTNVKAAWGK(V)	100 %	KV	63.04	47.3658	48.56	2	793,4389	1 584,8632	2	0,0006381	0,4023	1 146,82	92 434,0
(V)LSPADKTNVKAAWGK(V)	99 %	KV	57.15	47.512558	41.78	2	529,2942	1 584,8609	3	-0,001708	-1,077	1 674,87	23 780,0
(V)LSPADKTNVKAAWGK(V)	99 %	KV	57.08	47.547073	46.78	2	529,2934	1 584,8584	3	-0,004198	-2,647	1 147,32	32 859,0
(V)LSPADKTNVKAAWGK(V)	99 %	KV	52.65	47.83003	33.64	2	529,2912	1 584,8518	3	-0,0108	-6,809	1 020,03	54 276,0
(V)LSPADKTNVKAAWGK(V)	97 %	KV	47.44	47.386696	35.33	2	793,4396	1 584,8647	2	0,002118	1,336	1 669,05	93 543,0
(V)LSPADKTNVKAAWGKVG(A)	100 %	GA	93.65	47.038757	72.77	2	871,4842	1 740,9538	2	0,001358	0,7796	1 417,17	419425
(V)LSPADKTNVKAAWGKVG(A)	100 %	GA	92.04	47.38606	71.23	2	871,4831	1 740,9517	2	-0,0008019	-0,4604	1 392,62	284568
(V)LSPADKTNVKAAWGKVG(A)	100 %	GA	79.85	47.47404	69.77	2	871,4815	1 740,9484	2	-0,004042	-2,32	1 776,67	55 945,0
(V)LSPADKTNVKAAWGKVG(A)	100 %	GA	73.8	47.460968	59.56	2	581,3233	1 740,9480	3	-0,004518	-2,594	1 292,35	306717
(V)LSPADKTNVKAAWGKVG(A)	100 %	GA	78.04	52.283516	64.06	2	871,483	1 740,9515	2	-0,0009619	-0,5522	1 422,91	194067
(V)LSPADKTNVKAAWGKVG(A)	100 %	GA	71.9	47.553333	57.83	2	871,4817	1 740,9487	2	-0,003722	-2,137	1 777,58	88 175,0
(V)LSPADKTNVKAAWGKVG(A)	100 %	GA	68.46	47.298046	49.17	2	871,4823	1 740,9500	2	-0,002502	-1,436	1 290,10	76 202,0
(V)LSPADKTNVKAAWGKVG(A)	100 %	GA	66.82	47.25119	53.95	2	871,4827	1 740,9509	2	-0,001582	-0,9081	1 293,51	211898
(V)LSPADKTNVKAAWGKVG(A)	97 %	GA	48.26	46.99482	36.63	2	871,4866	1 740,9587	2	0,006258	3,593	1 391,46	92 113,0
(V)LSPADKTNVKAAWGKVGAHA(G)	100 %	AG	66.71	52.664032	53.11	2	674,3714	2 020,0924	3	0,006802	3,366	1 348,51	129481
(V)LSPADKTNVKAAWGKVGAHA(G)	100 %	AG	59.7	48.136078	53.15	2	506,0279	2 020,0824	4	-0,003154	-1,561	1 349,76	27 051,0
(V)LSPADKTNVKAAWGKVGAHAG(E)	100 %	GE	117.83	47.941532	102.6	2	693,3797	2 077,1173	3	0,01026	4,938	1 314,49	240430
(V)LSPADKTNVKAAWGKVGAHAG(E)	100 %	GE	67.74	48.80362	53.47	2	520,2803	2 077,0921	4	-0,01497	-7,206	1 313,83	24 992,0
(V)LSPADKTNVKAAWGKVGAHAG(E)	99 %	GE	57.49	47.978077	41.7	2	693,3795	2 077,1168	3	0,009722	4,678	1 313,33	68 671,0
(V)LSPADKTNVKAAWGKVGAHAG(E)	98 %	GE	51.76	48.078262	42.12	2	693,3788	2 077,1147	3	0,007592	3,653	1 315,99	36 456,0
(V)LSPADKTNVKAAWGKVGAHAGE(Y)	100 %	EY	114.51	48.975224	99.46	2	736,3936	2 206,1590	3	0,009362	4,242	1 344,84	274584
(V)LSPADKTNVKAAWGKVGAHAGE(Y)	100 %	EY	96.25	49.15009	81.76	2	736,3913	2 206,1521	3	0,002462	1,115	1 225,08	57 851,0
(V)LSPADKTNVKAAWGKVGAHAGE(Y)	100 %	EY	81.19	49.130558	70.39	2	736,3915	2 206,1526	3	0,002972	1,347	1 225,99	75 171,0
(V)LSPADKTNVKAAWGKVGAHAGE(Y)	100 %	EY	78.1	48.959915	67.65	2	736,394	2 206,1602	3	0,01053	4,772	1 332,07	136417
(V)LSPADKTNVKAAWGKVGAHAGE(Y)	100 %	EY	73.61	49.302406	71.3	2	552,5436	2 206,1455	4	-0,004214	-1,909	1 342,51	45 366,0
(V)LSPADKTNVKAAWGKVGAHAGE(Y)	100 %	EY	64.79	49.130558	50.7	2	736,3915	2 206,1526	3	0,002882	1,306	1 224,91	65 134,0
(L)SPADKTNVK(A)	100 %	KA	72.19	48.36267	45.96	2	480,2605	958,5065	2	-0,001982	-2,066	686,516	54 546,0
(L)SPADKTNVK(A)	100 %	KA	63.45	48.380173	33.01	2	480,2607	958,5068	2	-0,001702	-1,774	660,76	536342
(L)SPADKTNVK(A)	100 %	KA	61.54	48.37973	31.33	2	480,2609	958,5072	2	-0,001262	-1,315	645,581	40 565,0
(L)SPADKTNVK(A)	100 %	KA	59.62	48.37973	31.58	2	480,261	958,5075	2	-0,0009819	-1,023	556,602	30 983,0
(L)SPADKTNVK(A)	99 %	KA	58.88	48.37973	32.15	2	480,2609	958,5072	2	-0,001222	-1,274	661,929	71 663,0
(L)SPADKTNVK(A)	99 %	KA	56.65	48.439735	33.4	2	480,26	958,5055	2	-0,002942	-3,066	685,267	462394
(L)SPADKTNVK(A)	99 %	KA	56.07	48.25277	23.68	2	480,2618	958,509	2	0,0004981	0,5191	691,107	123233

(L)SPADKTNVK(A)	99 %	KA	55.71	48.15817	34.46	2	480,2596	958,5046	2	-0,003822	-3,983	808,942	47 156,0
(L)SPADKTNVK(A)	99 %	KA	54.67	48.37973	24.13	2	480,261	958,5074	2	-0,001062	-1,107	628,072	120222
(L)SPADKTNVK(A)	99 %	KA	54.47	48.37973	26.77	2	480,2609	958,5072	2	-0,001242	-1,294	644,412	283783
(L)SPADKTNVK(A)	99 %	KA	52.79	48.387863	22.07	2	480,2612	958,5079	2	-0,0005619	-0,5857	596,598	138105
(L)SPADKTNVK(A)	99 %	KA	52.53	48.15817	18.91	2	480,2596	958,5047	2	-0,003742	-3,9	621,07	50 471,0
(L)SPADKTNVK(A)	98 %	KA	51.95	48.37973	19.31	2	480,2609	958,5072	2	-0,001302	-1,357	630,408	22 452,0
(L)SPADKTNVK(A)	97 %	KA	47.83	48.414783	24.67	2	480,2601	958,5056	2	-0,002842	-2,962	764,926	24 076,0
(L)SPADKTNVK(A)	95 %	KA	45.87	48.37973	21.28	2	480,2608	958,507	2	-0,001462	-1,524	575,248	68 769,0
(L)SPADKTNVKA(A)	100 %	AA	61.89	48.388744	41.04	2	515,7804	1 029,5462	2	0,0006381	0,6191	812,438	698167
(L)SPADKTNVKA(A)	100 %	AA	61.79	48.289112	34.37	2	515,7782	1 029,5419	2	-0,003642	-3,534	763,431	207051
(L)SPADKTNVKA(A)	100 %	AA	60.36	48.14161	42.13	2	515,7815	1 029,5484	2	0,002818	2,735	761,603	58 878,0
(L)SPADKTNVKA(A)	99 %	AA	53.68	48.01671	26.73	2	515,7791	1 029,5437	2	-0,001842	-1,787	762,267	57 989,0
(L)SPADKTNVKA(A)	98 %	AA	51.12	48.289112	32.38	2	515,7781	1 029,5417	2	-0,003882	-3,767	787,93	561160
(L)SPADKTNVKA(A)	96 %	AA	46.5	48.289112	25.51	2	515,7783	1 029,5421	2	-0,003482	-3,379	519,381	21 666,0
(L)SPADKTNVKA(A)	93 %	AA	43.6	48.29722	15.96	2	515,7786	1 029,5425	2	-0,003022	-2,932	837,013	45 394,0
(L)SPADKTNVKAAWG(K)	100 %	GK	88.17	48.292717	64.55	2	672,8473	1 343,6801	2	-0,003362	-2,5	1 407,77	110014
(L)SPADKTNVKAAWG(K)	100 %	GK	85.51	48.28821	62.51	2	672,8482	1 343,6818	2	-0,001642	-1,221	1 406,60	365384
(L)SPADKTNVKAAWG(K)	100 %	GK	73.31	48.349167	57.56	2	672,8475	1 343,6805	2	-0,002962	-2,203	1 406,69	133070
(L)SPADKTNVKAAWG(K)	100 %	GK	61.53	48.199978	37.24	2	672,849	1 343,6834	2	-0,00002194	-0,01632	1 404,27	110076
(L)SPADKTNVKAAWG(K)	99 %	GK	58.99	48.307747	35.23	2	672,8482	1 343,6819	2	-0,001562	-1,162	1 406,60	37 485,0
(L)SPADKTNVKAAWG(K)	99 %	GK	58.4	48.19958	40.56	2	672,8492	1 343,6838	2	0,0003381	0,2514	1 148,15	38 132,0
(L)SPADKTNVKAAWG(K)	99 %	GK	52.89	48.467945	33.29	2	672,8505	1 343,6864	2	0,002938	2,185	975,873	35 247,0
(L)SPADKTNVKAAWG(K)	92 %	GK	43.15	48.278408	23.34	2	672,8484	1 343,6823	2	-0,001202	-0,8938	1 161,96	18 115,0
(L)SPADKTNVKAAWGK(V)	100 %	KV	117.28	48.118027	88.18	2	736,897	1 471,7795	2	0,001038	0,7048	790,263	169291
(L)SPADKTNVKAAWGK(V)	100 %	KV	109.46	48.112938	87.97	2	736,8973	1 471,7800	2	0,001578	1,071	1 146,82	447355
(L)SPADKTNVKAAWGK(V)	100 %	KV	105.9	48.2713	76.98	2	736,8981	1 471,7817	2	0,003218	2,185	1 149,15	943274
(L)SPADKTNVKAAWGK(V)	100 %	KV	100.0	48.112396	83.44	2	491,6009	1 471,7808	3	0,002282	1,549	1 153,81	313018
(L)SPADKTNVKAAWGK(V)	100 %	KV	93.22	48.21022	76.59	2	491,5988	1 471,7746	3	-0,003838	-2,606	789,096	141503
(L)SPADKTNVKAAWGK(V)	100 %	KV	92.11	48.100643	71.6	2	491,5996	1 471,7769	3	-0,001528	-1,037	1 146,82	61 458,0
(L)SPADKTNVKAAWGK(V)	100 %	KV	92.06	48.111862	74.61	2	491,5992	1 471,7757	3	-0,002788	-1,893	1 150,31	135619
(L)SPADKTNVKAAWGK(V)	100 %	KV	80.92	48.391953	61.83	2	491,5982	1 471,7727	3	-0,005788	-3,93	1 146,90	45 717,0
(L)SPADKTNVKAAWGK(V)	100 %	KV	80.15	48.227238	57.55	2	736,8966	1 471,7787	2	0,0002381	0,1616	793,768	53 422,0
(L)SPADKTNVKAAWGK(V)	100 %	KV	73.4	48.13954	57.29	2	491,5991	1 471,7754	3	-0,003118	-2,117	1 147,98	215242
(L)SPADKTNVKAAWGK(V)	100 %	KV	72.13	48.1193	53.96	2	736,8971	1 471,7797	2	0,001218	0,827	1 168,94	225481
(L)SPADKTNVKAAWGK(V)	100 %	KV	64.76	48.1193	50.35	2	736,8971	1 471,7796	2	0,001178	0,7999	1 667,97	68 120,0
(L)SPADKTNVKAAWGK(V)	99 %	KV	59.09	48.391953	43.02	2	491,5982	1 471,7727	3	-0,005788	-3,93	774,006	39 674,0
(L)SPADKTNVKAAWGK(V)	94 %	KV	44.45	48.380867	18.85	2	491,5983	1 471,7732	3	-0,005278	-3,584	1 147,98	15 564,0
(L)SPADKTNVKAAWGK(V)	92 %	KV	42.85	48.105015	32.09	2	491,5994	1 471,7764	3	-0,002038	-1,384	1 669,38	14 737,0
(L)SPADKTNVKAAWGK(V)	92 %	KV	42.58	48.111862	30.62	2	491,5992	1 471,7759	3	-0,002578	-1,75	1 670,30	17 553,0
(L)SPADKTNVKAAWGKVGAHA(G)	100 %	AG	76.42	48.647247	75.13	2	477,7577	1 907,0019	4	0,0003861	0,2023	1 347,17	29 101,0
(L)SPADKTNVKAAWGKVGAHA(G)	100 %	AG	67.67	48.79113	51.65	2	636,6729	1 906,9968	3	-0,004718	-2,473	1 347,17	241837
(L)SPADKTNVKAAWGKVGAHA(G)	100 %	AG	61.67	48.780617	54.99	2	477,757	1 906,9989	4	-0,002614	-1,37	1 351,84	101232
(L)SPADKTNVKAAWGKVGAHAG(E)	100 %	GE	140.97	48.789295	121.64	2	655,6835	1 964,0286	3	0,005582	2,841	1 314,57	1563680
(L)SPADKTNVKAAWGKVGAHAG(E)	100 %	GE	117.68	48.708656	104.72	2	655,6841	1 964,0306	3	0,007592	3,864	1 313,33	317294
(L)SPADKTNVKAAWGKVGAHAG(E)	100 %	GE	116.04	48.702637	95.37	2	655,6833	1 964,0280	3	0,005042	2,566	1 315,74	319163
(L)SPADKTNVKAAWGKVGAHAG(E)	100 %	GE	101.91	49.10843	91.26	2	655,6789	1 964,0150	3	-0,007978	-4,06	1 314,49	101483

(L)SPADKTNVKAAWGKVGAHAG(E)	100 %	GE	79.31	48.997223	61.37	2	492,0113	1 964,0163	4	-0,006714	-3,417	1 314,49	48 448,0
(L)SPADKTNVKAAWGKVGAHAG(E)	100 %	GE	75.88	48.498245	63.6	2	655,6872	1 964,0398	3	0,01683	8,566	1 312,49	346485
(L)SPADKTNVKAAWGKVGAHAG(E)	100 %	GE	74.34	49.01905	71.34	2	492,0119	1 964,0183	4	-0,004634	-2,358	1 314,49	154646
(L)SPADKTNVKAAWGKVGAHAG(E)	100 %	GE	64.56	48.983414	62.58	2	492,0115	1 964,0169	4	-0,006034	-3,071	1 313,33	98 467,0
(L)SPADKTNVKAAWGKVGAHAG(E)	99 %	GE	58.31	49.056934	46.71	2	655,6803	1 964,0191	3	-0,003898	-1,984	1 313,66	75 100,0
(L)SPADKTNVKAAWGKVGAHAG(E)	99 %	GE	55.96	48.83571	43.14	2	983,0198	1 964,0250	2	0,002038	1,037	1 313,49	51 969,0
(L)SPADKTNVKAAWGKVGAHAG(E)	96 %	GE	48.15	48.856384	34.87	2	983,0192	1 964,0238	2	0,0008381	0,4265	1 314,57	54 134,0
(L)SPADKTNVKAAWGKVGAHAGE(Y)	100 %	EY	143.56	49.45242	128.51	2	698,6973	2 093,0701	3	0,004532	2,164	1 353,00	374021
(L)SPADKTNVKAAWGKVGAHAGE(Y)	100 %	EY	137.12	49.42856	122.1	2	698,6975	2 093,0706	3	0,005072	2,422	1 332,07	816788
(L)SPADKTNVKAAWGKVGAHAGE(Y)	100 %	EY	140.94	49.319557	128.25	2	698,6991	2 093,0755	3	0,009932	4,743	1 333,16	846629
(L)SPADKTNVKAAWGKVGAHAGE(Y)	100 %	EY	137.31	55.302498	125.8	2	1 047,5434	2 093,0722	2	0,006678	3,189	1 337,92	372457
(L)SPADKTNVKAAWGKVGAHAGE(Y)	100 %	EY	123.45	49.313766	108.92	2	1 047,5438	2 093,0730	2	0,007458	3,561	1 333,16	519672
(L)SPADKTNVKAAWGKVGAHAGE(Y)	100 %	EY	121.54	49.442062	107.51	2	524,2739	2 093,0663	4	0,0007661	0,3658	1 333,16	425645
(L)SPADKTNVKAAWGKVGAHAGE(Y)	100 %	EY	123.87	55.353474	111.45	2	1 047,5423	2 093,0701	2	0,004558	2,177	1 337,83	483132
(L)SPADKTNVKAAWGKVGAHAGE(Y)	100 %	EY	109.31	49.50569	96.94	2	524,271	2 093,0549	4	-0,01067	-5,097	1 331,99	248822
(L)SPADKTNVKAAWGKVGAHAGE(Y)	100 %	EY	107.98	55.316795	98.24	2	1 047,5432	2 093,0718	2	0,006238	2,979	1 332,49	215899
(L)SPADKTNVKAAWGKVGAHAGE(Y)	100 %	EY	99.8	49.432026	92.25	2	698,6976	2 093,0711	3	0,005492	2,623	1 044,33	112278
(L)SPADKTNVKAAWGKVGAHAGE(Y)	100 %	EY	88.92	49.42137	71.54	2	698,6959	2 093,0660	3	0,0004221	0,2015	1 333,24	72 202,0
(L)SPADKTNVKAAWGKVGAHAGE(Y)	100 %	EY	81.32	49.449657	70.42	2	524,2729	2 093,0624	4	-0,003154	-1,506	1 044,33	105592
(L)SPADKTNVKAAWGKVGAHAGE(Y)	100 %	EY	77.54	49.42018	61.32	2	698,6977	2 093,0712	3	0,005582	2,666	1 358,82	55 580,0
(L)SPADKTNVKAAWGKVGAHAGE(Y)	100 %	EY	77.45	49.380993	65.68	2	1 047,5429	2 093,0713	2	0,005698	2,721	1 332,07	234375
(L)SPADKTNVKAAWGKVGAHAGE(Y)	99 %	EY	66.09	55.443695	60.54	2	1 047,5386	2 093,0626	2	-0,002942	-1,405	1 334,33	98 758,0
(L)SPADKTNVKAAWGKVGAHAGE(Y)	98 %	EY	52.19	49.58416	47.57	2	524,2714	2 093,0564	4	-0,009154	-4,371	1 357,66	26 958,0
(L)SPADKTNVKAAWGKVGAHAGE(Y)	93 %	EY	45.1	49.442604	33.83	2	698,6957	2 093,0651	3	-0,0004479	-0,2139	1 049,23	65 710,0
(S)PADKTNVK(A)	99 %	KA	59.14	47.74495	30.12	2	436,7463	871,4781	2	0,001658	1,9	687,599	159891
(S)PADKTNVK(A)	99 %	KA	53.19	47.77318	27.51	2	436,745	871,4755	2	-0,0009419	-1,08	660,76	126529
(S)PADKTNVK(A)	99 %	KA	52.93	47.93602	30.21	2	436,7449	871,4753	2	-0,001142	-1,309	694,604	218250
(S)PADKTNVK(A)	98 %	KA	49.07	47.00998	29.03	2	436,744	871,4734	2	-0,003042	-3,487	688,768	39 959,0
(S)PADKTNVK(A)	95 %	KA	47.89	50.32679	29.13	2	436,7441	871,4736	2	-0,002902	-3,326	624,735	51 146,0
(S)PADKTNVK(A)	94 %	KA	43.66	47.00998	19.01	2	436,7438	871,4731	2	-0,003342	-3,83	668,942	36 263,0
(S)PADKTNVK(A)	91 %	KA	40.82	47.108112	23.52	2	436,747	871,4794	2	0,002978	3,413	638,579	20 147,0
(S)PADKTNVKA(A)	99 %	AA	57.37	47.249557	32.38	2	472,2642	942,5137	2	0,0001781	0,1887	812,522	158965
(S)PADKTNVKA(A)	99 %	AA	54.89	47.39074	30.1	2	472,2631	942,5116	2	-0,001922	-2,037	812,438	250562
(S)PADKTNVKA(A)	98 %	AA	49.47	46.967407	31.68	2	472,262	942,5095	2	-0,004082	-4,326	763,431	52 242,0
(S)PADKTNVKA(A)	96 %	AA	46.09	47.406548	18.84	2	472,2633	942,5121	2	-0,001442	-1,528	787,93	117724
(S)PADKTNVKAAWG(K)	100 %	GK	78.54	47.71514	53.47	2	629,3324	1 256,6503	2	-0,001142	-0,908	1 152,64	76 652,0
(S)PADKTNVKAAWG(K)	97 %	GK	48.01	47.90046	33.44	2	629,3314	1 256,6482	2	-0,003262	-2,594	1 406,69	373784
(S)PADKTNVKAAWG(K)	97 %	GK	53.43	53.783997	34.08	2	629,3312	1 256,6479	2	-0,003542	-2,816	1 405,52	110651
(S)PADKTNVKAAWG(K)	94 %	GK	50.25	53.68921	26.79	2	629,3318	1 256,6491	2	-0,002362	-1,878	1 153,97	40 561,0
(S)PADKTNVKAAWGK(V)	100 %	KV	101.86	47.29877	77.85	2	693,3835	1 384,7524	2	0,005898	4,256	1 147,98	746378
(S)PADKTNVKAAWGK(V)	100 %	KV	101.8	52.684002	84.79	2	693,3808	1 384,7469	2	0,0004781	0,345	1 149,23	162716
(S)PADKTNVKAAWGK(V)	100 %	KV	100.7	52.310593	79.79	2	693,3838	1 384,7531	2	0,006638	4,79	1 158,47	796978
(S)PADKTNVKAAWGK(V)	100 %	KV	95.91	47.606712	75.46	2	693,3807	1 384,7469	2	0,0004381	0,3161	1 146,90	382213
(S)PADKTNVKAAWGK(V)	100 %	KV	86.03	52.764297	61.82	2	693,3797	1 384,7449	2	-0,001562	-1,127	1 147,32	182000
(S)PADKTNVKAAWGK(V)	100 %	KV	80.3	47.485832	63.27	2	462,5888	1 384,7446	3	-0,001868	-1,348	1 148,06	95 373,0
(S)PADKTNVKAAWGK(V)	100 %	KV	74.43	47.475437	58.68	2	462,5885	1 384,7438	3	-0,002708	-1,954	1 146,98	54 240,0

(S)PADKTNVKAAWGK(V)	100 %	KV	66.29	47.463074	52.45	2	462,5883	1 384,7430	3	-0,003488	-2,517	1 022,19	36 118,0
(S)PADKTNVKAAWGK(V)	100 %	KV	64.0	47.301765	52.51	2	462,5891	1 384,7456	3	-0,0008779	-0,6335	1 151,47	55 087,0
(S)PADKTNVKAAWGK(V)	99 %	KV	57.09	47.574265	34.53	2	693,3794	1 384,7443	2	-0,002142	-1,546	1 165,45	46 634,0
(S)PADKTNVKAAWGK(V)	98 %	KV	50.49	47.525787	36.35	2	462,5879	1 384,7419	3	-0,004568	-3,296	1 021,02	15 931,0
(S)PADKTNVKAAWGK(V)	97 %	KV	48.52	47.606712	33.03	2	693,3808	1 384,7471	2	0,0006581	0,4749	1 021,11	38 613,0
(S)PADKTNVKAAWGKVGA(H)	100 %	AH	62.64	47.508015	55.17	2	806,9403	1 611,8659	2	-0,007522	-4,664	1 477,73	89 501,0
(S)PADKTNVKAAWGKVGA(H)	95 %	AH	44.19	47.44731	37.84	2	806,9434	1 611,8721	2	-0,001322	-0,8196	1 480,15	22 412,0
(S)PADKTNVKAAWGKVGA(H)	91 %	AH	41.87	47.756542	33.1	2	538,2951	1 611,8636	3	-0,009878	-6,124	1 479,40	21 769,0
(S)PADKTNVKAAWGKVGAHA(G)	100 %	AK	103.22	48.297607	82.88	2	607,6626	1 819,9658	3	-0,003648	-2,003	1 349,51	249866
(S)PADKTNVKAAWGKVGAHA(G)	99 %	AK	53.04	48.193466	45.57	2	607,6633	1 819,9682	3	-0,001308	-0,7183	1 346,09	72 019,0
(S)PADKTNVKAAWGKVGAHAG(E)	100 %	GE	106.14	48.557373	94.39	2	626,6713	1 876,9921	3	0,001102	0,5868	1 313,41	160689
(S)PADKTNVKAAWGKVGAHAG(E)	100 %	GE	102.48	48.568436	89.69	2	939,5018	1 876,9891	2	-0,001902	-1,013	1 314,49	68 930,0
(S)PADKTNVKAAWGKVGAHAG(E)	100 %	GE	94.67	48.14667	82.54	2	626,674	1 877,0003	3	0,009292	4,948	1 313,83	865124
(S)PADKTNVKAAWGKVGAHAG(E)	100 %	GE	93.95	48.358936	78.66	2	626,6723	1 876,9951	3	0,004132	2,2	1 316,83	269773
(S)PADKTNVKAAWGKVGAHAG(E)	100 %	GE	87.23	48.357666	73.4	2	626,6723	1 876,9951	3	0,004102	2,184	1 315,66	109817
(S)PADKTNVKAAWGKVGAHAG(E)	100 %	GE	70.63	48.44769	52.14	2	470,2548	1 876,9901	4	-0,0008739	-0,4654	1 313,66	71 072,0
(S)PADKTNVKAAWGKVGAHAG(E)	100 %	GE	64.39	48.524494	51.23	2	470,2547	1 876,9897	4	-0,001234	-0,6571	1 314,57	63 137,0
(S)PADKTNVKAAWGKVGAHAG(E)	99 %	GE	57.78	48.557373	49.4	2	626,6713	1 876,9922	3	0,001222	0,6507	1 340,25	57 393,0
(S)PADKTNVKAAWGKVGAHAGE(Y)	100 %	EY	130.65	49.052616	119.15	2	669,6867	2 006,0382	3	0,004642	2,313	1 332,16	755893
(S)PADKTNVKAAWGKVGAHAGE(Y)	100 %	EY	134.61	49.060764	128.62	2	669,6878	2 006,0416	3	0,008002	3,987	1 333,24	843312
(S)PADKTNVKAAWGKVGAHAGE(Y)	100 %	EY	116.18	49.20562	103.41	2	1 004,0274	2 006,0402	2	0,006618	3,297	1 333,16	366784
(S)PADKTNVKAAWGKVGAHAGE(Y)	100 %	EY	108.64	49.29562	104.22	2	502,5154	2 006,0324	4	-0,001154	-0,5749	1 333,16	197181
(S)PADKTNVKAAWGKVGAHAGE(Y)	100 %	EY	105.25	49.227673	94.85	2	1 004,0252	2 006,0358	2	0,002278	1,135	1 332,07	137946
(S)PADKTNVKAAWGKVGAHAGE(Y)	100 %	EY	102.57	49.14052	96.3	2	669,6857	2 006,0353	3	0,001732	0,863	1 337,92	66 907,0
(S)PADKTNVKAAWGKVGAHAGE(Y)	100 %	EY	102.56	49.172535	99.51	2	669,6886	2 006,0440	3	0,01046	5,213	1 349,51	114039
(S)PADKTNVKAAWGKVGAHAGE(Y)	100 %	EY	99.21	49.46644	91.75	2	502,5108	2 006,0140	4	-0,01955	-9,743	1 332,49	192867
(S)PADKTNVKAAWGKVGAHAGE(Y)	100 %	EY	96.89	49.22045	91.63	2	502,515	2 006,0307	4	-0,002874	-1,432	1 340,17	285574
(S)PADKTNVKAAWGKVGAHAGE(Y)	100 %	EY	85.12	49.106937	77.14	2	1 004,0265	2 006,0385	2	0,004898	2,44	1 333,24	90 239,0
(S)PADKTNVKAAWGKVGAHAGE(Y)	100 %	EY	62.59	49.118923	62.2	2	669,6858	2 006,0356	3	0,002002	0,9975	1 701,72	64 213,0
(S)PADKTNVKAAWGKVGAHAGE(Y)	97 %	EY	50.21	49.25441	42.55	2	502,5142	2 006,0275	4	-0,006074	-3,026	1 332,16	29 020,0
(S)PADKTNVKAAWGKVGAHAGE(Y)	95 %	EY	46.18	49.359856	41.49	2	502,5146	2 006,0291	4	-0,004434	-2,209	1 341,51	47 317,0
(P)ADKTNVKAAWGK(V)	100 %	KV	92.1	47.78137	69.9	2	644,8533	1 287,6920	2	-0,001622	-1,259	1 147,98	301723
(P)ADKTNVKAAWGK(V)	100 %	KV	74.2	48.105217	48.19	2	430,2374	1 287,6903	3	-0,003358	-2,606	1 153,81	20 935,0
(P)ADKTNVKAAWGK(V)	100 %	KV	72.49	47.882812	46.95	2	644,8539	1 287,6932	2	-0,0004219	-0,3274	1 146,82	82 624,0
(P)ADKTNVKAAWGK(V)	100 %	KV	68.9	48.028217	52.02	2	644,8518	1 287,6889	2	-0,004722	-3,664	1 673,71	32 868,0
(P)ADKTNVKAAWGK(V)	99 %	KV	54.01	48.408646	39.18	2	644,8454	1 287,6763	2	-0,0174	-13,5	788,43	46 359,0
(P)ADKTNVKAAWGK(V)	93 %	KV	43.56	47.88303	31.1	2	644,854	1 287,6935	2	-0,0002019	-0,1567	1 166,95	10 499,0
(P)ADKTNVKAAWGKVG(A)	100 %	GA	113.27	47.668438	88.09	2	722,8973	1 443,7800	2	-0,003582	-2,479	1 417,17	260959
(P)ADKTNVKAAWGKVG(A)	100 %	GA	97.75	47.854862	81.11	2	722,8969	1 443,7791	2	-0,004422	-3,061	1 401,95	233112
(P)ADKTNVKAAWGKVG(A)	100 %	GA	95.47	47.724907	74.93	2	722,8985	1 443,7824	2	-0,001202	-0,8319	1 426,40	371663
(P)ADKTNVKAAWGKVG(A)	100 %	GA	95.33	47.72417	76.64	2	722,8975	1 443,7805	2	-0,003042	-2,105	1 432,22	245800
(P)ADKTNVKAAWGKVG(A)	100 %	GA	89.15	47.72505	71.81	2	722,8978	1 443,7811	2	-0,002442	-1,69	1 392,71	181604
(P)ADKTNVKAAWGKVG(A)	100 %	GA	82.62	50.724777	65.0	2	722,9	1 443,7854	2	0,001878	1,3	1 415,92	58 683,0
(P)ADKTNVKAAWGKVG(A)	100 %	GA	74.14	47.724907	54.13	2	722,8985	1 443,7825	2	-0,001082	-0,7489	1 392,79	234896
(P)ADKTNVKAAWGKVG(A)	99 %	GA	56.76	47.638172	40.73	2	722,8973	1 443,7801	2	-0,003442	-2,382	1 392,87	55 794,0
(P)ADKTNVKAAWGKVG(A)	99 %	GA	56.56	47.723145	36.69	2	722,898	1 443,7815	2	-0,002042	-1,413	1 427,56	73 241,0

(P)ADKTNVKAAWGKVGAHAGE(Y)	100 %	EY	122.33	49.03757	111.21	2	637,3347	1 908,9822	3	0,001472	0,7707	1 337,83	105160
(P)ADKTNVKAAWGKVGAHAGE(Y)	100 %	EY	97.93	48.978027	80.81	2	637,3336	1 908,9791	3	-0,001708	-0,8942	1 333,49	67 078,0
(P)ADKTNVKAAWGKVGAHAGE(Y)	100 %	EY	86.43	48.951626	74.35	2	955,4957	1 908,9768	2	-0,003942	-2,064	1 333,16	79 057,0
(P)ADKTNVKAAWGKVGAHAGE(Y)	100 %	EY	80.69	49.089462	68.6	2	955,4949	1 908,9751	2	-0,005622	-2,943	1 334,33	73 513,0
(P)ADKTNVKAAWGKVGAHAGE(Y)	100 %	EY	83.32	53.89237	71.92	2	637,3358	1 908,9855	3	0,004742	2,483	1 338,08	102046
(P)ADKTNVKAAWGKVGAHAGE(Y)	100 %	EY	82.56	53.944817	76.28	2	637,3336	1 908,9789	3	-0,001858	-0,9728	1 343,84	114582
(P)ADKTNVKAAWGKVGAHAGE(Y)	100 %	EY	74.62	48.984726	61.04	2	637,3336	1 908,9789	3	-0,001828	-0,957	1 333,16	96 297,0
(P)ADKTNVKAAWGKVGAHAGE(Y)	100 %	EY	66.32	49.01175	59.08	2	955,4951	1 908,9756	2	-0,005142	-2,692	1 333,24	68 975,0
(P)ADKTNVKAAWGKVGAHAGE(Y)	91 %	EY	43.04	48.920723	38.24	2	637,3351	1 908,9834	3	0,002642	1,383	1 335,50	37 150,0
(A)DKTNVKAAWGK(V)	100 %	KV	92.53	47.795387	60.08	2	609,3339	1 216,6533	2	-0,003282	-2,695	1 147,98	129371
(A)DKTNVKAAWGK(V)	100 %	KV	82.26	47.772312	49.16	2	609,3348	1 216,6551	2	-0,001442	-1,184	1 165,54	74 677,0
(A)DKTNVKAAWGK(V)	100 %	KV	86.32	52.78662	53.29	2	609,3333	1 216,6520	2	-0,004602	-3,779	1 150,31	182547
(A)DKTNVKAAWGK(V)	100 %	KV	77.52	52.909737	42.82	2	609,3321	1 216,6497	2	-0,006842	-5,619	1 148,48	80 338,0
(A)DKTNVKAAWGK(V)	100 %	KV	59.03	47.866096	34.02	2	609,334	1 216,6535	2	-0,003082	-2,531	1 148,15	27 916,0
(A)DKTNVKAAWGK(V)	99 %	KV	57.28	47.77347	29.13	2	609,3341	1 216,6536	2	-0,002942	-2,416	1 146,90	73 385,0
(A)DKTNVKAAWGKVG(A)	100 %	GA	111.49	47.7158	86.31	2	687,3793	1 372,7441	2	-0,002402	-1,748	1 392,79	171242
(A)DKTNVKAAWGKVG(A)	100 %	GA	97.77	47.700047	74.77	2	687,3791	1 372,7435	2	-0,002922	-2,127	1 417,17	223231
(A)DKTNVKAAWGKVGAHAGE(Y)	100 %	EY	114.42	48.86367	95.29	2	919,9772	1 837,9399	2	-0,003782	-2,057	1 333,16	149860
(A)DKTNVKAAWGKVGAHAGE(Y)	100 %	EY	72.95	48.86367	60.77	2	613,6539	1 837,9398	3	-0,003828	-2,082	1 333,41	71 157,0
(A)DKTNVKAAWGKVGAHAGE(Y)	99 %	EY	59.36	48.879547	51.41	2	613,654	1 837,9400	3	-0,003648	-1,984	1 338,33	197474
(D)KTNVKAAWGK(V)	100 %	KV	80.32	45.925873	50.46	2	551,8212	1 101,6278	2	-0,001782	-1,616	1 148,15	83 032,0
(D)KTNVKAAWGK(V)	100 %	KV	67.86	45.925873	39.21	2	551,8211	1 101,6277	2	-0,001862	-1,689	1 147,32	56 784,0
(D)KTNVKAAWGK(V)	93 %	KV	41.54	45.84953	25.19	2	551,8217	1 101,6288	2	-0,0008019	-0,7273	1 150,64	36 697,0
(D)KTNVKAAWGKVGAHAG(E)	100 %	GE	81.76	46.91037	53.91	2	532,2967	1 593,8681	3	-0,005948	-3,729	1 314,57	38 075,0
(D)KTNVKAAWGKVGAHAG(E)	100 %	GE	68.2	46.60353	57.85	2	797,9444	1 593,8743	2	0,0001981	0,1242	1 314,49	45 147,0
(D)KTNVKAAWGKVGAHAG(E)	100 %	GE	63.83	46.784454	47.71	2	797,9426	1 593,8706	2	-0,003462	-2,171	1 313,41	32 788,0
(D)KTNVKAAWGKVGAHAGE(Y)	100 %	EY	110.26	47.863255	92.31	2	862,4645	1 722,9144	2	-0,002222	-1,289	1 332,16	173668
(D)KTNVKAAWGKVGAHAGE(Y)	100 %	EY	100.27	48.02158	89.24	2	862,4636	1 722,9127	2	-0,003982	-2,31	1 333,24	296199
(D)KTNVKAAWGKVGAHAGE(Y)	100 %	EY	90.03	48.261204	72.46	2	862,4615	1 722,9083	2	-0,008322	-4,827	1 332,49	153151
(D)KTNVKAAWGKVGAHAGE(Y)	100 %	EY	83.17	48.005867	69.7	2	575,3114	1 722,9123	3	-0,004418	-2,563	790,43	94 518,0
(D)KTNVKAAWGKVGAHAGE(Y)	99 %	EY	57.96	47.87666	49.29	2	575,3119	1 722,9139	3	-0,002798	-1,623	1 335,50	46 392,0
(D)KTNVKAAWGKVGAHAGE(Y)	99 %	EY	58.41	52.945103	43.9	2	575,3114	1 722,9125	3	-0,004208	-2,441	789,262	118667
(D)KTNVKAAWGKVGAHAGE(Y)	98 %	EY	49.49	47.88981	45.06	2	862,4645	1 722,9145	2	-0,002162	-1,254	1 334,58	20 298,0
(K)TNVKAAWGKVG(A)	100 %	GA	67.77	47.307743	40.98	2	565,8194	1 129,6243	2	-0,0002019	-0,1786	801,941	31 608,0
(K)TNVKAAWGKVG(A)	99 %	GA	57.02	47.396515	33.71	2	565,8185	1 129,6224	2	-0,002062	-1,824	1 412,43	20 744,0
(K)TNVKAAWGKVG(A)	99 %	GA	55.04	46.632957	31.03	2	565,8209	1 129,6273	2	0,002838	2,51	1 018,70	85 794,0
(K)TNVKAAWGKVG(A)	99 %	GA	53.01	47.541267	39.02	2	1 130,6294	1 129,6221	1	-0,002356	-2,084	1 411,60	4 859,00
(K)TNVKAAWGKVGAHAG(E)	100 %	GE	90.21	47.588737	65.4	2	733,8962	1 465,7778	2	-0,001262	-0,8603	922,316	17 736,0
(K)TNVKAAWGKVGAHAG(E)	100 %	GE	59.1	47.629036	49.19	2	489,5991	1 465,7754	3	-0,003718	-2,535	920,987	15 548,0
(K)TNVKAAWGKVGAHAG(E)	97 %	GE	48.65	47.312016	29.09	2	733,899	1 465,7834	2	0,004338	2,958	1 314,66	31 261,0
(K)TNVKAAWGKVGAHAGE(Y)	100 %	EY	94.77	48.40451	67.58	2	798,4163	1 594,8181	2	-0,003562	-2,232	1 333,16	155476
(K)TNVKAAWGKVGAHAGE(Y)	99 %	EY	58.42	48.368805	21.89	2	798,4181	1 594,8217	2	0,00001806	0,01132	1 332,07	77 628,0
(T)NVKAAWGKVG(A)	91 %	GA	44.77	51.26459	18.36	2	515,2953	1 028,5760	2	-0,0007219	-0,7012	954,671	31 730,0
(T)NVKAAWGKVGAHAGE(Y)	100 %	EY	68.56	48.12713	41.68	2	747,8932	1 493,7719	2	-0,002042	-1,366	882,518	43 758,0
(T)NVKAAWGKVGAHAGEYG(A)	100 %	GA	84.22	48.852142	83.21	2	857,9366	1 713,8586	2	-0,0001619	-0,09443	1 461,53	131744
(T)NVKAAWGKVGAHAGEYG(A)	100 %	GA	75.9	48.850727	63.48	2	857,9366	1 713,8587	2	-0,00008194	-0,04778	1 485,98	79 129,0

(T)NVKAAWGKVGAHAGEYG(A)	100 %	GA	64.39	48.793255	49.21	2	857,9371	1 713,8595	2	0,0007781	0,4537	1 070,25	40 873,0
(T)NVKAAWGKVGAHAGEYG(A)	99 %	GA	53.52	48.78246	48.15	2	857,9386	1 713,8626	2	0,003818	2,226	1 071,08	37 208,0
(T)NVKAAWGKVGAHAGEYG(A)	98 %	GA	51.05	48.814816	50.51	2	572,2936	1 713,8590	3	0,0002621	0,1528	1 071,25	81 836,0
(N)VKAAWGKVGAHAGE(Y)	100 %	EY	64.29	47.340157	0.0	2	690,8722	1 379,7298	2	-0,001242	-0,8995	796,439	47 670,0
(N)VKAAWGKVGAHAGE(Y)	100 %	EY	61.78	47.611755	0.0	2	690,8735	1 379,7324	2	0,001358	0,9836	798,443	72 080,0
(N)VKAAWGKVGAHAGE(Y)	92 %	EY	41.64	47.11546	0.0	2	690,8758	1 379,7371	2	0,006018	4,359	1 337,00	65 159,0
(N)VKAAWGKVGAHAGEYG(A)	100 %	GA	116.55	48.661873	0.0	2	800,9168	1 599,8191	2	0,003198	1,998	984,889	467122
(N)VKAAWGKVGAHAGEYG(A)	100 %	GA	100.65	48.637867	0.0	2	800,9178	1 599,8210	2	0,005118	3,197	984,221	133385
(N)VKAAWGKVGAHAGEYG(A)	100 %	GA	95.3	48.664646	0.0	2	800,9164	1 599,8181	2	0,002278	1,423	983,72	132238
(N)VKAAWGKVGAHAGEYG(A)	100 %	GA	88.26	48.7509	0.0	2	800,9143	1 599,8139	2	-0,001922	-1,201	1 487,14	41 016,0
(N)VKAAWGKVGAHAGEYG(A)	100 %	GA	63.42	48.810078	0.0	2	534,2782	1 599,8129	3	-0,002978	-1,86	984,889	147195
(V)KAAWGKVGAHAGEYG(A)	100 %	GA	75.4	48.679977	64.71	2	751,3823	1 500,7500	2	0,002498	1,663	1 489,57	46 240,0
(V)KAAWGKVGAHAGEYG(A)	100 %	GA	72.07	48.583927	63.07	2	751,3845	1 500,7544	2	0,006918	4,607	1 491,75	68 958,0
(V)KAAWGKVGAHAGEYG(A)	99 %	GA	57.56	48.808422	48.94	2	751,3824	1 500,7503	2	0,002858	1,903	1 489,48	73 087,0
(V)KAAWGKVGAHAGEYG(A)	99 %	GA	53.12	48.57863	46.43	2	751,3785	1 500,7424	2	-0,005042	-3,357	1 462,53	59 013,0
(V)KAAWGKVGAHAGEYG(A)	93 %	GA	43.89	48.566986	33.13	2	751,3832	1 500,7518	2	0,004318	2,875	985,14	25 005,0
(V)KAAWGKVGAHAGEYG(A)	92 %	GA	42.77	48.421474	34.89	2	751,3849	1 500,7553	2	0,007858	5,233	1 491,07	54 826,0
(K)AAWGKVGAHA(G)	100 %	AG	72.1	46.317276	56.13	2	484,2597	966,5048	2	0,001218	1,259	822,102	27 111,0
(K)AAWGKVGAHA(G)	100 %	AG	71.09	46.63965	56.2	2	484,2578	966,501	2	-0,002582	-2,669	826,519	36 043,0
(K)AAWGKVGAHAG(E)	100 %	GE	69.25	46.48896	38.43	2	512,7704	1 023,5262	2	0,001178	1,15	746,038	79 230,0
(K)AAWGKVGAHAG(E)	95 %	GE	44.97	47.369617	28.72	2	512,7672	1 023,5198	2	-0,005262	-5,136	744,118	36 675,0
(K)AAWGKVGAHAGE(Y)	100 %	EY	92.92	47.380585	71.43	2	577,2905	1 152,5665	2	-0,001162	-1,007	1 339,00	116184
(K)AAWGKVGAHAGE(Y)	100 %	EY	82.0	47.37312	65.86	2	577,2928	1 152,5711	2	0,003458	2,998	789,096	220072
(K)AAWGKVGAHAGE(Y)	100 %	EY	70.76	47.349197	35.39	2	577,2927	1 152,5708	2	0,003178	2,755	813,607	313811
(K)AAWGKVGAHAGEYG(A)	100 %	GA	96.85	47.619053	76.4	2	687,3318	1 372,6491	2	-0,003402	-2,477	1 461,36	149024
(K)AAWGKVGAHAGEYG(A)	100 %	GA	92.11	47.739326	70.22	2	687,3353	1 372,6561	2	0,003638	2,648	1 032,68	625548
(K)AAWGKVGAHAGEYG(A)	100 %	GA	91.62	47.72439	65.94	2	687,3335	1 372,6525	2	0,00001806	0,01315	1 024,61	93 640,0
(K)AAWGKVGAHAGEYG(A)	100 %	GA	89.79	47.752243	66.61	2	687,3348	1 372,6549	2	0,002478	1,804	1 486,06	206928
(K)AAWGKVGAHAGEYG(A)	100 %	GA	75.38	47.66487	56.64	2	687,3334	1 372,6523	2	-0,0001419	-0,1033	1 038,59	40 099,0
(K)AAWGKVGAHAGEYG(A)	100 %	GA	81.02	53.48947	60.76	2	687,3341	1 372,6537	2	0,001258	0,9158	1 035,01	42 861,0
(K)AAWGKVGAHAGEYG(A)	100 %	GA	78.92	53.632885	53.65	2	687,3372	1 372,6599	2	0,007398	5,386	1 043,16	888247
(K)AAWGKVGAHAGEYG(A)	100 %	GA	72.88	48.041874	47.51	2	687,3378	1 372,6610	2	0,008518	6,201	1 039,67	1196780
(K)AAWGKVGAHAGEYG(A)	100 %	GA	69.06	47.72439	44.94	2	687,3336	1 372,6526	2	0,0001381	0,1005	1 057,12	146189
(K)AAWGKVGAHAGEYG(A)	100 %	GA	60.58	47.9629	39.7	2	687,3338	1 372,6531	2	0,0006181	0,4499	984,889	93 863,0
(K)AAWGKVGAHAGEYG(A)	99 %	GA	55.74	47.75086	32.74	2	687,3349	1 372,6552	2	0,002698	1,964	1 037,59	30 857,0
(K)AAWGKVGAHAGEYG(A)	98 %	GA	50.41	47.7818	12.2	2	687,3344	1 372,6543	2	0,001798	1,309	1 023,44	15 929,0
(K)AAWGKVGAHAGEYG(A)	96 %	GA	45.59	47.63271	30.95	2	687,3325	1 372,6504	2	-0,002022	-1,472	1 037,51	31 180,0
(K)AAWGKVGAHAGEYG(A)	95 %	GA	51.1	53.37445	31.45	2	687,3335	1 372,6525	2	-1,942E-06	-0,001414	1 041,17	45 755,0
(K)AAWGKVGAHAGEYG(A)	91 %	GA	41.46	47.94502	29.4	2	687,3342	1 372,6537	2	0,001278	0,9304	1 114,44	22 356,0
(K)AAWGKVGAHAGEYGA(E)	100 %	AE	132.11	47.964775	100.29	2	722,8521	1 443,6896	2	-1,942E-06	-0,001344	1 110,70	321804
(K)AAWGKVGAHAGEYGA(E)	100 %	AE	93.97	47.96699	67.09	2	722,8518	1 443,6890	2	-0,0005419	-0,3751	1 107,54	61 994,0
(K)AAWGKVGAHAGEYGA(E)	100 %	AE	66.04	47.74765	53.71	2	722,8495	1 443,6844	2	-0,005122	-3,545	1 108,38	56 326,0
(K)AAWGKVGAHAGEYGA(E)	100 %	AE	59.96	47.77492	45.34	2	722,8534	1 443,6922	2	0,002678	1,854	1 527,24	21 648,0
(K)AAWGKVGAHAGEYGAE(A)	100 %	EA	97.9	47.58253	82.19	2	787,3745	1 572,7345	2	0,002318	1,473	1 095,65	148406
(K)AAWGKVGAHAGEYGAE(A)	100 %	EA	81.65	47.724686	71.51	2	787,3767	1 572,7387	2	0,006578	4,18	1 093,24	44 895,0
(K)AAWGKVGAHAGEYGAEALER(M)	100 %	RM	140.02	49.237465	130.83	2	681,6726	2 041,9958	3	-0,001248	-0,6108	1 445,03	380095

(K)AAWGKVGAHAGEYGAEALER(M)	100 %	RM	161.84	49.29066	144.44	2		681,6758	2 042,0055	3	0,008382	4,103	1 448,53	801135
(K)AAWGKVGAHAGEYGAEALER(M)	100 %	RM	128.93	49.300266	114.61	2		681,6739	2 042,0000	3	0,002922	1,43	1 445,03	178015
(K)AAWGKVGAHAGEYGAEALER(M)	100 %	RM	118.51	49.238914	102.93	2		681,6731	2 041,9974	3	0,0003421	0,1674	1 447,36	445484
(K)AAWGKVGAHAGEYGAEALER(M)	100 %	RM	105.79	49.30684	91.38	2		681,6741	2 042,0004	3	0,003282	1,606	1 444,03	72 302,0
(K)AAWGKVGAHAGEYGAEALER(M)	100 %	RM	99.68	49.24574	97.2	2		681,6732	2 041,9978	3	0,0007021	0,3436	1 445,53	64 439,0
(K)AAWGKVGAHAGEYGAEALER(M)	100 %	RM	97.6	49.255596	89.67	2		681,6747	2 042,0022	3	0,005112	2,502	1 443,87	82 767,0
(K)AAWGKVGAHAGEYGAEALER(M)	100 %	RM	95.01	49.25673	81.13	2		1 022,0065	2 041,9984	2	0,001298	0,6354	1 447,36	264555
(K)AAWGKVGAHAGEYGAEALER(M)	100 %	RM	93.79	49.36463	86.73	2		681,6743	2 042,0010	3	0,003912	1,915	1 454,85	47 835,0
(K)AAWGKVGAHAGEYGAEALER(M)	100 %	RM	71.33	49.217335	69.85	2		681,6724	2 041,9953	3	-0,001758	-0,8605	1 449,78	86 392,0
(K)AAWGKVGAHAGEYGAEALER(M)	100 %	RM	70.11	49.253635	55.98	2		1 022,0057	2 041,9969	2	-0,0001619	-0,07927	1 445,03	77 471,0
(K)AAWGKVGAHAGEYGAEALER(M)	99 %	RM	56.78	49.13586	52.39	2		681,672	2 041,9943	3	-0,002808	-1,374	1 446,20	34 096,0
(K)AAWGKVGAHAGEYGAEALER(M)	96 %	RM	47.5	49.29133	38.88	2		681,6744	2 042,0015	3	0,004422	2,164	1 447,45	39 070,0
(A)AWGKVGAHAGE(Y)	100 %	EY	64.33	47.19273	42.25	2		541,7735	1 081,5325	2	0,001898	1,753	705,097	112066
(A)AWGKVGAHAGE(Y)	98 %	EY	48.82	47.220657	29.23	2		541,7733	1 081,5320	2	0,001478	1,365	649,088	56 911,0
(A)AWGKVGAHAGEYG(A)	100 %	GA	92.67	47.79127	59.51	2		651,8158	1 301,6170	2	0,001638	1,258	1 037,34	144084
(A)AWGKVGAHAGEYG(A)	100 %	GA	88.4	47.725563	67.67	2		651,8164	1 301,6183	2	0,002918	2,24	930,136	363205
(A)AWGKVGAHAGEYG(A)	100 %	GA	87.78	47.70314	57.12	2		651,8189	1 301,6231	2	0,007778	5,971	933,632	924575
(A)AWGKVGAHAGEYG(A)	100 %	GA	71.79	47.72762	55.47	2		651,8166	1 301,6186	2	0,003198	2,455	1 034,01	66 430,0
(A)AWGKVGAHAGEYG(A)	100 %	GA	58.68	47.566742	32.1	2		651,8145	1 301,6145	2	-0,0009019	-0,6924	958,079	30 355,0
(A)AWGKVGAHAGEYG(A)	99 %	GA	57.14	47.72762	43.65	2		651,8168	1 301,6190	2	0,003618	2,778	931,801	50 207,0
(A)AWGKVGAHAGEYG(A)	98 %	GA	50.83	47.506546	38.5	2		651,8141	1 301,6137	2	-0,001682	-1,291	1 488,31	29 867,0
(A)AWGKVGAHAGEYGA(E)	100 %	AE	67.08	47.76803	41.1	2		687,3319	1 372,6492	2	-0,003302	-2,404	1 009,38	52 703,0
(A)AWGKVGAHAGEYGA(E)	98 %	AE	57.02	53.29823	39.55	2		687,3331	1 372,6516	2	-0,0009019	-0,6566	1 007,14	44 893,0
(A)AWGKVGAHAGEYGA(E)	96 %	AE	45.67	47.606636	35.56	2		687,3323	1 372,6501	2	-0,002342	-1,705	1 002,90	20 567,0
(A)AWGKVGAHAGEYGAEA(L)	100 %	AL	116.26	52.803574	98.35	2		787,3731	1 572,7317	2	-0,0004419	-0,2808	1 074,58	164034
(A)AWGKVGAHAGEYGAEA(L)	100 %	AL	89.43	52.874588	69.22	2		787,3735	1 572,7324	2	0,0002581	0,164	1 072,25	67 614,0
(A)AWGKVGAHAGEYGAEA(L)	100 %	AL	63.1	47.540344	45.89	2		787,3738	1 572,7330	2	0,0008581	0,5452	1 071,08	50 116,0
(A)WGKVGAHAGEYG(A)	100 %	GA	84.25	47.29845	70.7	2		616,2957	1 230,5769	2	-0,001362	-1,106	1 033,85	238963
(A)WGKVGAHAGEYG(A)	100 %	GA	70.53	47.16137	56.29	2		616,2954	1 230,5762	2	-0,002042	-1,658	1 037,34	561686
(A)WGKVGAHAGEYG(A)	100 %	GA	69.43	47.33318	56.15	2		616,2975	1 230,5803	2	0,002078	1,687	869,631	113432
(A)WGKVGAHAGEYG(A)	100 %	GA	67.54	47.36795	52.12	2		616,2977	1 230,5808	2	0,002498	2,028	934,796	87 649,0
(A)WGKVGAHAGEYG(A)	100 %	GA	62.72	47.28232	45.54	2		616,2966	1 230,5786	2	0,0003381	0,2745	845,1	57 290,0
(A)WGKVGAHAGEYG(A)	98 %	GA	50.94	47.244396	36.85	2		616,2943	1 230,5741	2	-0,004202	-3,412	1 489,48	43 147,0
(A)WGKVGAHAGEYG(A)	93 %	GA	42.62	47.161453	32.06	2		616,2954	1 230,5761	2	-0,002122	-1,723	1 465,12	26 638,0
(W)GKVGAHAGEYG(A)	98 %	GA	49.45	47.349197	38.9	2		523,2558	1 044,4971	2	-0,001842	-1,762	932,551	71 964,0
(W)GKVGAHAGEYGAEALER(M)	100 %	RM	82.65	48.6425	64.89	2		572,2869	1 713,8390	3	-0,004608	-2,687	895,257	37 785,0
(W)GKVGAHAGEYGAEALER(M)	94 %	RM	44.94	48.609547	35.93	2		857,9286	1 713,8427	2	-0,0008619	-0,5026	896,341	81 279,0
(G)KVGAHAGEYGA(E)	100 %	AE	59.13	47.656906	45.26	2		530,2646	1 058,5146	2	0,00001806	0,01704	592,01	31 419,0
(G)KVGAHAGEYGAEALER(M)	100 %	RM	113.95	48.671844	93.86	2		553,2816	1 656,8228	3	0,0007521	0,4536	883,516	56 156,0
(G)KVGAHAGEYGAEALER(M)	100 %	RM	89.4	48.697655	74.1	2		553,2805	1 656,8196	3	-0,002428	-1,465	885,851	245345
(G)KVGAHAGEYGAEALER(M)	100 %	RM	86.49	48.61779	67.59	2		829,4195	1 656,8245	2	0,002398	1,447	885,851	335810
(G)KVGAHAGEYGAEALER(M)	99 %	RM	59.54	54.680195	51.32	2		829,4191	1 656,8236	2	0,001538	0,9278	887,021	211679
(G)KVGAHAGEYGAEALER(M)	97 %	RM	48.14	48.630196	32.31	2		829,4177	1 656,8209	2	-0,001202	-0,725	885,851	62 204,0
(G)AHAGEYGAEALERmFL(S)	100 %	LS	101.06	47.883095	92.01	2	Oxidation (+16)	890,9228	1 779,8311	2	0,006023	3,382	1 918,77	218235
(G)AHAGEYGAEALERmFL(S)	100 %	LS	75.48	47.63615	57.92	2	Oxidation (+16)	594,2824	1 779,8254	3	0,000287	0,1612	1 924,52	45 061,0
(G)AHAGEYGAEALERmFL(S)	98 %	LS	51.81	47.564537	26.63	2	Oxidation (+16)	594,281	1 779,8212	3	-0,003883	-2,18	1 919,85	23 705,0

(G)AEALERmFLSFPTT(K)	100 %	TK	73.44	48.517536	49.25	2	Oxidation (+16)	814,9066	1 627,7987	2	0,007003	4,299	2 059,77	446821
(G)AEALERmFLSFPTT(K)	100 %	TK	68.38	48.719463	43.89	2	Oxidation (+16)	814,9023	1 627,7900	2	-0,001637	-1,005	2 058,61	128279
(G)AEALERmFLSFPTT(K)	100 %	TK	66.29	48.545425	44.27	2	Oxidation (+16)	814,9054	1 627,7961	2	0,004463	2,74	2 063,27	242059
(G)AEALERmFLSFPTT(K)	95 %	TK	45.79	48.624535	24.62	2	Oxidation (+16)	814,9042	1 627,7938	2	0,002103	1,291	2 062,10	38 139,0
(G)AEALERmFLSFPTTK(T)	100 %	KT	71.52	48.728554	56.19	2	Oxidation (+16)	586,3022	1 755,8848	3	-0,001853	-1,055	1 902,34	94 259,0
(G)AEALERMFLSFPTTK(T)	99 %	KT	57.32	48.773136	44.79	2		580,9714	1 739,8923	3	0,0005121	0,2941	2 087,70	23 448,0
(L)ERmFLSFPTT(K)	99 %	TK	57.68	47.24153	20.98	2	Oxidation (+16)	622,8008	1 243,5870	2	-0,003817	-3,067	1 929,19	94 165,0
(L)ERMFLSFPTT(K)	99 %	TK	54.8	47.312424	30.4	2		614,8043	1 227,5941	2	-0,001762	-1,434	2 052,80	59 141,0
(L)ERmFLSFPTT(K)	97 %	TK	48.41	47.5141	18.44	2	Oxidation (+16)	622,8043	1 243,5940	2	0,003263	2,622	1 928,35	95 356,0
(R)mFLSFPTTK(T)	99 %	KT	58.2	47.898834	24.17	2	Oxidation (+16)	544,2772	1 086,5397	2	-0,002337	-2,149	1 873,17	96 515,0
(R)mFLSFPTTK(T)	94 %	KT	43.13	47.29901	17.65	2	Oxidation (+16)	544,2761	1 086,5377	2	-0,004357	-4,006	1 872,01	25 807,0
(R)MFLSFPTTK(T)	92 %	KT	41.63	47.349037	16.29	2		536,281	1 070,5475	2	0,0003181	0,2968	1 988,80	251813
(R)mFLSFPTTKTYFPH(F)	100 %	HF	85.39	48.724884	65.12	2	Oxidation (+16)	866,9262	1 731,8379	2	0,004723	2,726	1 989,97	64 531,0
(R)mFLSFPTTKTYFPH(F)	100 %	HF	78.52	48.64131	50.18	2	Oxidation (+16)	578,2834	1 731,8283	3	-0,004933	-2,847	1 991,05	81 714,0
(R)mFLSFPTTKTYFPH(F)	100 %	HF	76.57	48.830254	57.13	2	Oxidation (+16)	866,9251	1 731,8357	2	0,002483	1,433	1 991,05	138419
(R)MFLSFPTTKTYFPH(F)	100 %	HF	71.85	48.695072	55.54	2		572,9526	1 715,8360	3	-0,002268	-1,321	2 071,41	28 443,0
(R)mFLSFPTTKTYFPH(F)	100 %	HF	68.56	48.499718	51.99	2	Oxidation (+16)	578,2844	1 731,8313	3	-0,001843	-1,064	1 993,39	44 619,0
(R)mFLSFPTTKTYFPH(F)	98 %	HF	51.39	48.6479	34.27	2	Oxidation (+16)	866,9231	1 731,8317	2	-0,001517	-0,8754	1 990,22	33 671,0
(R)mFLSFPTTKTYFPH(F)	97 %	HF	48.4	48.68174	39.1	2	Oxidation (+16)	578,2849	1 731,8328	3	-0,000433	-0,2499	1 991,14	33 623,0
(R)mFLSFPTTKTYFPH(F)	92 %	HF	42.67	48.533634	25.03	2	Oxidation (+16)	578,2835	1 731,8288	3	-0,004423	-2,552	1 989,97	31 221,0
(M)FLSFPTTKTYFPH(F)	100 %	HF	83.76	48.52852	62.35	2		793,406	1 584,7975	2	-0,0002619	-0,1652	1 961,95	45 284,0
(M)FLSFPTTKTYFPH(F)	100 %	HF	77.42	48.52596	54.71	2		529,2733	1 584,7980	3	0,0002721	0,1716	1 961,95	24 402,0
(M)FLSFPTTKTYFPH(F)	99 %	HF	54.56	48.508667	36.05	2		793,4072	1 584,7999	2	0,002098	1,323	1 961,95	28 655,0
(M)FLSFPTTKTYFPH(F)	95 %	HF	45.93	48.569466	14.63	2		793,4079	1 584,8012	2	0,003398	2,143	1 963,04	26 337,0
(L)SFPTTKTYFPH(F)	100 %	HF	60.52	48.074677	45.22	2		663,3355	1 324,6565	2	0,01126	8,492	1 538,40	919350
(L)SFPTTKTYFPH(F)	100 %	HF	60.39	48.0923	41.16	2		663,3317	1 324,6489	2	0,003598	2,714	1 534,90	421129
(L)SFPTTKTYFPH(F)	100 %	HF	59.42	48.124725	44.4	2		663,3306	1 324,6465	2	0,001278	0,9641	1 562,88	88 750,0
(L)SFPTTKTYFPH(F)	99 %	HF	60.06	53.955727	41.88	2		663,3319	1 324,6493	2	0,004038	3,046	1 548,89	843810
(L)SFPTTKTYFPH(F)	98 %	HF	50.28	48.05671	36.81	2		663,332	1 324,6495	2	0,004238	3,197	1 532,82	47 632,0
(L)SFPTTKTYFPH(F)	97 %	HF	55.13	54.012573	41.39	2		663,3332	1 324,6518	2	0,006558	4,947	1 802,31	127192
(S)FPTTKTYFPH(F)	100 %	HF	81.31	47.86858	60.71	2		413,5438	1 237,6097	3	-0,003608	-2,913	1 542,07	69 057,0
(S)FPTTKTYFPH(F)	100 %	HF	76.37	47.86858	54.61	2		413,5438	1 237,6096	3	-0,003668	-2,961	1 541,23	162177
(S)FPTTKTYFPH(F)	100 %	HF	71.66	47.734867	50.47	2		619,8127	1 237,6108	2	-0,002482	-2,004	1 485,98	88 864,0
(S)FPTTKTYFPH(F)	100 %	HF	69.71	48.029514	50.61	2		619,8136	1 237,6127	2	-0,0005419	-0,4375	1 805,55	307141
(S)FPTTKTYFPH(F)	100 %	HF	68.73	48.026848	46.98	2		619,814	1 237,6134	2	0,0001581	0,1276	1 540,73	111909
(S)FPTTKTYFPH(F)	100 %	HF	68.41	48.026028	47.11	2		619,8136	1 237,6126	2	-0,0007019	-0,5667	1 800,89	565192
(S)FPTTKTYFPH(F)	100 %	HF	67.35	47.77514	55.32	2		413,5432	1 237,6077	3	-0,005588	-4,511	1 802,22	14 625,0
(S)FPTTKTYFPH(F)	100 %	HF	63.14	48.003117	46.07	2		619,8151	1 237,6157	2	0,002398	1,936	1 537,23	60 923,0
(S)FPTTKTYFPH(F)	100 %	HF	60.1	47.926716	37.81	2		413,5425	1 237,6057	3	-0,007538	-6,086	1 480,06	10 543,0
(S)FPTTKTYFPH(F)	100 %	HF	59.27	47.865173	36.82	2		413,5439	1 237,6099	3	-0,003398	-2,743	1 485,89	37 908,0
(S)FPTTKTYFPH(F)	99 %	HF	57.18	47.734867	33.94	2		619,8126	1 237,6107	2	-0,002582	-2,085	1 799,73	64 352,0
(S)FPTTKTYFPH(F)	98 %	HF	51.44	47.74057	35.34	2		619,8129	1 237,6112	2	-0,002062	-1,665	1 802,56	61 206,0
(S)FPTTKTYFPH(F)	98 %	HF	51.12	47.852444	30.21	2		619,8116	1 237,6086	2	-0,004702	-3,796	1 825,36	48 804,0
(S)FPTTKTYFPH(F)	98 %	HF	49.42	47.738815	39.15	2		413,5443	1 237,6111	3	-0,002198	-1,775	1 479,23	15 618,0
(S)FPTTKTYFPH(F)	95 %	HF	45.59	47.744366	35.35	2		619,8131	1 237,6116	2	-0,001702	-1,374	1 477,73	31 138,0
(T)TKTYFPHFD(L)	100 %	DL	69.64	46.694565	43.52	2		578,2753	1 154,5360	2	-0,003782	-3,273	1 874,42	32 388,0

(T)TKTYFPHFD(L)	100 %	DL	58.16	46.694565	31.73	2		578,2753	1 154,5360	2	-0,003742	-3,238	1 625,88	73 763,0
(T)TKTYFPHFD(L)	97 %	DL	47.17	46.88767	33.17	2		578,276	1 154,5375	2	-0,002302	-1,992	2 007,72	15 058,0
(T)KTYFPHFDLS(H)	99 %	SH	53.76	48.109047	38.08	2		627,8109	1 253,6072	2	-0,0009219	-0,7348	1 861,51	236086
(T)KTYFPHFDLS(H)	92 %	SH	42.43	47.656242	24.33	2		627,8096	1 253,6047	2	-0,003462	-2,759	1 860,34	56 612,0
(T)KTYFPHFDLS(H)	92 %	SH	41.92	47.511868	23.93	2		627,81	1 253,6055	2	-0,002682	-2,138	1 862,68	120490
(T)KTYFPHFDLSH(G)	100 %	HG	77.35	48.337715	59.55	2		696,3397	1 390,6647	2	-0,002322	-1,668	1 726,22	34 650,0
(T)KTYFPHFDLSH(G)	97 %	HG	49.6	48.427464	38.39	2		464,5618	1 390,6635	3	-0,003588	-2,578	1 726,22	31 292,0
(T)KTYFPHFDLSHGSA(Q)	97 %	AQ	47.86	48.198467	38.69	2		803,887	1 605,7594	2	0,001738	1,082	1 715,80	49 998,0
(T)KTYFPHFDLSHGSA(Q)	94 %	AQ	44.41	48.098625	34.37	2		536,2591	1 605,7553	3	-0,002348	-1,461	1 714,55	30 995,0
(T)KTYFPHFDLSHGSAQ(V)	100 %	QV	83.9	48.12198	68.0	2		867,9153	1 733,8161	2	-0,0001419	-0,08182	1 691,21	107254
(T)KTYFPHFDLSHGSAQ(V)	100 %	QV	60.45	48.08103	47.2	2		867,9157	1 733,8169	2	0,0006381	0,3678	1 690,13	46 676,0
(T)KTYFPHFDLSHGSAQ(V)	96 %	QV	46.21	48.12111	34.37	2		578,9456	1 733,8151	3	-0,001178	-0,679	1 691,21	129823
(T)KTYFPHFDLSHGSAQ(V)	92 %	QV	42.67	47.96859	29.4	2		578,9443	1 733,8109	3	-0,005348	-3,083	1 690,13	46 485,0
(K)TYFPHFDLSHGSAQ(V)	100 %	QV	73.76	46.686096	52.04	2		803,8701	1 605,7256	2	0,004298	2,675	1 818,54	82 957,0
(K)TYFPHFDLSHGSAQ(V)	100 %	QV	70.13	46.958843	34.69	2		803,8723	1 605,7300	2	0,008778	5,463	1 818,87	99 662,0
(K)TYFPHFDLSHGSAQ(V)	99 %	QV	57.43	46.66162	35.57	2		803,8683	1 605,7220	2	0,0007181	0,4469	1 690,29	51 837,0
(Y)FPHFDLSHGSAQ(V)	100 %	QV	65.25	46.579826	43.22	2		671,8131	1 341,6116	2	0,001318	0,9817	1 684,21	120062
(Y)FPHFDLSHGSAQ(V)	99 %	QV	54.03	46.565292	39.31	2		671,8141	1 341,6137	2	0,003418	2,546	1 683,37	78 027,0
(Y)FPHFDLSHGSAQ(V)	94 %	QV	43.2	46.499382	28.98	2		671,8128	1 341,6111	2	0,0007981	0,5944	1 683,12	58 576,0
(H)FDLSHGSAQVKGHGKKVA(D)	100 %	AD	78.72	48.170563	69.85	2		622,6713	1 864,9920	3	0,0009821	0,5263	727,458	110049
(H)FDLSHGSAQVKGHGKKVA(D)	100 %	AD	71.7	47.926296	65.01	2		622,6737	1 864,9992	3	0,008212	4,401	722,612	85 586,0
(D)LSHGSAQVKGHGKKVADAL(T)	97 %	LT	46.3	46.374596	41.11	2		635,0265	1 902,0578	3	0,01402	7,368	644,747	116488
(D)LSHGSAQVKGHGKKVADAL(T)	94 %	LT	43.1	47.072212	37.69	2		635,0228	1 902,0465	3	0,002742	1,441	645,915	63 637,0
(L)SHGSAQVKGHGKKVADALTN(A)	100 %	NA	83.93	48.95102	75.49	2		1 003,0315	2 004,0485	2	-0,001782	-0,8887	601,274	235753
(L)SHGSAQVKGHGKKVADALTN(A)	100 %	NA	81.04	48.834515	68.99	2		669,0262	2 004,0568	3	0,006532	3,258	598,937	322111
(L)SHGSAQVKGHGKKVADALTN(A)	100 %	NA	64.16	49.14047	52.37	2		1 003,0290	2 004,0434	2	-0,006842	-3,412	602,444	203930
(S)HGSAQVKGHGKKVADALTN(A)	100 %	NA	73.67	48.662106	62.98	2		959,5148	1 917,0151	2	-0,003162	-1,649	602,444	73 590,0
(S)HGSAQVKGHGKKVADALTN(A)	98 %	NA	52.56	48.414284	36.49	2		640,015	1 917,0232	3	0,004932	2,571	595,681	110486
(S)HGSAQVKGHGKKVADALTN(A)	97 %	NA	48.69	48.198532	38.32	2		640,0157	1 917,0251	3	0,006852	3,572	596,598	119740
(H)GSAQVKGHGKKVADALTN(A)	100 %	NA	88.32	47.41238	70.65	2		890,9861	1 779,9576	2	-0,001802	-1,012	601,274	63 919,0
(H)GSAQVKGHGKKVADALTN(A)	100 %	NA	76.46	47.611534	59.96	2		890,9847	1 779,9548	2	-0,004602	-2,584	603,611	112302
(H)GSAQVKGHGKKVADALTN(A)	92 %	NA	42.39	47.61274	25.83	2		890,9844	1 779,9542	2	-0,005142	-2,887	602,611	44 641,0
(K)KVADALTNAVAHVDD(M)	97 %	DM	53.88	52.735542	40.38	2		769,8942	1 537,7738	2	-1,942E-06	-0,001262	1 679,87	73 932,0
(K)KVADALTNAVAHVDD(M)	97 %	DM	48.83	48.34929	33.19	2		769,8932	1 537,7718	2	-0,002002	-1,301	1 680,79	36 623,0
(K)VADALTNAVAHVDDMPN(A)	100 %	NA	123.16	47.985474	112.65	2		876,9167	1 751,8189	2	0,003958	2,258	1 918,69	180241
(K)VADALTNAVAHVDDMPN(A)	98 %	NA	50.47	47.77615	43.14	2		876,9144	1 751,8143	2	-0,0006819	-0,3891	1 919,94	13 884,0
(K)VADALTNAVAHVDDMPN(A)	94 %	NA	44.24	47.956505	38.74	2		876,9164	1 751,8181	2	0,003178	1,813	1 919,85	12 766,0
(K)VADALTNAVAHVDDMPNA(L)	100 %	AL	138.59	48.20786	128.1	2		912,436	1 822,8574	2	0,005338	2,927	1 946,70	219090
(K)VADALTNAVAHVDDmPNA(L)	100 %	AL	98.26	47.399597	84.58	2	Oxidation (+16)	920,4297	1 838,8448	2	-0,002197	-1,194	1 805,55	45 965,0
(K)VADALTNAVAHVDDMPNA(L)	100 %	AL	79.73	48.180473	72.38	2		912,4354	1 822,8562	2	0,004158	2,28	1 945,53	63 351,0
(K)VADALTNAVAHVDDmPNA(L)	100 %	AL	67.96	47.281994	62.11	2	Oxidation (+16)	920,4282	1 838,8419	2	-0,005097	-2,77	1 804,55	39 906,0
(K)VADALTNAVAHVDDMPNA(L)	100 %	AL	64.3	48.27537	60.57	2		912,4371	1 822,8596	2	0,007538	4,133	1 947,20	40 982,0
(K)VADALTNAVAHVDDMPNAL(S)	100 %	LS	134.9	49.09417	122.51	2		968,9762	1 935,9378	2	0,001658	0,856	2 116,87	95 323,0
(K)VADALTNAVAHVDDMPNAL(S)	100 %	LS	134.74	49.09529	118.47	2		968,9764	1 935,9383	2	0,002118	1,094	2 116,78	138006
(K)VADALTNAVAHVDDmPNAL(S)	100 %	LS	136.09	48.812706	119.34	2	Oxidation (+16)	976,9732	1 951,9318	2	0,000743	0,3805	1 968,88	161065
(K)VADALTNAVAHVDDmPNAL(S)	100 %	LS	126.14	48.83156	109.26	2	Oxidation (+16)	976,9744	1 951,9343	2	0,003223	1,65	1 968,88	54 436,0

(K)VADALTNAVAHVDDmPNAL(S)	100 %	LS	119.66	52.444626	111.5	2	Oxidation (+16)	976,973	1 951,9314	2	0,000283	0,1449	1 967,88	111740
(K)VADALTNAVAHVDDmPNAL(S)	100 %	LS	101.55	52.508224	89.87	2	Oxidation (+16)	976,9736	1 951,9325	2	0,001463	0,7492	1 967,88	59 243,0
(K)VADALTNAVAHVDDmPNAL(S)	95 %	LS	46.37	48.8975	33.8	2	Oxidation (+16)	976,9752	1 951,9358	2	0,004743	2,429	1 968,88	18 043,0
(K)VADALTNAVAHVDDmPNALS(A)	100 %	SA	105.78	48.557735	95.06	2	Oxidation (+16)	1 020,4901	2 038,9657	2	0,002603	1,276	1 919,19	50 216,0
(K)VADALTNAVAHVDDmPNALS(A)	100 %	SA	104.94	48.558342	93.6	2	Oxidation (+16)	1 020,4894	2 038,9642	2	0,001163	0,5701	1 919,85	37 552,0
(K)VADALTNAVAHVDDMPNALS(A)	100 %	SA	83.4	48.8864	72.93	2		1 012,4917	2 022,9689	2	0,0007381	0,3647	2 077,22	58 510,0
(K)VADALTNAVAHVDDMPNALS(A)	91 %	SA	42.67	48.62251	27.78	2		1 012,4884	2 022,9622	2	-0,005942	-2,936	2 076,06	18 513,0
(V)ADALTNAVAHVDDMPNA(L)	100 %	AL	116.98	46.804714	106.11	2		862,8986	1 723,7826	2	-0,001042	-0,6041	1 897,68	84 655,0
(V)ADALTNAVAHVDDmPNA(L)	100 %	AL	110.89	46.292263	98.66	2	Oxidation (+16)	870,8972	1 739,7799	2	0,001283	0,7371	1 736,72	116837
(V)ADALTNAVAHVDDmPNA(L)	100 %	AL	92.42	46.214775	79.3	2	Oxidation (+16)	870,8961	1 739,7776	2	-0,001017	-0,5842	1 735,63	40 480,0
(V)ADALTNAVAHVDDMPNA(L)	99 %	AL	53.99	46.838394	45.96	2		862,8976	1 723,7807	2	-0,002962	-1,717	1 896,51	22 537,0
(V)ADALTNAVAHVDDmPNAL(S)	100 %	LS	78.39	47.64968	64.46	2	Oxidation (+16)	927,4356	1 852,8567	2	-0,006017	-3,246	1 920,35	42 218,0
(V)ADALTNAVAHVDDmPNAL(S)	100 %	LS	69.99	47.895664	56.09	2	Oxidation (+16)	927,439	1 852,8635	2	0,000803	0,4332	1 921,10	57 200,0
(V)ADALTNAVAHVDDmPNALS(A)	100 %	SA	63.76	47.53376	55.4	2	Oxidation (+16)	970,9545	1 939,8944	2	-0,000297	-0,153	1 871,09	26 219,0
(V)ADALTNAVAHVDDmPNALS(A)	99 %	SA	53.24	47.675636	46.11	2	Oxidation (+16)	970,9563	1 939,8981	2	0,003383	1,743	1 871,17	20 795,0
(A)DALTNAVAHVDD(M)	100 %	DM	61.58	46.594124	48.71	2		1 240,5753	1 239,5680	1	-0,005286	-4,261	1 712,22	8 676,00
(A)DALTNAVAHVDD(M)	99 %	DM	55.05	47.221405	34.86	2		620,7939	1 239,5733	2	0,00001806	0,01456	1 711,22	109952
(A)DALTNAVAHVDD(M)	94 %	DM	43.14	46.61491	24.67	2		1 240,5761	1 239,5688	1	-0,004466	-3,6	1 713,38	6 301,00
(A)DALTNAVAHVDDM(P)	98 %	MP	48.71	46.227528	35.82	2		686,3147	1 370,6149	2	0,001098	0,8006	1 970,38	43 884,0
(A)DALTNAVAHVDDMPNA(L)	100 %	AL	110.69	51.578827	92.8	2		827,3811	1 652,7477	2	0,001138	0,6882	1 946,70	252475
(A)DALTNAVAHVDDMPNA(L)	100 %	AL	101.9	46.866184	84.37	2		827,3825	1 652,7505	2	0,003898	2,357	1 940,85	474405
(A)DALTNAVAHVDDMPNA(L)	100 %	AL	75.09	46.873684	68.5	2		827,3824	1 652,7502	2	0,003658	2,212	1 845,69	25 593,0
(A)DALTNAVAHVDDMPNA(L)	100 %	AL	76.79	51.657696	65.2	2		827,3816	1 652,7487	2	0,002158	1,305	1 942,02	216264
(A)DALTNAVAHVDDmPNA(L)	100 %	AL	68.56	46.16507	49.92	2	Oxidation (+16)	835,3787	1 668,7428	2	0,001323	0,7924	1 793,99	55 963,0
(A)DALTNAVAHVDDMPNA(L)	99 %	AL	59.78	51.40005	53.74	2		827,3803	1 652,7460	2	-0,0005419	-0,3277	1 942,02	98 322,0
(A)DALTNAVAHVDDmPNA(L)	99 %	AL	51.05	45.857765	49.61	2	Oxidation (+16)	835,3778	1 668,7411	2	-0,000397	-0,2377	1 700,64	21 234,0
(A)DALTNAVAHVDDmPNA(L)	98 %	AL	52.02	50.51735	45.55	2	Oxidation (+16)	835,3783	1 668,7421	2	0,000643	0,3851	1 800,06	35 443,0
(A)DALTNAVAHVDDMPNA(L)	93 %	AL	42.17	47.058296	36.54	2		827,3836	1 652,7527	2	0,006158	3,724	1 999,47	18 726,0
(A)DALTNAVAHVDDmPNALS(A)	100 %	SA	128.92	47.458862	109.87	2	Oxidation (+16)	935,4371	1 868,8596	2	0,002063	1,103	1 911,68	399979
(A)DALTNAVAHVDDMPNALS(A)	100 %	SA	118.08	48.052017	101.62	2		927,4397	1 852,8649	2	0,002258	1,218	2 084,20	569566
(A)DALTNAVAHVDDMPNALS(A)	100 %	SA	94.11	48.07427	80.48	2		927,4399	1 852,8653	2	0,002638	1,423	2 083,04	74 368,0
(A)DALTNAVAHVDDMPNALS(A)	100 %	SA	94.3	53.62062	82.42	2		927,4434	1 852,8723	2	0,009618	5,188	2 087,70	566852
(A)DALTNAVAHVDDMPNALS(A)	100 %	SA	91.45	53.110245	77.87	2		927,4391	1 852,8637	2	0,001018	0,5492	2 089,36	334648
(A)DALTNAVAHVDDMPNALS(A)	100 %	SA	91.66	53.471622	76.38	2		927,4425	1 852,8705	2	0,007838	4,228	2 085,37	1114040
(A)DALTNAVAHVDDMPNALS(A)	100 %	SA	59.52	48.148933	44.7	2		927,4412	1 852,8677	2	0,005078	2,739	2 085,53	18 339,0
(A)DALTNAVAHVDDMPNALS(A)	98 %	SA	51.29	47.94516	36.01	2		927,4392	1 852,8638	2	0,001178	0,6355	2 084,45	26 694,0
(A)DALTNAVAHVDDMPNALS(A)	96 %	SA	47.11	47.915436	33.05	2		927,4381	1 852,8615	2	-0,001122	-0,6052	2 024,85	23 819,0
(A)DALTNAVAHVDDmPNALSA(L)	100 %	AL	109.57	47.810654	85.67	2	Oxidation (+16)	970,9576	1 939,9007	2	0,005983	3,083	1 944,36	721764
(A)DALTNAVAHVDDMPNALSA(L)	100 %	AL	109.42	48.336063	98.65	2		962,9611	1 923,9076	2	0,007818	4,062	2 114,45	966857
(A)DALTNAVAHVDDmPNALSA(L)	100 %	AL	96.18	47.56446	80.54	2	Oxidation (+16)	970,9547	1 939,8947	2	0,00006305	0,03248	1 942,02	214038
(A)DALTNAVAHVDDMPNALSA(L)	100 %	AL	93.48	48.253742	82.65	2		962,9605	1 923,9065	2	0,006758	3,511	2 114,53	693645
(A)DALTNAVAHVDDmPNALSA(L)	99 %	AL	54.88	47.671337	43.98	2	Oxidation (+16)	970,956	1 939,8975	2	0,002843	1,465	1 945,70	17 474,0
(A)DALTNAVAHVDDmPNALSA(L)	97 %	AL	47.04	47.53185	36.58	2	Oxidation (+16)	970,9539	1 939,8932	2	-0,001477	-0,761	1 943,27	22 602,0
(D)ALTNAVAHVDDMPN(A)	100 %	NA	83.1	47.453087	67.26	2		734,3499	1 466,6852	2	0,002778	1,893	1 611,86	156608
(D)ALTNAVAHVDDMPN(A)	100 %	NA	76.43	47.458553	60.88	2		734,3498	1 466,6851	2	0,002618	1,784	1 610,78	86 833,0
(D)ALTNAVAHVDDMPN(A)	99 %	NA	58.08	47.45972	44.74	2		734,3495	1 466,6845	2	0,002038	1,389	1 610,86	39 196,0

(D)ALTNAVAHVDDmPN(A)	97 %	NA	47.87	46.874756	23.36	2	Oxidation (+16)	742,3464	1 482,6782	2	0,000843	0,5682	1 294,75	28 359,0
(D)ALTNAVAHVDDMPNALS(A)	100 %	SA	80.18	48.447998	66.0	2		869,9243	1 737,8341	2	-0,001602	-0,9213	1 855,76	103309
(D)ALTNAVAHVDDMPNALS(A)	97 %	SA	49.39	48.68186	37.74	2		869,9253	1 737,8360	2	0,0003781	0,2174	1 858,18	32 368,0
(A)LTNAVAHVDD(M)	100 %	DM	71.32	47.34808	34.54	2		527,7603	1 053,5060	2	-0,003182	-3,017	826,436	53 131,0
(A)LTNAVAHVDD(M)	100 %	DM	59.71	47.35	22.56	2		527,7603	1 053,5061	2	-0,003102	-2,942	875,365	81 023,0
(A)LTNAVAHVDD(M)	100 %	DM	58.14	47.340073	20.95	2		527,7602	1 053,5058	2	-0,003402	-3,226	1 711,05	59 900,0
(A)LTNAVAHVDD(M)	99 %	DM	57.77	47.343834	21.56	2		527,7607	1 053,5069	2	-0,002282	-2,164	851,009	47 494,0
(A)LTNAVAHVDD(M)	99 %	DM	57.54	47.34488	21.08	2		527,7606	1 053,5067	2	-0,002502	-2,373	925,558	84 770,0
(A)LTNAVAHVDD(M)	99 %	DM	53.74	47.340073	16.42	2		527,7601	1 053,5055	2	-0,003622	-3,435	900,998	60 643,0
(A)LTNAVAHVDD(M)	99 %	DM	53.59	47.343834	16.3	2		527,7607	1 053,5068	2	-0,002322	-2,202	1 713,38	158499
(A)LTNAVAHVDD(M)	98 %	DM	49.36	46.825516	15.67	2		527,7609	1 053,5073	2	-0,001902	-1,804	883,516	89 112,0
(A)LTNAVAHVDDmPN(A)	100 %	NA	77.97	46.491108	64.37	2	Oxidation (+16)	706,8275	1 411,6403	2	0,00006305	0,04463	1 000,08	58 080,0
(A)LTNAVAHVDDMPN(A)	100 %	NA	73.11	47.45676	55.76	2		698,8308	1 395,6471	2	0,001718	1,23	1 911,77	124016
(A)LTNAVAHVDDmPN(A)	100 %	NA	71.38	46.317078	61.72	2	Oxidation (+16)	706,8252	1 411,6357	2	-0,004537	-3,212	1 756,66	72 959,0
(A)LTNAVAHVDDmPN(A)	100 %	NA	68.67	46.487698	53.37	2	Oxidation (+16)	706,8276	1 411,6407	2	0,000423	0,2995	1 017,53	81 435,0
(A)LTNAVAHVDDmPN(A)	100 %	NA	64.79	46.76529	52.92	2	Oxidation (+16)	706,8287	1 411,6429	2	0,002603	1,843	1 030,35	69 134,0
(A)LTNAVAHVDDmPN(A)	100 %	NA	64.68	46.706078	54.91	2	Oxidation (+16)	706,8277	1 411,6408	2	0,000563	0,3986	973,21	67 028,0
(A)LTNAVAHVDDmPN(A)	100 %	NA	60.88	49.71531	44.56	2	Oxidation (+16)	706,8263	1 411,6380	2	-0,002237	-1,584	1 009,47	23 741,0
(A)LTNAVAHVDDmPN(A)	99 %	NA	59.46	50.29278	49.23	2	Oxidation (+16)	706,8304	1 411,6461	2	0,005863	4,15	997,913	46 264,0
(A)LTNAVAHVDDMPN(A)	99 %	NA	52.76	47.271263	40.45	2		698,8294	1 395,6443	2	-0,001042	-0,746	1 909,52	50 985,0
(A)LTNAVAHVDDmPNA(L)	100 %	AL	91.29	46.76776	76.14	2	Oxidation (+16)	742,347	1 482,6795	2	0,002143	1,444	1 116,52	333788
(A)LTNAVAHVDDMPNA(L)	100 %	AL	85.51	47.662567	72.41	2		734,3514	1 466,6881	2	0,005678	3,869	1 503,41	271482
(A)LTNAVAHVDDMPNA(L)	100 %	AL	65.06	47.29958	54.04	2		734,3467	1 466,6788	2	-0,003642	-2,481	1 943,19	70 901,0
(A)LTNAVAHVDDmPNA(L)	100 %	AL	61.22	46.48545	49.41	2	Oxidation (+16)	742,342	1 482,6695	2	-0,007897	-5,323	1 095,56	89 374,0
(A)LTNAVAHVDDMPNA(L)	100 %	AL	61.25	47.383606	51.02	2		734,3465	1 466,6784	2	-0,004082	-2,781	1 941,02	42 806,0
(A)LTNAVAHVDDMPNA(L)	96 %	AL	46.84	47.46237	37.61	2		734,3495	1 466,6844	2	0,001898	1,293	1 502,32	133624
(A)LTNAVAHVDDmPNAL(S)	100 %	LS	90.92	48.10844	78.65	2	Oxidation (+16)	798,8879	1 595,7612	2	-0,000237	-0,1484	1 596,68	134789
(A)LTNAVAHVDDmPNAL(S)	100 %	LS	87.19	48.26897	67.76	2	Oxidation (+16)	798,8916	1 595,7686	2	0,007083	4,436	1 597,84	606874
(A)LTNAVAHVDDMPNAL(S)	98 %	LS	55.65	52.401424	38.8	2		790,8896	1 579,7647	2	-0,001862	-1,178	1 839,86	85 424,0
(A)LTNAVAHVDDMPNAL(S)	97 %	LS	48.78	48.41497	34.23	2		790,8912	1 579,7679	2	0,001298	0,8212	1 839,44	36 274,0
(A)LTNAVAHVDDmPNAL(S)	92 %	LS	42.92	48.271366	34.16	2	Oxidation (+16)	798,892	1 595,7695	2	0,008003	5,012	1 598,34	35 992,0
(A)LTNAVAHVDDMPNALS(A)	100 %	SA	87.14	48.394844	69.6	2		834,4082	1 666,8018	2	0,003278	1,965	1 764,75	639301
(A)LTNAVAHVDDMPNALS(A)	100 %	SA	67.89	48.336124	53.61	2		834,4059	1 666,7973	2	-0,001242	-0,7447	1 764,83	571205
(A)LTNAVAHVDDMPNALSA(L)	100 %	AL	107.11	48.64547	85.99	2		869,9247	1 737,8347	2	-0,0009219	-0,5302	1 813,70	77 554,0
(A)LTNAVAHVDDMPNALSA(L)	100 %	AL	100.27	48.634357	86.48	2		869,925	1 737,8354	2	-0,0002619	-0,1506	1 812,54	127793
(A)LTNAVAHVDDmPNALSA(L)	100 %	AL	95.66	48.17526	83.12	2	Oxidation (+16)	877,9238	1 753,8330	2	0,002463	1,404	1 585,01	506826
(A)LTNAVAHVDDMPNALSA(L)	100 %	AL	92.76	48.571632	78.84	2		869,9265	1 737,8384	2	0,002718	1,563	1 813,04	61 854,0
(A)LTNAVAHVDDmPNALSA(L)	100 %	AL	85.01	48.027054	63.83	2	Oxidation (+16)	877,9219	1 753,8292	2	-0,001357	-0,7733	1 583,84	193485
(A)LTNAVAHVDDmPNALSA(L)	100 %	AL	82.09	51.835884	74.41	2	Oxidation (+16)	877,9216	1 753,8287	2	-0,001857	-1,058	1 585,51	74 752,0
(A)LTNAVAHVDDMPNALSA(L)	100 %	AL	75.11	48.550407	60.9	2		869,9261	1 737,8376	2	0,001958	1,126	1 813,70	177305
(A)LTNAVAHVDDMPNALSALSD(L)	100 %	DL	86.92	48.837807	71.21	2		1 027,4993	2 052,9841	2	0,005318	2,589	2 027,18	70 948,0
(A)LTNAVAHVDDMPNALSALSD(L)	100 %	DL	82.83	49.003784	70.04	2		1 027,5018	2 052,9889	2	0,01018	4,955	2 026,27	66 580,0
(A)LTNAVAHVDDmPNALSALSD(L)	99 %	DL	56.33	48.449306	44.62	2	Oxidation (+16)	1 035,4938	2 068,9730	2	-0,000677	-0,327	1 862,68	35 548,0
(A)LTNAVAHVDDMPNALSALSD(L)	95 %	DL	46.31	48.71468	34.17	2		1 027,4960	2 052,9775	2	-0,001302	-0,6339	2 027,43	27 839,0
(A)LTNAVAHVDDmPNALSALSD(L)	91 %	DL	42.18	48.319912	34.1	2	Oxidation (+16)	1 035,4925	2 068,9704	2	-0,003317	-1,602	1 860,43	14 044,0
(A)LTNAVAHVDDMPNALSALSDLH(A)	100 %	HA	72.39	49.515755	59.21	2		768,7143	2 303,1209	3	-0,0008479	-0,368	2 105,23	27 683,0

(A)LTNAVAHVDDMPNALSALSDLH(A)	100 %	HA	70.81	49.55577	60.87	2		768,7155	2 303,1248	3	0,002992	1,299	2 103,99	11 738,0
(A)LTNAVAHVDDmPNALSALSDLH(A)	99 %	HA	57.09	49.27473	51.1	2	Oxidation (+16)	774,0458	2 319,1156	3	-0,001113	-0,4797	1 951,45	26 983,0
(L)TNAVAHVDD(M)	92 %	DM	40.52	45.724068	15.65	2		471,2186	940,4226	2	-0,002502	-2,658	1 711,05	22 372,0
(L)TNAVAHVDD(M)	91 %	DM	39.54	45.724068	15.68	2		471,2186	940,4227	2	-0,002362	-2,509	1 712,22	9 252,00
(L)TNAVAHVDDmPN(A)	100 %	NA	79.28	44.078327	67.79	2	Oxidation (+16)	650,2839	1 298,5532	2	-0,002957	-2,275	1 756,57	65 499,0
(L)TNAVAHVDDMPN(A)	100 %	NA	62.09	45.556625	46.12	2		642,2903	1 282,5660	2	0,004758	3,707	1 051,31	31 926,0
(L)TNAVAHVDDMPN(A)	100 %	NA	60.86	49.45237	45.74	2		642,2867	1 282,5587	2	-0,002522	-1,965	1 910,68	107974
(L)TNAVAHVDDMPN(A)	98 %	NA	48.03	45.516815	34.75	2		642,2904	1 282,5662	2	0,004938	3,847	1 049,31	42 879,0
(L)TNAVAHVDDmPN(A)	97 %	NA	45.07	44.27762	23.0	2	Oxidation (+16)	650,2838	1 298,5530	2	-0,003177	-2,445	1 755,90	54 517,0
(L)TNAVAHVDDMPN(A)	97 %	NA	45.24	45.040085	34.4	2		642,2868	1 282,5590	2	-0,002222	-1,731	1 909,68	44 878,0
(L)TNAVAHVDDMPN(A)	94 %	NA	41.44	45.105446	31.38	2		642,2869	1 282,5593	2	-0,001982	-1,544	1 909,52	27 941,0
(L)TNAVAHVDDMPNAL(S)	100 %	LS	86.64	47.351994	63.26	2		734,3478	1 466,6811	2	-0,001382	-0,9416	1 730,89	175549
(L)TNAVAHVDDmPNAL(S)	100 %	LS	65.06	46.766754	52.92	2	Oxidation (+16)	742,3442	1 482,6738	2	-0,003597	-2,424	1 965,37	82 109,0
(L)TNAVAHVDDMPNAL(S)	100 %	LS	62.99	47.365078	42.02	2		734,3473	1 466,6799	2	-0,002522	-1,718	1 729,97	91 945,0
(L)TNAVAHVDDmPNAL(S)	98 %	LS	49.52	46.7809	39.68	2	Oxidation (+16)	742,3465	1 482,6785	2	0,001083	0,73	1 357,66	38 046,0
(L)TNAVAHVDDmPNAL(S)	91 %	LS	41.07	47.17096	30.38	2	Oxidation (+16)	742,351	1 482,6874	2	0,01	6,742	1 353,17	26 560,0
(L)TNAVAHVDDMPNALSA(L)	100 %	AL	92.28	47.766285	80.38	2		813,3865	1 624,7583	2	0,006778	4,169	1 716,88	620993
(L)TNAVAHVDDMPNALSA(L)	100 %	AL	84.36	47.425365	73.09	2		813,383	1 624,7514	2	-0,0001819	-0,1119	1 715,72	120843
(L)TNAVAHVDDMPNALSA(L)	100 %	AL	78.59	52.84494	63.3	2		813,384	1 624,7534	2	0,001798	1,106	1 716,97	309715
(L)TNAVAHVDDmPNALSA(L)	99 %	AL	51.93	46.77607	42.87	2	Oxidation (+16)	821,3802	1 640,7459	2	-0,000557	-0,3392	1 362,31	48 189,0
(L)TNAVAHVDDMPNALSA(L)	96 %	AL	45.88	47.4311	32.26	2		813,3828	1 624,7510	2	-0,0006019	-0,3703	1 718,05	13 642,0
(L)TNAVAHVDDmPNALSALSDLHAHKLR(V)	99 %	RV	57.71	50.37586	54.86	2	Oxidation (+16)	703,8619	2 811,4186	4	0,009791	3,481	1 717,38	41 547,0
(L)TNAVAHVDDmPNALSALSDLHAHKLR(V)	92 %	RV	45.01	50.380913	41.62	2	Oxidation (+16)	703,8588	2 811,4063	4	-0,002529	-0,8992	1 718,30	28 325,0
(T)NAVAHVDDMPN(A)	99 %	NA	52.87	47.872475	30.77	2		591,7643	1 181,5140	2	0,0003981	0,3366	1 009,47	279770
(T)NAVAHVDDmPN(A)	98 %	NA	48.55	44.236557	30.62	2	Oxidation (+16)	599,7616	1 197,5086	2	0,00008305	0,06929	708,6	76 135,0
(T)NAVAHVDDMPN(A)	97 %	NA	46.57	45.21256	31.63	2		591,7643	1 181,5139	2	0,0003781	0,3197	979,12	40 469,0
(T)NAVAHVDDmPN(A)	91 %	NA	37.81	43.961643	32.0	2	Oxidation (+16)	599,7613	1 197,5081	2	-0,000417	-0,3479	557,102	18 174,0
(T)NAVAHVDDMPNA(L)	100 %	AL	59.37	45.661663	48.04	2		627,2839	1 252,5532	2	0,002498	1,993	1 170,10	198288
(T)NAVAHVDDmPNA(L)	96 %	AL	48.57	49.309036	32.05	2	Oxidation (+16)	635,2821	1 268,5496	2	0,004003	3,153	716,931	60 821,0
(T)NAVAHVDDmPNA(L)	96 %	AL	43.25	44.694096	32.74	2	Oxidation (+16)	635,2807	1 268,5468	2	0,001243	0,9791	712,1	41 185,0
(T)NAVAHVDDMPNA(L)	94 %	AL	46.94	50.55944	37.33	2		627,2854	1 252,5562	2	0,005538	4,418	1 170,60	62 602,0
(T)NAVAHVDDMPNA(L)	93 %	AL	45.39	50.309032	35.37	2		627,2837	1 252,5528	2	0,002178	1,737	1 171,27	47 101,0
(T)NAVAHVDDMPNA(L)	92 %	AL	40.02	45.412544	29.78	2		1 253,5562	1 252,5490	1	-0,001706	-1,361	1 160,80	2 051,00
(T)NAVAHVDDmPNAL(S)	100 %	LS	81.74	46.826862	62.97	2	Oxidation (+16)	691,8234	1 381,6323	2	0,002603	1,883	1 297,00	274239
(T)NAVAHVDDMPNAL(S)	100 %	LS	75.07	47.48955	62.35	2		683,8241	1 365,6336	2	-0,001122	-0,8209	1 706,39	122658
(T)NAVAHVDDMPNAL(S)	100 %	LS	58.43	47.283295	39.89	2		683,8201	1 365,6257	2	-0,009102	-6,66	1 705,31	85 105,0
(T)NAVAHVDDmPNAL(S)	99 %	LS	55.2	46.687584	43.92	2	Oxidation (+16)	691,8217	1 381,6288	2	-0,000917	-0,6632	1 288,86	62 912,0
(T)NAVAHVDDMPNALSALSDLH(A)	100 %	HA	76.44	48.427776	58.37	2		697,336	2 088,9863	3	-0,003678	-1,76	2 055,12	33 241,0
(T)NAVAHVDDmPNALSALSDLH(A)	100 %	HA	65.53	48.62823	57.77	2	Oxidation (+16)	702,6662	2 104,9767	3	-0,008173	-3,881	1 907,34	37 656,0
(T)NAVAHVDDMPNALSALSDLH(A)	99 %	HA	59.64	48.86192	51.56	2		1 045,5072	2 088,9998	2	0,009798	4,688	2 055,29	34 160,0
(T)NAVAHVDDmPNALSALSDLHAHKLR(V)	100 %	RV	114.84	50.272106	113.22	2	Oxidation (+16)	678,5981	2 710,3633	4	0,002211	0,8155	1 714,55	132067
(T)NAVAHVDDmPNALSALSDLHAHKLR(V)	100 %	RV	70.14	50.200462	69.99	2	Oxidation (+16)	678,5996	2 710,3695	4	0,008371	3,087	1 710,13	43 627,0
(T)NAVAHVDDmPNALSALSDLHAHKLR(V)	100 %	RV	64.27	50.27749	51.19	2	Oxidation (+16)	543,0788	2 710,3578	5	-0,003315	-1,223	1 716,05	35 007,0
(T)NAVAHVDDmPNALSALSDLHAHKLR(V)	94 %	RV	46.33	50.23067	45.31	2	Oxidation (+16)	678,5959	2 710,3546	4	-0,006469	-2,386	1 947,20	37 072,0
(N)AVAHVDDMPN(A)	100 %	NA	64.19	45.55723	37.61	2		1 068,4758	1 067,4686	1	-0,002106	-1,971	1 911,02	23 237,0
(N)AVAHVDDMPN(A)	97 %	NA	45.96	45.48242	26.0	2		534,7421	1 067,4696	2	-0,001082	-1,013	952,262	106862

(N)AVAHVDDMPN(A)	97 %	NA	45.81	45.494938	27.49	2		534,7419	1 067,4693	2	-0,001362	-1,275	965,062	43 102,0
(N)AVAHVDDMPN(A)	94 %	NA	42.08	45.637062	26.7	2		534,7423	1 067,4700	2	-0,0006819	-0,6382	920,821	58 832,0
(N)AVAHVDDMPN(A)	94 %	NA	41.88	45.494938	23.99	2		534,7419	1 067,4692	2	-0,001442	-1,35	880,02	46 831,0
(N)AVAHVDDMPN(A)	94 %	NA	41.61	45.44911	23.18	2		534,7415	1 067,4684	2	-0,002222	-2,08	951,098	21 944,0
(N)AVAHVDDMPNA(L)	100 %	AL	57.34	46.212177	43.78	2		570,2615	1 138,5085	2	0,0007181	0,6301	1 068,75	68 815,0
(N)AVAHVDDMPNA(L)	98 %	AL	47.47	45.90686	37.43	2		1 139,5092	1 138,5020	1	-0,005816	-5,104	1 095,65	1 228,00
(N)AVAHVDDMPNA(L)	96 %	AL	44.89	45.767673	31.25	2		570,2614	1 138,5083	2	0,0005581	0,4897	1 068,75	34 061,0
(N)AVAHVDDMPNA(L)	92 %	AL	40.38	45.761223	26.05	2		570,261	1 138,5074	2	-0,0003819	-0,3352	1 111,87	61 590,0
(N)AVAHVDDMPNA(L)	92 %	AL	39.92	45.761223	16.86	2		570,2609	1 138,5071	2	-0,0006219	-0,5458	1 110,70	42 858,0
(N)AVAHVDDMPNA(L)	91 %	AL	39.41	45.738922	29.68	2		570,2595	1 138,5045	2	-0,003282	-2,88	1 113,11	39 686,0
(N)AVAHVDDmPNALS(A)	98 %	SA	49.23	46.881973	32.03	2	Oxidation (+16)	678,3174	1 354,6202	2	0,001443	1,064	1 109,70	39 123,0
(N)AVAHVDDMPNALS(A)	96 %	SA	46.28	47.402122	31.49	2		670,3196	1 338,6246	2	0,0007781	0,5808	1 574,78	25 570,0
(N)AVAHVDDMPNALSALSDLH(A)	100 %	HA	71.98	48.839508	56.85	2		659,3237	1 974,9493	3	0,002202	1,114	2 050,47	76 768,0
(N)AVAHVDDMPNALSALSDLH(A)	100 %	HA	60.74	48.593307	48.46	2		659,321	1 974,9411	3	-0,005988	-3,03	2 049,56	88 786,0
(N)AVAHVDDmPNALSALSDLHAHKLR(V)	100 %	RV	64.23	50.052277	63.61	2	Oxidation (+16)	650,0874	2 596,3206	4	0,002431	0,936	1 699,56	78 105,0
(N)AVAHVDDmPNALSALSDLHAHKLR(V)	100 %	RV	63.23	50.080296	63.23	2	Oxidation (+16)	650,0865	2 596,3170	4	-0,001209	-0,4655	1 702,89	178051
(N)AVAHVDDMPNALSALSDLHAHKLRVDPVN(F)	99 %	NF	60.23	50.49315	57.9	2		777,155	3 104,5909	4	0,008086	2,604	2 074,23	46 773,0
(N)AVAHVDDmPNALSALSDLHAHKLRVDPVN(F)	98 %	NF	52.25	50.672306	30.95	2	Oxidation (+16)	781,1523	3 120,5800	4	0,002331	0,7468	2 016,70	58 799,0
(A)HVDDMPNAL(S)	100 %	LS	59.2	45.651394	45.43	2		1 011,4570	1 010,4497	1	0,0004541	0,4489	1 467,31	9 900,00
(A)HVDDMPNAL(S)	98 %	LS	47.0	45.583244	31.57	2		1 011,4577	1 010,4505	1	0,001184	1,171	1 467,31	9 230,00
(A)HVDDMPNAL(S)	93 %	LS	41.22	46.1721	26.33	2		1 011,4594	1 010,4521	1	0,002864	2,832	1 470,81	10 574,0
(A)HVDDMPNALSALSDLH(A)	100 %	HA	100.22	47.474743	86.15	2		867,9084	1 733,8023	2	-0,002202	-1,269	2 022,52	47 666,0
(A)HVDDmPNALSALSDLH(A)	100 %	HA	80.29	47.050423	68.29	2	Oxidation (+16)	875,9076	1 749,8005	2	0,001163	0,6643	1 884,93	43 289,0
(D)DMPNALSALSDLHAHK(L)	100 %	KL	97.96	49.06658	83.61	2		860,4292	1 718,8437	2	0,002578	1,499	1 902,67	23 600,0
(D)DmPNALSALSDLHAHK(L)	94 %	KL	45.06	48.83633	41.55	2	Oxidation (+16)	868,4252	1 734,8358	2	-0,000297	-0,1711	1 745,40	6 440,00
(D)DmPNALSALSDLHAHKLR(V)	100 %	RV	91.77	49.305977	84.56	2	Oxidation (+16)	669,0148	2 004,0227	3	0,001407	0,7018	1 734,47	47 582,0
(D)DmPNALSALSDLHAHKLR(V)	96 %	RV	48.51	49.31478	38.38	2	Oxidation (+16)	502,0121	2 004,0192	4	-0,002069	-1,032	1 733,30	41 509,0
(D)DmPNALSALSDLHAHKLR(V)	93 %	RV	44.87	49.195694	40.0	2	Oxidation (+16)	1 003,0237	2 004,0329	2	0,0116	5,787	1 736,72	42 478,0
(D)MPNALSALSDLH(A)	100 %	HA	69.36	47.857	48.34	2		634,8193	1 267,6241	2	0,0008981	0,7079	2 051,80	30 623,0
(D)mPNALSALSDLH(A)	99 %	HA	55.72	47.60445	39.2	2	Oxidation (+16)	642,8158	1 283,6170	2	-0,001037	-0,8072	1 926,93	17 792,0
(M)PNALSALSDLHAHKLR(V)	100 %	RV	93.25	47.21959	82.58	2		871,9864	1 741,9582	2	-0,0006619	-0,3798	1 733,22	26 099,0
(M)PNALSALSDLHAHKLR(V)	100 %	RV	71.88	47.25814	61.6	2		581,6588	1 741,9545	3	-0,004388	-2,518	1 875,67	34 668,0
(M)PNALSALSDLHAHKLR(V)	100 %	RV	70.5	47.03197	70.02	2		871,9859	1 741,9573	2	-0,001582	-0,9076	1 875,50	14 806,0
(M)PNALSALSDLHAHKLR(V)	100 %	RV	70.41	47.02999	64.11	2		581,6595	1 741,9566	3	-0,002288	-1,313	1 619,38	30 366,0
(M)PNALSALSDLHAHKLR(V)	100 %	RV	63.65	47.209526	60.09	2		581,6599	1 741,9578	3	-0,001088	-0,6242	1 874,33	19 752,0
(M)PNALSALSDLHAHKLR(V)	97 %	RV	47.1	47.134064	42.89	2		436,4955	1 741,9529	4	-0,006014	-3,45	1 620,04	21 161,0
(M)PNALSALSDLHAHKLR(V)	96 %	RV	45.98	46.735645	43.78	2		871,9888	1 741,9630	2	0,004178	2,397	1 874,83	15 723,0
(M)PNALSALSDLHAHKLR(V)	95 %	RV	44.19	47.156525	42.08	2		581,659	1 741,9552	3	-0,003638	-2,087	1 701,89	28 807,0
(N)ALSALSDLHAHKLR(V)	100 %	RV	121.69	45.87408	87.92	2		766,4389	1 530,8633	2	0,0001581	0,1032	1 239,97	141688
(N)ALSALSDLHAHKLR(V)	100 %	RV	113.77	45.87408	82.7	2		766,439	1 530,8634	2	0,0002781	0,1815	1 237,64	54 114,0
(N)ALSALSDLHAHKLR(V)	100 %	RV	100.04	46.083443	69.09	2		511,2935	1 530,8588	3	-0,004378	-2,858	1 237,64	73 104,0
(N)ALSALSDLHAHKLR(V)	99 %	RV	51.08	45.570618	40.8	2		766,4401	1 530,8656	2	0,002418	1,578	1 128,68	17 719,0
(N)ALSALSDLHAHKLRVDPVN(F)	100 %	NF	96.19	47.382015	75.7	2		686,0481	2 055,1225	3	-0,0001979	-0,09627	1 662,04	143369
(N)ALSALSDLHAHKLRVDPVN(F)	100 %	NF	85.99	47.378998	72.01	2		686,0477	2 055,1214	3	-0,001278	-0,6215	1 659,87	73 266,0
(L)SALSDLHAHKLRVDPVN(F)	100 %	NF	99.43	48.001877	86.62	2		936,5097	1 871,0048	2	0,003298	1,762	1 435,80	179127
(L)SALSDLHAHKLRVDPVN(F)	100 %	NF	94.98	48.141808	76.79	2		624,6741	1 871,0004	3	-0,001028	-0,5491	1 434,55	301375

(L)SALSDLHAHKLRVDPVN(F)	100 %	NF	66.36	48.5124	49.61	2	624,6714	1 870,9923	3	-0,009218	-4,924	1 432,72	72 283,0
(L)SALSDLHAHKLRVDPVN(F)	100 %	NF	65.95	48.21225	51.7	2	624,673	1 870,9971	3	-0,004388	-2,344	1 433,38	103579
(L)SALSDLHAHKLRVDPVN(F)	99 %	NF	53.76	48.36729	44.58	2	624,6736	1 870,9990	3	-0,002468	-1,318	1 590,01	42 330,0
(L)SALSDLHAHKLRVDPVN(F)	93 %	NF	43.85	48.341278	26.19	2	624,6727	1 870,9964	3	-0,005078	-2,713	1 592,01	57 725,0
(S)ALSDLHAHKLRVDPVN(F)	100 %	NF	122.83	50.500546	111.15	2	892,9946	1 783,9746	2	0,005138	2,878	1 248,13	947527
(S)ALSDLHAHKLRVDPVN(F)	100 %	NF	119.57	50.755615	106.47	2	892,9934	1 783,9722	2	0,002758	1,545	1 242,39	1005520
(S)ALSDLHAHKLRVDPVN(F)	100 %	NF	115.44	47.219013	104.68	2	892,9947	1 783,9747	2	0,005278	2,957	1 243,47	995780
(S)ALSDLHAHKLRVDPVN(F)	100 %	NF	110.47	47.277607	94.2	2	892,9953	1 783,9759	2	0,006478	3,629	1 255,11	494060
(S)ALSDLHAHKLRVDPVN(F)	100 %	NF	111.95	50.44391	94.87	2	892,9957	1 783,9769	2	0,007418	4,156	1 252,78	666444
(S)ALSDLHAHKLRVDPVN(F)	100 %	NF	102.65	47.69754	87.65	2	892,9919	1 783,9692	2	-0,0002219	-0,1243	1 245,80	1096700
(S)ALSDLHAHKLRVDPVN(F)	100 %	NF	87.61	47.638546	77.94	2	892,9908	1 783,9670	2	-0,002462	-1,379	1 279,54	80 144,0
(S)ALSDLHAHKLRVDPVN(F)	100 %	NF	76.34	47.44598	67.24	2	892,9931	1 783,9716	2	0,002098	1,175	1 590,84	94 183,0
(S)ALSDLHAHKLRVDPVN(F)	100 %	NF	73.59	47.756104	59.01	2	595,6615	1 783,9625	3	-0,006948	-3,892	1 241,39	300504
(S)ALSDLHAHKLRVDPVN(F)	100 %	NF	65.29	47.681423	50.47	2	595,6636	1 783,9690	3	-0,0004679	-0,2622	1 266,74	452202
(S)ALSDLHAHKLRVDPVN(F)	100 %	NF	63.74	47.287594	54.63	2	595,6652	1 783,9737	3	0,004272	2,393	1 248,13	452871
(S)ALSDLHAHKLRVDPVN(F)	100 %	NF	61.5	47.69754	49.16	2	595,6637	1 783,9692	3	-0,0002279	-0,1277	1 242,39	275716
(S)ALSDLHAHKLRVDPVN(F)	100 %	NF	58.91	47.498672	48.52	2	595,6646	1 783,9721	3	0,002622	1,469	1 246,97	344701
(S)ALSDLHAHKLRVDPVN(F)	100 %	NF	59.05	47.755375	49.67	2	595,6616	1 783,9628	3	-0,006648	-3,724	1 592,01	101013
(S)ALSDLHAHKLRVDPVN(F)	99 %	NF	55.15	47.670074	42.36	2	595,6634	1 783,9683	3	-0,001188	-0,6655	1 242,30	691545
(S)ALSDLHAHKLRVDPVN(F)	99 %	NF	53.06	47.68164	40.02	2	595,6621	1 783,9645	3	-0,004938	-2,766	1 291,18	67 069,0
(S)ALSDLHAHKLRVDPVN(F)	98 %	NF	51.0	47.675194	36.73	2	595,6628	1 783,9665	3	-0,003018	-1,691	1 245,80	303726
(S)ALSDLHAHKLRVDPVN(F)	97 %	NF	47.9	47.660927	33.61	2	595,6618	1 783,9636	3	-0,005838	-3,271	1 589,68	37 045,0
(S)ALSDLHAHKLRVDPVNFK(L)	100 %	KL	75.2	47.27777	60.04	2	515,789	2 059,1269	4	-0,006014	-2,919	1 559,38	142658
(S)ALSDLHAHKLRVDPVNFK(L)	100 %	KL	61.69	47.08922	51.93	2	515,7898	2 059,1303	4	-0,002614	-1,269	1 583,84	53 242,0
(S)ALSDLHAHKLRVDPVNFK(L)	100 %	KL	59.74	46.866806	46.55	2	687,3853	2 059,1339	3	0,001052	0,5107	1 559,38	157859
(S)ALSDLHAHKLRVDPVNFK(L)	99 %	KL	54.24	46.82136	44.3	2	515,7912	2 059,1358	4	0,002906	1,411	1 534,98	89 539,0
(S)ALSDLHAHKLRVDPVNFK(L)	97 %	KL	47.95	46.87841	36.29	2	687,3851	2 059,1334	3	0,0005721	0,2777	1 535,06	86 381,0
(S)ALSDLHAHKLRVDPVNFK(L)	97 %	KL	46.17	46.636917	28.97	2	515,7918	2 059,1381	4	0,005226	2,537	1 733,72	20 729,0
(S)ALSDLHAHKLRVDPVNFK(L)	94 %	KL	43.41	47.25013	33.75	2	515,7896	2 059,1291	4	-0,003734	-1,812	1 961,87	27 420,0
(S)ALSDLHAHKLRVDPVNFK(L)	92 %	KL	41.62	46.87841	32.64	2	687,3851	2 059,1334	3	0,0005121	0,2486	1 583,84	57 392,0
(A)LSDLHAHKLRVDPVNFK(L)	100 %	KL	115.59	46.50113	103.88	2	663,7081	1 988,1026	3	0,006822	3,43	1 369,29	541943
(A)LSDLHAHKLRVDPVNFK(L)	100 %	KL	115.47	46.33024	105.32	2	663,7083	1 988,1030	3	0,007212	3,626	1 361,15	674830
(A)LSDLHAHKLRVDPVNFK(L)	100 %	KL	84.84	46.921505	70.85	2	663,7048	1 988,0927	3	-0,003108	-1,562	1 385,60	506795
(A)LSDLHAHKLRVDPVNFK(L)	100 %	KL	84.18	46.8882	72.66	2	663,7057	1 988,0951	3	-0,0006479	-0,3257	1 378,68	235966
(A)LSDLHAHKLRVDPVNFK(L)	100 %	KL	76.49	46.442715	63.89	2	663,7074	1 988,1003	3	0,004482	2,253	1 359,98	204487
(A)LSDLHAHKLRVDPVNFK(L)	100 %	KL	71.4	46.891403	32.69	2	498,0306	1 988,0931	4	-0,002634	-1,324	1 361,15	365724
(A)LSDLHAHKLRVDPVNFK(L)	100 %	KL	67.19	46.52575	56.69	2	663,7072	1 988,0999	3	0,004122	2,072	1 367,13	83 454,0
(A)LSDLHAHKLRVDPVNFK(L)	100 %	KL	63.87	47.12262	51.51	2	498,0301	1 988,0914	4	-0,004394	-2,209	1 436,96	124689
(A)LSDLHAHKLRVDPVNFK(L)	100 %	KL	61.5	47.06231	51.29	2	498,0303	1 988,0919	4	-0,003874	-1,948	1 904,67	65 695,0
(A)LSDLHAHKLRVDPVNFK(L)	100 %	KL	61.44	47.269142	48.98	2	663,7032	1 988,0878	3	-0,007968	-4,006	1 449,70	122993
(A)LSDLHAHKLRVDPVNFK(L)	100 %	KL	61.26	47.118576	52.84	2	498,0298	1 988,0901	4	-0,005714	-2,873	1 442,70	94 467,0
(A)LSDLHAHKLRVDPVNFK(L)	100 %	KL	60.93	47.060776	43.57	2	498,0303	1 988,0923	4	-0,003514	-1,767	1 872,01	37 235,0
(A)LSDLHAHKLRVDPVNFK(L)	100 %	KL	60.72	47.057014	38.17	2	498,0303	1 988,0922	4	-0,003594	-1,807	1 385,60	96 075,0
(A)LSDLHAHKLRVDPVNFK(L)	100 %	KL	59.98	46.859207	48.11	2	663,7059	1 988,0957	3	-0,00004794	-0,0241	1 901,17	79 310,0
(A)LSDLHAHKLRVDPVNFK(L)	98 %	KL	50.54	46.892822	21.6	2	498,0307	1 988,0937	4	-0,002114	-1,063	1 359,98	111903
(A)LSDLHAHKLRVDPVNFK(L)	97 %	KL	48.17	46.89735	37.18	2	498,0305	1 988,0931	4	-0,002714	-1,364	1 536,06	33 351,0
(A)LSDLHAHKLRVDPVNFK(L)	97 %	KL	47.48	46.888733	30.59	2	498,0306	1 988,0933	4	-0,002514	-1,264	1 511,66	33 981,0

(A)LSDLHAHKLRVDPVNFK(L)	94 %	KL	43.54	47.201923	36.07	2	498,0295	1 988,0887	4	-0,007034	-3,536	1 553,55	28 586,0
(A)LSDLHAHKLRVDPVNFK(L)	91 %	KL	40.54	46.72144	31.42	2	995,0556	1 988,0967	2	0,0009381	0,4716	1 364,64	113043
(A)LSDLHAHKLRVDPVNFK(L)	91 %	KL	40.63	46.949825	28.5	2	663,7056	1 988,0950	3	-0,0007979	-0,4012	1 518,58	51 342,0
(L)SDLHAHKLR(V)	100 %	RV	78.83	46.718857	51.74	2	538,8005	1 075,5864	2	-0,002322	-2,157	603,861	106525
(L)SDLHAHKLR(V)	100 %	RV	73.46	46.87092	48.05	2	538,8019	1 075,5893	2	0,0004981	0,4626	729,624	513194
(L)SDLHAHKLR(V)	100 %	RV	70.31	46.934807	43.12	2	538,7997	1 075,5848	2	-0,003982	-3,699	603,611	234312
(L)SDLHAHKLR(V)	100 %	RV	66.65	46.853745	43.03	2	538,8009	1 075,5873	2	-0,001502	-1,395	754,125	51 949,0
(L)SDLHAHKLR(V)	100 %	RV	65.16	46.852577	37.66	2	538,8011	1 075,5877	2	-0,001062	-0,9864	890,513	92 830,0
(L)SDLHAHKLR(V)	100 %	RV	65.22	46.92671	37.37	2	538,8031	1 075,5917	2	0,002938	2,729	727,29	1052680
(L)SDLHAHKLR(V)	100 %	RV	65.18	46.917183	37.45	2	538,8028	1 075,5911	2	0,002318	2,153	629,239	894480
(L)SDLHAHKLR(V)	100 %	RV	65.13	46.934807	34.95	2	538,7997	1 075,5847	2	-0,004022	-3,736	604,775	498196
(L)SDLHAHKLR(V)	100 %	RV	64.95	46.87092	37.54	2	538,8016	1 075,5886	2	-0,0001819	-0,169	737,787	274072
(L)SDLHAHKLR(V)	100 %	RV	60.56	46.925297	37.91	2	538,8026	1 075,5907	2	0,001898	1,763	728,459	120662
(L)SDLHAHKLR(V)	100 %	RV	58.69	46.927322	31.99	2	538,8033	1 075,5921	2	0,003298	3,063	721,443	122306
(L)SDLHAHKLR(V)	100 %	RV	58.49	46.934807	34.56	2	538,8	1 075,5853	2	-0,003422	-3,178	605,939	37 501,0
(L)SDLHAHKLRVDPVN(F)	100 %	NF	92.45	51.99226	76.21	2	800,9313	1 599,8480	2	-0,0002819	-0,1761	1 075,84	467518
(L)SDLHAHKLRVDPVN(F)	100 %	NF	84.48	48.233437	68.6	2	800,9307	1 599,8468	2	-0,001462	-0,9132	1 100,22	175371
(L)SDLHAHKLRVDPVN(F)	100 %	NF	83.27	52.041878	64.92	2	800,9321	1 599,8496	2	0,001318	0,8233	1 243,55	1045230
(L)SDLHAHKLRVDPVN(F)	100 %	NF	77.75	52.151375	65.07	2	800,9291	1 599,8437	2	-0,004562	-2,85	1 242,55	702012
(L)SDLHAHKLRVDPVN(F)	100 %	NF	72.37	48.22148	58.51	2	800,9315	1 599,8485	2	0,0002181	0,1362	892,844	104245
(L)SDLHAHKLRVDPVN(F)	100 %	NF	61.38	48.27318	47.47	2	800,9337	1 599,8529	2	0,004598	2,872	894,009	292875
(L)SDLHAHKLRVDPVN(F)	100 %	NF	63.86	51.758453	54.32	2	800,9334	1 599,8523	2	0,004038	2,522	1 075,08	266745
(L)SDLHAHKLRVDPVN(F)	99 %	NF	53.35	48.144676	41.71	2	800,9309	1 599,8473	2	-0,0009419	-0,5884	1 436,88	112329
(L)SDLHAHKLRVDPVN(F)	99 %	NF	53.28	48.1304	46.75	2	800,9335	1 599,8524	2	0,004138	2,585	894,092	19 633,0
(L)SDLHAHKLRVDPVN(F)	99 %	NF	52.65	48.14354	42.51	2	800,9312	1 599,8479	2	-0,0004019	-0,2511	902,161	72 981,0
(L)SDLHAHKLRVDPVN(F)	98 %	NF	52.61	48.24484	42.44	2	800,9303	1 599,8460	2	-0,002302	-1,438	1 590,84	75 354,0
(L)SDLHAHKLRVDPVN(F)	98 %	NF	52.49	48.144005	42.3	2	800,9312	1 599,8478	2	-0,0004419	-0,2761	1 243,55	57 931,0
(L)SDLHAHKLRVDPVN(F)	97 %	NF	48.33	48.271107	43.22	2	800,9338	1 599,8530	2	0,004718	2,947	1 435,96	18 334,0
(L)SDLHAHKLRVDPVN(F)	94 %	NF	47.8	51.814575	37.59	2	800,9338	1 599,8529	2	0,004678	2,922	1 085,18	60 250,0
(S)DLHAHKLR(V)	99 %	RV	66.88	45.981995	34.86	2	495,2842	988,5539	2	-0,002842	-2,872	603,861	117932
(S)DLHAHKLR(V)	99 %	RV	67.12	50.479794	34.88	2	495,2848	988,5551	2	-0,001662	-1,679	628,072	345244
(S)DLHAHKLR(V)	99 %	RV	67.19	50.76626	34.87	2	495,2852	988,5557	2	-0,001022	-1,033	628,155	131262
(S)DLHAHKLR(V)	99 %	RV	66.61	50.548965	34.87	2	495,2846	988,5545	2	-0,002222	-2,245	604,941	70 652,0
(S)DLHAHKLR(V)	99 %	RV	59.0	45.981995	23.1	2	495,2847	988,5549	2	-0,001842	-1,861	629,239	463651
(S)DLHAHKLR(V)	99 %	RV	58.69	45.981995	26.77	2	495,2848	988,555	2	-0,001722	-1,74	604,775	148988
(S)DLHAHKLR(V)	99 %	RV	55.68	46.555706	19.83	2	495,2853	988,5561	2	-0,0006419	-0,6487	727,29	230452
(S)DLHAHKLR(V)	93 %	RV	46.33	50.71731	28.98	2	495,2853	988,5561	2	-0,0007019	-0,7093	730,789	28 998,0
(S)DLHAHKLR(V)	92 %	RV	44.88	50.562378	15.44	2	495,2851	988,5557	2	-0,001062	-1,073	731,954	93 179,0
(S)DLHAHKLR(V)	91 %	RV	39.58	45.981995	14.92	2	495,2842	988,5538	2	-0,002962	-2,993	464,071	30 049,0
(S)DLHAHKLRVDPVN(F)	100 %	NF	94.42	47.274925	79.48	2	757,418	1 512,8214	2	0,005178	3,421	944,109	587545
(S)DLHAHKLRVDPVN(F)	100 %	NF	75.91	47.561035	62.5	2	757,4138	1 512,8130	2	-0,003222	-2,128	1 075,92	202998
(S)DLHAHKLRVDPVN(F)	100 %	NF	71.07	52.880142	58.39	2	757,4153	1 512,8160	2	-0,0002619	-0,173	943,028	150850
(S)DLHAHKLRVDPVN(F)	100 %	NF	63.16	47.5131	51.35	2	757,4139	1 512,8133	2	-0,003002	-1,983	1 100,30	112802
(S)DLHAHKLRVDPVN(F)	100 %	NF	67.51	52.8784	55.45	2	757,4153	1 512,8161	2	-0,0002019	-0,1334	944,276	182964
(S)DLHAHKLRVDPVN(F)	100 %	NF	60.64	47.380188	48.63	2	757,4165	1 512,8184	2	0,002158	1,426	1 087,42	49 172,0
(S)DLHAHKLRVDPVN(F)	99 %	NF	53.81	47.664722	40.16	2	505,2789	1 512,8148	3	-0,001458	-0,9631	944,109	121513

(S)DLHAHKLRVDPVN(F)	98 %	NF	50.93	47.680458	27.25	2	757,4152	1 512,8159	2	-0,0003619	-0,2391	941,78	221207
(S)DLHAHKLRVDPVN(F)	94 %	NF	43.58	47.346558	35.02	2	757,4156	1 512,8166	2	0,0003781	0,2497	941,864	32 505,0
(S)DLHAHKLRVDPVN(F)	94 %	NF	43.65	47.655643	27.51	2	757,4148	1 512,8151	2	-0,001162	-0,7676	1 243,63	83 615,0
(S)DLHAHKLRVDPVN(F)	93 %	NF	48.78	53.351105	40.01	2	757,411	1 512,8073	2	-0,008922	-5,894	1 095,90	41 750,0
(S)DLHAHKLRVDPVNFK(L)	100 %	KL	83.64	47.248413	70.13	2	596,9998	1 787,9775	3	-0,002158	-1,206	1 365,88	275524
(S)DLHAHKLRVDPVNFK(L)	100 %	KL	64.6	47.36747	47.18	2	596,9982	1 787,9727	3	-0,007018	-3,923	1 362,39	114694
(D)LHAHKLRVDPVN(F)	100 %	NF	60.35	45.848396	48.53	2	699,9011	1 397,7876	2	-0,001682	-1,202	1 076,09	89 687,0
(D)LHAHKLRVDPVN(F)	100 %	NF	58.35	45.43621	43.92	2	699,9012	1 397,7878	2	-0,001502	-1,074	1 243,80	57 643,0
(D)LHAHKLRVDPVN(F)	99 %	NF	55.88	45.43621	43.47	2	699,9014	1 397,7882	2	-0,001062	-0,7592	801,941	35 435,0
(D)LHAHKLRVDPVN(F)	98 %	NF	48.18	45.848396	32.61	2	699,9011	1 397,7876	2	-0,001682	-1,202	1 592,35	21 960,0
(H)AHKLRVDPVNFK(L)	100 %	KL	75.7	44.022785	58.42	2	712,4134	1 422,8122	2	0,002518	1,769	1 023,36	196487
(H)AHKLRVDPVNFK(L)	98 %	KL	46.38	44.451214	25.48	2	475,2762	1 422,8068	3	-0,002898	-2,035	1 023,36	215702
(H)AHKLRVDPVNFK(L)	97 %	KL	43.84	43.94924	36.14	2	712,4136	1 422,8127	2	0,003018	2,12	1 034,18	32 072,0
(H)AHKLRVDPVNFK(L)	97 %	KL	43.75	43.977837	29.97	2	712,4133	1 422,8120	2	0,002318	1,628	1 022,28	71 475,0
(H)AHKLRVDPVNFK(L)	93 %	KL	39.08	44.08664	24.45	2	712,414	1 422,8134	2	0,003778	2,653	1 382,60	49 795,0
(L)RVDPVNFKLLSH(C)	100 %	HC	66.28	45.599667	50.82	2	475,6039	1 423,7898	3	-0,003828	-2,687	1 700,55	22 229,0
(L)RVDPVNFKLLSH(C)	100 %	HC	61.81	45.681313	47.93	2	475,6043	1 423,7911	3	-0,002598	-1,823	1 701,72	90 699,0
(R)VDPVNFKLL(S)	100 %	LS	57.12	45.948677	35.5	2	522,8072	1 043,5999	2	-0,001782	-1,706	2 079,55	471482
(R)VDPVNFKLL(S)	93 %	LS	41.46	46.18383	25.9	2	522,8081	1 043,6017	2	0,00005806	0,05558	2 079,55	79 401,0
(R)VDPVNFKLL(S)	93 %	LS	40.12	45.14734	20.03	2	522,8097	1 043,6048	2	0,003178	3,042	2 080,71	535448
(R)VDPVNFKLL(S)	92 %	LS	39.97	45.365963	23.9	2	522,8083	1 043,6020	2	0,0002981	0,2853	2 081,87	30 375,0
(R)VDPVNFKLL(S)	91 %	LS	40.25	46.178493	23.01	2	522,8079	1 043,6013	2	-0,0003419	-0,3273	2 078,47	194305
(V)DPVNFKLLSH(C)	100 %	HC	90.68	46.76236	69.8	2	585,318	1 168,6215	2	-0,002642	-2,259	1 841,69	345517
(V)DPVNFKLLSH(C)	100 %	HC	81.73	46.794006	59.81	2	585,3184	1 168,6222	2	-0,002002	-1,712	1 799,73	155416
(V)DPVNFKLLSH(C)	100 %	HC	77.81	52.573593	53.75	2	585,3188	1 168,6231	2	-0,001082	-0,925	1 841,86	139022
(V)DPVNFKLLSH(C)	100 %	HC	77.82	52.633614	54.29	2	585,318	1 168,6214	2	-0,002782	-2,378	1 842,85	243406
(V)DPVNFKLLSH(C)	100 %	HC	76.86	52.640854	56.97	2	585,3186	1 168,6226	2	-0,001602	-1,37	1 798,56	102052
(V)DPVNFKLLSH(C)	100 %	HC	61.48	46.754208	41.47	2	585,3179	1 168,6212	2	-0,002962	-2,532	1 797,48	64 983,0
(V)DPVNFKLLSH(C)	99 %	HC	58.11	52.59701	38.17	2	585,3183	1 168,6220	2	-0,002182	-1,865	1 799,81	25 908,0
(P)VNFKLLSH(C)	99 %	HC	59.83	45.736027	32.91	2	479,2792	956,5439	2	-0,0005019	-0,5242	1 842,85	180593
(P)VNFKLLSH(C)	99 %	HC	59.92	47.096348	33.12	2	479,2787	956,5429	2	-0,001502	-1,569	1 670,21	27 054,0
(P)VNFKLLSH(C)	99 %	HC	58.0	47.096348	33.12	2	479,2789	956,5432	2	-0,001182	-1,234	1 601,43	10 313,0
(P)VNFKLLSH(C)	99 %	HC	54.93	45.680843	17.16	2	479,2797	956,5448	2	0,0004181	0,4366	1 841,77	60 312,0
(C)LLVTLAAHLPAE(F)	100 %	EF	62.03	43.301907	36.72	2	624,369	1 246,7233	2	-0,005322	-4,265	2 048,14	19 410,0
(C)LLVTLAAHLPAE(F)	100 %	EF	57.96	42.90346	35.29	2	624,3706	1 246,7266	2	-0,002042	-1,637	2 047,48	18 905,0
(C)LLVTLAAHLPAE(F)	98 %	EF	44.55	42.8704	27.07	2	624,3709	1 246,7271	2	-0,001522	-1,22	2 048,22	15 444,0
(L)LVTLAAHLPAEFTPA(V)	100 %	AV	92.6	46.36648	84.06	2	775,9338	1 549,8531	2	0,002558	1,649	2 097,01	211693
(L)LVTLAAHLPAEFTPA(V)	100 %	AV	84.52	46.588127	72.98	2	775,933	1 549,8514	2	0,0007981	0,5146	2 095,85	45 947,0
(L)LVTLAAHLPAEFTPA(V)	100 %	AV	72.42	46.276215	61.88	2	775,9333	1 549,8520	2	0,001438	0,9273	2 098,17	27 927,0
(L)LVTLAAHLPAEFTPA(V)	92 %	AV	41.24	46.58326	32.56	2	775,9332	1 549,8518	2	0,001218	0,7854	2 097,18	3 711,00
(L)LVTLAAHLPAEFTPAVHA(S)	100 %	AS	105.56	47.013264	88.81	2	929,5184	1 857,0222	2	0,007198	3,874	2 036,49	278316
(L)LVTLAAHLPAEFTPAVHA(S)	100 %	AS	102.35	47.365562	87.27	2	929,5144	1 857,0143	2	-0,0006419	-0,3455	2 034,16	173866
(L)LVTLAAHLPAEFTPAVHA(S)	100 %	AS	100.69	47.16496	85.33	2	929,5164	1 857,0181	2	0,003178	1,71	2 036,49	571542
(L)LVTLAAHLPAEFTPAVHA(S)	100 %	AS	99.75	47.59101	79.53	2	620,0106	1 857,0098	3	-0,005148	-2,771	2 035,33	51 471,0
(L)LVTLAAHLPAEFTPAVHA(S)	100 %	AS	92.96	47.532154	79.81	2	620,0114	1 857,0125	3	-0,002508	-1,35	2 035,33	64 390,0
(L)LVTLAAHLPAEFTPAVHA(S)	100 %	AS	77.33	47.339672	60.97	2	929,5148	1 857,0150	2	0,00005806	0,03125	2 034,16	84 054,0

(L)LVTLAAHLPAEFTPAVHA(S)	100 %	AS	75.47	47.61364	61.2	2	620,0111	1 857,0115	3	-0,003468	-1,866	2 035,33	58 746,0
(L)LVTLAAHLPAEFTPAVHA(S)	100 %	AS	65.75	47.452545	50.74	2	929,5154	1 857,0161	2	0,001178	0,634	2 033,00	38 943,0
(L)LVTLAAHLPAEFTPAVHA(S)	94 %	AS	43.94	48.023907	34.29	2	620,0136	1 857,0189	3	0,003912	2,105	2 034,33	43 445,0
(L)LVTLAAHLPAEFTPAVHASLDK(F)	100 %	KF	130.83	47.539047	124.9	2	767,7588	2 300,2545	3	0,001382	0,6006	1 977,05	495857
(L)LVTLAAHLPAEFTPAVHASLDK(F)	100 %	KF	122.71	47.53805	118.22	2	767,7588	2 300,2545	3	0,001472	0,6397	1 974,72	99 373,0
(L)LVTLAAHLPAEFTPAVHASLDK(F)	100 %	KF	112.24	47.507015	106.9	2	767,7593	2 300,2561	3	0,003002	1,305	2 001,64	131629
(L)LVTLAAHLPAEFTPAVHASLDK(F)	100 %	KF	104.48	47.53499	99.14	2	767,7588	2 300,2546	3	0,001562	0,6788	2 026,02	106310
(L)LVTLAAHLPAEFTPAVHASLDK(F)	100 %	KF	81.86	47.65728	72.49	2	576,0705	2 300,2528	4	-0,0002939	-0,1277	1 978,22	46 527,0
(L)LVTLAAHLPAEFTPAVHASLDK(F)	98 %	KF	51.05	47.57692	48.55	2	767,7589	2 300,2550	3	0,001922	0,8352	1 973,72	36 037,0
(L)VTLAAHLPAEFTPA(V)	100 %	AV	86.48	47.323616	69.38	2	719,3916	1 436,7687	2	0,002198	1,529	1 982,89	416606
(L)VTLAAHLPAEFTPA(V)	99 %	AV	57.83	53.021164	40.93	2	719,3921	1 436,7696	2	0,003158	2,196	1 983,06	128845
(L)VTLAAHLPAEFTPA(V)	93 %	AV	42.23	47.231106	21.75	2	719,3888	1 436,7630	2	-0,003442	-2,394	1 982,89	72 658,0
(L)VTLAAHLPAEFTPAV(H)	100 %	VH	77.87	46.793915	56.08	2	768,927	1 535,8394	2	0,004498	2,927	2 083,12	487623
(L)VTLAAHLPAEFTPAV(H)	100 %	VH	80.04	50.017426	58.86	2	768,928	1 535,8413	2	0,006478	4,215	2 085,45	685166
(L)VTLAAHLPAEFTPAV(H)	100 %	VH	68.41	46.553497	53.26	2	768,9276	1 535,8406	2	0,005758	3,747	2 083,04	543614
(L)VTLAAHLPAEFTPAV(H)	100 %	VH	68.21	46.553497	44.46	2	768,9275	1 535,8404	2	0,005538	3,604	2 085,37	885085
(L)VTLAAHLPAEFTPAV(H)	91 %	VH	40.63	47.0299	26.87	2	768,9249	1 535,8351	2	0,0002781	0,1809	2 083,37	24 025,0
(L)VTLAAHLPAEFTPAVHA(S)	100 %	AS	97.69	48.332108	83.75	2	582,3156	1 743,9248	3	-0,006048	-3,466	1 931,69	46 712,0
(L)VTLAAHLPAEFTPAVHA(S)	100 %	AS	93.52	48.387608	80.7	2	582,3165	1 743,9277	3	-0,003168	-1,816	1 943,27	33 906,0
(L)VTLAAHLPAEFTPAVHA(S)	100 %	AS	75.94	48.241325	55.62	2	582,3168	1 743,9285	3	-0,002358	-1,351	1 931,60	56 012,0
(L)VTLAAHLPAEFTPAVHA(S)	96 %	AS	47.44	48.338478	33.36	2	582,3171	1 743,9295	3	-0,001338	-0,7668	1 958,37	25 081,0
(L)VTLAAHLPAEFTPAVHAS(L)	100 %	SL	82.63	48.7801	67.17	2	916,4892	1 830,9639	2	0,001058	0,5776	1 905,84	122996
(L)VTLAAHLPAEFTPAVHAS(L)	100 %	SL	61.31	48.61827	49.23	2	916,49	1 830,9654	2	0,002558	1,396	1 903,76	32 175,0
(L)VTLAAHLPAEFTPAVHAS(L)	97 %	SL	50.05	48.83133	38.75	2	611,3258	1 830,9556	3	-0,007268	-3,967	1 908,27	11 824,0
(L)VTLAAHLPAEFTPAVHASLDK(F)	100 %	KF	140.05	48.39359	124.62	2	730,0646	2 187,1719	3	0,002882	1,317	1 917,52	531935
(L)VTLAAHLPAEFTPAVHASLDK(F)	100 %	KF	171.0	48.292076	155.83	2	730,0648	2 187,1727	3	0,003692	1,687	1 886,01	443058
(L)VTLAAHLPAEFTPAVHASLDK(F)	100 %	KF	139.07	48.288532	122.58	2	730,0651	2 187,1734	3	0,004412	2,016	1 893,01	1040890
(L)VTLAAHLPAEFTPAVHASLDK(F)	100 %	KF	156.46	48.031357	142.26	2	730,0671	2 187,1794	3	0,01041	4,758	1 888,34	910322
(L)VTLAAHLPAEFTPAVHASLDK(F)	100 %	KF	116.16	48.276985	97.34	2	1 094,5943	2 187,1740	2	0,005018	2,293	1 888,34	393289
(L)VTLAAHLPAEFTPAVHASLDK(F)	100 %	KF	111.98	48.207397	100.08	2	730,0658	2 187,1754	3	0,006452	2,949	1 887,43	383453
(L)VTLAAHLPAEFTPAVHASLDK(F)	100 %	KF	111.18	48.59096	92.71	2	730,0634	2 187,1684	3	-0,0005679	-0,2596	1 884,84	94 271,0
(L)VTLAAHLPAEFTPAVHASLDK(F)	100 %	KF	103.84	48.665176	88.23	2	730,0619	2 187,1638	3	-0,005218	-2,385	1 942,02	91 280,0
(L)VTLAAHLPAEFTPAVHASLDK(F)	100 %	KF	84.48	48.507137	72.24	2	547,799	2 187,1670	4	-0,001954	-0,893	1 888,34	176730
(L)VTLAAHLPAEFTPAVHASLDK(F)	100 %	KF	73.16	48.768875	65.46	2	730,0605	2 187,1597	3	-0,009298	-4,249	1 893,35	19 778,0
(L)VTLAAHLPAEFTPAVHASLDK(F)	100 %	KF	71.18	48.505116	64.79	2	730,0629	2 187,1670	3	-0,001978	-0,9039	1 966,71	26 675,0
(L)VTLAAHLPAEFTPAVHASLDK(F)	100 %	KF	68.1	49.223152	58.8	2	730,0624	2 187,1654	3	-0,003538	-1,617	1 888,59	54 822,0
(L)VTLAAHLPAEFTPAVHASLDK(F)	100 %	KF	65.0	48.19063	54.75	2	1 094,5954	2 187,1762	2	0,007238	3,308	1 909,35	86 869,0
(L)VTLAAHLPAEFTPAVHASLDK(F)	99 %	KF	53.54	48.276215	45.15	2	730,0653	2 187,1742	3	0,005192	2,373	2 078,39	23 488,0
(L)VTLAAHLPAEFTPAVHASLDK(F)	99 %	KF	54.33	49.26157	46.47	2	730,0545	2 187,1416	3	-0,02742	-12,53	2 129,73	113843
(L)VTLAAHLPAEFTPAVHASLDK(F)	99 %	KF	53.09	48.56463	44.52	2	547,7994	2 187,1683	4	-0,0006339	-0,2897	1 886,09	55 975,0
(L)VTLAAHLPAEFTPAVHASLDK(F)	97 %	KF	49.24	48.356148	35.06	2	1 094,5934	2 187,1722	2	0,003278	1,498	1 912,85	59 411,0
(L)VTLAAHLPAEFTPAVHASLDK(F)	96 %	KF	47.89	48.621613	39.27	2	730,0623	2 187,1650	3	-0,003928	-1,795	2 103,16	19 274,0
(L)VTLAAHLPAEFTPAVHASLDK(F)	95 %	KF	46.16	49.257397	38.58	2	730,0545	2 187,1417	3	-0,0273	-12,48	2 129,82	138597
(L)VTLAAHLPAEFTPAVHASLDK(F)	93 %	KF	44.34	49.116478	34.23	2	730,0658	2 187,1757	3	0,006692	3,058	1 991,56	45 477,0
(V)TLAAHLPAEFTPAV(H)	100 %	VH	98.97	47.39818	80.96	2	719,3891	1 436,7637	2	-0,002782	-1,935	2 015,53	113439
(V)TLAAHLPAEFTPAV(H)	100 %	VH	73.84	53.29351	53.31	2	719,3887	1 436,7628	2	-0,003642	-2,533	2 015,78	101686

(V)TLAAHLPAEFTPAVHA(S)	100 %	AS	93.84	53.981796	76.77	2	823,44	1 644,8655	2	0,003058	1,858	1 874,33	207674
(V)TLAAHLPAEFTPAVHA(S)	100 %	AS	75.47	48.600685	57.59	2	823,4394	1 644,8642	2	0,001738	1,056	1 870,84	148333
(V)TLAAHLPAEFTPAVHA(S)	100 %	AS	71.42	48.606552	52.64	2	823,4384	1 644,8623	2	-0,0001619	-0,09839	1 935,02	129430
(V)TLAAHLPAEFTPAVHA(S)	100 %	AS	71.76	54.284264	56.15	2	823,4372	1 644,8599	2	-0,002582	-1,569	2 034,66	127280
(V)TLAAHLPAEFTPAVHA(S)	100 %	AS	64.62	48.60446	48.14	2	823,4387	1 644,8627	2	0,0002781	0,1689	2 036,49	70 897,0
(V)TLAAHLPAEFTPAVHA(S)	93 %	AS	49.28	54.342	36.8	2	823,4359	1 644,8572	2	-0,005282	-3,209	1 936,19	41 177,0
(V)TLAAHLPAEFTPAVHASLDK(F)	100 %	KF	134.44	48.85288	113.73	2	697,0415	2 088,1026	3	0,001982	0,9488	1 888,42	297601
(V)TLAAHLPAEFTPAVHASLDK(F)	100 %	KF	111.14	48.93162	91.76	2	697,0411	2 088,1016	3	0,0009921	0,4749	1 830,03	395200
(V)TLAAHLPAEFTPAVHASLDK(F)	100 %	KF	70.01	48.809338	54.93	2	697,0417	2 088,1034	3	0,002792	1,336	1 831,69	33 867,0
(V)TLAAHLPAEFTPAVHASLDK(F)	100 %	KF	69.87	49.088867	52.18	2	697,0389	2 088,0949	3	-0,005668	-2,713	1 826,61	77 622,0
(V)TLAAHLPAEFTPAVHASLDK(F)	100 %	KF	67.39	48.84716	57.14	2	697,0406	2 088,1001	3	-0,0005079	-0,2431	1 975,97	58 723,0
(V)TLAAHLPAEFTPAVHASLDK(F)	99 %	KF	56.93	49.030083	42.69	2	1 045,0564	2 088,0983	2	-0,002262	-1,083	1 828,86	49 248,0
(V)TLAAHLPAEFTPAVHASLDK(F)	99 %	KF	55.4	49.02525	42.01	2	1 045,0566	2 088,0985	2	-0,002022	-0,9679	1 889,59	72 963,0
(V)TLAAHLPAEFTPAVHASLDK(F)	99 %	KF	55.11	48.8344	49.35	2	1 045,0601	2 088,1057	2	0,005098	2,44	1 888,51	51 851,0
(V)TLAAHLPAEFTPAVHASLDK(F)	99 %	KF	54.2	49.341904	41.39	2	697,0411	2 088,1015	3	0,0009321	0,4462	1 887,34	194052
(V)TLAAHLPAEFTPAVHASLDK(F)	98 %	KF	52.03	48.94327	28.64	2	697,0397	2 088,0973	3	-0,003268	-1,564	1 825,53	36 401,0
(V)TLAAHLPAEFTPAVHASLDK(F)	94 %	KF	46.05	49.345135	30.76	2	697,0343	2 088,0809	3	-0,01965	-9,405	2 007,46	31 282,0
(T)LAAHLPAEFTPA(V)	98 %	AV	51.5	47.269226	36.63	2	619,3316	1 236,6486	2	-0,001742	-1,407	1 849,93	62 066,0
(T)LAAHLPAEFTPA(V)	98 %	AV	49.92	47.42395	35.93	2	1 237,6534	1 236,6461	1	-0,004226	-3,414	1 847,85	4 637,00
(T)LAAHLPAEFTPA(V)	98 %	AV	48.74	47.2711	34.55	2	619,332	1 236,6495	2	-0,0008619	-0,6964	2 285,99	8 080,00
(T)LAAHLPAEFTPA(V)	95 %	AV	50.9	53.282204	26.25	2	619,3317	1 236,6489	2	-0,001442	-1,165	1 848,02	99 436,0
(T)LAAHLPAEFTPA(V)	95 %	AV	50.86	53.27904	26.08	2	619,3317	1 236,6488	2	-0,001602	-1,294	1 856,93	37 007,0
(T)LAAHLPAEFTPAV(H)	100 %	VH	63.06	50.761013	44.27	2	668,8665	1 335,7184	2	-0,0003619	-0,2708	1 977,05	247437
(T)LAAHLPAEFTPAV(H)	100 %	VH	62.92	50.63581	32.69	2	668,8684	1 335,7223	2	0,003558	2,662	1 972,47	1172800
(T)LAAHLPAEFTPAV(H)	100 %	VH	58.25	46.860638	40.92	2	668,8662	1 335,7178	2	-0,001002	-0,7495	1 971,38	102952
(T)LAAHLPAEFTPAV(H)	98 %	VH	49.76	46.86538	31.35	2	668,8663	1 335,7180	2	-0,0007219	-0,5401	2 334,82	10 447,0
(T)LAAHLPAEFTPAV(H)	94 %	VH	43.86	47.372643	22.59	2	668,8633	1 335,7121	2	-0,006682	-4,999	2 199,97	9 584,00
(T)LAAHLPAEFTPAV(H)	94 %	VH	43.16	46.859207	18.63	2	668,8665	1 335,7184	2	-0,0003219	-0,2408	2 333,65	9 458,00
(T)LAAHLPAEFTPAV(H)	93 %	VH	41.95	46.6529	19.62	2	668,8696	1 335,7247	2	0,005958	4,457	2 332,49	9 877,00
(T)LAAHLPAEFTPAV(H)	93 %	VH	42.19	47.024307	8.0	2	668,8686	1 335,7227	2	0,003898	2,916	1 973,64	114977
(T)LAAHLPAEFTPAV(H)	91 %	VH	40.53	46.860638	24.08	2	668,8662	1 335,7178	2	-0,001002	-0,7495	2 346,44	11 976,0
(T)LAAHLPAEFTPAVHASLDK(F)	100 %	KF	158.23	48.56868	147.58	2	663,3596	1 987,0570	3	0,004162	2,094	1 789,24	940583
(T)LAAHLPAEFTPAVHASLDK(F)	100 %	KF	132.09	48.56868	120.62	2	663,3597	1 987,0571	3	0,004252	2,139	1 888,34	674256
(T)LAAHLPAEFTPAVHASLDK(F)	100 %	KF	131.18	53.001907	120.42	2	663,3601	1 987,0583	3	0,005452	2,742	1 768,26	433785
(T)LAAHLPAEFTPAVHASLDK(F)	100 %	KF	123.33	48.391384	112.58	2	663,3609	1 987,0608	3	0,007882	3,965	1 792,74	509485
(T)LAAHLPAEFTPAVHASLDK(F)	100 %	KF	120.36	48.68603	111.81	2	663,3576	1 987,0509	3	-0,002018	-1,015	1 765,92	553446
(T)LAAHLPAEFTPAVHASLDK(F)	100 %	KF	121.85	48.646297	111.1	2	663,3594	1 987,0563	3	0,003412	1,716	1 789,24	237921
(T)LAAHLPAEFTPAVHASLDK(F)	100 %	KF	122.82	53.01544	112.27	2	663,3604	1 987,0593	3	0,006412	3,225	1 795,07	591815
(T)LAAHLPAEFTPAVHASLDK(F)	100 %	KF	117.28	48.755417	100.92	2	663,3573	1 987,0500	3	-0,002828	-1,422	1 912,94	143088
(T)LAAHLPAEFTPAVHASLDK(F)	100 %	KF	117.04	48.71415	104.79	2	663,3591	1 987,0554	3	0,002542	1,279	1 788,08	294871
(T)LAAHLPAEFTPAVHASLDK(F)	100 %	KF	110.47	48.767605	102.56	2	663,3579	1 987,0517	3	-0,001148	-0,5774	1 825,36	560148
(T)LAAHLPAEFTPAVHASLDK(F)	100 %	KF	107.55	48.596542	97.38	2	663,3586	1 987,0541	3	0,001222	0,6147	1 874,33	105002
(T)LAAHLPAEFTPAVHASLDK(F)	100 %	KF	107.62	48.57658	94.56	2	994,5345	1 987,0544	2	0,001558	0,7837	1 767,25	320887
(T)LAAHLPAEFTPAVHASLDK(F)	100 %	KF	110.52	53.229465	100.77	2	663,3577	1 987,0511	3	-0,001748	-0,8792	1 776,50	423186
(T)LAAHLPAEFTPAVHASLDK(F)	100 %	KF	104.51	48.80465	93.72	2	994,5304	1 987,0462	2	-0,006642	-3,341	1 818,37	133930
(T)LAAHLPAEFTPAVHASLDK(F)	100 %	KF	101.63	48.646416	92.57	2	994,5354	1 987,0561	2	0,003278	1,649	1 793,91	515101

(T)LAAHLPAEFTPAVHASLDK(F)	100 %	KF	106.92	54.186344	93.28	2	994,5337	1 987,0528	2	-0,00002194	-0,01104	1 767,17	586904
(T)LAAHLPAEFTPAVHASLDK(F)	100 %	KF	103.29	53.118214	93.91	2	663,3584	1 987,0534	3	0,0005621	0,2827	1 820,70	267501
(T)LAAHLPAEFTPAVHASLDK(F)	100 %	KF	103.42	53.30511	90.93	2	663,3561	1 987,0465	3	-0,006368	-3,203	1 766,00	354277
(T)LAAHLPAEFTPAVHASLDK(F)	100 %	KF	95.94	48.71094	79.44	2	994,5338	1 987,0531	2	0,0001981	0,09962	1 889,51	155872
(T)LAAHLPAEFTPAVHASLDK(F)	100 %	KF	93.22	48.57658	86.0	2	663,3588	1 987,0545	3	0,001642	0,826	1 813,70	688494
(T)LAAHLPAEFTPAVHASLDK(F)	100 %	KF	92.4	48.711056	82.35	2	663,3574	1 987,0505	3	-0,002408	-1,211	1 849,85	175285
(T)LAAHLPAEFTPAVHASLDK(F)	100 %	KF	84.42	48.760155	73.89	2	663,3571	1 987,0496	3	-0,003308	-1,664	2 109,80	42 648,0
(T)LAAHLPAEFTPAVHASLDK(F)	100 %	KF	83.51	48.70977	67.59	2	994,5326	1 987,0506	2	-0,002262	-1,138	1 765,92	61 158,0
(T)LAAHLPAEFTPAVHASLDK(F)	100 %	KF	80.0	48.922615	72.34	2	497,7695	1 987,0489	4	-0,003934	-1,979	1 765,92	145071
(T)LAAHLPAEFTPAVHASLDK(F)	100 %	KF	74.95	48.916546	63.78	2	497,7696	1 987,0493	4	-0,003574	-1,798	1 790,41	104602
(T)LAAHLPAEFTPAVHASLDK(F)	100 %	KF	70.66	48.86135	63.39	2	663,3567	1 987,0483	3	-0,004568	-2,298	2 012,04	41 041,0
(T)LAAHLPAEFTPAVHASLDK(F)	100 %	KF	70.71	49.03215	63.58	2	994,528	1 987,0414	2	-0,01148	-5,775	1 765,25	82 457,0
(T)LAAHLPAEFTPAVHASLDK(F)	100 %	KF	68.0	48.760155	61.69	2	663,3571	1 987,0496	3	-0,003308	-1,664	1 819,53	128864
(T)LAAHLPAEFTPAVHASLDK(F)	100 %	KF	70.38	53.161488	58.55	2	663,358	1 987,0521	3	-0,0007279	-0,3662	2 058,94	41 100,0
(T)LAAHLPAEFTPAVHASLDK(F)	100 %	KF	69.2	53.289787	63.13	2	663,3562	1 987,0469	3	-0,005978	-3,007	1 766,17	266744
(T)LAAHLPAEFTPAVHASLDK(F)	100 %	KF	62.57	48.7586	44.16	2	497,7691	1 987,0473	4	-0,005534	-2,784	1 839,36	39 553,0
(T)LAAHLPAEFTPAVHASLDK(F)	99 %	KF	63.92	53.229755	53.53	2	663,3576	1 987,0511	3	-0,001778	-0,8943	2 109,88	33 025,0
(T)LAAHLPAEFTPAVHASLDK(F)	99 %	KF	59.57	48.919777	52.07	2	663,357	1 987,0491	3	-0,003818	-1,92	1 937,52	34 196,0
(T)LAAHLPAEFTPAVHASLDK(F)	99 %	KF	63.66	53.305885	56.02	2	663,3561	1 987,0466	3	-0,006308	-3,173	2 034,50	34 451,0
(T)LAAHLPAEFTPAVHASLDK(F)	99 %	KF	57.58	48.596542	47.42	2	663,3586	1 987,0541	3	0,001222	0,6147	2 090,11	29 638,0
(T)LAAHLPAEFTPAVHASLDK(F)	99 %	KF	61.41	53.449856	51.03	2	663,3554	1 987,0444	3	-0,008468	-4,259	1 817,37	142679
(T)LAAHLPAEFTPAVHASLDK(F)	99 %	KF	55.54	48.814358	42.03	2	497,7693	1 987,0480	4	-0,004894	-2,462	1 865,01	29 845,0
(T)LAAHLPAEFTPAVHASLDK(F)	99 %	KF	54.37	48.767776	44.31	2	663,3578	1 987,0515	3	-0,001328	-0,668	2 066,76	29 591,0
(T)LAAHLPAEFTPAVHASLDK(F)	99 %	KF	53.1	48.72797	36.79	2	994,5352	1 987,0557	2	0,002878	1,448	1 769,51	28 732,0
(T)LAAHLPAEFTPAVHASLDK(F)	98 %	KF	52.78	48.754143	42.22	2	663,3572	1 987,0497	3	-0,003128	-1,573	1 986,38	51 397,0
(T)LAAHLPAEFTPAVHASLDK(F)	98 %	KF	56.77	53.260612	51.91	2	663,3572	1 987,0498	3	-0,003068	-1,543	1 964,46	114392
(T)LAAHLPAEFTPAVHASLDK(F)	98 %	KF	51.62	48.637573	42.41	2	994,5353	1 987,0560	2	0,003178	1,599	2 119,20	33 207,0
(T)LAAHLPAEFTPAVHASLDK(F)	98 %	KF	51.8	48.86203	45.77	2	497,7694	1 987,0485	4	-0,004334	-2,18	1 889,59	42 454,0
(T)LAAHLPAEFTPAVHASLDK(F)	98 %	KF	51.39	48.91972	41.18	2	497,7696	1 987,0491	4	-0,003774	-1,898	1 793,91	45 137,0
(T)LAAHLPAEFTPAVHASLDK(F)	97 %	KF	53.64	53.134075	47.99	2	663,3578	1 987,0515	3	-0,001328	-0,668	1 923,68	54 980,0
(T)LAAHLPAEFTPAVHASLDK(F)	96 %	KF	46.64	48.7017	34.64	2	663,3577	1 987,0514	3	-0,001508	-0,7585	1 961,87	62 634,0
(T)LAAHLPAEFTPAVHASLDK(F)	95 %	KF	45.76	48.754433	34.82	2	663,3573	1 987,0500	3	-0,002858	-1,438	2 095,85	27 208,0
(T)LAAHLPAEFTPAVHASLDK(F)	94 %	KF	45.36	48.9084	38.29	2	663,3547	1 987,0424	3	-0,01051	-5,286	1 785,08	25 237,0
(T)LAAHLPAEFTPAVHASLDK(F)	91 %	KF	42.8	48.91972	29.22	2	994,5318	1 987,0491	2	-0,003802	-1,912	1 842,85	30 728,0
(T)LAAHLPAEFTPAVHASLDK(F)	91 %	KF	42.34	48.76922	32.12	2	994,533	1 987,0515	2	-0,001402	-0,7052	1 914,02	10 356,0
(T)LAAHLPAEFTPAVHASLDKF(L)	100 %	KF	78.5	48.932014	69.14	2	712,3815	2 134,1226	3	0,001342	0,6286	2 000,39	70 067,0
(T)LAAHLPAEFTPAVHASLDKF(L)	100 %	KF	61.63	48.969173	51.29	2	712,3812	2 134,1217	3	0,0003821	0,1789	1 949,03	83 789,0
(T)LAAHLPAEFTPAVHASLDKF(L)	98 %	KF	50.34	48.894585	38.64	2	712,3809	2 134,1208	3	-0,0005179	-0,2426	1 998,14	25 575,0
(L)AAHLPAEFTPAV(H)	100 %	VH	61.05	47.47272	42.5	2	612,3239	1 222,6332	2	-0,001422	-1,162	1 864,34	170976
(L)AAHLPAEFTPAV(H)	99 %	VH	54.64	47.418842	33.89	2	612,3246	1 222,6347	2	-1,942E-06	-0,001587	1 865,01	135741
(L)AAHLPAEFTPAVHASLDK(F)	100 %	KF	136.24	49.077984	125.91	2	625,6633	1 873,9680	3	-0,0008179	-0,4362	1 768,26	272690
(L)AAHLPAEFTPAVHASLDK(F)	100 %	KF	135.87	48.99804	121.24	2	625,6646	1 873,9719	3	0,003172	1,692	1 667,88	609572
(L)AAHLPAEFTPAVHASLDK(F)	100 %	KF	127.9	49.079216	115.62	2	625,6622	1 873,9649	3	-0,003878	-2,068	1 770,60	149696
(L)AAHLPAEFTPAVHASLDK(F)	100 %	KF	128.47	49.231457	117.9	2	625,6628	1 873,9665	3	-0,002258	-1,204	1 888,42	215581
(L)AAHLPAEFTPAVHASLDK(F)	100 %	KF	125.57	49.05758	114.76	2	625,6643	1 873,9712	3	0,002392	1,276	1 667,97	316566
(L)AAHLPAEFTPAVHASLDK(F)	100 %	KF	116.32	49.18591	102.09	2	937,9904	1 873,9663	2	-0,002482	-1,324	1 889,51	105154
(L)AAHLPAEFTPAVHASLDK(F)	100 %	KF	114.27	49.146812	104.45	2	625,6624	1 873,9653	3	-0,003488	-1,86	1 831,19	83 068,0

(L)AAHLPAEFTPAVHASLDK(F)	100 %	KF	112.28	49.137024	99.14	2	937,9927	1 873,9709	2	0,002118	1,13	1 770,68	83 230,0
(L)AAHLPAEFTPAVHASLDK(F)	100 %	KF	112.27	49.20108	98.53	2	625,663	1 873,9672	3	-0,001598	-0,8522	1 792,74	193955
(L)AAHLPAEFTPAVHASLDK(F)	100 %	KF	106.93	49.174267	95.3	2	625,6636	1 873,9691	3	0,0002921	0,1558	1 767,17	119445
(L)AAHLPAEFTPAVHASLDK(F)	100 %	KF	104.48	49.060764	90.45	2	937,9915	1 873,9684	2	-0,0003219	-0,1717	1 669,05	524375
(L)AAHLPAEFTPAVHASLDK(F)	100 %	KF	93.68	49.082275	78.93	2	937,9895	1 873,9644	2	-0,004322	-2,305	1 666,72	92 329,0
(L)AAHLPAEFTPAVHASLDK(F)	100 %	KF	89.76	49.236378	78.94	2	625,6625	1 873,9658	3	-0,003008	-1,604	1 692,46	87 733,0
(L)AAHLPAEFTPAVHASLDK(F)	100 %	KF	84.35	49.092403	67.49	2	625,6653	1 873,9739	3	0,005152	2,748	1 665,63	124425
(L)AAHLPAEFTPAVHASLDK(F)	100 %	KF	81.53	49.077553	65.54	2	937,9913	1 873,9680	2	-0,0007219	-0,385	1 768,42	39 435,0
(L)AAHLPAEFTPAVHASLDK(F)	100 %	KF	73.98	49.146812	66.95	2	625,6625	1 873,9656	3	-0,003218	-1,716	1 978,47	24 951,0
(L)AAHLPAEFTPAVHASLDK(F)	100 %	KF	73.76	49.143642	66.21	2	625,6642	1 873,9707	3	0,001912	1,02	1 676,12	42 555,0
(L)AAHLPAEFTPAVHASLDK(F)	100 %	KF	71.43	49.072876	62.01	2	469,4994	1 873,9684	4	-0,0003539	-0,1888	1 677,20	174827
(L)AAHLPAEFTPAVHASLDK(F)	99 %	KF	57.41	49.146812	48.38	2	625,6624	1 873,9653	3	-0,003488	-1,86	1 718,22	27 256,0
(L)AAHLPAEFTPAVHASLDK(F)	99 %	KF	57.32	49.198204	42.31	2	625,6601	1 873,9584	3	-0,01036	-5,524	1 912,94	34 553,0
(L)AAHLPAEFTPAVHASLDK(F)	97 %	KF	50.37	49.23187	45.78	2	625,6625	1 873,9657	3	-0,003068	-1,636	1 886,34	54 340,0
(L)AAHLPAEFTPAVHASLDK(F)	97 %	KF	49.18	49.235756	39.72	2	937,9906	1 873,9666	2	-0,002142	-1,142	1 665,55	25 013,0
(L)AAHLPAEFTPAVHASLDK(F)	96 %	KF	47.49	49.17437	32.09	2	937,9919	1 873,9693	2	0,0004981	0,2656	1 888,34	23 618,0
(L)AAHLPAEFTPAVHASLDK(F)	94 %	KF	45.39	49.078575	35.77	2	469,4993	1 873,9679	4	-0,0008739	-0,4661	1 667,88	43 828,0
(L)AAHLPAEFTPAVHASLDK(F)	93 %	KF	44.31	49.23845	34.41	2	625,6613	1 873,9620	3	-0,006818	-3,636	1 817,45	28 544,0
(A)AHLPAEFTPA(V)	99 %	AV	57.71	47.21588	38.39	2	1 053,5297	1 052,5224	1	-0,006726	-6,384	1 628,22	38 334,0
(A)AHLPAEFTPA(V)	99 %	AV	55.19	47.02051	34.99	2	1 053,5313	1 052,5240	1	-0,005196	-4,932	1 628,30	33 573,0
(A)AHLPAEFTPA(V)	95 %	AV	44.26	47.36245	22.62	2	1 053,5322	1 052,5249	1	-0,004286	-4,068	1 628,22	30 895,0
(A)AHLPAEFTPAV(H)	100 %	VH	69.83	47.19812	46.05	2	1 152,6017	1 151,5945	1	-0,003106	-2,695	1 835,94	6 805,00
(A)AHLPAEFTPAV(H)	93 %	VH	42.21	47.201675	27.28	2	1 152,6011	1 151,5938	1	-0,003736	-3,241	1 838,19	9 561,00
(A)AHLPAEFTPAVHA(S)	100 %	AS	77.92	48.49493	63.36	2	680,8527	1 359,6908	2	-0,002742	-2,015	1 816,21	93 481,0
(A)AHLPAEFTPAVHA(S)	100 %	AS	74.95	48.44881	53.49	2	680,856	1 359,6974	2	0,003818	2,806	1 652,79	79 522,0
(A)AHLPAEFTPAVHA(S)	100 %	AS	69.78	48.401184	56.08	2	680,853	1 359,6915	2	-0,002082	-1,53	1 816,21	106772
(A)AHLPAEFTPAVHA(S)	100 %	AS	66.58	52.49966	43.97	2	680,8563	1 359,6980	2	0,004398	3,232	1 649,37	920900
(A)AHLPAEFTPAVHA(S)	99 %	AS	53.7	48.694958	39.38	2	680,8526	1 359,6906	2	-0,002922	-2,147	1 813,87	48 745,0
(A)AHLPAEFTPAVHA(S)	92 %	AS	43.36	48.528152	29.62	2	680,8521	1 359,6897	2	-0,003882	-2,853	1 649,45	25 823,0
(A)AHLPAEFTPAVHA(S)	92 %	AS	42.63	48.499165	33.3	2	680,8556	1 359,6965	2	0,002978	2,189	1 677,71	26 746,0
(A)AHLPAEFTPAVHA(S)	91 %	AS	42.3	48.421535	21.5	2	680,8531	1 359,6917	2	-0,001882	-1,383	1 813,79	32 038,0
(A)AHLPAEFTPAVHA(S)	91 %	AS	41.87	48.33345	31.71	2	680,8537	1 359,6929	2	-0,0006819	-0,5012	1 676,12	39 678,0
(A)AHLPAEFTPAVHAS(L)	100 %	SL	76.48	48.80116	62.48	2	724,37	1 446,7253	2	-0,0002219	-0,1533	1 589,84	102538
(A)AHLPAEFTPAVHAS(L)	100 %	SL	63.51	48.795147	40.55	2	724,3698	1 446,7251	2	-0,0004819	-0,3329	1 589,68	331222
(A)AHLPAEFTPAVHASLD(K)	100 %	DK	90.69	49.040497	79.33	2	838,4264	1 674,8382	2	0,001558	0,9297	1 795,07	340294
(A)AHLPAEFTPAVHASLD(K)	100 %	DK	83.86	48.987747	69.77	2	838,4247	1 674,8348	2	-0,001842	-1,099	1 793,91	113694
(A)AHLPAEFTPAVHASLD(K)	99 %	DK	58.91	48.921055	51.87	2	838,4277	1 674,8408	2	0,004178	2,493	1 795,15	32 409,0
(A)AHLPAEFTPAVHASLDK(F)	100 %	KF	141.87	49.140415	131.58	2	601,9841	1 802,9304	3	-0,001228	-0,6807	1 627,05	470677
(A)AHLPAEFTPAVHASLDK(F)	100 %	KF	141.85	48.99716	127.47	2	601,9852	1 802,9339	3	0,002222	1,232	1 651,54	286819
(A)AHLPAEFTPAVHASLDK(F)	100 %	KF	127.26	49.107048	114.51	2	601,9839	1 802,9298	3	-0,001888	-1,047	1 768,26	245473
(A)AHLPAEFTPAVHASLDK(F)	100 %	KF	124.43	49.086082	114.68	2	902,4715	1 802,9285	2	-0,003162	-1,753	1 627,05	242206
(A)AHLPAEFTPAVHASLDK(F)	100 %	KF	119.18	49.024433	104.45	2	902,4746	1 802,9347	2	0,003038	1,684	1 629,38	876648
(A)AHLPAEFTPAVHASLDK(F)	100 %	KF	109.12	49.140415	98.17	2	902,4725	1 802,9304	2	-0,001222	-0,6774	1 770,60	267546
(A)AHLPAEFTPAVHASLDK(F)	100 %	KF	104.06	49.103844	93.22	2	601,984	1 802,9303	3	-0,001408	-0,7805	1 792,74	197877
(A)AHLPAEFTPAVHASLDK(F)	100 %	KF	102.67	49.12387	91.44	2	902,4703	1 802,9259	2	-0,005722	-3,172	1 769,59	177628
(A)AHLPAEFTPAVHASLDK(F)	100 %	KF	99.79	49.00657	93.35	2	601,9849	1 802,9328	3	0,001112	0,6165	1 676,04	128795

(A)AHLPAEFTPAVHASLDK(F)	100 %	KF	94.33	49.15547	83.28	2	902,4728	1 802,9311	2	-0,0005819	-0,3226	1 653,87	225569
(A)AHLPAEFTPAVHASLDK(F)	100 %	KF	94.06	49.136868	86.77	2	601,9836	1 802,9290	3	-0,002638	-1,462	1 625,96	139695
(A)AHLPAEFTPAVHASLDK(F)	100 %	KF	93.39	49.15838	80.93	2	601,9846	1 802,9319	3	0,0002121	0,1176	1 700,55	82 356,0
(A)AHLPAEFTPAVHASLDK(F)	100 %	KF	91.6	49.14962	73.46	2	902,4709	1 802,9273	2	-0,004362	-2,418	1 782,24	76 680,0
(A)AHLPAEFTPAVHASLDK(F)	100 %	KF	91.04	49.14962	79.28	2	601,9831	1 802,9273	3	-0,004348	-2,41	1 889,51	132657
(A)AHLPAEFTPAVHASLDK(F)	100 %	KF	86.25	49.086082	71.28	2	902,4717	1 802,9287	2	-0,002922	-1,62	1 889,59	105771
(A)AHLPAEFTPAVHASLDK(F)	100 %	KF	83.87	49.112213	73.69	2	601,9822	1 802,9249	3	-0,006808	-3,774	1 725,05	76 932,0
(A)AHLPAEFTPAVHASLDK(F)	100 %	KF	76.04	49.05812	67.13	2	601,985	1 802,9332	3	0,001562	0,8659	1 749,57	43 897,0
(A)AHLPAEFTPAVHASLDK(F)	100 %	KF	74.68	49.12233	67.54	2	902,4702	1 802,9258	2	-0,005862	-3,25	1 667,97	24 282,0
(A)AHLPAEFTPAVHASLDK(F)	100 %	KF	72.5	49.12233	61.95	2	902,4701	1 802,9257	2	-0,005982	-3,316	1 625,96	51 297,0
(A)AHLPAEFTPAVHASLDK(F)	100 %	KF	70.34	49.107048	62.63	2	601,9839	1 802,9298	3	-0,001888	-1,047	1 996,89	25 113,0
(A)AHLPAEFTPAVHASLDK(F)	100 %	KF	68.0	49.19763	48.32	2	601,9797	1 802,9172	3	-0,01446	-8,015	1 923,35	40 719,0
(A)AHLPAEFTPAVHASLDK(F)	100 %	KF	66.41	49.156586	55.23	2	601,9843	1 802,9312	3	-0,0004779	-0,2649	1 358,99	31 702,0
(A)AHLPAEFTPAVHASLDK(F)	100 %	KF	65.82	49.150463	57.69	2	601,9832	1 802,9279	3	-0,003808	-2,111	1 870,84	43 157,0
(A)AHLPAEFTPAVHASLDK(F)	100 %	KF	63.11	49.037407	54.65	2	902,4736	1 802,9327	2	0,0009981	0,5533	1 678,37	42 129,0
(A)AHLPAEFTPAVHASLDK(F)	100 %	KF	60.45	49.090744	51.62	2	601,9833	1 802,9281	3	-0,003568	-1,978	2 021,36	21 488,0
(A)AHLPAEFTPAVHASLDK(F)	99 %	KF	58.6	49.091225	51.41	2	601,9838	1 802,9295	3	-0,002158	-1,196	1 972,47	26 612,0
(A)AHLPAEFTPAVHASLDK(F)	99 %	KF	56.53	49.124077	50.91	2	601,9829	1 802,9268	3	-0,004888	-2,71	1 841,77	45 212,0
(A)AHLPAEFTPAVHASLDK(F)	98 %	KF	53.33	49.05812	48.13	2	601,985	1 802,9331	3	0,001412	0,7828	2 078,39	24 908,0
(A)AHLPAEFTPAVHASLDK(F)	98 %	KF	52.91	49.037357	44.34	2	902,4734	1 802,9322	2	0,0004981	0,2761	1 702,97	17 337,0
(A)AHLPAEFTPAVHASLDK(F)	98 %	KF	52.77	49.157005	46.63	2	601,9845	1 802,9316	3	-0,0001179	-0,06538	2 045,81	21 942,0
(A)AHLPAEFTPAVHASLDK(F)	96 %	KF	48.52	49.156586	44.26	2	902,473	1 802,9314	2	-0,0002819	-0,1563	1 806,72	18 692,0
(A)AHLPAEFTPAVHASLDK(F)	96 %	KF	47.12	48.9619	42.89	2	902,4762	1 802,9378	2	0,006158	3,414	2 013,46	16 509,0
(A)AHLPAEFTPAVHASLDK(F)	92 %	KF	43.39	49.14962	33.4	2	601,983	1 802,9273	3	-0,004378	-2,427	1 947,95	19 306,0
(A)HLPAEFTPAVHA(S)	100 %	AS	73.19	48.36299	58.99	2	645,3359	1 288,6573	2	0,0008181	0,6343	1 622,38	153840
(A)HLPAEFTPAVHA(S)	100 %	AS	74.41	54.24048	56.61	2	645,3335	1 288,6524	2	-0,004102	-3,181	1 819,53	60 310,0
(A)HLPAEFTPAVHA(S)	100 %	AS	62.94	48.668716	48.68	2	645,3331	1 288,6516	2	-0,004862	-3,77	1 817,21	149764
(A)HLPAEFTPAVHA(S)	99 %	AS	59.52	54.2404	39.9	2	645,3335	1 288,6525	2	-0,003962	-3,072	1 814,96	121015
(A)HLPAEFTPAVHA(S)	97 %	AS	54.47	54.148865	42.0	2	645,3341	1 288,6536	2	-0,002822	-2,188	1 816,12	52 288,0
(A)HLPAEFTPAVHA(S)	96 %	AS	47.38	48.346054	31.56	2	645,3356	1 288,6567	2	0,0002181	0,1691	1 688,96	124130
(A)HLPAEFTPAVHASLDK(F)	100 %	KF	130.12	48.941	116.21	2	578,3057	1 731,8953	3	0,0007321	0,4224	1 613,03	380675
(A)HLPAEFTPAVHASLDK(F)	100 %	KF	113.45	49.12504	98.74	2	866,9525	1 731,8904	2	-0,004162	-2,402	1 768,34	72 576,0
(A)HLPAEFTPAVHASLDK(F)	100 %	KF	112.6	49.082813	99.14	2	866,9529	1 731,8913	2	-0,003262	-1,882	1 670,30	63 202,0
(A)HLPAEFTPAVHASLDK(F)	100 %	KF	109.78	49.17369	94.4	2	866,9541	1 731,8936	2	-0,0009419	-0,5436	1 889,51	174405
(A)HLPAEFTPAVHASLDK(F)	100 %	KF	108.77	48.98747	95.32	2	866,9534	1 731,8923	2	-0,002282	-1,317	1 769,51	232071
(A)HLPAEFTPAVHASLDK(F)	100 %	KF	97.56	49.08844	87.07	2	578,305	1 731,8932	3	-0,001368	-0,7894	1 769,43	167325
(A)HLPAEFTPAVHASLDK(F)	100 %	KF	88.51	48.985275	75.21	2	578,3049	1 731,8929	3	-0,001698	-0,9798	1 610,69	67 907,0
(A)HLPAEFTPAVHASLDK(F)	100 %	KF	87.18	48.9284	78.76	2	578,3059	1 731,8959	3	0,001332	0,7687	1 889,67	43 865,0
(A)HLPAEFTPAVHASLDK(F)	100 %	KF	84.48	49.176105	71.58	2	866,9542	1 731,8938	2	-0,0008019	-0,4628	1 613,03	111861
(A)HLPAEFTPAVHASLDK(F)	100 %	KF	82.79	49.04499	71.63	2	578,3053	1 731,8941	3	-0,0004679	-0,27	1 669,05	151530
(A)HLPAEFTPAVHASLDK(F)	100 %	KF	70.82	48.91571	55.43	2	578,3062	1 731,8966	3	0,002052	1,184	1 637,54	82 645,0
(A)HLPAEFTPAVHASLDK(F)	100 %	KF	60.71	48.785164	45.56	2	866,9572	1 731,8998	2	0,005238	3,023	1 637,54	49 321,0
(A)HLPAEFTPAVHASLDK(F)	99 %	KF	57.98	49.087795	49.54	2	578,3051	1 731,8935	3	-0,001098	-0,6336	1 888,84	39 319,0
(A)HLPAEFTPAVHASLDK(F)	99 %	KF	56.23	49.145126	46.51	2	578,3042	1 731,8907	3	-0,003858	-2,226	1 609,69	31 160,0
(A)HLPAEFTPAVHASLDK(F)	98 %	KF	52.95	49.17411	42.66	2	866,9541	1 731,8937	2	-0,0008619	-0,4974	1 888,34	41 914,0
(A)HLPAEFTPAVHASLDK(F)	96 %	KF	48.42	49.124508	40.77	2	578,3043	1 731,8910	3	-0,003618	-2,088	1 662,29	43 130,0
(H)LPAEFTPAVHA(S)	100 %	AS	79.85	47.409626	49.61	2	576,8056	1 151,5967	2	-0,0008819	-0,7652	1 767,09	211189

(H)LPAEFTPAVHA(S)	100 %	AS	77.63	47.254543	47.78	2	576,8059	1 151,5973	2	-0,0002619	-0,2273	1 645,70	106887
(H)LPAEFTPAVHA(S)	100 %	AS	73.42	47.254868	44.37	2	576,8059	1 151,5971	2	-0,0004219	-0,3661	1 643,37	33 403,0
(H)LPAEFTPAVHA(S)	100 %	AS	68.38	47.409626	41.51	2	576,8055	1 151,5964	2	-0,001122	-0,9734	1 766,09	69 765,0
(H)LPAEFTPAVHASL(D)	100 %	LD	89.09	47.481415	69.16	2	676,8633	1 351,7120	2	-0,001702	-1,258	1 830,11	156152
(H)LPAEFTPAVHASL(D)	100 %	LD	88.35	51.57339	64.77	2	676,863	1 351,7115	2	-0,002162	-1,598	1 938,60	846367
(H)LPAEFTPAVHASL(D)	100 %	LD	78.3	47.491257	55.92	2	676,8604	1 351,7062	2	-0,007422	-5,487	1 937,35	184840
(H)LPAEFTPAVHASL(D)	100 %	LD	77.64	51.611732	57.81	2	676,8632	1 351,7119	2	-0,001802	-1,332	1 831,28	156755
(H)LPAEFTPAVHASL(D)	100 %	LD	72.55	47.29586	48.99	2	676,8628	1 351,7111	2	-0,002582	-1,909	1 826,70	79 287,0
(H)LPAEFTPAVHASL(D)	100 %	LD	72.83	47.5969	55.74	2	676,8616	1 351,7086	2	-0,005082	-3,757	1 937,44	134586
(H)LPAEFTPAVHASL(D)	100 %	LD	72.32	47.327232	47.09	2	676,8617	1 351,7088	2	-0,004902	-3,624	1 826,78	56 195,0
(H)LPAEFTPAVHASLD(K)	98 %	DK	51.14	48.21579	45.04	2	734,3768	1 466,7391	2	-0,001602	-1,091	1 897,76	35 819,0
(H)LPAEFTPAVHASLD(K)	91 %	DK	42.46	48.365013	35.06	2	734,3772	1 466,7398	2	-0,0008619	-0,5873	1 723,22	31 004,0
(H)LPAEFTPAVHASLDK(F)	100 %	KF	132.33	47.865387	110.01	2	798,4268	1 594,8390	2	0,003358	2,104	1 628,22	698690
(H)LPAEFTPAVHASLDK(F)	100 %	KF	123.34	47.99251	105.13	2	798,4256	1 594,8365	2	0,0008781	0,5502	1 627,13	164764
(H)LPAEFTPAVHASLDK(F)	100 %	KF	122.66	48.169304	99.58	2	798,4261	1 594,8376	2	0,001938	1,214	1 769,43	639364
(H)LPAEFTPAVHASLDK(F)	100 %	KF	126.54	47.905807	103.31	2	798,4276	1 594,8407	2	0,005058	3,17	1 721,56	724432
(H)LPAEFTPAVHASLDK(F)	100 %	KF	118.65	47.93497	96.08	2	798,4262	1 594,8379	2	0,002258	1,415	1 720,39	213937
(H)LPAEFTPAVHASLDK(F)	100 %	KF	112.54	48.097214	90.35	2	798,4226	1 594,8306	2	-0,005082	-3,184	1 768,26	206746
(H)LPAEFTPAVHASLDK(F)	100 %	KF	102.25	47.937347	82.81	2	798,4261	1 594,8377	2	0,002058	1,29	1 677,20	86 509,0
(H)LPAEFTPAVHASLDK(F)	100 %	KF	97.06	47.891434	73.31	2	798,4266	1 594,8386	2	0,002938	1,841	1 889,51	128687
(H)LPAEFTPAVHASLDK(F)	100 %	KF	97.01	48.006966	76.37	2	798,425	1 594,8354	2	-0,0002219	-0,1391	1 652,71	170992
(H)LPAEFTPAVHASLDK(F)	100 %	KF	94.66	48.19004	71.76	2	798,4236	1 594,8327	2	-0,002942	-1,844	1 793,99	55 310,0
(H)LPAEFTPAVHASLDK(F)	100 %	KF	88.81	47.86283	67.01	2	798,4271	1 594,8397	2	0,003998	2,505	1 888,59	80 981,0
(H)LPAEFTPAVHASLDK(F)	100 %	KF	80.07	48.332172	67.88	2	532,6159	1 594,8258	3	-0,009888	-6,196	1 719,55	104348
(H)LPAEFTPAVHASLDK(F)	100 %	KF	67.83	48.0465	50.3	2	532,6187	1 594,8344	3	-0,001278	-0,8008	1 720,39	113130
(H)LPAEFTPAVHASLDK(F)	100 %	KF	63.56	48.074947	43.74	2	798,4229	1 594,8313	2	-0,004342	-2,721	1 818,45	17 283,0
(H)LPAEFTPAVHASLDK(F)	100 %	KF	62.03	48.32394	49.96	2	532,6157	1 594,8252	3	-0,01052	-6,591	1 888,84	25 335,0
(H)LPAEFTPAVHASLDK(F)	99 %	KF	53.49	48.005657	41.48	2	532,6191	1 594,8356	3	-0,00007794	-0,04884	1 889,67	24 712,0
(H)LPAEFTPAVHASLDK(F)	92 %	KF	42.42	47.86482	30.94	2	798,4269	1 594,8393	2	0,003638	2,28	1 712,38	17 009,0
(L)PAEFTPAVHA(S)	100 %	AS	70.36	47.04485	50.19	2	520,2643	1 038,5140	2	0,0005781	0,5561	1 873,42	22 011,0
(L)PAEFTPAVHA(S)	100 %	AS	63.4	47.33141	35.16	2	520,2634	1 038,5122	2	-0,001242	-1,195	1 934,10	18 027,0
(L)PAEFTPAVHA(S)	100 %	AS	63.31	47.571224	33.13	2	1 039,5204	1 038,5131	1	-0,0003859	-0,3713	1 645,70	17 266,0
(L)PAEFTPAVHA(S)	100 %	AS	62.59	47.601887	44.04	2	1 039,5210	1 038,5137	1	0,0002041	0,1963	1 646,87	17 193,0
(L)PAEFTPAVHA(S)	99 %	AS	57.35	47.419548	41.94	2	1 039,5185	1 038,5112	1	-0,002256	-2,17	1 644,78	10 935,0
(L)PAEFTPAVHA(S)	99 %	AS	57.1	47.184185	36.68	2	520,2614	1 038,5083	2	-0,005202	-5,004	2 036,74	14 059,0
(L)PAEFTPAVHA(S)	99 %	AS	54.77	47.601887	36.23	2	1 039,5205	1 038,5132	1	-0,0002259	-0,2174	1 821,86	8 868,00
(L)PAEFTPAVHA(S)	99 %	AS	54.31	47.571224	29.89	2	520,2636	1 038,5126	2	-0,0009019	-0,8677	1 644,87	27 633,0
(L)PAEFTPAVHA(S)	99 %	AS	54.12	47.571224	31.19	2	520,2635	1 038,5125	2	-0,0009419	-0,9061	1 767,34	36 992,0
(L)PAEFTPAVHA(S)	98 %	AS	50.75	47.33141	33.26	2	520,2634	1 038,5122	2	-0,001262	-1,214	1 768,26	57 201,0
(L)PAEFTPAVHA(S)	97 %	AS	54.01	53.2567	38.3	2	520,2626	1 038,5106	2	-0,002842	-2,734	1 816,54	23 592,0
(L)PAEFTPAVHA(S)	96 %	AS	46.53	47.469612	22.78	2	1 039,5178	1 038,5105	1	-0,002946	-2,834	1 688,04	14 394,0
(L)PAEFTPAVHA(S)	95 %	AS	44.23	47.32892	28.94	2	1 039,5192	1 038,5119	1	-0,001576	-1,516	1 644,78	15 860,0
(L)PAEFTPAVHA(S)	92 %	AS	41.13	46.882507	21.9	2	1 039,5227	1 038,5154	1	0,001944	1,87	1 655,04	5 770,00
(L)PAEFTPAVHA(S)	91 %	AS	41.41	47.601887	25.83	2	1 039,5207	1 038,5134	1	-0,00008594	-0,08267	1 645,70	25 608,0
(L)PAEFTPAVHA(S)	91 %	AS	40.86	47.184185	25.12	2	520,2614	1 038,5082	2	-0,005242	-5,043	1 439,38	19 961,0
(L)PAEFTPAVHASLDK(F)	100 %	KF	117.58	48.16301	92.48	2	741,8821	1 481,7497	2	-0,001882	-1,269	1 768,26	416023

(L)PAEFTPAVHASLDK(F)	100 %	KF	115.01	48.333702	90.84	2	741,8847	1 481,7548	2	0,003198	2,157	1 629,38	999242
(L)PAEFTPAVHASLDK(F)	100 %	KF	107.31	48.227562	71.51	2	741,8836	1 481,7526	2	0,001078	0,7271	1 653,87	224563
(L)PAEFTPAVHASLDK(F)	100 %	KF	106.09	48.23852	87.43	2	494,9231	1 481,7475	3	-0,004068	-2,744	1 722,72	195037
(L)PAEFTPAVHASLDK(F)	100 %	KF	99.78	48.222202	76.35	2	741,8837	1 481,7529	2	0,001318	0,8889	1 792,74	203100
(L)PAEFTPAVHASLDK(F)	100 %	KF	96.6	48.22325	80.36	2	741,8838	1 481,7531	2	0,001518	1,024	1 889,51	303184
(L)PAEFTPAVHASLDK(F)	100 %	KF	96.3	48.207794	71.34	2	741,8844	1 481,7541	2	0,002578	1,739	1 888,34	111456
(L)PAEFTPAVHASLDK(F)	100 %	KF	95.41	48.177967	70.2	2	741,882	1 481,7493	2	-0,002222	-1,499	1 615,37	203588
(L)PAEFTPAVHASLDK(F)	100 %	KF	95.56	48.467083	62.52	2	741,8832	1 481,7519	2	0,0002981	0,201	1 457,86	207558
(L)PAEFTPAVHASLDK(F)	100 %	KF	95.03	48.30229	84.85	2	494,9247	1 481,7523	3	0,0007021	0,4735	1 721,89	45 592,0
(L)PAEFTPAVHASLDK(F)	100 %	KF	91.28	48.23852	74.66	2	494,9231	1 481,7475	3	-0,004068	-2,744	1 721,56	37 959,0
(L)PAEFTPAVHASLDK(F)	100 %	KF	91.33	48.31921	71.71	2	494,923	1 481,7471	3	-0,004518	-3,047	1 888,42	45 397,0
(L)PAEFTPAVHASLDK(F)	100 %	KF	89.93	48.274796	74.6	2	494,9248	1 481,7525	3	0,0009721	0,6556	1 767,09	24 247,0
(L)PAEFTPAVHASLDK(F)	100 %	KF	89.01	48.327003	56.0	2	741,8803	1 481,7460	2	-0,005602	-3,778	1 938,52	129125
(L)PAEFTPAVHASLDK(F)	100 %	KF	88.69	48.16301	70.84	2	741,8822	1 481,7498	2	-0,001742	-1,175	1 914,11	61 788,0
(L)PAEFTPAVHASLDK(F)	100 %	KF	86.63	48.30229	51.05	2	741,8834	1 481,7523	2	0,0006981	0,4708	1 678,37	128027
(L)PAEFTPAVHASLDK(F)	100 %	KF	76.56	48.392517	60.18	2	494,9245	1 481,7516	3	0,00001206	0,008132	1 671,55	12 045,0
(L)PAEFTPAVHASLDK(F)	100 %	KF	76.39	48.376137	58.89	2	741,8825	1 481,7505	2	-0,001082	-0,7297	1 613,03	61 418,0
(L)PAEFTPAVHASLDK(F)	100 %	KF	76.22	48.311848	55.43	2	741,8804	1 481,7462	2	-0,005362	-3,616	1 629,55	83 776,0
(L)PAEFTPAVHASLDK(F)	100 %	KF	75.87	48.16301	57.5	2	494,9238	1 481,7495	3	-0,002088	-1,408	1 457,86	142337
(L)PAEFTPAVHASLDK(F)	100 %	KF	75.61	48.207794	54.08	2	741,8843	1 481,7540	2	0,002418	1,631	1 887,67	122210
(L)PAEFTPAVHASLDK(F)	100 %	KF	73.11	48.392517	41.43	2	741,8831	1 481,7515	2	-0,00002194	-0,0148	1 722,72	92 124,0
(L)PAEFTPAVHASLDK(F)	100 %	KF	70.75	48.157837	38.41	2	741,8823	1 481,7500	2	-0,001602	-1,08	1 455,52	59 845,0
(L)PAEFTPAVHASLDK(F)	100 %	KF	69.65	48.319977	34.51	2	741,8806	1 481,7467	2	-0,004902	-3,306	1 770,93	38 576,0
(L)PAEFTPAVHASLDK(F)	100 %	KF	69.61	48.333702	36.63	2	741,8846	1 481,7546	2	0,003058	2,062	1 828,94	43 217,0
(L)PAEFTPAVHASLDK(F)	100 %	KF	65.81	48.16301	42.12	2	494,9238	1 481,7496	3	-0,001938	-1,307	1 769,43	52 233,0
(L)PAEFTPAVHASLDK(F)	100 %	KF	64.68	48.16414	51.46	2	494,924	1 481,7503	3	-0,001278	-0,8619	1 793,91	42 592,0
(L)PAEFTPAVHASLDK(F)	100 %	KF	61.93	48.319977	48.29	2	494,9228	1 481,7465	3	-0,005028	-3,391	1 455,52	30 777,0
(L)PAEFTPAVHASLDK(F)	100 %	KF	60.13	48.371777	45.21	2	741,8862	1 481,7578	2	0,006198	4,18	1 817,21	29 844,0
(L)PAEFTPAVHASLDK(F)	99 %	KF	58.3	48.155907	44.75	2	741,8824	1 481,7502	2	-0,001362	-0,9185	1 747,48	15 019,0
(L)PAEFTPAVHASLDK(F)	99 %	KF	55.56	48.406643	34.57	2	741,8829	1 481,7512	2	-0,0003419	-0,2306	1 853,35	32 223,0
(L)PAEFTPAVHASLDK(F)	99 %	KF	55.04	48.48275	34.29	2	741,8795	1 481,7444	2	-0,007182	-4,844	1 773,01	22 256,0
(L)PAEFTPAVHASLDK(F)	99 %	KF	54.04	48.31921	32.8	2	741,8808	1 481,7471	2	-0,004482	-3,023	1 782,32	31 201,0
(L)PAEFTPAVHASLDK(F)	96 %	KF	47.25	48.235783	36.71	2	494,9231	1 481,7473	3	-0,004248	-2,865	1 623,63	15 635,0
(L)PAEFTPAVHASLDK(F)	91 %	KF	41.81	48.18001	20.59	2	741,8818	1 481,7491	2	-0,002482	-1,674	1 877,00	32 198,0
(P)AEFTPAVHASLDK(F)	100 %	KF	78.53	47.959145	49.84	2	693,355	1 384,6955	2	-0,003302	-2,383	1 722,72	141850
(P)AEFTPAVHASLDK(F)	100 %	KF	74.62	47.982018	45.86	2	693,3547	1 384,6948	2	-0,003942	-2,845	1 629,47	198564
(P)AEFTPAVHASLDK(F)	100 %	KF	74.64	54.130768	43.39	2	693,3552	1 384,6958	2	-0,002962	-2,138	1 630,55	118841
(P)AEFTPAVHASLDK(F)	100 %	KF	67.0	48.200375	28.63	2	693,356	1 384,6975	2	-0,001242	-0,8963	1 628,30	93 733,0
(P)AEFTPAVHASLDK(F)	100 %	KF	61.93	48.36242	36.93	2	693,3579	1 384,7012	2	0,002438	1,759	1 888,84	35 964,0
(P)AEFTPAVHASLDK(F)	100 %	KF	60.45	47.869995	29.83	2	693,3565	1 384,6983	2	-0,0004219	-0,3045	1 768,59	73 572,0
(P)AEFTPAVHASLDK(F)	99 %	KF	55.06	48.200897	33.61	2	693,3558	1 384,6970	2	-0,001802	-1,3	1 792,99	55 836,0
(P)AEFTPAVHASLDK(F)	99 %	KF	60.22	54.108734	35.46	2	693,3565	1 384,6984	2	-0,0003619	-0,2612	1 723,97	98 749,0
(P)AEFTPAVHASLDK(F)	98 %	KF	49.72	47.97434	15.2	2	693,3561	1 384,6977	2	-0,001042	-0,7519	1 658,79	34 590,0
(E)FTPAVHASLDK(F)	100 %	KF	92.07	47.42615	67.63	2	593,3177	1 184,6208	2	0,001738	1,466	1 136,34	369940
(E)FTPAVHASLDK(F)	100 %	KF	91.88	47.620106	67.64	2	593,3144	1 184,6143	2	-0,004782	-4,033	1 888,51	83 069,0
(E)FTPAVHASLDK(F)	100 %	KF	79.5	47.411198	59.16	2	593,3153	1 184,6159	2	-0,003122	-2,633	1 792,82	171296
(E)FTPAVHASLDK(F)	100 %	KF	66.61	47.501225	44.92	2	593,3162	1 184,6178	2	-0,001222	-1,031	1 766,17	43 586,0

(E)FTPAVHASLDK(F)	100 %	KF	65.22	47.50362	43.46	2	593,316	1 184,6175	2	-0,001582	-1,334	1 767,09	78 154,0
(E)FTPAVHASLDK(F)	100 %	KF	63.07	47.431885	42.13	2	593,3154	1 184,6162	2	-0,002882	-2,431	1 791,57	173485
(E)FTPAVHASLDK(F)	100 %	KF	66.89	53.1016	49.98	2	593,3181	1 184,6217	2	0,002658	2,242	1 138,67	225722
(E)FTPAVHASLDK(F)	100 %	KF	66.32	53.42703	45.0	2	593,3158	1 184,6171	2	-0,001962	-1,655	1 768,51	33 133,0
(E)FTPAVHASLDK(F)	99 %	KF	63.64	53.380096	34.22	2	593,3165	1 184,6184	2	-0,0006419	-0,5414	1 767,59	38 366,0
(E)FTPAVHASLDK(F)	99 %	KF	63.17	53.22575	40.96	2	593,3178	1 184,6209	2	0,001878	1,584	1 135,42	367535
(E)FTPAVHASLDK(F)	99 %	KF	56.23	47.30734	39.71	2	593,3188	1 184,6231	2	0,004018	3,389	1 196,22	44 960,0
(E)FTPAVHASLDK(F)	99 %	KF	53.05	47.43118	36.14	2	593,3186	1 184,6226	2	0,003578	3,018	1 167,86	30 567,0
(E)FTPAVHASLDK(F)	99 %	KF	52.83	47.236282	26.02	2	593,3169	1 184,6191	2	0,00007806	0,06584	1 677,20	89 256,0
(E)FTPAVHASLDK(F)	99 %	KF	52.86	47.43118	39.33	2	593,3186	1 184,6227	2	0,003638	3,068	1 195,05	18 015,0
(E)FTPAVHASLDK(F)	99 %	KF	57.99	53.550835	34.26	2	593,3146	1 184,6146	2	-0,004482	-3,78	1 643,53	37 729,0
(E)FTPAVHASLDK(F)	98 %	KF	56.9	53.435936	40.64	2	593,3153	1 184,6160	2	-0,003082	-2,599	1 771,09	41 245,0
(E)FTPAVHASLDK(F)	98 %	KF	50.39	47.271343	36.77	2	593,3134	1 184,6123	2	-0,006762	-5,703	1 885,34	22 080,0
(E)FTPAVHASLDK(F)	97 %	KF	47.67	46.903465	32.26	2	593,318	1 184,6215	2	0,002438	2,056	1 723,89	60 033,0
(E)FTPAVHASLDK(F)	96 %	KF	45.78	47.236282	29.94	2	1 185,6264	1 184,6192	1	0,00009406	0,07933	1 641,53	4 953,00
(E)FTPAVHASLDK(F)	95 %	KF	45.19	47.450825	29.12	2	593,3167	1 184,6189	2	-0,0001419	-0,1197	1 238,98	24 137,0
(E)FTPAVHASLDK(F)	95 %	KF	44.6	46.882595	31.53	2	593,3181	1 184,6216	2	0,002538	2,141	1 628,22	35 960,0
(E)FTPAVHASLDK(F)	95 %	KF	44.68	47.29626	29.36	2	593,321	1 184,6275	2	0,008418	7,1	1 295,17	13 842,0
(E)FTPAVHASLDK(F)	92 %	KF	42.06	47.431885	21.59	2	593,3153	1 184,6161	2	-0,002962	-2,498	1 238,14	9 251,00
(F)TPAVHASLDK(F)	100 %	KF	87.97	46.621532	63.65	2	519,7827	1 037,5508	2	0,0001581	0,1522	1 332,49	39 506,0
(F)TPAVHASLDK(F)	100 %	KF	75.35	46.865734	57.82	2	519,7807	1 037,5468	2	-0,003822	-3,68	1 629,38	130505
(F)TPAVHASLDK(F)	100 %	KF	73.56	46.301533	54.84	2	519,7822	1 037,5498	2	-0,0009019	-0,8685	742,448	764985
(F)TPAVHASLDK(F)	100 %	KF	73.3	46.290733	56.19	2	519,7817	1 037,5488	2	-0,001902	-1,831	1 333,32	82 598,0
(F)TPAVHASLDK(F)	100 %	KF	73.27	46.260838	55.66	2	519,782	1 037,5493	2	-0,001322	-1,273	1 630,55	158113
(F)TPAVHASLDK(F)	100 %	KF	73.33	46.621532	56.11	2	519,7825	1 037,5505	2	-0,0001419	-0,1367	735,454	80 280,0
(F)TPAVHASLDK(F)	100 %	KF	73.56	46.878857	55.91	2	519,7811	1 037,5476	2	-0,003082	-2,968	1 768,26	198780
(F)TPAVHASLDK(F)	100 %	KF	72.82	46.311295	49.56	2	519,7823	1 037,5501	2	-0,0005419	-0,5218	1 767,09	53 630,0
(F)TPAVHASLDK(F)	100 %	KF	70.6	46.87056	53.2	2	519,7813	1 037,5481	2	-0,002582	-2,486	1 888,51	54 359,0
(F)TPAVHASLDK(F)	100 %	KF	65.88	46.312004	49.18	2	519,7818	1 037,5490	2	-0,001682	-1,619	1 889,51	101017
(F)TPAVHASLDK(F)	100 %	KF	65.6	46.18895	47.27	2	519,7803	1 037,5460	2	-0,004702	-4,527	1 769,59	42 463,0
(F)TPAVHASLDK(F)	100 %	KF	64.5	46.311295	47.72	2	519,7823	1 037,5500	2	-0,0007019	-0,6759	1 136,42	148765
(F)TPAVHASLDK(F)	100 %	KF	64.4	46.343464	47.31	2	519,7839	1 037,5532	2	0,002538	2,444	686,433	55 009,0
(F)TPAVHASLDK(F)	100 %	KF	64.29	46.344772	47.17	2	519,7835	1 037,5525	2	0,001838	1,77	710,936	79 362,0
(F)TPAVHASLDK(F)	100 %	KF	63.63	46.86779	47.53	2	519,7809	1 037,5473	2	-0,003342	-3,218	1 627,13	37 365,0
(F)TPAVHASLDK(F)	100 %	KF	62.88	46.621532	38.75	2	519,7824	1 037,5503	2	-0,0003819	-0,3678	1 669,05	110355
(F)TPAVHASLDK(F)	100 %	KF	64.01	50.7558	47.99	2	519,7815	1 037,5485	2	-0,002182	-2,101	1 629,88	46 299,0
(F)TPAVHASLDK(F)	100 %	KF	59.57	46.87056	39.47	2	519,7813	1 037,5481	2	-0,002602	-2,505	742,448	83 492,0
(F)TPAVHASLDK(F)	100 %	KF	62.95	50.7064	43.13	2	519,7819	1 037,5492	2	-0,001462	-1,408	1 137,76	65 217,0
(F)TPAVHASLDK(F)	100 %	KF	57.81	46.87056	40.41	2	519,7814	1 037,5482	2	-0,002502	-2,409	1 792,74	113765
(F)TPAVHASLDK(F)	100 %	KF	57.76	46.867615	42.02	2	519,7808	1 037,5471	2	-0,003602	-3,468	1 817,37	15 610,0
(F)TPAVHASLDK(F)	100 %	KF	57.22	46.343464	41.84	2	519,784	1 037,5534	2	0,002698	2,598	684,103	24 033,0
(F)TPAVHASLDK(F)	99 %	KF	57.01	50.77513	35.75	2	519,7825	1 037,5505	2	-0,0001619	-0,1559	714,929	40 318,0
(F)TPAVHASLDK(F)	99 %	KF	51.97	46.18895	39.64	2	519,7803	1 037,5461	2	-0,004562	-4,393	1 793,99	16 646,0
(F)TPAVHASLDK(F)	98 %	KF	50.12	46.182365	33.87	2	519,7798	1 037,5451	2	-0,005602	-5,394	1 162,29	13 835,0
(F)TPAVHASLDK(F)	98 %	KF	49.21	46.54446	32.89	2	519,7794	1 037,5442	2	-0,006502	-6,261	1 655,13	13 461,0
(F)TPAVHASLDK(F)	98 %	KF	48.68	46.865734	33.88	2	519,7807	1 037,5469	2	-0,003802	-3,661	1 889,51	20 392,0
(F)TPAVHASLDK(F)	94 %	KF	42.3	46.260838	12.43	2	519,782	1 037,5494	2	-0,001222	-1,177	1 722,97	16 287,0

(F)TPAVHASLDK(F)	92 %	KF	40.85	46.442024	20.86	2	519,7824	1 037,5502	2	-0,0004419	-0,4255	1 723,97	17 519,0
(F)TPAVHASLDKF(L)	100 %	KF	68.39	47.614918	47.23	2	593,3145	1 184,6144	2	-0,004642	-3,915	1 877,84	61 415,0
(F)TPAVHASLDKF(L)	99 %	KF	57.45	47.427486	34.11	2	593,3153	1 184,6160	2	-0,003082	-2,599	1 876,75	39 727,0
(T)PAVHASLDK(F)	100 %	KF	73.22	46.439457	38.77	2	469,259	936,5034	2	0,0003981	0,4246	1 160,88	16 803,0
(T)PAVHASLDK(F)	100 %	KF	76.17	52.3453	42.14	2	469,2581	936,5015	2	-0,001422	-1,517	1 629,38	46 761,0
(T)PAVHASLDK(F)	100 %	KF	65.92	45.815285	36.48	2	469,2573	936,4999	2	-0,003022	-3,223	1 332,07	37 742,0
(T)PAVHASLDK(F)	100 %	KF	66.2	46.575626	34.75	2	469,2576	936,5007	2	-0,002302	-2,455	1 823,03	48 994,0
(T)PAVHASLDK(F)	100 %	KF	65.97	46.5583	36.05	2	469,2582	936,5018	2	-0,001202	-1,282	1 767,17	123338
(T)PAVHASLDK(F)	100 %	KF	65.93	46.5583	35.87	2	469,2583	936,502	2	-0,0009219	-0,9834	1 141,00	127117
(T)PAVHASLDK(F)	100 %	KF	65.9	46.575626	35.99	2	469,2577	936,5008	2	-0,002182	-2,327	1 334,33	121745
(T)PAVHASLDK(F)	100 %	KF	65.81	46.57572	35.84	2	469,258	936,5014	2	-0,001542	-1,645	1 631,72	130288
(T)PAVHASLDK(F)	100 %	KF	65.43	46.57572	36.37	2	469,2581	936,5015	2	-0,001422	-1,517	1 723,89	23 474,0
(T)PAVHASLDK(F)	100 %	KF	64.32	46.5583	33.28	2	469,2582	936,5019	2	-0,001062	-1,133	742,448	112032
(T)PAVHASLDK(F)	100 %	KF	61.83	46.5583	35.58	2	469,2583	936,502	2	-0,0009219	-0,9834	1 888,34	73 552,0
(T)PAVHASLDK(F)	100 %	KF	61.11	46.557335	34.05	2	469,2588	936,5029	2	-0,00002194	-0,0234	1 160,80	44 570,0
(T)PAVHASLDK(F)	100 %	KF	66.09	52.34287	35.89	2	469,258	936,5015	2	-0,001482	-1,581	1 673,79	253243
(T)PAVHASLDK(F)	100 %	KF	66.0	52.28318	35.98	2	469,2579	936,5013	2	-0,001642	-1,751	1 632,88	177802
(T)PAVHASLDK(F)	100 %	KF	65.98	52.283363	35.87	2	469,2579	936,5013	2	-0,001662	-1,773	1 770,60	227992
(T)PAVHASLDK(F)	100 %	KF	66.04	52.344894	36.0	2	469,2582	936,5018	2	-0,001182	-1,261	1 136,42	324267
(T)PAVHASLDK(F)	100 %	KF	65.84	52.21511	35.8	2	469,2586	936,5027	2	-0,0002619	-0,2794	1 136,34	191080
(T)PAVHASLDK(F)	100 %	KF	65.98	52.36111	35.98	2	469,2583	936,502	2	-0,001002	-1,069	1 139,83	124057
(T)PAVHASLDK(F)	100 %	KF	65.97	52.36023	36.0	2	469,2582	936,5018	2	-0,001122	-1,197	1 888,51	109586
(T)PAVHASLDK(F)	100 %	KF	60.16	46.5583	33.34	2	469,2583	936,5021	2	-0,0008419	-0,8981	1 792,74	244606
(T)PAVHASLDK(F)	100 %	KF	60.12	46.57572	33.32	2	469,2581	936,5016	2	-0,001342	-1,431	1 136,34	553812
(T)PAVHASLDK(F)	100 %	KF	60.05	46.557335	33.49	2	469,2587	936,5028	2	-0,0001819	-0,1941	1 889,51	200883
(T)PAVHASLDK(F)	100 %	KF	60.05	46.575626	33.37	2	469,2578	936,5011	2	-0,001862	-1,986	1 768,26	372710
(T)PAVHASLDK(F)	100 %	KF	59.98	46.5583	33.38	2	469,2581	936,5017	2	-0,001242	-1,325	1 669,05	221320
(T)PAVHASLDK(F)	100 %	KF	59.55	46.575626	33.5	2	469,2577	936,5009	2	-0,002042	-2,178	1 129,35	40 924,0
(T)PAVHASLDK(F)	100 %	KF	58.34	46.57572	34.0	2	469,2581	936,5016	2	-0,001362	-1,453	1 914,27	18 833,0
(T)PAVHASLDK(F)	100 %	KF	57.7	46.6404	33.64	2	469,2594	936,5042	2	0,001258	1,342	1 334,33	13 982,0
(T)PAVHASLDK(F)	99 %	KF	56.98	46.575626	23.91	2	469,2579	936,5012	2	-0,001762	-1,879	1 656,21	33 575,0
(T)PAVHASLDK(F)	99 %	KF	56.06	46.557335	26.0	2	469,2587	936,5028	2	-0,0001419	-0,1514	1 798,56	125110
(T)PAVHASLDK(F)	99 %	KF	60.13	52.318058	34.74	2	469,2567	936,4988	2	-0,004202	-4,482	1 139,92	43 393,0
(T)PAVHASLDK(F)	99 %	KF	60.05	52.284004	33.25	2	469,2579	936,5012	2	-0,001782	-1,901	1 138,67	436899
(T)PAVHASLDK(F)	99 %	KF	53.82	46.28542	25.76	2	469,2563	936,498	2	-0,004982	-5,314	1 817,54	24 568,0
(T)PAVHASLDK(F)	99 %	KF	59.87	52.361816	33.31	2	469,2583	936,502	2	-0,0009219	-0,9834	1 138,75	160699
(T)PAVHASLDK(F)	99 %	KF	59.61	52.216537	33.13	2	469,2587	936,5029	2	-0,0001019	-0,1087	1 890,68	22 760,0
(T)PAVHASLDK(F)	99 %	KF	53.84	46.5583	24.81	2	469,2583	936,502	2	-0,0009219	-0,9834	1 615,37	29 994,0
(T)PAVHASLDK(F)	99 %	KF	59.37	52.34074	29.71	2	469,2581	936,5017	2	-0,001262	-1,346	1 768,34	120175
(T)PAVHASLDK(F)	99 %	KF	53.16	46.575626	32.4	2	469,2576	936,5006	2	-0,002382	-2,541	1 673,71	79 481,0
(T)PAVHASLDK(F)	99 %	KF	58.61	52.206627	35.77	2	469,2576	936,5006	2	-0,002382	-2,541	1 129,43	30 838,0
(T)PAVHASLDK(F)	99 %	KF	58.51	52.220554	25.02	2	469,2587	936,5028	2	-0,0002019	-0,2154	1 767,25	75 692,0
(T)PAVHASLDK(F)	99 %	KF	52.26	46.575626	23.25	2	469,2579	936,5013	2	-0,001642	-1,751	1 333,49	19 752,0
(T)PAVHASLDK(F)	99 %	KF	57.93	52.36124	34.32	2	469,2583	936,502	2	-0,0009619	-1,026	1 615,87	17 644,0
(T)PAVHASLDK(F)	99 %	KF	51.43	46.575626	22.28	2	469,2576	936,5006	2	-0,002402	-2,562	1 628,22	46 875,0
(T)PAVHASLDK(F)	98 %	KF	49.84	46.575626	20.15	2	469,2579	936,5012	2	-0,001782	-1,901	1 628,22	158739
(T)PAVHASLDK(F)	98 %	KF	54.61	52.344917	24.9	2	469,2582	936,5018	2	-0,001162	-1,239	1 638,71	45 899,0

(T)PAVHASLDK(F)	98 %	KF	48.46	46.5583	29.67	2	469,2582	936,5019	2	-0,001102	-1,175	1 768,26	34 642,0
(T)PAVHASLDK(F)	97 %	KF	52.55	52.30017	29.41	2	469,2578	936,501	2	-0,001942	-2,071	1 655,38	24 055,0
(T)PAVHASLDK(F)	97 %	KF	46.33	46.575626	17.29	2	469,2574	936,5003	2	-0,002642	-2,818	1 580,35	21 606,0
(T)PAVHASLDK(F)	97 %	KF	51.76	52.3453	22.47	2	469,2581	936,5016	2	-0,001322	-1,41	1 636,63	23 288,0
(T)PAVHASLDK(F)	97 %	KF	45.98	46.57572	25.75	2	469,2581	936,5016	2	-0,001342	-1,431	1 643,37	21 221,0
(T)PAVHASLDK(F)	97 %	KF	45.95	46.5583	25.04	2	469,2585	936,5024	2	-0,0005819	-0,6207	1 889,67	17 070,0
(T)PAVHASLDK(F)	96 %	KF	45.64	46.575626	23.11	2	469,2576	936,5007	2	-0,002262	-2,413	1 694,88	25 522,0
(T)PAVHASLDK(F)	96 %	KF	51.14	52.12193	22.69	2	469,2572	936,4999	2	-0,003102	-3,309	1 136,42	93 068,0
(T)PAVHASLDK(F)	96 %	KF	45.21	46.575626	17.27	2	469,2579	936,5012	2	-0,001802	-1,922	1 386,94	21 212,0
(T)PAVHASLDK(F)	96 %	KF	44.34	45.8304	20.93	2	469,2571	936,4997	2	-0,003242	-3,458	1 719,55	16 404,0
(T)PAVHASLDK(F)	96 %	KF	44.41	46.28542	20.02	2	469,2563	936,498	2	-0,004982	-5,314	1 847,52	15 751,0
(T)PAVHASLDK(F)	95 %	KF	43.47	46.5583	19.55	2	937,5092	936,5019	1	-0,001086	-1,158	1 138,75	2 427,00
(T)PAVHASLDK(F)	94 %	KF	42.53	46.28542	23.89	2	937,5053	936,498	1	-0,004996	-5,329	1 771,09	12 482,0
(T)PAVHASLDK(F)	93 %	KF	42.1	46.57572	23.43	2	469,258	936,5015	2	-0,001462	-1,559	701,931	37 983,0
(T)PAVHASLDK(F)	93 %	KF	47.56	52.310566	22.61	2	469,2574	936,5003	2	-0,002642	-2,818	687,766	37 263,0
(T)PAVHASLDK(F)	92 %	KF	46.77	52.31658	17.82	2	469,2567	936,4989	2	-0,004082	-4,354	1 844,10	22 446,0
(T)PAVHASLDK(F)	92 %	KF	40.7	46.28542	23.48	2	469,2565	936,4984	2	-0,004582	-4,887	1 605,10	15 178,0
(T)PAVHASLDK(F)	91 %	KF	46.37	52.283363	29.86	2	469,2579	936,5013	2	-0,001662	-1,773	1 796,32	16 565,0
(P)AVHASLDK(F)	99 %	KF	54.7	45.280937	32.32	2	420,7306	839,4467	2	-0,003442	-4,095	1 670,21	18 270,0
(P)AVHASLDK(F)	99 %	KF	52.42	45.280937	33.25	2	420,7307	839,4469	2	-0,003282	-3,905	743,617	17 237,0
(P)AVHASLDK(F)	99 %	KF	51.87	46.10309	29.01	2	420,7318	839,4489	2	-0,001222	-1,454	1 770,60	29 650,0
(P)AVHASLDK(F)	95 %	KF	42.87	45.280937	17.16	2	420,7308	839,4471	2	-0,003062	-3,643	1 136,59	48 189,0
(P)AVHASLDK(F)	95 %	KF	42.86	45.280937	22.49	2	420,7308	839,447	2	-0,003162	-3,762	1 768,34	22 414,0
(P)AVHASLDK(F)	95 %	KF	42.13	45.280937	18.88	2	420,731	839,4474	2	-0,002782	-3,31	1 632,88	23 649,0
(P)AVHASLDK(F)	94 %	KF	41.34	45.280937	21.69	2	420,7316	839,4487	2	-0,001502	-1,787	1 333,32	11 819,0
**Beta-subunit**													
(M)VHLTPEEK(S)	99 %	KS	52.82	46.463055	17.66	2	476,7587	951,5028	2	0,0001781	0,1869	547,288	96 723,0
(M)VHLTPEEK(S)	99 %	KS	52.03	46.06102	16.58	2	476,7589	951,5033	2	0,0006181	0,6489	679,437	233143
(M)VHLTPEEK(S)	99 %	KS	52.02	46.06102	12.09	2	476,7589	951,5033	2	0,0006381	0,6699	630,408	171310
(M)VHLTPEEK(S)	98 %	KS	49.36	46.569504	14.12	2	476,7577	951,5008	2	-0,001822	-1,913	628,155	56 052,0
(M)VHLTPEEK(S)	98 %	KS	53.33	50.56756	13.3	2	476,759	951,5034	2	0,0006981	0,7329	655,006	673246
(M)VHLTPEEK(S)	98 %	KS	48.11	46.535793	7.99	2	476,7583	951,5021	2	-0,0005819	-0,611	682,937	233996
(M)VHLTPEEK(S)	97 %	KS	52.04	50.79503	12.03	2	476,7581	951,5017	2	-0,0009619	-1,01	684,187	100308
(M)VHLTPEEK(S)	97 %	KS	46.67	46.06102	16.09	2	476,7589	951,5033	2	0,0006381	0,6699	542,632	34 937,0
(M)VHLTPEEK(S)	96 %	KS	45.64	46.459038	5.71	2	476,7586	951,5027	2	0,00003806	0,03996	657,257	65 141,0
(M)VHLTPEEK(S)	96 %	KS	49.03	50.72573	13.95	2	476,7586	951,5026	2	-0,0001019	-0,107	561,262	32 256,0
(M)VHLTPEEK(S)	94 %	KS	42.62	46.06102	8.15	2	476,7588	951,5031	2	0,0004581	0,4809	532,169	52 899,0
(M)VHLTPEEK(S)	94 %	KS	42.71	46.33408	2.83	2	476,759	951,5035	2	0,0008581	0,9008	541,469	246751
(M)VHLTPEEK(S)	94 %	KS	42.62	46.459038	7.34	2	476,7587	951,5028	2	0,00009806	0,1029	544,962	177644
(M)VHLTPEEK(S)	93 %	KS	42.01	46.459038	7.98	2	476,7586	951,5026	2	-0,0001019	-0,107	576,415	29 466,0
(M)VHLTPEEK(S)	91 %	KS	44.5	50.56756	9.65	2	476,759	951,5034	2	0,0007381	0,7749	637,413	56 093,0
(M)VHLTPEEK(S)	91 %	KS	40.15	46.541183	11.34	2	476,7576	951,5007	2	-0,002002	-2,102	637,413	25 402,0
(M)VHLTPEEKSAVTALWGK(V)	100 %	KV	134.95	47.52041	119.6	2	622,676	1 865,0062	3	0,001302	0,6978	1 753,15	301231
(M)VHLTPEEKSAVTALWGK(V)	100 %	KV	128.61	47.53958	117.33	2	933,5096	1 865,0047	2	-0,0001619	-0,08678	1 750,73	459402
(M)VHLTPEEKSAVTALWGK(V)	100 %	KV	124.19	47.4164	95.34	2	622,6773	1 865,0099	3	0,005052	2,707	1 750,73	609778
(M)VHLTPEEKSAVTALWGK(V)	100 %	KV	121.66	53.02716	111.2	2	933,5102	1 865,0059	2	0,001058	0,567	1 753,07	274535

(M)VHLTPEEKSAVTALWGK(V)	100 %	KV	101.4	53.021946	90.92	2	933,5102	1 865,0057	2	0,0008781	0,4706	1 750,82	333436
(M)VHLTPEEKSAVTALWGK(V)	100 %	KV	95.25	47.52041	86.62	2	622,676	1 865,0062	3	0,001302	0,6978	1 757,74	87 667,0
(M)VHLTPEEKSAVTALWGK(V)	100 %	KV	82.04	47.409782	71.89	2	622,6773	1 865,0102	3	0,005322	2,852	1 748,48	66 970,0
(M)VHLTPEEKSAVTALWGK(V)	100 %	KV	78.42	53.092552	69.44	2	933,5097	1 865,0047	2	-0,0001219	-0,06535	1 751,90	132771
(M)VHLTPEEKSAVTALWGK(V)	100 %	KV	71.11	52.998405	59.81	2	933,5104	1 865,0062	2	0,001378	0,7385	1 751,98	72 896,0
(M)VHLTPEEKSAVTALWGK(V)	98 %	KV	51.41	47.277283	45.35	2	933,5115	1 865,0084	2	0,003538	1,896	1 756,74	522147
(M)VHLTPEEKSAVTALWGK(V)	96 %	KV	45.86	47.594486	39.75	2	933,5082	1 865,0018	2	-0,003042	-1,63	1 748,48	41 888,0
(M)VHLTPEEKSAVTALWGKVN(V)	100 %	NV	100.97	48.18384	84.76	2	1 040,0642	2 078,1138	2	-0,002362	-1,136	1 845,18	136583
(M)VHLTPEEKSAVTALWGKVN(V)	100 %	NV	99.44	48.03587	87.98	2	693,7121	2 078,1145	3	-0,001638	-0,7878	1 846,35	129321
(M)VHLTPEEKSAVTALWGKVN(V)	100 %	NV	99.19	48.026234	86.09	2	693,712	2 078,1143	3	-0,001908	-0,9177	1 895,35	66 553,0
(M)VHLTPEEKSAVTALWGKVN(V)	100 %	NV	86.87	48.024178	73.86	2	693,7123	2 078,1150	3	-0,001158	-0,5569	1 870,84	84 283,0
(M)VHLTPEEKSAVTALWGKVN(V)	100 %	NV	81.79	48.031498	69.78	2	693,7131	2 078,1174	3	0,001212	0,583	1 845,27	55 718,0
(M)VHLTPEEKSAVTALWGKVN(V)	100 %	NV	59.95	47.933155	54.05	2	693,7129	2 078,1167	3	0,0005521	0,2655	1 847,52	43 843,0
(M)VHLTPEEKSAVTALWGKVN(V)	99 %	NV	52.64	47.8963	43.11	2	693,7135	2 078,1186	3	0,002412	1,16	1 846,35	27 842,0
(M)VHLTPEEKSAVTALWGKVN(V)	94 %	NV	44.42	47.94021	40.11	2	693,7127	2 078,1163	3	0,0001621	0,07795	1 919,94	26 886,0
(M)VHLTPEEKSAVTALWGKVN(V)	93 %	NV	43.75	48.30544	36.61	2	693,7108	2 078,1107	3	-0,005508	-2,649	1 888,67	24 228,0
(M)VHLTPEEKSAVTALWGKVNV(D)	100 %	VD	104.6	47.53177	95.71	2	726,7353	2 177,1841	3	-0,0004379	-0,2011	2 022,52	37 862,0
(M)VHLTPEEKSAVTALWGKVNV(D)	100 %	VD	97.16	47.448715	87.23	2	726,7365	2 177,1877	3	0,003102	1,424	2 027,18	65 538,0
(M)VHLTPEEKSAVTALWGKVNV(D)	100 %	VD	75.48	47.470154	69.54	2	726,7361	2 177,1866	3	0,002022	0,9283	2 023,69	45 966,0
(M)VHLTPEEKSAVTALWGKVNV(D)	99 %	VD	52.56	47.67223	41.5	2	726,7344	2 177,1812	3	-0,003348	-1,537	1 966,71	25 850,0
(M)VHLTPEEKSAVTALWGKVNV(D)	98 %	VD	51.51	47.894894	45.52	2	726,7331	2 177,1774	3	-0,007218	-3,314	1 968,96	22 730,0
(M)VHLTPEEKSAVTALWGKVNV(D)	97 %	VD	48.54	47.628883	41.02	2	726,7348	2 177,1827	3	-0,001908	-0,8759	2 004,13	22 347,0
(M)VHLTPEEKSAVTALWGKVNVDE(V)	100 %	EV	140.81	49.46909	131.87	2	808,0932	2 421,2577	3	0,003512	1,45	1 972,38	244516
(M)VHLTPEEKSAVTALWGKVNVDE(V)	100 %	EV	120.04	49.46884	111.58	2	808,0931	2 421,2576	3	0,003422	1,413	1 928,02	222897
(M)VHLTPEEKSAVTALWGKVNVDE(V)	100 %	EV	107.1	49.50788	89.77	2	808,0924	2 421,2554	3	0,001232	0,5086	1 968,88	69 454,0
(M)VHLTPEEKSAVTALWGKVNVDE(V)	100 %	EV	103.67	49.467663	90.8	2	808,0932	2 421,2579	3	0,003722	1,537	1 996,89	95 746,0
(M)VHLTPEEKSAVTALWGKVNVDE(V)	100 %	EV	76.66	49.497948	66.69	2	808,0926	2 421,2559	3	0,001772	0,7316	1 925,77	49 771,0
(M)VHLTPEEKSAVTALWGKVNVDE(V)	100 %	EV	71.41	49.499897	53.74	2	808,0924	2 421,2555	3	0,001292	0,5334	1 993,47	39 071,0
(M)VHLTPEEKSAVTALWGKVNVDE(V)	100 %	EV	65.2	49.499702	48.85	2	808,0926	2 421,2560	3	0,001862	0,7687	1 926,93	32 492,0
(M)VHLTPEEKSAVTALWGKVNVDE(V)	100 %	EV	61.85	49.494877	55.48	2	808,0927	2 421,2562	3	0,002012	0,8307	1 952,53	61 885,0
(L)TPEEKSAVTALWGK(V)	100 %	KV	94.82	48.632217	80.29	2	758,9018	1 515,7890	2	-0,004462	-2,942	1 751,90	77 318,0
(L)TPEEKSAVTALWGK(V)	91 %	KV	42.54	48.617615	31.46	2	758,9045	1 515,7943	2	0,0008781	0,5789	1 750,90	35 990,0
(L)TPEEKSAVTALWGKVN(V)	100 %	NV	72.96	48.768814	58.63	2	865,4577	1 728,9009	2	-0,003902	-2,256	1 767,25	31 861,0
(L)TPEEKSAVTALWGKVN(V)	99 %	NV	55.16	48.651043	44.93	2	865,4569	1 728,8992	2	-0,005562	-3,215	1 765,25	39 867,0
(E)EKSAVTALWGKVNVDE(V)	100 %	EV	109.53	49.00657	94.66	2	873,4566	1 744,8986	2	-0,001102	-0,6312	1 835,86	139640
(E)EKSAVTALWGKVNVDE(V)	100 %	EV	85.3	54.76562	69.06	2	873,4567	1 744,8988	2	-0,0008619	-0,4937	1 836,03	130821
(E)KSAVTALWGKVN(V)	100 %	NV	91.78	45.88104	64.14	2	637,3671	1 272,7196	2	0,0005181	0,4067	1 615,45	167248
(E)KSAVTALWGKVN(V)	100 %	NV	85.38	45.447746	65.33	2	637,3689	1 272,7233	2	0,004258	3,343	1 614,37	69 156,0
(E)KSAVTALWGKVNVDE(V)	100 %	EV	60.23	48.30165	47.13	2	808,9372	1 615,8598	2	0,002738	1,693	1 781,41	64 329,0
(E)KSAVTALWGKVNVDE(V)	99 %	EV	57.65	48.36742	42.02	2	808,9338	1 615,8530	2	-0,004082	-2,525	1 781,32	78 509,0
(K)SAVTALWGKVNVDE(V)	100 %	EV	88.49	48.906055	73.56	2	744,8906	1 487,7665	2	0,004478	3,008	1 964,21	440221
(K)SAVTALWGKVNVDE(V)	100 %	EV	78.23	48.86857	58.9	2	744,8901	1 487,7656	2	0,003518	2,363	1 962,04	70 051,0
(K)SAVTALWGKVNVDE(V)	100 %	EV	75.85	48.827576	62.38	2	744,8887	1 487,7628	2	0,0006981	0,4689	1 963,04	145619
(K)SAVTALWGKVNVDE(V)	100 %	EV	73.38	48.768353	60.0	2	744,8897	1 487,7649	2	0,002838	1,906	1 963,20	173760
(K)SAVTALWGKVNVDE(V)	100 %	EV	73.24	48.82906	59.37	2	744,8887	1 487,7628	2	0,0007781	0,5226	1 964,21	120641
(K)SAVTALWGKVNVDE(V)	100 %	EV	66.17	48.80156	51.91	2	744,8881	1 487,7616	2	-0,0005019	-0,3372	1 964,29	98 413,0

(K)SAVTALWGKVNVDE(V)	100 %	EV	65.91	48.82906	47.18	2	744,8888	1 487,7629	2	0,0008781	0,5898	1 963,12	165289
(K)SAVTALWGKVNVDE(V)	100 %	EV	63.97	48.789238	49.67	2	744,8889	1 487,7632	2	0,001118	0,751	1 963,04	178203
(K)SAVTALWGKVNVDE(V)	100 %	EV	62.98	48.879097	44.73	2	744,8901	1 487,7657	2	0,003638	2,444	1 961,95	54 403,0
(K)SAVTALWGKVNVDE(V)	100 %	EV	60.7	48.519978	48.42	2	744,893	1 487,7713	2	0,009278	6,232	1 963,04	546861
(A)VTALWGKVNV(D)	97 %	VD	46.43	46.219753	31.16	2	1 086,6240	1 085,6168	1	-0,006606	-6,079	1 913,02	6 285,00
(A)VTALWGKVNV(D)	97 %	VD	46.65	46.462273	31.21	2	543,8175	1 085,6204	2	-0,002942	-2,707	1 913,02	37 828,0
(A)VTALWGKVNV(D)	92 %	VD	40.58	46.392273	25.37	2	1 086,6293	1 085,6221	1	-0,001316	-1,211	1 913,19	11 095,0
(A)VTALWGKVNVDE(V)	100 %	EV	61.76	48.45594	28.62	2	665,8503	1 329,6861	2	-0,006862	-5,157	1 836,19	97 624,0
(A)VTALWGKVNVDE(V)	99 %	EV	56.39	48.532722	23.11	2	665,8546	1 329,6947	2	0,001758	1,321	1 837,03	180100
(A)VTALWGKVNVDE(V)	92 %	EV	43.16	48.397484	17.52	2	665,8525	1 329,6904	2	-0,002562	-1,925	1 839,36	20 552,0
(A)VTALWGKVNVDEVGGEALGR(L)	100 %	RL	83.19	48.823196	70.57	2	690,703	2 069,0871	3	-0,003658	-1,767	1 931,52	25 752,0
(A)VTALWGKVNVDEVGGEALGR(L)	100 %	RL	64.56	48.556587	50.51	2	1 035,5557	2 069,0968	2	0,006038	2,917	1 930,69	54 343,0
(A)VTALWGKVNVDEVGGEALGR(L)	99 %	RL	55.2	48.779068	50.53	2	1 035,5533	2 069,0919	2	0,001178	0,5691	1 931,52	43 001,0
(T)ALWGKVNVDE(V)	99 %	EV	52.53	48.017124	27.14	2	1 130,5813	1 129,5740	1	-0,002886	-2,553	1 704,14	16 507,0
(T)ALWGKVNVDE(V)	97 %	EV	48.11	48.01815	28.59	2	1 130,5810	1 129,5737	1	-0,003136	-2,774	1 704,06	13 005,0
(T)ALWGKVNVDE(V)	96 %	EV	46.55	48.01815	25.01	2	1 130,5811	1 129,5738	1	-0,003056	-2,703	1 707,55	5 782,00
(T)ALWGKVNVDE(V)	94 %	EV	43.97	48.023422	27.34	2	1 130,5803	1 129,5730	1	-0,003876	-3,428	1 706,39	12 079,0
(T)ALWGKVNVDE(V)	91 %	EV	41.93	48.12385	31.94	2	565,7956	1 129,5765	2	-0,0003219	-0,2848	1 704,39	29 756,0
(T)ALWGKVNVDEVGGEA(L)	100 %	AL	84.57	48.65536	58.96	2	772,3915	1 542,7685	2	0,0005181	0,3356	1 839,36	126806
(T)ALWGKVNVDEVGGEA(L)	99 %	AL	54.98	48.4822	41.13	2	772,3924	1 542,7703	2	0,002358	1,527	1 838,27	51 839,0
(T)ALWGKVNVDEVGGEALG(R)	100 %	GR	126.51	48.713512	106.34	2	857,4469	1 712,8792	2	0,005598	3,266	1 971,22	360244
(T)ALWGKVNVDEVGGEALG(R)	100 %	GR	117.66	48.826614	101.55	2	857,4456	1 712,8766	2	0,002998	1,749	1 970,05	149106
(T)ALWGKVNVDEVGGEALG(R)	100 %	GR	80.93	54.72917	66.42	2	857,4458	1 712,8770	2	0,003458	2,018	1 971,30	80 692,0
(T)ALWGKVNVDEVGGEALG(R)	92 %	GR	43.19	48.8361	25.72	2	857,4455	1 712,8765	2	0,002898	1,691	1 971,38	20 998,0
(T)ALWGKVNVDEVGGEALGR(L)	100 %	RL	129.0	48.90879	106.32	2	935,494	1 868,9734	2	-0,001242	-0,6641	1 866,17	228196
(T)ALWGKVNVDEVGGEALGR(L)	100 %	RL	125.81	48.788548	101.17	2	935,4955	1 868,9765	2	0,001838	0,9829	1 867,34	537202
(T)ALWGKVNVDEVGGEALGR(L)	100 %	RL	66.76	48.791016	52.61	2	935,4951	1 868,9756	2	0,0008981	0,4802	1 865,26	50 563,0
(A)LWGKVNVDEVGGEA(L)	100 %	AL	98.66	48.58639	76.75	2	736,8723	1 471,7301	2	-0,0007619	-0,5174	1 743,73	120727
(A)LWGKVNVDEVGGEA(L)	100 %	AL	82.12	48.550526	56.3	2	736,8748	1 471,7351	2	0,004238	2,878	1 743,06	172860
(A)LWGKVNVDEVGGEA(L)	100 %	AL	82.69	52.57191	64.22	2	736,8719	1 471,7293	2	-0,001582	-1,074	1 743,98	54 749,0
(A)LWGKVNVDEVGGEALG(R)	100 %	GR	114.52	48.569283	78.62	2	821,9256	1 641,8366	2	0,0001781	0,1084	1 902,50	85 303,0
(A)LWGKVNVDEVGGEALG(R)	100 %	GR	92.65	48.741455	67.4	2	821,9241	1 641,8336	2	-0,002822	-1,718	1 903,51	176676
(A)LWGKVNVDEVGGEALGR(L)	100 %	RL	103.78	48.721157	81.87	2	899,9741	1 797,9335	2	-0,004022	-2,236	1 785,74	112813
(A)LWGKVNVDEVGGEALGR(L)	93 %	RL	44.32	48.606735	31.66	2	899,973	1 797,9314	2	-0,006182	-3,436	1 783,41	44 189,0
(N)VDEVGGEALGR(L)	100 %	RL	76.3	47.497902	49.32	2	551,2792	1 100,5439	2	-0,002442	-2,217	1 051,31	18 017,0
(N)VDEVGGEALGR(L)	100 %	RL	65.0	53.469433	34.44	2	551,2799	1 100,5453	2	-0,001062	-0,964	1 060,61	18 631,0
(N)VDEVGGEALGR(L)	94 %	RL	43.74	47.43549	16.52	2	551,2782	1 100,5419	2	-0,004502	-4,087	1 027,10	26 937,0
(D)EVGGEALGRLLV(V)	100 %	VV	70.37	45.156487	53.33	2	606,851	1 211,6874	2	-0,0001419	-0,117	2 048,14	119667
(D)EVGGEALGRLLV(V)	97 %	VV	45.94	45.156487	32.24	2	606,8503	1 211,6861	2	-0,001442	-1,189	2 046,98	43 319,0
(E)VGGEALGRLLV(V)	99 %	VV	52.92	42.925438	27.22	2	542,3298	1 082,6450	2	0,00007806	0,07203	1 988,71	362176
(E)VGGEALGRLLV(V)	98 %	VV	45.83	42.923225	22.89	2	542,3294	1 082,6442	2	-0,0008019	-0,74	1 987,55	97 730,0
(E)VGGEALGRLLV(V)	95 %	VV	40.13	42.923225	24.0	2	542,3294	1 082,6442	2	-0,0007219	-0,6662	1 989,22	19 509,0
(L)GRLLVVYPWTQ(R)	95 %	QR	44.33	46.589363	30.1	2	666,3785	1 330,7425	2	0,002618	1,966	2 088,86	45 385,0
(L)GRLLVVYPWTQ(R)	91 %	QR	40.73	46.667614	24.24	2	666,3779	1 330,7412	2	0,001318	0,9897	2 087,95	37 568,0
(G)RLLVVYPWT(Q)	91 %	TQ	38.51	44.827023	21.59	2	573,8361	1 145,6577	2	-0,002102	-1,833	2 117,12	558817
(G)RLLVVYPWT(Q)	91 %	TQ	38.54	44.88128	21.46	2	573,8361	1 145,6576	2	-0,002142	-1,868	2 117,95	418280

(G)RLLVVYPWTQ(R)	99 %	QR	52.59	45.87284	31.31	2		637,8663	1 273,7181	2	-0,0002619	-0,2055	2 086,53	146009
(G)RLLVVYPWTQ(R)	94 %	QR	42.08	45.438446	12.31	2		637,8686	1 273,7226	2	0,004238	3,325	2 087,70	502190
(W)TQRFFESFGDLSTPDA(V)	100 %	AV	72.95	46.762817	55.34	2		909,4194	1 816,8243	2	-0,002662	-1,464	2 025,10	47 803,0
(W)TQRFFESFGDLSTPDA(V)	97 %	AV	48.0	46.84091	36.71	2		909,4202	1 816,8258	2	-0,001142	-0,6282	2 023,77	16 761,0
(T)QRFFESFGDLSTPDA(V)	100 %	AV	86.47	47.00522	63.37	2		858,8995	1 715,7844	2	0,005178	3,016	2 015,53	416917
(T)QRFFESFGDLSTPDA(V)	100 %	AV	87.79	50.090214	74.38	2		858,8983	1 715,7819	2	0,002678	1,56	2 014,45	248194
(T)QRFFESFGDLSTPDA(V)	100 %	AV	64.51	46.970116	38.29	2		858,8987	1 715,7828	2	0,003498	2,038	2 013,46	80 985,0
(Q)RFFESFGDLSTPDA(V)	100 %	AV	64.26	46.8191	45.57	2		794,8686	1 587,7227	2	0,001998	1,258	2 013,21	192805
(Q)RFFESFGDLSTPDA(V)	97 %	AV	46.6	46.648487	35.08	2		794,8669	1 587,7193	2	-0,001402	-0,8824	2 014,37	25 462,0
(F)ESFGDLSTPDA(V)	100 %	AV	60.73	44.153908	36.35	2		1 138,4850	1 137,4777	1	-0,005046	-4,432	1 790,41	129023
(F)ESFGDLSTPDA(V)	100 %	AV	54.86	43.902992	31.61	2		1 138,4856	1 137,4783	1	-0,004476	-3,931	1 786,91	60 246,0
(F)ESFGDLSTPDA(V)	99 %	AV	52.81	43.78071	31.46	2		1 138,4861	1 137,4788	1	-0,003986	-3,501	1 793,91	20 476,0
(F)ESFGDLSTPDA(V)	99 %	AV	51.12	43.78016	29.41	2		1 138,4861	1 137,4788	1	-0,003946	-3,466	1 790,41	154001
(F)ESFGDLSTPDA(V)	99 %	AV	50.8	43.930313	26.72	2		1 138,4844	1 137,4771	1	-0,005626	-4,942	1 786,08	33 808,0
(F)ESFGDLSTPDA(V)	94 %	AV	40.1	43.902992	20.63	2		1 138,4856	1 137,4783	1	-0,004466	-3,923	1 789,24	101662
(F)ESFGDLSTPDAVMGNPK(V)	100 %	KV	98.12	47.20746	87.28	2		882,9091	1 763,8037	2	-0,0001819	-0,1031	1 855,68	60 127,0
(F)ESFGDLSTPDAVMGNPK(V)	100 %	KV	64.47	47.207954	57.82	2		882,9091	1 763,8037	2	-0,0001419	-0,08043	1 856,85	85 379,0
(F)ESFGDLSTPDAVmGNPK(V)	100 %	KV	59.66	46.406803	42.66	2	Oxidation (+16)	890,9074	1 779,8002	2	0,001443	0,8103	1 678,37	57 560,0
(F)ESFGDLSTPDAVmGNPK(V)	99 %	KV	54.46	46.682648	41.97	2	Oxidation (+16)	890,9087	1 779,8028	2	0,004023	2,259	1 676,21	34 772,0
(F)ESFGDLSTPDAVmGNPKVK(A)	100 %	KA	90.26	48.898956	79.1	2	Oxidation (+16)	1 004,4892	2 006,9639	2	0,001683	0,8382	1 601,35	160564
(F)ESFGDLSTPDAVMGNPKVK(A)	100 %	KA	62.67	49.022804	50.81	2		996,4883	1 990,9620	2	-0,005222	-2,621	1 774,09	109894
(F)ESFGDLSTPDAVMGNPKVK(A)	99 %	KA	53.75	49.087315	52.78	2		996,489	1 990,9633	2	-0,003922	-1,969	1 773,18	71 243,0
(F)ESFGDLSTPDAVmGNPKVK(A)	94 %	KA	45.38	49.21572	45.38	2	Oxidation (+16)	1 004,4938	2 006,9729	2	0,01076	5,36	1 598,34	33 746,0
(F)ESFGDLSTPDAVmGNPKVKAHGKK(V)	100 %	KV	70.5	50.078545	61.91	2	Oxidation (+16)	843,7622	2 528,2648	3	-0,004883	-1,931	1 108,38	129662
(F)ESFGDLSTPDAVMGNPKVKAHGKK(V)	99 %	KV	57.46	49.989304	43.55	2		838,4304	2 512,2692	3	-0,005548	-2,207	1 385,93	62 930,0
(F)ESFGDLSTPDAVmGNPKVKAHGKK(V)	99 %	KV	55.01	50.08285	39.61	2	Oxidation (+16)	843,7636	2 528,2689	3	-0,000803	-0,3175	1 107,21	66 146,0
(F)ESFGDLSTPDAVmGNPKVKAHGKK(V)	98 %	KV	52.16	50.10147	43.76	2	Oxidation (+16)	843,7629	2 528,2667	3	-0,002963	-1,171	1 106,05	43 120,0
(F)ESFGDLSTPDAVMGNPKVKAHGKKV(L)	100 %	VL	102.04	49.852516	83.64	2		871,4559	2 611,3460	3	0,002792	1,069	1 452,02	203767
(F)ESFGDLSTPDAVMGNPKVKAHGKKV(L)	100 %	VL	100.31	49.99683	97.17	2		653,8425	2 611,3407	4	-0,002474	-0,947	1 453,19	214796
(F)ESFGDLSTPDAVmGNPKVKAHGKKV(L)	100 %	VL	90.62	50.22931	77.38	2	Oxidation (+16)	876,7872	2 627,3397	3	0,001657	0,6305	1 145,74	184320
(F)ESFGDLSTPDAVMGNPKVKAHGKKV(L)	98 %	VL	53.64	49.73659	40.11	2		871,459	2 611,3552	3	0,01203	4,606	1 451,11	29 505,0
(F)ESFGDLSTPDAVMGNPKVKAHGKKV(L)	95 %	VL	47.44	49.852604	36.54	2		871,4559	2 611,3458	3	0,002672	1,023	1 450,94	27 158,0
(F)ESFGDLSTPDAVmGNPKVKAHGKKV(L)	95 %	VL	47.37	50.066284	32.67	2	Oxidation (+16)	876,7887	2 627,3442	3	0,006067	2,308	1 143,66	31 265,0
(F)ESFGDLSTPDAVmGNPKVKAHGKKVLG(A)	100 %	GA	103.53	50.172672	94.85	2	Oxidation (+16)	933,4877	2 797,4412	3	-0,002473	-0,8837	1 370,54	192292
(F)ESFGDLSTPDAVmGNPKVKAHGKKVLG(A)	100 %	GA	100.16	49.90348	97.85	2	Oxidation (+16)	700,3717	2 797,4575	4	0,01381	4,935	1 369,29	383339
(F)ESFGDLSTPDAVMGNPKVKAHGKKVLG(A)	100 %	GA	93.03	49.56845	89.84	2		696,3719	2 781,4587	4	0,009886	3,553	1 614,20	151335
(F)ESFGDLSTPDAVMGNPKVKAHGKKVLG(A)	100 %	GA	76.07	49.786873	69.58	2		928,157	2 781,4493	3	0,0005221	0,1876	1 616,53	213104
(F)ESFGDLSTPDAVmGNPKVKAHGKKVLG(A)	100 %	GA	75.83	50.068935	67.81	2	Oxidation (+16)	933,4899	2 797,4478	3	0,004067	1,453	1 370,46	229690
(F)ESFGDLSTPDAVmGNPKVKAHGKKVLG(A)	100 %	GA	74.91	50.174255	66.92	2	Oxidation (+16)	700,3676	2 797,4413	4	-0,002389	-0,8537	1 373,95	71 300,0
(F)ESFGDLSTPDAVmGNPKVKAHGKKVLG(A)	100 %	GA	77.03	54.76356	72.17	2	Oxidation (+16)	933,4907	2 797,4502	3	0,006497	2,322	1 373,95	306208
(F)ESFGDLSTPDAVmGNPKVKAHGKKVLG(A)	100 %	GA	71.59	50.019974	68.08	2	Oxidation (+16)	700,3701	2 797,4512	4	0,007491	2,677	1 365,88	112923
(F)ESFGDLSTPDAVMGNPKVKAHGKKVLG(A)	99 %	GA	64.19	53.812107	62.25	2		696,3717	2 781,4576	4	0,008806	3,165	1 683,54	117881
(F)ESFGDLSTPDAVmGNPKVKAHGKKVLG(A)	98 %	GA	52.9	50.09277	47.25	2	Oxidation (+16)	933,4894	2 797,4463	3	0,002627	0,9388	1 368,21	31 269,0
(F)ESFGDLSTPDAVMGNPKVKAHGKKVLG(A)	97 %	GA	50.36	49.864136	41.83	2		928,1556	2 781,4450	3	-0,003768	-1,354	1 614,28	58 512,0
(F)ESFGDLSTPDAVmGNPKVKAHGKKVLG(A)	96 %	GA	49.03	50.226715	41.38	2	Oxidation (+16)	933,4866	2 797,4379	3	-0,005743	-2,052	1 365,80	59 935,0
(F)ESFGDLSTPDAVmGNPKVKAHGKKVLG(A)	95 %	GA	46.78	49.944054	42.45	2	Oxidation (+16)	700,3708	2 797,4542	4	0,01053	3,763	1 367,46	51 637,0

(F)ESFGDLSTPDAVmGNPKVKAHGKKVLG(A)	94 %	GA	46.36	50.0659	35.83	2	Oxidation (+16)	933,4896	2 797,4471	3	0,003407	1,217	1 379,76	96 435,0
(F)ESFGDLSTPDAVMGNPKVKAHGKKVLG(A)	93 %	GA	45.13	49.75818	44.0	2		696,3699	2 781,4505	4	0,001686	0,606	1 613,03	40 028,0
(E)SFGDLSTPDA(V)	100 %	AV	59.82	45.24941	37.08	2		1 009,4465	1 008,4392	1	-0,0009759	-0,9668	1 747,24	190048
(E)SFGDLSTPDA(V)	97 %	AV	46.65	45.51023	21.86	2		1 009,4441	1 008,4368	1	-0,003356	-3,325	1 747,24	198514
(E)SFGDLSTPDA(V)	97 %	AV	47.02	46.05919	23.24	2		1 009,4512	1 008,4439	1	0,003754	3,719	1 744,89	167845
(E)SFGDLSTPDA(V)	93 %	AV	41.31	45.69643	20.44	2		1 009,4471	1 008,4398	1	-0,0003359	-0,3328	1 746,07	184359
(E)SFGDLSTPDAVMGNPK(V)	100 %	KV	85.89	47.92707	69.66	2		818,3912	1 634,7679	2	0,006638	4,058	1 834,78	92 392,0
(E)SFGDLSTPDAVMGNPK(V)	97 %	KV	47.96	48.092228	34.56	2		818,3931	1 634,7716	2	0,01034	6,32	1 834,03	36 606,0
(E)SFGDLSTPDAVmGNPKVK(A)	100 %	KA	112.82	49.079216	95.2	2	Oxidation (+16)	939,967	1 877,9194	2	-0,000137	-0,07289	1 551,22	284584
(E)SFGDLSTPDAVMGNPKVK(A)	100 %	KA	104.74	53.374054	88.62	2		931,9732	1 861,9317	2	0,007078	3,799	1 756,66	1948620
(E)SFGDLSTPDAVmGNPKVK(A)	100 %	KA	83.35	49.19303	74.48	2	Oxidation (+16)	626,9808	1 877,9206	3	0,000987	0,5253	1 551,22	73 871,0
(E)SFGDLSTPDAVMGNPKVK(A)	100 %	KA	76.38	49.217644	71.08	2		621,6479	1 861,9219	3	-0,002798	-1,502	1 755,49	249415
(E)SFGDLSTPDAVMGNPKVK(A)	100 %	KA	68.09	49.26476	50.17	2		931,972	1 861,9294	2	0,004698	2,522	1 754,23	427303
(E)SFGDLSTPDAVmGNPKVK(A)	100 %	KA	65.16	49.066044	51.33	2	Oxidation (+16)	939,9654	1 877,9163	2	-0,003277	-1,744	1 548,89	37 629,0
(E)SFGDLSTPDAVMGNPKVK(A)	99 %	KA	53.99	49.190468	46.45	2		621,6477	1 861,9213	3	-0,003398	-1,824	1 756,66	277056
(E)SFGDLSTPDAVMGNPKVKA(H)	100 %	AH	61.07	49.319405	50.87	2		967,4845	1 932,9544	2	-0,007402	-3,827	1 774,59	59 909,0
(E)SFGDLSTPDAVmGNPKVKA(H)	99 %	AH	54.99	49.253227	48.7	2	Oxidation (+16)	650,6595	1 948,9567	3	0,00001705	0,008744	1 604,94	32 887,0
(E)SFGDLSTPDAVMGNPKVKA(H)	99 %	AH	54.03	49.264606	45.59	2		967,4864	1 932,9582	2	-0,003522	-1,821	1 775,26	37 287,0
(E)SFGDLSTPDAVMGNPKVKAHGKK(V)	100 %	KV	113.2	49.445763	89.05	2		795,4199	2 383,2378	3	0,005612	2,354	1 322,65	303954
(E)SFGDLSTPDAVMGNPKVKAHGKK(V)	100 %	KV	94.71	49.667095	89.64	2		596,8139	2 383,2264	4	-0,005754	-2,413	1 322,65	264840
(E)SFGDLSTPDAVMGNPKVKAHGKK(V)	100 %	KV	92.5	49.365288	76.01	2		795,4223	2 383,2451	3	0,0129	5,411	1 324,98	850186
(E)SFGDLSTPDAVMGNPKVKAHGKK(V)	100 %	KV	87.68	49.48266	80.56	2		596,8152	2 383,2316	4	-0,0005939	-0,2491	1 324,98	648098
(E)SFGDLSTPDAVMGNPKVKAHGKK(V)	100 %	KV	73.93	49.443546	68.91	2		1 192,6252	2 383,2357	2	0,003578	1,501	1 331,99	251239
(E)SFGDLSTPDAVmGNPKVKAHGKK(V)	100 %	KV	72.24	49.743828	60.4	2	Oxidation (+16)	800,7516	2 399,2331	3	0,005977	2,49	1 021,02	259162
(E)SFGDLSTPDAVMGNPKVKAHGKK(V)	100 %	KV	69.74	49.723137	68.79	2		596,813	2 383,2230	4	-0,009154	-3,839	1 321,99	198876
(E)SFGDLSTPDAVMGNPKVKAHGKK(V)	99 %	KV	62.47	54.314426	52.34	2		795,4199	2 383,2379	3	0,005702	2,392	1 320,41	57 856,0
(E)SFGDLSTPDAVMGNPKVKAHGKK(V)	99 %	KV	57.18	49.478992	40.31	2		795,4194	2 383,2363	3	0,004142	1,737	1 360,23	60 376,0
(E)SFGDLSTPDAVmGNPKVKAHGKK(V)	99 %	KV	56.14	49.823933	52.97	2	Oxidation (+16)	600,8141	2 399,2272	4	0,00009105	0,03793	1 021,02	106727
(E)SFGDLSTPDAVMGNPKVKAHGKK(V)	99 %	KV	54.15	49.411488	48.71	2		596,8176	2 383,2411	4	0,008926	3,744	1 356,74	25 267,0
(E)SFGDLSTPDAVMGNPKVKAHGKK(V)	97 %	KV	50.11	49.52744	44.42	2		1 192,6244	2 383,2342	2	0,002018	0,8464	1 334,33	80 020,0
(E)SFGDLSTPDAVMGNPKVKAHGKKV(L)	100 %	VL	119.74	49.05105	108.36	2		828,4438	2 482,3097	3	0,009092	3,661	1 366,97	403406
(E)SFGDLSTPDAVMGNPKVKAHGKKV(L)	100 %	VL	94.31	49.3222	87.58	2		621,5821	2 482,2992	4	-0,001354	-0,5452	1 365,80	235776
(E)SFGDLSTPDAVMGNPKVKAHGKKV(L)	100 %	VL	73.14	49.176212	62.89	2		828,4426	2 482,3061	3	0,005492	2,212	1 364,64	116485
(E)SFGDLSTPDAVMGNPKVKAHGKKV(L)	99 %	VL	55.0	49.084686	42.68	2		828,4435	2 482,3085	3	0,007952	3,202	1 365,88	55 960,0
(E)SFGDLSTPDAVMGNPKVKAHGKKV(L)	97 %	VL	54.92	54.030518	41.83	2		828,4421	2 482,3046	3	0,003992	1,608	1 363,64	79 112,0
(E)SFGDLSTPDAVMGNPKVKAHGKKV(L)	97 %	VL	49.88	49.12142	38.24	2		828,4426	2 482,3058	3	0,005252	2,115	1 379,76	90 065,0
(E)SFGDLSTPDAVmGNPKVKAHGKKV(L)	91 %	VL	43.45	49.723236	29.59	2	Oxidation (+16)	833,771	2 498,2910	3	-0,004463	-1,786	1 080,51	74 633,0
(E)SFGDLSTPDAVmGNPKVKAHGKKVL(G)	100 %	LG	102.46	48.945045	92.53	2	Oxidation (+16)	871,4711	2 611,3914	3	0,0118	4,516	1 322,65	595208
(E)SFGDLSTPDAVMGNPKVKAHGKKVL(G)	100 %	LG	81.23	48.65536	79.39	2		649,8546	2 595,3892	4	0,004546	1,751	1 684,29	48 171,0
(E)SFGDLSTPDAVMGNPKVKAHGKKVL(G)	100 %	LG	80.91	48.725	79.17	2		649,8535	2 595,3849	4	0,0002661	0,1025	1 610,69	273803
(E)SFGDLSTPDAVmGNPKVKAHGKKVL(G)	100 %	LG	77.6	48.945045	66.51	2	Oxidation (+16)	871,4711	2 611,3914	3	0,0118	4,516	1 323,82	306094
(E)SFGDLSTPDAVmGNPKVKAHGKKVL(G)	100 %	LG	77.37	48.983578	76.2	2	Oxidation (+16)	653,8547	2 611,3895	4	0,009911	3,794	1 319,16	173800
(E)SFGDLSTPDAVmGNPKVKAHGKKVL(G)	100 %	LG	77.56	53.862347	67.93	2	Oxidation (+16)	871,4683	2 611,3832	3	0,003607	1,381	1 321,57	138814
(E)SFGDLSTPDAVMGNPKVKAHGKKVL(G)	100 %	LG	71.42	48.602844	58.61	2		866,1375	2 595,3906	3	0,005882	2,265	1 603,69	104361
(E)SFGDLSTPDAVMGNPKVKAHGKKVL(G)	100 %	LG	64.34	48.768353	63.98	2		649,8539	2 595,3866	4	0,001946	0,7495	1 601,35	182254
(E)SFGDLSTPDAVMGNPKVKAHGKKVL(G)	99 %	LG	56.75	48.92929	54.2	2		649,8518	2 595,3781	4	-0,006534	-2,517	1 940,85	56 893,0

(E)SFGDLSTPDAVmGNPKVKAHGKKVL(G)	99 %	LG	53.94	48.935173	48.83	2	Oxidation (+16)	871,4706	2 611,3900	3	0,01039	3,976	1 319,16	162608
(E)SFGDLSTPDAVMGNPKVKAHGKKVL(G)	99 %	LG	53.43	48.487312	39.39	2		866,1381	2 595,3924	3	0,007772	2,993	1 600,43	42 135,0
(E)SFGDLSTPDAVMGNPKVKAHGKKVL(G)	98 %	LG	50.71	48.971428	49.09	2		649,852	2 595,3787	4	-0,005974	-2,301	1 965,37	55 515,0
(E)SFGDLSTPDAVmGNPKVKAHGKKVL(G)	94 %	LG	45.13	49.160164	42.85	2	Oxidation (+16)	653,8536	2 611,3853	4	0,005751	2,201	1 360,15	60 458,0
(E)SFGDLSTPDAVmGNPKVKAHGKKVL(G)	91 %	LG	42.03	48.503952	29.01	2	Oxidation (+16)	871,4752	2 611,4039	3	0,02431	9,304	1 314,82	58 009,0
(E)SFGDLSTPDAVMGNPKVKAHGKKVLG(A)	100 %	GA	121.6	49.114666	96.29	2		885,1427	2 652,4062	3	0,00004206	0,01585	1 588,51	361189
(E)SFGDLSTPDAVmGNPKVKAHGKKVLG(A)	100 %	GA	103.87	49.333504	101.83	2	Oxidation (+16)	668,1091	2 668,4074	4	0,006331	2,372	1 302,82	479824
(E)SFGDLSTPDAVmGNPKVKAHGKKVLG(A)	100 %	GA	96.2	49.32032	78.85	2	Oxidation (+16)	890,4766	2 668,4079	3	0,006797	2,546	1 302,82	435138
(E)SFGDLSTPDAVMGNPKVKAHGKKVLG(A)	100 %	GA	93.23	49.030575	91.9	2		664,1097	2 652,4097	4	0,003566	1,344	1 610,78	257394
(E)SFGDLSTPDAVMGNPKVKAHGKKVLG(A)	100 %	GA	82.47	50.201374	68.05	2		885,1429	2 652,4068	3	0,0006121	0,2307	1 613,11	227005
(E)SFGDLSTPDAVMGNPKVKAHGKKVLG(A)	100 %	GA	71.88	49.103737	46.59	2		664,1095	2 652,4089	4	0,002766	1,042	1 865,01	98 381,0
(E)SFGDLSTPDAVmGNPKVKAHGKKVLG(A)	100 %	GA	67.88	49.673748	67.88	2	Oxidation (+16)	534,6855	2 668,3910	5	-0,01011	-3,789	1 302,90	88 776,0
(E)SFGDLSTPDAVMGNPKVKAHGKKVLG(A)	100 %	GA	67.33	50.245228	46.69	2		885,1419	2 652,4038	3	-0,002328	-0,8773	1 585,09	261850
(E)SFGDLSTPDAVMGNPKVKAHGKKVLG(A)	100 %	GA	65.4	49.220398	59.37	2		664,1083	2 652,4041	4	-0,002074	-0,7816	1 586,18	214012
(E)SFGDLSTPDAVMGNPKVKAHGKKVLG(A)	100 %	GA	61.75	49.112057	44.22	2		885,1428	2 652,4067	3	0,0005221	0,1967	1 585,17	112851
(E)SFGDLSTPDAVMGNPKVKAHGKKVLG(A)	100 %	GA	60.66	48.99777	51.02	2		885,1438	2 652,4095	3	0,003282	1,237	1 614,28	62 436,0
(E)SFGDLSTPDAVmGNPKVKAHGKKVLG(A)	99 %	GA	59.85	49.35442	58.85	2	Oxidation (+16)	668,1089	2 668,4066	4	0,005491	2,057	1 301,65	126659
(E)SFGDLSTPDAVMGNPKVKAHGKKVLG(A)	99 %	GA	58.0	49.381695	58.0	2		531,4867	2 652,3972	5	-0,009	-3,392	1 596,76	17 536,0
(E)SFGDLSTPDAVMGNPKVKAHGKKVLG(A)	98 %	GA	51.25	49.297966	34.79	2		885,1408	2 652,4006	3	-0,005538	-2,087	1 584,34	151864
(E)SFGDLSTPDAVmGNPKVKAHGKKVLG(A)	98 %	GA	51.37	49.427666	38.41	2	Oxidation (+16)	890,4753	2 668,4040	3	0,002957	1,108	1 301,65	106721
(E)SFGDLSTPDAVmGNPKVKAHGKKVLG(A)	98 %	GA	51.16	49.54638	37.17	2	Oxidation (+16)	668,1066	2 668,3972	4	-0,003869	-1,449	1 302,99	50 747,0
(S)FGDLSTPDAVMGNPKVK(A)	100 %	KA	128.22	49.21546	108.27	2		592,6385	1 774,8935	3	0,0008521	0,4798	1 702,89	220024
(S)FGDLSTPDAVMGNPKVK(A)	100 %	KA	79.09	49.13867	73.47	2		592,6365	1 774,8875	3	-0,005148	-2,899	1 700,64	96 411,0
(S)FGDLSTPDAVMGNPKVK(A)	100 %	KA	78.04	49.047966	57.26	2		888,4542	1 774,8939	2	0,001238	0,6971	1 702,89	156612
(S)FGDLSTPDAVMGNPKVK(A)	100 %	KA	76.21	49.26836	66.66	2		592,637	1 774,8891	3	-0,003528	-1,987	1 701,72	37 461,0
(S)FGDLSTPDAVMGNPKVK(A)	100 %	KA	74.4	49.047966	57.19	2		888,4543	1 774,8939	2	0,001278	0,7197	1 700,55	26 638,0
(S)FGDLSTPDAVmGNPKVK(A)	100 %	KA	71.89	49.23011	60.63	2	Oxidation (+16)	597,9688	1 790,8845	3	-0,003103	-1,732	1 552,88	36 065,0
(S)FGDLSTPDAVMGNPKVK(A)	100 %	KA	68.91	49.202747	58.99	2		888,4534	1 774,8922	2	-0,0005019	-0,2826	1 702,89	46 666,0
(S)FGDLSTPDAVmGNPKVK(A)	100 %	KA	65.48	49.157005	46.22	2	Oxidation (+16)	597,9717	1 790,8931	3	0,005537	3,09	1 449,86	25 917,0
(S)FGDLSTPDAVMGNPKVK(A)	99 %	KA	59.71	49.21546	51.47	2		592,6384	1 774,8935	3	0,0007921	0,446	1 755,65	21 869,0
(S)FGDLSTPDAVMGNPKVK(A)	94 %	KA	45.35	49.035892	29.85	2		888,4544	1 774,8942	2	0,001498	0,8435	1 755,65	47 846,0
(S)FGDLSTPDAVMGNPKVKAHGKK(V)	100 %	KV	96.45	49.338013	92.72	2		575,0567	2 296,1977	4	-0,002514	-1,094	1 206,19	203975
(S)FGDLSTPDAVMGNPKVKAHGKK(V)	100 %	KV	75.31	49.060493	63.11	2		766,4101	2 296,2086	3	0,008392	3,653	1 204,36	69 130,0
(S)FGDLSTPDAVMGNPKVKAHGKK(V)	100 %	KV	73.56	49.204884	68.9	2		575,0578	2 296,2021	4	0,001926	0,8384	1 207,36	272994
(S)FGDLSTPDAVMGNPKVKAHGKK(V)	100 %	KV	70.84	48.905495	60.51	2		766,4123	2 296,2150	3	0,01484	6,461	1 205,11	63 373,0
(S)FGDLSTPDAVMGNPKVKAHGKK(V)	100 %	KV	69.0	49.44971	64.85	2		575,0556	2 296,1931	4	-0,007034	-3,062	1 204,03	47 764,0
(S)FGDLSTPDAVMGNPKVKAHGKK(V)	98 %	KV	51.6	49.106136	41.39	2		766,4095	2 296,2068	3	0,006592	2,87	1 206,19	138665
(S)FGDLSTPDAVmGNPKVKAHGKK(V)	95 %	KV	46.99	49.32164	33.46	2	Oxidation (+16)	771,7426	2 312,2060	3	0,01089	4,706	943,194	58 883,0
(S)FGDLSTPDAVMGNPKVKAHGKKVL(G)	96 %	LG	46.2	48.123116	46.2	2		628,0958	2 508,3541	4	0,001466	0,5842	1 547,73	32 391,0
(S)FGDLSTPDAVmGNPKVKAHGKKVL(G)	91 %	LG	43.23	49.19481	43.13	2	Oxidation (+16)	632,0903	2 524,3321	4	-0,01553	-6,149	1 229,82	53 014,0
(S)FGDLSTPDAVmGNPKVKAHGKKVLG(A)	100 %	GA	64.63	48.87882	58.34	2	Oxidation (+16)	861,4649	2 581,3728	3	0,003757	1,455	1 215,51	124915
(S)FGDLSTPDAVMGNPKVKAHGKKVLG(A)	100 %	GA	61.51	48.687325	60.94	2		642,3502	2 565,3717	4	-0,002514	-0,9796	1 522,07	45 674,0
(S)FGDLSTPDAVMGNPKVKAHGKKVLG(A)	100 %	GA	59.75	48.37961	58.97	2		642,3517	2 565,3777	4	0,003566	1,39	1 617,79	62 203,0
(S)FGDLSTPDAVMGNPKVKAHGKKVLG(A)	99 %	GA	59.07	48.77924	59.03	2		642,3489	2 565,3666	4	-0,007554	-2,943	1 524,49	211441
(S)FGDLSTPDAVmGNPKVKAHGKKVLG(A)	98 %	GA	51.1	48.61588	47.26	2	Oxidation (+16)	646,3521	2 581,3795	4	0,01037	4,016	1 213,18	56 833,0
(S)FGDLSTPDAVmGNPKVKAHGKKVLG(A)	97 %	GA	49.56	48.734543	41.1	2	Oxidation (+16)	861,4661	2 581,3765	3	0,007447	2,884	1 216,67	102601

(S)FGDLSTPDAVmGNPKVKAHGKKVLG(A)	97 %	GA	52.98	52.92564	46.92	2	Oxidation (+16)	646,3511	2 581,3752	4	0,006131	2,374	1 213,51	116533
(F)GDLSTPDAVMGNPKVKAHGKK(V)	100 %	KV	86.79	49.11802	72.43	2		717,3825	2 149,1256	3	-0,006158	-2,864	1 325,07	119717
(F)GDLSTPDAVMGNPKVKAHGKK(V)	100 %	KV	79.32	48.94072	65.06	2		717,384	2 149,1303	3	-0,001478	-0,6874	1 332,07	67 048,0
(F)GDLSTPDAVMGNPKVKAHGKK(V)	99 %	KV	54.12	48.93995	44.03	2		717,3839	2 149,1300	3	-0,001808	-0,8408	1 322,65	52 870,0
(F)GDLSTPDAVmGNPKVKAHGKK(V)	97 %	KV	49.65	49.202747	31.25	2	Oxidation (+16)	722,7169	2 165,1288	3	0,002067	0,9543	1 021,11	76 235,0
(F)GDLSTPDAVMGNPKVKAHGKK(V)	93 %	KV	44.45	48.953896	33.09	2		717,3836	2 149,1289	3	-0,002828	-1,315	878,856	34 180,0
(F)GDLSTPDAVMGNPKVKAHGKK(V)	93 %	KV	44.03	48.608948	37.44	2		717,3871	2 149,1394	3	0,007672	3,568	875,865	27 578,0
(F)GDLSTPDAVMGNPKVKAHGKKV(L)	100 %	VL	67.85	48.30941	53.92	2		750,4096	2 248,2069	3	0,006772	3,011	1 366,97	131931
(F)GDLSTPDAVMGNPKVKAHGKKV(L)	100 %	VL	67.02	48.30974	53.28	2		750,4094	2 248,2065	3	0,006322	2,811	1 365,80	97 337,0
(F)GDLSTPDAVMGNPKVKAHGKKV(L)	100 %	VL	66.94	52.991604	54.99	2		750,4098	2 248,2077	3	0,007492	3,331	1 364,88	139914
(F)GDLSTPDAVMGNPKVKAHGKKV(L)	99 %	VL	53.47	48.003597	43.27	2		750,412	2 248,2140	3	0,01385	6,159	1 364,97	73 528,0
(F)GDLSTPDAVMGNPKVKAHGKKV(L)	97 %	VL	48.81	48.17949	43.66	2		750,4108	2 248,2105	3	0,01031	4,585	1 363,97	41 003,0
(G)DLSTPDAVMGNPKVK(A)	100 %	KA	111.1	48.493885	92.56	2		786,4054	1 570,7962	2	-0,006562	-4,175	1 468,39	131024
(G)DLSTPDAVMGNPKVK(A)	100 %	KA	94.36	48.576702	71.81	2		786,408	1 570,8014	2	-0,001362	-0,8665	1 754,74	58 260,0
(G)DLSTPDAVMGNPKVK(A)	100 %	KA	84.91	48.53911	59.27	2		786,4083	1 570,8020	2	-0,0007219	-0,4593	1 755,40	103973
(G)DLSTPDAVmGNPKVK(A)	100 %	KA	81.76	48.8143	61.25	2	Oxidation (+16)	794,4066	1 586,7987	2	0,000983	0,6191	1 600,35	43 474,0
(G)DLSTPDAVmGNPKVK(A)	100 %	KA	80.55	48.8403	62.37	2	Oxidation (+16)	794,4059	1 586,7972	2	-0,000517	-0,3256	1 071,16	37 439,0
(G)DLSTPDAVMGNPKVK(A)	100 %	KA	67.27	48.620537	55.2	2		786,4059	1 570,7973	2	-0,005482	-3,488	1 466,13	59 078,0
(G)DLSTPDAVMGNPKVK(A)	99 %	KA	55.44	48.39176	38.75	2		786,41	1 570,8053	2	0,002578	1,64	1 701,72	30 659,0
(G)DLSTPDAVmGNPKVK(A)	94 %	KA	45.08	48.869587	32.47	2	Oxidation (+16)	794,4069	1 586,7993	2	0,001603	1,01	1 550,39	43 152,0
(G)DLSTPDAVMGNPKVKA(H)	100 %	AH	66.17	48.736053	55.79	2		821,9263	1 641,8380	2	-0,001882	-1,146	1 561,71	32 450,0
(G)DLSTPDAVMGNPKVKA(H)	98 %	AH	51.01	48.570007	45.73	2		821,9273	1 641,8401	2	0,0002381	0,1449	1 560,88	28 073,0
(G)DLSTPDAVMGNPKVKAHGKK(V)	100 %	KV	102.04	48.56838	73.77	2		698,3777	2 092,1112	3	0,0009121	0,4357	911,495	403500
(G)DLSTPDAVmGNPKVKAHGKK(V)	100 %	KV	87.5	48.582184	72.12	2	Oxidation (+16)	703,7123	2 108,1152	3	0,01001	4,745	675,942	84 569,0
(G)DLSTPDAVMGNPKVKAHGKK(V)	100 %	KV	80.26	48.624832	54.2	2		698,3762	2 092,1068	3	-0,003438	-1,643	910,326	243542
(G)DLSTPDAVMGNPKVKAHGKK(V)	99 %	KV	55.22	48.65347	36.25	2		698,3759	2 092,1059	3	-0,004368	-2,087	909,158	90 570,0
(G)DLSTPDAVMGNPKVKAHGKKV(L)	100 %	VL	65.07	47.95595	56.26	2		731,4015	2 191,1826	3	0,003942	1,798	979,036	114962
(G)DLSTPDAVMGNPKVKAHGKKV(L)	100 %	VL	63.57	47.927135	47.79	2		731,4014	2 191,1824	3	0,003762	1,716	979,203	54 084,0
(G)DLSTPDAVMGNPKVKAHGKKVLG(A)	100 %	GA	133.04	49.32768	119.54	2		788,1026	2 361,2860	3	0,001702	0,7205	1 233,06	1207150
(G)DLSTPDAVMGNPKVKAHGKKVLG(A)	100 %	GA	80.2	47.663235	67.79	2		788,1017	2 361,2834	3	-0,0009079	-0,3843	1 231,81	108113
(G)DLSTPDAVMGNPKVKAHGKKVLG(A)	100 %	GA	63.94	47.559204	50.54	2		788,1021	2 361,2845	3	0,0002021	0,08553	1 233,06	69 417,0
(G)DLSTPDAVmGNPKVKAHGKKVLG(A)	99 %	GA	57.51	52.85845	36.99	2	Oxidation (+16)	793,4356	2 377,2849	3	0,005667	2,383	979,12	77 284,0
(G)DLSTPDAVmGNPKVKAHGKKVLG(A)	92 %	GA	42.03	47.8814	29.75	2	Oxidation (+16)	793,4361	2 377,2864	3	0,007227	3,039	977,952	85 502,0
(D)LSTPDAVMGNPKVK(A)	100 %	KA	92.51	47.88211	71.34	2		728,8947	1 455,7749	2	-0,0009019	-0,6191	1 279,54	63 429,0
(D)LSTPDAVMGNPKVK(A)	100 %	KA	77.89	47.879562	57.98	2		728,8949	1 455,7751	2	-0,0006219	-0,4269	1 280,70	47 696,0
(D)LSTPDAVMGNPKVK(A)	100 %	KA	74.99	47.880978	56.87	2		728,8943	1 455,7740	2	-0,001782	-1,223	1 755,57	109514
(D)LSTPDAVMGNPKVK(A)	100 %	KA	74.34	51.60724	53.34	2		728,894	1 455,7734	2	-0,002342	-1,608	1 277,46	34 884,0
(D)LSTPDAVmGNPKVK(A)	100 %	KA	68.67	48.111862	51.48	2	Oxidation (+16)	736,8951	1 471,7756	2	0,004903	3,329	1 601,43	64 852,0
(D)LSTPDAVMGNPKVK(A)	99 %	KA	58.62	47.853085	50.16	2		486,2647	1 455,7723	3	-0,003438	-2,36	1 277,71	8 310,00
(D)LSTPDAVMGNPKVK(A)	99 %	KA	54.46	47.837532	41.45	2		486,2649	1 455,7728	3	-0,003018	-2,072	1 278,46	16 768,0
(D)LSTPDAVMGNPKVKAHGKK(V)	100 %	KV	108.68	52.242924	107.05	2		989,5475	1 977,0804	2	-0,002822	-1,427	1 389,20	92 957,0
(D)LSTPDAVMGNPKVKAHGKK(V)	98 %	KV	50.77	46.759525	43.27	2		660,0372	1 977,0897	3	0,006472	3,272	791,431	77 890,0
(D)LSTPDAVMGNPKVKAHGKK(V)	97 %	KV	47.02	46.669296	34.39	2		660,0368	1 977,0885	3	0,005272	2,665	789,428	69 321,0
(D)LSTPDAVMGNPKVKAHGKK(V)	97 %	KV	47.12	47.121105	39.62	2		989,5476	1 977,0807	2	-0,002542	-1,285	1 331,99	75 650,0
(D)LSTPDAVMGNPKVKAHGKK(V)	91 %	KV	41.24	47.16354	41.02	2		989,5484	1 977,0823	2	-0,0009619	-0,4863	1 386,86	56 428,0
(D)LSTPDAVMGNPKVKAHGKKVLG(A)	100 %	GA	69.02	45.830173	61.1	2		749,7589	2 246,2549	3	-0,002398	-1,067	1 123,51	224053

(D)LSTPDAVmGNPKVKAHGKKVLG(A)	94 %	GA	42.04	46.0651	33.15	2	Oxidation (+16)	755,0931	2 262,2576	3	0,005377	2,376	909,158	68 114,0
(L)STPDAVMGNPKVK(A)	100 %	KA	89.72	48.009575	75.8	2		672,3517	1 342,6888	2	-0,002882	-2,145	1 756,57	151598
(L)STPDAVMGNPKVK(A)	100 %	KA	81.36	48.401875	63.2	2		672,3521	1 342,6896	2	-0,002042	-1,52	1 754,40	83 382,0
(L)STPDAVmGNPKVK(A)	100 %	KA	78.84	48.53917	57.46	2	Oxidation (+16)	680,3516	1 358,6886	2	0,002023	1,488	1 601,35	96 819,0
(L)STPDAVMGNPKVK(A)	100 %	KA	71.2	48.009163	55.09	2		672,3509	1 342,6872	2	-0,004462	-3,321	1 468,39	22 701,0
(L)STPDAVMGNPKVK(A)	100 %	KA	68.42	48.01705	54.88	2		672,351	1 342,6874	2	-0,004222	-3,142	1 756,57	172277
(L)STPDAVMGNPKVK(A)	100 %	KA	67.84	48.009575	52.75	2		672,3517	1 342,6888	2	-0,002822	-2,1	1 754,23	47 312,0
(L)STPDAVMGNPKVK(A)	100 %	KA	68.08	48.249847	38.26	2		672,3542	1 342,6938	2	0,002138	1,591	1 701,97	33 343,0
(L)STPDAVMGNPKVK(A)	100 %	KA	67.28	53.992027	56.93	2		672,3537	1 342,6928	2	0,001098	0,8172	887,52	52 175,0
(L)STPDAVmGNPKVK(A)	100 %	KA	59.43	48.57079	46.39	2	Oxidation (+16)	680,3502	1 358,6858	2	-0,000757	-0,5567	1 551,22	75 520,0
(L)STPDAVMGNPKVK(A)	99 %	KA	58.89	48.20595	46.69	2		672,3538	1 342,6930	2	0,001318	0,9809	884,933	26 026,0
(L)STPDAVmGNPKVK(A)	99 %	KA	58.23	48.48146	39.49	2	Oxidation (+16)	680,3518	1 358,6890	2	0,002463	1,811	1 601,51	45 057,0
(L)STPDAVMGNPKVK(A)	93 %	KA	43.81	48.34001	31.66	2		672,3536	1 342,6926	2	0,0009381	0,6981	1 467,56	37 481,0
(L)STPDAVMGNPKVKAHGKK(V)	100 %	KV	91.6	48.015064	77.23	2		622,34	1 863,9981	3	-0,001058	-0,5673	911,746	170652
(L)STPDAVMGNPKVKAHGKK(V)	100 %	KV	82.75	47.748962	68.99	2		622,3413	1 864,0019	3	0,002752	1,476	917,329	112940
(L)STPDAVMGNPKVKAHGKK(V)	100 %	KV	78.14	48.015064	68.83	2		933,0062	1 863,9979	2	-0,001242	-0,6659	1 331,99	292272
(L)STPDAVMGNPKVKAHGKK(V)	100 %	KV	77.33	47.775787	66.97	2		933,0082	1 864,0018	2	0,002678	1,436	1 386,77	171712
(L)STPDAVMGNPKVKAHGKK(V)	100 %	KV	74.7	48.019657	63.82	2		622,3407	1 864,0004	3	0,001192	0,6392	595,514	71 622,0
(L)STPDAVMGNPKVKAHGKK(V)	100 %	KV	68.5	47.91143	60.7	2		933,007	1 863,9994	2	0,0001981	0,1062	1 384,51	50 321,0
(L)STPDAVMGNPKVKAHGKK(V)	100 %	KV	68.0	47.917957	61.26	2		933,0068	1 863,9991	2	-0,00008194	-0,04394	601,274	77 818,0
(L)STPDAVmGNPKVKAHGKK(V)	99 %	KV	57.66	48.264374	50.67	2	Oxidation (+16)	627,672	1 879,9941	3	0,00005705	0,03033	1 109,54	57 604,0
(L)STPDAVMGNPKVKAHGKK(V)	99 %	KV	56.92	47.918938	49.89	2		933,0066	1 863,9985	2	-0,0006219	-0,3335	1 326,15	35 988,0
(L)STPDAVMGNPKVKAHGKK(V)	98 %	KV	51.8	48.078594	45.45	2		933,006	1 863,9975	2	-0,001682	-0,9018	911,495	67 655,0
(L)STPDAVmGNPKVKAHGKK(V)	98 %	KV	49.55	48.019318	37.73	2	Oxidation (+16)	627,6746	1 880,0019	3	0,007857	4,177	676,441	48 646,0
(L)STPDAVMGNPKVKAHGKK(V)	92 %	KV	46.17	51.68076	46.05	2		933,0074	1 864,0001	2	0,0009781	0,5244	1 335,50	35 498,0
(L)STPDAVMGNPKVKAHGKKV(L)	100 %	VL	79.03	51.76725	70.08	2		655,3647	1 963,0722	3	0,004672	2,379	980,292	249112
(L)STPDAVMGNPKVKAHGKKV(L)	100 %	VL	70.63	47.414825	61.87	2		655,3631	1 963,0675	3	-0,00006794	-0,03459	1 366,97	90 004,0
(L)STPDAVMGNPKVKAHGKKV(L)	95 %	VL	44.34	47.32177	37.89	2		982,5422	1 963,0698	2	0,002258	1,15	981,382	108680
(L)STPDAVMGNPKVKAHGKKVL(G)	100 %	LG	82.4	46.17084	73.98	2		693,0582	2 076,1528	3	0,001162	0,5594	889,348	217164
(L)STPDAVMGNPKVKAHGKKVL(G)	100 %	LG	76.09	46.347893	68.01	2		693,0575	2 076,1506	3	-0,001058	-0,5093	1 250,46	77 518,0
(L)STPDAVMGNPKVKAHGKKVL(G)	95 %	LG	43.47	46.31971	35.11	2		693,0578	2 076,1514	3	-0,0002479	-0,1194	887,104	35 834,0
(L)STPDAVmGNPKVKAHGKKVL(G)	92 %	LG	41.21	46.53338	36.69	2	Oxidation (+16)	698,3919	2 092,1539	3	0,007317	3,496	1 321,57	34 800,0
(L)STPDAVMGNPKVKAHGKKVLG(A)	100 %	GA	102.45	46.852844	96.48	2		712,0657	2 133,1754	3	0,002222	1,041	1 232,98	232258
(L)STPDAVMGNPKVKAHGKKVLG(A)	100 %	GA	101.64	46.75228	91.07	2		712,0665	2 133,1776	3	0,004442	2,081	1 616,53	218444
(L)STPDAVMGNPKVKAHGKKVLG(A)	100 %	GA	90.68	46.843155	86.4	2		712,0659	2 133,1757	3	0,002552	1,196	884,683	344927
(L)STPDAVMGNPKVKAHGKKVLG(A)	100 %	GA	82.97	47.096092	79.35	2		712,0645	2 133,1717	3	-0,001468	-0,6878	1 588,60	213758
(L)STPDAVMGNPKVKAHGKKVLG(A)	100 %	GA	65.99	47.014904	58.28	2		712,0655	2 133,1746	3	0,001412	0,6616	1 585,34	108472
(L)STPDAVMGNPKVKAHGKKVLG(A)	100 %	GA	59.23	47.122368	54.99	2		712,0641	2 133,1706	3	-0,002578	-1,208	882,352	97 767,0
(L)STPDAVMGNPKVKAHGKKVLG(A)	99 %	GA	56.16	46.774883	50.81	2		712,0667	2 133,1782	3	0,005012	2,348	1 124,01	41 479,0
(L)STPDAVmGNPKVKAHGKKVLG(A)	99 %	GA	55.02	47.590103	46.88	2	Oxidation (+16)	717,3971	2 149,1694	3	0,001357	0,6311	977,952	133467
(L)STPDAVmGNPKVKAHGKKVLG(A)	97 %	GA	48.63	47.382492	41.76	2	Oxidation (+16)	717,398	2 149,1722	3	0,004147	1,929	1 302,90	55 728,0
(L)STPDAVmGNPKVKAHGKKVLG(A)	97 %	GA	48.6	47.46206	41.22	2	Oxidation (+16)	717,3976	2 149,1709	3	0,002857	1,329	1 375,11	52 343,0
(L)STPDAVMGNPKVKAHGKKVLG(A)	97 %	GA	47.17	46.852844	40.02	2		712,0657	2 133,1754	3	0,002192	1,027	1 231,89	75 570,0
(L)STPDAVmGNPKVKAHGKKVLG(A)	97 %	GA	47.18	47.623634	42.39	2	Oxidation (+16)	717,3964	2 149,1675	3	-0,000623	-0,2897	1 213,68	47 624,0
(L)STPDAVMGNPKVKAHGKKVLG(A)	93 %	GA	42.37	47.31121	38.91	2		712,0627	2 133,1662	3	-0,006928	-3,246	1 194,89	39 137,0
(L)STPDAVmGNPKVKAHGKKVLG(A)	92 %	GA	41.81	47.404972	33.8	2	Oxidation (+16)	717,3985	2 149,1736	3	0,005557	2,584	1 367,13	87 607,0

(S)TPDAVMGNPKVK(A)	100 %	KA	84.78	47.52563	68.0	2		628,8375	1 255,6604	2	0,0007581	0,6032	848,596	107976
(S)TPDAVMGNPKVK(A)	100 %	KA	79.88	47.525173	66.2	2		628,8373	1 255,6600	2	0,0003181	0,2531	853,338	56 999,0
(S)TPDAVmGNPKVK(A)	100 %	KA	78.9	48.380238	52.37	2	Oxidation (+16)	636,8342	1 271,6538	2	-0,000797	-0,6262	639,744	63 365,0
(S)TPDAVMGNPKVK(A)	100 %	KA	74.29	47.54004	59.51	2		628,8358	1 255,6571	2	-0,002602	-2,071	1 756,82	30 770,0
(S)TPDAVMGNPKVK(A)	100 %	KA	73.36	47.348877	56.45	2		628,8362	1 255,6579	2	-0,001742	-1,386	824,103	93 593,0
(S)TPDAVMGNPKVK(A)	100 %	KA	63.34	47.504005	51.7	2		628,8371	1 255,6597	2	0,00001806	0,01437	847,429	34 697,0
(S)TPDAVMGNPKVK(A)	100 %	KA	63.04	47.83532	55.05	2		628,8371	1 255,6595	2	-0,0001219	-0,09704	1 471,05	22 802,0
(S)TPDAVMGNPKVK(A)	100 %	KA	61.36	47.348877	45.09	2		628,8363	1 255,6580	2	-0,001702	-1,354	823,019	23 928,0
(S)TPDAVMGNPKVK(A)	100 %	KA	61.38	47.405205	46.55	2		628,8346	1 255,6546	2	-0,005102	-4,06	1 755,40	44 735,0
(S)TPDAVMGNPKVK(A)	100 %	KA	60.16	47.592297	45.33	2		628,8391	1 255,6635	2	0,003878	3,086	821,768	24 464,0
(S)TPDAVmGNPKVK(A)	98 %	KA	51.62	48.11756	29.43	2	Oxidation (+16)	636,8367	1 271,6589	2	0,004323	3,397	1 552,55	30 576,0
(S)TPDAVMGNPKVK(A)	98 %	KA	49.76	47.33141	36.16	2		628,8381	1 255,6616	2	0,001938	1,542	847,597	35 808,0
(S)TPDAVmGNPKVK(A)	92 %	KA	42.94	48.178497	32.95	2	Oxidation (+16)	636,835	1 271,6554	2	0,000803	0,631	633,077	36 218,0
(S)TPDAVMGNPKVKAHGKK(V)	100 %	KV	109.23	47.363487	93.95	2		593,3297	1 776,9674	3	0,0002221	0,1249	585,749	706996
(S)TPDAVMGNPKVKAHGKK(V)	100 %	KV	99.49	46.87065	84.52	2		593,3323	1 776,9750	3	0,007812	4,394	583,416	575941
(S)TPDAVMGNPKVKAHGKK(V)	100 %	KV	93.04	47.36229	78.06	2		593,3297	1 776,9673	3	0,0001021	0,0574	579,912	218949
(S)TPDAVMGNPKVKAHGKK(V)	100 %	KV	67.42	47.10329	57.06	2		593,3303	1 776,9690	3	0,001812	1,019	575,332	89 973,0
(S)TPDAVMGNPKVKAHGKK(V)	100 %	KV	65.8	47.21753	58.27	2		889,492	1 776,9695	2	0,002318	1,304	1 386,77	116116
(S)TPDAVMGNPKVKAHGKK(V)	100 %	KV	67.9	52.036476	57.96	2		593,3296	1 776,9671	3	-0,00007794	-0,04384	579,995	173992
(S)TPDAVmGNPKVKAHGKK(V)	100 %	KV	62.63	47.528008	43.98	2	Oxidation (+16)	598,6618	1 792,9636	3	0,001547	0,8624	1 022,44	51 235,0
(S)TPDAVMGNPKVKAHGKK(V)	99 %	KV	61.29	52.490562	50.55	2		593,3272	1 776,9599	3	-0,007278	-4,093	1 324,32	45 127,0
(S)TPDAVMGNPKVKAHGKK(V)	99 %	KV	55.56	47.42874	46.23	2		593,3295	1 776,9666	3	-0,0005579	-0,3138	532,667	24 349,0
(S)TPDAVMGNPKVKAHGKK(V)	99 %	KV	54.06	47.422222	40.06	2		593,3285	1 776,9638	3	-0,003378	-1,9	910,66	36 450,0
(S)TPDAVMGNPKVKAHGKK(V)	98 %	KV	51.22	47.422142	39.46	2		593,3286	1 776,9638	3	-0,003348	-1,883	791,431	43 496,0
(S)TPDAVMGNPKVKAHGKK(V)	98 %	KV	49.55	47.17604	37.02	2		593,3304	1 776,9693	3	0,002172	1,222	790,764	41 686,0
(S)TPDAVmGNPKVKAHGKK(V)	98 %	KV	48.98	47.475746	40.55	2	Oxidation (+16)	598,6629	1 792,9670	3	0,004877	2,719	583,416	57 369,0
(S)TPDAVMGNPKVKAHGKK(V)	97 %	KV	48.34	47.671036	48.34	2		889,4886	1 776,9627	2	-0,004482	-2,521	1 331,99	164680
(S)TPDAVMGNPKVKAHGKK(V)	97 %	KV	46.72	47.10202	33.44	2		593,3302	1 776,9689	3	0,001692	0,9517	562,426	30 960,0
(S)TPDAVMGNPKVKAHGKK(V)	96 %	KV	51.04	52.29003	40.63	2		889,4917	1 776,9688	2	0,001638	0,9213	1 391,46	124858
(S)TPDAVMGNPKVKAHGKK(V)	96 %	KV	46.39	47.65505	41.18	2		445,2481	1 776,9633	4	-0,003914	-2,201	582,414	19 154,0
(S)TPDAVmGNPKVKAHGKK(V)	96 %	KV	45.94	47.950104	32.47	2	Oxidation (+16)	598,6596	1 792,9570	3	-0,005053	-2,817	676,441	52 305,0
(S)TPDAVMGNPKVKAHGKK(V)	93 %	KV	41.99	46.820724	29.84	2		593,3324	1 776,9754	3	0,008202	4,613	546,126	23 316,0
(S)TPDAVmGNPKVKAHGKK(V)	92 %	KV	42.01	47.532764	40.2	2	Oxidation (+16)	598,6635	1 792,9688	3	0,006707	3,739	1 107,21	22 484,0
(S)TPDAVmGNPKVKAHGKK(V)	92 %	KV	41.84	47.618675	35.66	2	Oxidation (+16)	897,4899	1 792,9652	2	0,003123	1,741	1 022,19	16 474,0
(S)TPDAVMGNPKVKAHGKKV(L)	100 %	VL	105.07	45.903736	94.78	2		626,3561	1 876,0465	3	0,01093	5,824	635,079	596768
(S)TPDAVMGNPKVKAHGKKV(L)	100 %	VL	89.16	46.481453	82.92	2		626,3541	1 876,0404	3	0,004872	2,596	633,91	312190
(S)TPDAVMGNPKVKAHGKKV(L)	100 %	VL	68.99	47.036266	61.74	2		626,3509	1 876,0308	3	-0,004758	-2,535	1 368,13	88 200,0
(S)TPDAVmGNPKVKAHGKKV(L)	100 %	VL	66.61	47.2385	61.45	2	Oxidation (+16)	631,6844	1 892,0314	3	0,000887	0,4686	562,676	81 268,0
(S)TPDAVMGNPKVKAHGKKV(L)	100 %	VL	60.31	46.587074	59.93	2		470,0166	1 876,0372	4	0,001646	0,8769	633,91	32 993,0
(S)TPDAVMGNPKVKAHGKKV(L)	99 %	VL	57.34	46.587364	52.36	2		626,3531	1 876,0374	3	0,001842	0,9814	635,163	41 201,0
(S)TPDAVMGNPKVKAHGKKV(L)	99 %	VL	55.46	46.380997	48.56	2		626,3539	1 876,0400	3	0,004392	2,34	628,155	46 585,0
(S)TPDAVMGNPKVKAHGKKV(L)	96 %	VL	45.74	47.02129	34.84	2		626,3502	1 876,0287	3	-0,006828	-3,638	1 452,02	68 987,0
(S)TPDAVmGNPKVKAHGKKVLG(A)	100 %	GA	127.86	46.39048	125.43	2	Oxidation (+16)	688,3879	2 062,1418	3	0,005667	2,747	665,435	569854
(S)TPDAVMGNPKVKAHGKKVLG(A)	100 %	GA	110.53	45.586246	108.44	2		683,0562	2 046,1468	3	0,005662	2,766	1 616,53	102906
(S)TPDAVMGNPKVKAHGKKVLG(A)	100 %	GA	102.1	45.87318	96.27	2		683,0548	2 046,1427	3	0,001522	0,7435	853,254	397273
(S)TPDAVMGNPKVKAHGKKVLG(A)	100 %	GA	98.42	45.586246	94.85	2		683,0562	2 046,1469	3	0,005722	2,795	835,76	789277
(S)TPDAVMGNPKVKAHGKKVLG(A)	100 %	GA	97.31	46.721718	97.31	2		512,5436	2 046,1453	4	0,004086	1,996	835,844	1110460

(S)TPDAVmGNPKVKAHGKKVLG(A)	100 %	GA	78.93	46.569313	78.93	2	Oxidation (+16)	516,5423	2 062,1401	4	0,004011	1,944	665,435	120446
(S)TPDAVMGNPKVKAHGKKVLG(A)	100 %	GA	71.82	48.15119	67.84	2		683,0573	2 046,1500	3	0,008782	4,29	1 618,21	68 919,0
(S)TPDAVmGNPKVKAHGKKVLG(A)	100 %	GA	70.08	46.57486	67.6	2	Oxidation (+16)	516,5419	2 062,1383	4	0,002251	1,091	664,432	93 283,0
(S)TPDAVMGNPKVKAHGKKVLG(A)	100 %	GA	64.35	46.138733	60.9	2		683,0539	2 046,1399	3	-0,001298	-0,634	1 123,59	39 735,0
(S)TPDAVMGNPKVKAHGKKVLG(A)	100 %	GA	62.73	46.2421	61.68	2		683,0534	2 046,1383	3	-0,002888	-1,411	1 588,51	119797
(S)TPDAVmGNPKVKAHGKKVLG(A)	100 %	GA	63.05	46.83812	56.07	2	Oxidation (+16)	688,3861	2 062,1364	3	0,000357	0,1731	664,349	116911
(S)TPDAVMGNPKVKAHGKKVLG(A)	100 %	GA	58.58	45.83675	57.24	2		683,0554	2 046,1443	3	0,003112	1,52	1 537,31	29 206,0
(S)TPDAVMGNPKVKAHGKKVLG(A)	100 %	GA	58.47	45.902287	54.76	2		683,0548	2 046,1425	3	0,001312	0,6409	1 234,14	72 453,0
(S)TPDAVMGNPKVKAHGKKVLG(A)	99 %	GA	55.35	45.62935	52.64	2		683,056	2 046,1462	3	0,005032	2,458	835,928	95 190,0
(S)TPDAVMGNPKVKAHGKKVLG(A)	99 %	GA	52.34	47.064278	48.58	2		512,5414	2 046,1367	4	-0,004514	-2,205	880,186	56 348,0
(S)TPDAVmGNPKVKAHGKKVLG(A)	98 %	GA	48.22	46.36618	44.55	2	Oxidation (+16)	688,3883	2 062,1429	3	0,006837	3,314	979,12	48 502,0
(S)TPDAVMGNPKVKAHGKKVLG(A)	96 %	GA	45.21	46.202507	45.21	2		512,542	2 046,1389	4	-0,002314	-1,13	855,666	59 787,0
(S)TPDAVMGNPKVKAHGKKVLG(A)	95 %	GA	43.08	46.136833	39.93	2		683,0544	2 046,1413	3	0,0001121	0,05474	1 232,31	34 820,0
(S)TPDAVMGNPKVKAHGKKVLG(A)	92 %	GA	40.76	46.132706	34.96	2		683,0537	2 046,1392	3	-0,001958	-0,9564	1 585,17	52 532,0
(S)TPDAVmGNPKVKAHGKKVLG(A)	91 %	GA	41.21	47.214397	38.36	2	Oxidation (+16)	516,5421	2 062,1391	4	0,003011	1,459	836,094	81 604,0
(S)TPDAVmGNPKVKAHGKKVLG(A)	91 %	GA	40.26	46.53589	33.3	2	Oxidation (+16)	688,3874	2 062,1405	3	0,004377	2,122	1 366,05	54 903,0
(S)TPDAVMGNPKVKAHGKKVLGA(F)	100 %	AF	57.89	46.262684	54.36	2		706,7332	2 117,1779	3	-0,0004079	-0,1926	889,431	41 127,0
(S)TPDAVMGNPKVKAHGKKVLGA(F)	95 %	AF	43.98	46.571804	43.98	2		530,3006	2 117,1731	4	-0,005134	-2,424	889,348	22 781,0
(T)PDAVMGNPKVK(A)	100 %	KA	69.43	46.330444	39.83	2		578,3126	1 154,6106	2	-0,001322	-1,144	1 468,64	25 734,0
(T)PDAVmGNPKVK(A)	100 %	KA	63.24	46.99864	48.69	2	Oxidation (+16)	586,3105	1 170,6065	2	-0,000397	-0,3388	1 600,68	46 612,0
(T)PDAVMGNPKVK(A)	100 %	KA	62.71	46.985966	38.01	2		578,3112	1 154,6079	2	-0,004102	-3,55	1 755,90	30 834,0
(T)PDAVmGNPKVK(A)	99 %	KA	57.87	47.5969	47.84	2	Oxidation (+16)	586,3078	1 170,6011	2	-0,005817	-4,965	1 552,55	25 948,0
(T)PDAVmGNPKVK(A)	97 %	KA	48.54	47.596603	38.5	2	Oxidation (+16)	586,308	1 170,6015	2	-0,005357	-4,572	1 551,72	22 020,0
(T)PDAVMGNPKVK(A)	92 %	KA	41.15	46.467075	19.49	2		578,3146	1 154,6147	2	0,002698	2,335	1 282,37	10 534,0
(T)PDAVMGNPKVKAHGKK(V)	100 %	KV	119.73	47.084465	105.19	2		838,9658	1 675,9170	2	-0,002462	-1,468	1 333,16	253454
(T)PDAVMGNPKVKAHGKK(V)	100 %	KV	115.92	46.83083	103.83	2		838,9678	1 675,9211	2	0,001658	0,9887	1 386,77	406250
(T)PDAVMGNPKVKAHGKK(V)	100 %	KV	91.2	47.311935	80.7	2		559,6454	1 675,9144	3	-0,005038	-3,004	1 385,77	49 449,0
(T)PDAVmGNPKVKAHGKK(V)	100 %	KV	89.53	47.387253	87.08	2	Oxidation (+16)	564,979	1 691,9151	3	0,000727	0,4295	677,107	44 563,0
(T)PDAVMGNPKVKAHGKK(V)	100 %	KV	89.21	47.084465	73.28	2		559,6463	1 675,9171	3	-0,002368	-1,412	910,326	39 844,0
(T)PDAVMGNPKVKAHGKK(V)	100 %	KV	83.93	46.78054	69.07	2		559,6469	1 675,9190	3	-0,0005079	-0,3029	792,6	72 795,0
(T)PDAVmGNPKVKAHGKK(V)	100 %	KV	84.6	47.590256	72.91	2	Oxidation (+16)	564,9777	1 691,9113	3	-0,003113	-1,839	1 022,19	96 475,0
(T)PDAVMGNPKVKAHGKK(V)	100 %	KV	83.1	46.78127	73.19	2		838,9668	1 675,9191	2	-0,0003619	-0,2158	1 384,68	146083
(T)PDAVMGNPKVKAHGKK(V)	100 %	KV	83.19	46.977264	68.56	2		559,6467	1 675,9183	3	-0,001198	-0,7144	601,274	60 784,0
(T)PDAVMGNPKVKAHGKK(V)	100 %	KV	78.12	47.082336	63.22	2		838,9659	1 675,9172	2	-0,002242	-1,337	601,274	109505
(T)PDAVmGNPKVKAHGKK(V)	100 %	KV	72.58	47.39683	66.18	2	Oxidation (+16)	846,9655	1 691,9164	2	0,002043	1,207	1 022,19	94 686,0
(T)PDAVMGNPKVKAHGKK(V)	100 %	KV	68.2	46.83083	57.77	2		559,6476	1 675,9211	3	0,001592	0,9494	585,749	43 911,0
(T)PDAVmGNPKVKAHGKK(V)	100 %	KV	64.38	47.38606	57.01	2	Oxidation (+16)	564,9787	1 691,9144	3	0,00000705	0,004164	1 110,70	38 751,0
(T)PDAVMGNPKVKAHGKK(V)	100 %	KV	61.8	46.66424	48.98	2		559,6483	1 675,9232	3	0,003692	2,202	790,597	47 440,0
(T)PDAVmGNPKVKAHGKK(V)	100 %	KV	60.87	47.53407	55.8	2	Oxidation (+16)	423,9856	1 691,9133	4	-0,001129	-0,6669	1 021,52	12 621,0
(T)PDAVMGNPKVKAHGKK(V)	100 %	KV	59.02	47.406864	52.92	2		559,6437	1 675,9092	3	-0,01026	-6,117	1 323,98	37 342,0
(T)PDAVMGNPKVKAHGKK(V)	99 %	KV	54.24	46.78045	44.57	2		838,9667	1 675,9187	2	-0,0007219	-0,4305	1 328,49	57 802,0
(T)PDAVMGNPKVKAHGKK(V)	98 %	KV	50.32	46.97578	47.79	2		838,9664	1 675,9182	2	-0,001282	-0,7645	1 411,35	23 081,0
(T)PDAVmGNPKVKAHGKK(V)	98 %	KV	49.76	47.548832	40.76	2	Oxidation (+16)	564,9774	1 691,9104	3	-0,003983	-2,353	1 021,02	39 955,0
(T)PDAVMGNPKVKAHGKK(V)	98 %	KV	48.74	47.08387	36.57	2		559,6464	1 675,9173	3	-0,002128	-1,269	792,933	28 923,0
(T)PDAVMGNPKVKAHGKK(V)	98 %	KV	48.31	46.83083	43.59	2		838,9676	1 675,9206	2	0,001098	0,6548	792,6	27 657,0
(T)PDAVMGNPKVKAHGKK(V)	97 %	KV	47.96	46.65928	41.02	2		559,6487	1 675,9242	3	0,004682	2,792	1 246,13	36 312,0
(T)PDAVMGNPKVKAHGKK(V)	97 %	KV	47.31	47.084465	47.31	2		838,9657	1 675,9169	2	-0,002582	-1,54	910,326	39 290,0

(T)PDAVmGNPKVKAHGKK(V)	96 %	KV	46.66	47.591915	43.67	2	Oxidation (+16)	846,963	1 691,9115	2	-0,002857	-1,688	675,942	37 944,0
(T)PDAVMGNPKVKAHGKK(V)	95 %	KV	43.98	46.8116	30.69	2		838,9674	1 675,9203	2	0,0007981	0,4759	1 332,07	52 457,0
(T)PDAVmGNPKVKAHGKK(V)	91 %	KV	41.45	47.45894	28.08	2	Oxidation (+16)	564,9786	1 691,9140	3	-0,000383	-0,2262	634,244	31 766,0
(T)PDAVMGNPKVKAHGKKV(L)	100 %	VL	108.04	46.35725	95.09	2		592,6697	1 774,9872	3	-0,0006579	-0,3705	1 366,97	105495
(T)PDAVMGNPKVKAHGKKV(L)	100 %	VL	78.22	46.36949	66.87	2		888,5011	1 774,9876	2	-0,0003019	-0,17	981,382	195407
(T)PDAVMGNPKVKAHGKKV(L)	100 %	VL	75.14	46.340942	68.78	2		444,754	1 774,9867	4	-0,001174	-0,661	1 366,97	41 265,0
(T)PDAVMGNPKVKAHGKKV(L)	100 %	VL	70.61	46.719406	59.58	2		592,6687	1 774,9842	3	-0,003688	-2,077	1 454,35	134453
(T)PDAVMGNPKVKAHGKKV(L)	100 %	VL	65.5	46.360764	58.38	2		888,5007	1 774,9868	2	-0,001022	-0,5754	1 366,97	79 630,0
(T)PDAVMGNPKVKAHGKKV(L)	100 %	VL	58.07	46.710617	46.28	2		592,6683	1 774,9832	3	-0,004678	-2,634	981,382	99 862,0
(T)PDAVmGNPKVKAHGKKV(L)	99 %	VL	56.41	47.514175	42.26	2	Oxidation (+16)	598,002	1 790,9842	3	0,001417	0,7908	1 081,67	35 688,0
(T)PDAVMGNPKVKAHGKKV(L)	99 %	VL	51.24	45.98703	34.04	2		592,6704	1 774,9893	3	0,001412	0,7951	979,371	60 314,0
(T)PDAVMGNPKVKAHGKKV(L)	98 %	VL	48.09	46.36608	41.27	2		888,5009	1 774,9873	2	-0,0005819	-0,3277	980,209	70 900,0
(T)PDAVMGNPKVKAHGKKV(L)	97 %	VL	46.54	46.108624	41.84	2		888,5027	1 774,9908	2	0,002938	1,654	1 454,35	49 607,0
(T)PDAVMGNPKVKAHGKKV(L)	96 %	VL	44.95	45.98856	44.29	2		444,7546	1 774,9893	4	0,001386	0,7804	1 364,97	19 587,0
(T)PDAVMGNPKVKAHGKKV(L)	96 %	VL	44.27	46.35715	26.09	2		592,6697	1 774,9872	3	-0,0006879	-0,3874	659,592	36 454,0
(T)PDAVMGNPKVKAHGKKVL(G)	100 %	LG	68.43	44.500954	61.1	2		630,3658	1 888,0757	3	0,003692	1,954	890,513	79 091,0
(T)PDAVmGNPKVKAHGKKVL(G)	100 %	LG	62.46	45.87307	59.12	2	Oxidation (+16)	635,6948	1 904,0626	3	-0,004313	-2,264	986,56	61 889,0
(T)PDAVMGNPKVKAHGKKVL(G)	100 %	LG	56.73	45.14694	55.29	2		630,3636	1 888,0691	3	-0,002908	-1,539	1 248,63	33 014,0
(T)PDAVmGNPKVKAHGKKVL(G)	99 %	LG	52.75	45.785934	40.34	2	Oxidation (+16)	953,0398	1 904,0650	2	-0,001897	-0,9957	1 383,43	64 745,0
(T)PDAVMGNPKVKAHGKKVL(G)	98 %	LG	46.79	45.390633	41.64	2		945,04	1 888,0654	2	-0,006522	-3,452	1 628,38	35 958,0
(T)PDAVMGNPKVKAHGKKVL(G)	97 %	LG	44.69	44.812565	44.69	2		473,025	1 888,0709	4	-0,001034	-0,5473	890,597	33 742,0
(T)PDAVMGNPKVKAHGKKVL(G)	96 %	LG	42.94	44.514793	37.07	2		630,3657	1 888,0752	3	0,003272	1,732	888,433	45 089,0
(T)PDAVMGNPKVKAHGKKVL(G)	94 %	LG	41.2	44.676968	37.16	2		630,3651	1 888,0735	3	0,001562	0,8269	1 137,67	35 462,0
(T)PDAVMGNPKVKAHGKKVL(G)	94 %	LG	40.89	44.569885	35.99	2		630,3663	1 888,0771	3	0,005132	2,717	891,765	33 586,0
(T)PDAVMGNPKVKAHGKKVL(G)	94 %	LG	40.8	44.680077	36.43	2		630,3645	1 888,0715	3	-0,0004479	-0,2371	1 638,71	36 190,0
(T)PDAVMGNPKVKAHGKKVLG(A)	100 %	GA	104.13	45.426884	99.75	2		649,3724	1 945,0953	3	0,001842	0,9465	1 585,17	114755
(T)PDAVMGNPKVKAHGKKVLG(A)	100 %	GA	94.98	50.023823	90.21	2		973,5526	1 945,0907	2	-0,002742	-1,409	1 585,51	201700
(T)PDAVMGNPKVKAHGKKVLG(A)	100 %	GA	89.5	45.442047	84.39	2		649,3715	1 945,0928	3	-0,0007079	-0,3638	1 587,35	217761
(T)PDAVmGNPKVKAHGKKVLG(A)	100 %	GA	85.83	46.030903	75.62	2	Oxidation (+16)	654,7052	1 961,0937	3	0,005267	2,684	981,465	143767
(T)PDAVmGNPKVKAHGKKVLG(A)	100 %	GA	83.65	45.795094	73.88	2	Oxidation (+16)	654,7063	1 961,0971	3	0,008687	4,427	1 371,62	105218
(T)PDAVMGNPKVKAHGKKVLG(A)	100 %	GA	82.09	45.18711	72.22	2		649,3733	1 945,0979	3	0,004452	2,288	1 624,80	181918
(T)PDAVMGNPKVKAHGKKVLG(A)	100 %	GA	77.15	45.432236	73.37	2		649,3722	1 945,0949	3	0,001422	0,7307	1 620,04	332255
(T)PDAVMGNPKVKAHGKKVLG(A)	100 %	GA	73.41	45.912205	70.21	2		487,2795	1 945,0891	4	-0,004414	-2,268	1 587,35	47 147,0
(T)PDAVMGNPKVKAHGKKVLG(A)	100 %	GA	70.96	45.18409	62.16	2		649,3736	1 945,0989	3	0,005412	2,781	836,929	79 107,0
(T)PDAVmGNPKVKAHGKKVLG(A)	100 %	GA	71.93	46.352623	65.15	2	Oxidation (+16)	654,7035	1 961,0886	3	0,000197	0,1004	977,868	52 864,0
(T)PDAVmGNPKVKAHGKKVLG(A)	100 %	GA	64.3	46.337513	55.16	2	Oxidation (+16)	654,7036	1 961,0889	3	0,000527	0,2686	1 213,68	62 089,0
(T)PDAVmGNPKVKAHGKKVLG(A)	100 %	GA	63.95	46.05682	63.65	2	Oxidation (+16)	654,7052	1 961,0938	3	0,005387	2,746	1 376,60	125755
(T)PDAVMGNPKVKAHGKKVLG(A)	100 %	GA	58.06	45.80001	47.65	2		973,5528	1 945,0910	2	-0,002482	-1,275	1 586,26	54 855,0
(T)PDAVMGNPKVKAHGKKVLG(A)	99 %	GA	56.46	45.768936	53.63	2		649,3714	1 945,0923	3	-0,001188	-0,6104	1 233,14	109850
(T)PDAVMGNPKVKAHGKKVLG(A)	99 %	GA	54.02	45.4449	54.02	2		487,2804	1 945,0927	4	-0,0008139	-0,4182	836,929	67 847,0
(T)PDAVMGNPKVKAHGKKVLG(A)	99 %	GA	52.19	45.81494	49.42	2		487,2799	1 945,0905	4	-0,002934	-1,508	1 586,34	43 107,0
(T)PDAVmGNPKVKAHGKKVLG(A)	99 %	GA	52.06	46.058975	45.54	2	Oxidation (+16)	654,7048	1 961,0926	3	0,004217	2,149	1 379,76	71 008,0
(T)PDAVMGNPKVKAHGKKVLG(A)	99 %	GA	50.53	45.432236	46.33	2		649,3722	1 945,0947	3	0,001212	0,6228	1 123,68	57 635,0
(T)PDAVmGNPKVKAHGKKVLG(A)	98 %	GA	49.4	46.506477	46.75	2	Oxidation (+16)	654,703	1 961,0873	3	-0,001093	-0,557	1 218,17	46 996,0
(T)PDAVmGNPKVKAHGKKVLG(A)	98 %	GA	48.67	46.033066	42.54	2	Oxidation (+16)	654,7052	1 961,0938	3	0,005447	2,776	1 303,15	43 403,0
(T)PDAVMGNPKVKAHGKKVLG(A)	97 %	GA	46.59	45.805717	45.57	2		649,3706	1 945,0900	3	-0,003468	-1,782	1 875,67	32 896,0
(T)PDAVMGNPKVKAHGKKVLG(A)	97 %	GA	46.34	45.925873	43.22	2		487,2797	1 945,0897	4	-0,003774	-1,939	890,513	29 748,0

(T)PDAVMGNPKVKAHGKKVLG(A)	94 %	GA	42.06	45.542587	38.93	2		649,3717	1 945,0932	3	-0,0002879	-0,148	884,683	64 742,0
(T)PDAVMGNPKVKAHGKKVLG(A)	92 %	GA	40.64	45.80001	36.76	2		973,5528	1 945,0910	2	-0,002442	-1,255	1 586,18	76 871,0
(T)PDAVMGNPKVKAHGKKVLG(A)	92 %	GA	39.8	45.426884	39.56	2		649,372	1 945,0941	3	0,0006721	0,3453	1 236,47	25 597,0
(T)PDAVmGNPKVKAHGKKVLG(A)	91 %	GA	39.94	46.058975	38.68	2	Oxidation (+16)	654,7049	1 961,0929	3	0,004487	2,287	1 365,13	40 885,0
(P)DAVMGNPKVKAHGKK(V)	100 %	KV	64.48	46.53367	54.11	2		527,296	1 578,8662	3	-0,0004379	-0,2772	585,833	62 196,0
(P)DAVMGNPKVKAHGKK(V)	100 %	KV	63.55	46.296135	56.13	2		790,4415	1 578,8683	2	0,001678	1,062	1 385,93	111710
(P)DAVMGNPKVKAHGKKVLG(A)	100 %	GA	97.57	45.036003	91.5	2		617,021	1 848,0411	3	0,0004421	0,2391	836,929	153832
(P)DAVMGNPKVKAHGKKVLG(A)	100 %	GA	88.6	45.037636	75.45	2		617,0207	1 848,0403	3	-0,0003679	-0,199	841,6	144058
(P)DAVMGNPKVKAHGKKVLG(A)	100 %	GA	70.56	44.8793	66.25	2		617,0215	1 848,0426	3	0,001942	1,05	838,098	176846
(A)VMGNPKVKAHGKK(V)	98 %	KV	46.32	43.428368	34.94	2		697,4068	1 392,7991	2	-0,003502	-2,513	584,915	114309
(A)VMGNPKVKAHGKK(V)	97 %	KV	43.69	43.49996	39.72	2		697,4062	1 392,7979	2	-0,004682	-3,359	1 387,95	72 219,0
(A)VMGNPKVKAHGKKVL(G)	100 %	LG	96.96	39.36463	86.14	2		803,4877	1 604,9609	2	0,005798	3,61	604,775	581855
(A)VMGNPKVKAHGKKVL(G)	100 %	LG	95.87	43.158028	85.89	2		803,4873	1 604,9600	2	0,004958	3,087	603,694	388910
(A)VMGNPKVKAHGKKVL(G)	100 %	LG	89.92	43.24488	79.05	2		803,4862	1 604,9579	2	0,002798	1,742	603,611	553825
(A)VMGNPKVKAHGKKVL(G)	100 %	LG	86.19	40.71293	78.02	2		803,4836	1 604,9525	2	-0,002522	-1,57	602,528	487319
(A)VMGNPKVKAHGKKVL(G)	100 %	LG	80.62	44.184338	69.69	2		803,4826	1 604,9506	2	-0,004502	-2,803	602,945	460942
(A)VMGNPKVKAHGKKVL(G)	100 %	LG	72.86	43.79614	63.12	2		803,4843	1 604,9541	2	-0,0009619	-0,599	607,101	253060
(A)VmGNPKVKAHGKKVL(G)	100 %	LG	65.75	41.844925	59.83	2	Oxidation (+16)	541,3245	1 620,9516	3	0,001597	0,9846	562,509	186690
(A)VMGNPKVKAHGKKVL(G)	100 %	LG	59.15	39.2947	54.62	2		535,9947	1 604,9621	3	0,007052	4,391	635,163	242217
(A)VMGNPKVKAHGKKVL(G)	99 %	LG	48.12	40.302757	40.72	2		535,9924	1 604,9555	3	0,0003921	0,2441	626,901	74 393,0
(A)VMGNPKVKAHGKKVLG(A)	100 %	GA	87.46	41.420765	72.49	2		831,9954	1 661,9763	2	-0,0002819	-0,1695	602,444	604602
(A)VMGNPKVKAHGKKVLG(A)	100 %	GA	77.78	41.46097	70.84	2		554,9998	1 661,9776	3	0,001002	0,6026	614,083	722347
(A)VmGNPKVKAHGKKVLG(A)	100 %	GA	73.24	42.636124	69.32	2	Oxidation (+16)	560,3321	1 677,9744	3	0,002957	1,761	561,262	231928
(A)VMGNPKVKAHGKKVLG(A)	99 %	GA	51.73	41.4925	45.46	2		554,9991	1 661,9755	3	-0,001098	-0,6602	602,444	246773
(A)VMGNPKVKAHGKKVLG(A)	98 %	GA	45.92	41.748444	38.99	2		554,9983	1 661,9731	3	-0,003498	-2,103	837,013	69 192,0
(A)VmGNPKVKAHGKKVLG(A)	98 %	GA	45.37	43.26008	37.12	2	Oxidation (+16)	839,9906	1 677,9667	2	-0,004757	-2,833	602,611	106050
(A)VMGNPKVKAHGKKVLG(A)	97 %	GA	41.98	41.47738	40.83	2		554,9995	1 661,9766	3	0,00007206	0,04333	1 070,25	30 384,0
(A)VmGNPKVKAHGKKVLG(A)	94 %	GA	38.67	42.8603	37.04	2	Oxidation (+16)	560,3315	1 677,9726	3	0,001157	0,6891	544,962	25 731,0
(A)VmGNPKVKAHGKKVLG(A)	93 %	GA	39.24	43.71364	39.24	2	Oxidation (+16)	560,329	1 677,9652	3	-0,006253	-3,724	616,41	38 342,0
(A)VMGNPKVKAHGKKVLGAFSDG(L)	99 %	GL	52.67	47.410255	49.18	2		714,0628	2 139,1665	3	0,003972	1,856	1 022,19	75 587,0
(A)VMGNPKVKAHGKKVLGAFSDG(L)	97 %	GL	47.23	47.61725	45.27	2		714,0616	2 139,1628	3	0,0002521	0,1178	1 071,33	37 942,0
(A)VMGNPKVKAHGKKVLGAFSDG(L)	93 %	GL	42.54	46.95919	35.85	2		714,0659	2 139,1760	3	0,01339	6,257	1 020,03	52 380,0
(V)MGNPKVKAHGKKV(L)	100 %	VL	68.48	43.49996	54.29	2		697,4062	1 392,7977	2	-0,004822	-3,46	610,672	76 543,0
(V)MGNPKVKAHGKKV(L)	97 %	VL	44.76	43.939262	31.47	2		697,4057	1 392,7967	2	-0,005822	-4,177	615,247	106404
(V)MGNPKVKAHGKKVL(G)	100 %	LG	53.75	41.56125	38.79	2		753,9489	1 505,8833	2	-0,003342	-2,218	602,695	98 346,0
(V)MGNPKVKAHGKKVL(G)	99 %	LG	47.15	41.56125	38.93	2		753,949	1 505,8834	2	-0,003302	-2,191	602,695	204440
(V)MGNPKVKAHGKKVL(G)	93 %	LG	36.55	41.49773	26.56	2		753,9496	1 505,8847	2	-0,001962	-1,302	664,767	60 090,0
(V)MGNPKVKAHGKKVL(G)	93 %	LG	36.55	41.598976	25.77	2		502,9695	1 505,8865	3	-0,0001479	-0,09818	665,685	95 448,0
(V)MGNPKVKAHGKKVLG(A)	100 %	GA	81.61	43.030663	65.99	2		782,4597	1 562,9048	2	-0,003342	-2,137	602,444	404508
(V)MGNPKVKAHGKKVLG(A)	100 %	GA	77.34	42.521973	65.92	2		521,9771	1 562,9094	3	0,001182	0,7558	630,491	240070
(V)MGNPKVKAHGKKVLG(A)	100 %	GA	69.77	42.484634	59.79	2		521,9773	1 562,9100	3	0,001872	1,197	665,435	295519
(V)MGNPKVKAHGKKVLG(A)	100 %	GA	69.62	42.800545	60.73	2		521,9763	1 562,9069	3	-0,001248	-0,798	603,611	326938
(V)MGNPKVKAHGKKVLG(A)	100 %	GA	59.74	42.801456	55.05	2		521,9765	1 562,9077	3	-0,0004379	-0,28	837,013	84 257,0
(V)MGNPKVKAHGKKVLG(A)	100 %	GA	55.78	43.037792	50.23	2		521,9751	1 562,9036	3	-0,004578	-2,927	605,939	260668
(V)MGNPKVKAHGKKVLG(A)	100 %	GA	54.59	42.480713	42.91	2		521,9773	1 562,9102	3	0,002022	1,293	664,266	295637
(V)MGNPKVKAHGKKVLG(A)	99 %	GA	52.52	42.745735	35.88	2		782,4603	1 562,9061	2	-0,002102	-1,344	601,779	320353

(V)MGNPKVKAHGKKVLG(A)	99 %	GA	47.62	43.115204	44.2	2	521,9748	1 562,9027	3	-0,005478	-3,503	602,611	52 408,0
(V)MGNPKVKAHGKKVLG(A)	97 %	GA	43.25	42.800087	30.38	2	782,4616	1 562,9087	2	0,0005381	0,344	626,901	61 896,0
(V)MGNPKVKAHGKKVLG(A)	95 %	GA	40.08	42.480713	30.73	2	521,9775	1 562,9106	3	0,002442	1,562	585,083	60 102,0
(V)MGNPKVKAHGKKVLG(A)	93 %	GA	38.24	43.056305	37.06	2	782,46	1 562,9054	2	-0,002742	-1,753	1 586,43	46 306,0
(V)MGNPKVKAHGKKVLG(A)	92 %	GA	37.32	42.52804	31.57	2	782,4621	1 562,9096	2	0,001478	0,9451	614,249	59 523,0
(M)GNPKVKAHGKKVL(G)	100 %	LG	63.77	37.351994	48.49	2	688,4309	1 374,8473	2	0,001118	0,8126	610,589	511176
(M)GNPKVKAHGKKVL(G)	100 %	LG	53.29	38.436066	37.85	2	688,4281	1 374,8417	2	-0,004442	-3,229	602,528	382303
(M)GNPKVKAHGKKVLG(A)	100 %	GA	71.41	40.86467	55.78	2	716,9405	1 431,8665	2	-0,001142	-0,797	602,528	1143690
(M)GNPKVKAHGKKVLG(A)	100 %	GA	67.61	38.396667	57.04	2	716,9432	1 431,8719	2	0,004218	2,944	603,611	810241
(M)GNPKVKAHGKKVLG(A)	100 %	GA	56.32	40.809868	47.59	2	716,942	1 431,8693	2	0,001678	1,171	604,775	152600
(M)GNPKVKAHGKKVLG(A)	100 %	GA	53.83	38.39855	32.87	2	716,944	1 431,8734	2	0,005758	4,019	602,528	123248
(M)GNPKVKAHGKKVLG(A)	100 %	GA	53.28	38.64036	45.57	2	716,942	1 431,8693	2	0,001678	1,171	612,918	137001
(M)GNPKVKAHGKKVLG(A)	99 %	GA	48.51	38.64036	36.12	2	716,9416	1 431,8685	2	0,0008781	0,6128	1 619,04	34 696,0
(M)GNPKVKAHGKKVLG(A)	99 %	GA	45.34	38.658737	37.77	2	716,9411	1 431,8677	2	0,00001806	0,0126	565,256	17 605,0
(M)GNPKVKAHGKKVLG(A)	99 %	GA	46.16	39.937008	37.25	2	716,9388	1 431,8631	2	-0,004562	-3,184	1 586,34	27 144,0
(M)GNPKVKAHGKKVLG(A)	96 %	GA	37.92	39.737743	32.09	2	716,9399	1 431,8652	2	-0,002482	-1,732	665,435	50 531,0
(N)PKVKAHGKKVL(G)	100 %	LG	72.19	30.979511	59.52	2	602,8974	1 203,7803	2	-0,001482	-1,23	611,754	240792
(N)PKVKAHGKKVL(G)	100 %	LG	70.63	30.979511	56.44	2	602,8966	1 203,7787	2	-0,003082	-2,558	602,528	149958
(N)PKVKAHGKKVL(G)	100 %	LG	56.71	35.92177	42.97	2	602,8978	1 203,7810	2	-0,0007619	-0,6324	623,733	227807
(N)PKVKAHGKKVL(G)	99 %	LG	38.45	31.044872	31.97	2	602,8961	1 203,7777	2	-0,004102	-3,405	605,273	56 257,0
(N)PKVKAHGKKVL(G)	97 %	LG	30.12	29.960737	25.35	2	602,8994	1 203,7842	2	0,002478	2,057	665,936	19 307,0
(N)PKVKAHGKKVLG(A)	100 %	GA	77.57	33.09843	64.99	2	631,4078	1 260,8010	2	-0,002302	-1,824	602,444	469180
(N)PKVKAHGKKVLG(A)	100 %	GA	67.76	33.119656	47.88	2	631,4087	1 260,8029	2	-0,0004019	-0,3185	603,611	82 792,0
(N)PKVKAHGKKVLG(A)	100 %	GA	64.89	32.83301	53.47	2	631,409	1 260,8035	2	0,0002381	0,1887	626,901	160860
(N)PKVKAHGKKVLG(A)	100 %	GA	66.72	35.748413	53.78	2	631,4076	1 260,8007	2	-0,002542	-2,015	605,939	106359
(N)PKVKAHGKKVLG(A)	100 %	GA	53.98	32.484634	43.91	2	631,4101	1 260,8057	2	0,002398	1,9	664,432	53 707,0
(N)PKVKAHGKKVLG(A)	99 %	GA	42.79	33.119656	33.37	2	631,4088	1 260,8030	2	-0,0002219	-0,1759	607,267	8 993,00
(N)PKVKAHGKKVLG(A)	99 %	GA	42.04	33.09843	34.23	2	631,4077	1 260,8009	2	-0,002362	-1,872	634,41	43 368,0
(N)PKVKAHGKKVLG(A)	99 %	GA	41.95	34.52553	33.58	2	631,4065	1 260,7984	2	-0,004902	-3,885	566,089	23 560,0
(N)PKVKAHGKKVLG(A)	99 %	GA	37.68	32.360332	37.68	2	631,4125	1 260,8105	2	0,007218	5,72	837,43	50 334,0
(N)PKVKAHGKKVLG(A)	98 %	GA	37.17	35.046066	37.17	2	631,4027	1 260,7909	2	-0,01236	-9,797	613,167	41 497,0
(N)PKVKAHGKKVLG(A)	97 %	GA	30.15	30.265333	30.15	2	631,4134	1 260,8122	2	0,008958	7,099	663,432	45 882,0
(K)AHGKKVLGAFSDGLAH(L)	100 %	HL	95.99	47.810654	85.73	2	804,4355	1 606,8564	2	-0,001762	-1,096	1 146,98	76 820,0
(K)AHGKKVLGAFSDGLAH(L)	100 %	HL	60.24	47.865883	54.16	2	804,4342	1 606,8539	2	-0,004302	-2,676	1 145,99	32 405,0
(K)AHGKKVLGAFSDGLAH(L)	99 %	HL	56.16	47.759453	48.31	2	804,4357	1 606,8569	2	-0,001282	-0,7973	1 145,65	31 458,0
(K)AHGKKVLGAFSDGLAH(L)	95 %	HL	45.33	47.688747	39.44	2	804,4373	1 606,8601	2	0,001918	1,193	1 171,35	16 305,0
(K)AHGKKVLGAFSDGLAH(L)	92 %	HL	42.61	47.787228	39.62	2	536,6259	1 606,8557	3	-0,002448	-1,522	1 171,35	22 167,0
(H)GKKVLGAFSDGLAHLDN(L)	100 %	NL	62.02	48.26923	52.3	2	871,4674	1 740,9203	2	0,004158	2,387	1 729,80	38 431,0
(H)GKKVLGAFSDGLAHLDN(L)	99 %	NL	62.63	54.002876	45.29	2	871,4668	1 740,9191	2	0,002938	1,687	1 730,97	37 786,0
(K)VLGAFSDGLAHL(D)	100 %	LD	60.32	47.07042	43.35	2	600,3248	1 198,6351	2	0,0003581	0,2985	2 075,15	14 177,0
(K)VLGAFSDGLAHL(D)	95 %	LD	44.69	47.18817	25.41	2	600,3245	1 198,6345	2	-0,0002619	-0,2184	2 074,23	7 049,00
(K)VLGAFSDGLAHLDN(L)	100 %	NL	85.88	48.156578	61.87	2	714,8584	1 427,7022	2	-0,002422	-1,695	1 966,54	296266
(K)VLGAFSDGLAHLDN(L)	100 %	NL	65.04	47.961163	46.08	2	714,858	1 427,7014	2	-0,003282	-2,297	1 965,37	31 592,0
(K)VLGAFSDGLAHLDNLK(G)	100 %	KG	102.99	48.011703	86.8	2	835,4505	1 668,8864	2	0,002678	1,604	1 989,97	26 489,0
(K)VLGAFSDGLAHLDNLK(G)	100 %	KG	102.07	48.05249	85.86	2	835,45	1 668,8854	2	0,001638	0,9809	1 991,05	33 594,0
(K)VLGAFSDGLAHLDNLK(G)	100 %	KG	72.7	48.057457	62.55	2	557,3026	1 668,8859	3	0,002112	1,265	1 991,39	16 309,0

(K)VLGAFSDGLAHLDNLKG(T)	100 %	GT	110.48	48.426216	90.15	2	863,9601	1 725,9056	2	0,0003181	0,1842	1 988,71	168906
(K)VLGAFSDGLAHLDNLKG(T)	100 %	GT	97.25	48.241127	76.87	2	863,9606	1 725,9067	2	0,001418	0,8212	1 987,55	55 757,0
(K)VLGAFSDGLAHLDNLKG(T)	99 %	GT	58.43	48.42628	46.18	2	576,3081	1 725,9024	3	-0,002888	-1,672	1 987,72	32 854,0
(V)LGAFSDGLAH(L)	100 %	HL	65.08	47.58806	40.34	2	494,2462	986,4779	2	-0,004402	-4,458	1 600,26	61 532,0
(V)LGAFSDGLAH(L)	99 %	HL	52.34	47.931194	35.04	2	494,2467	986,4788	2	-0,003482	-3,526	1 597,93	17 015,0
(V)LGAFSDGLAHLDN(L)	100 %	NL	94.2	53.541756	61.81	2	665,3257	1 328,6368	2	0,0005581	0,4197	1 876,75	147294
(V)LGAFSDGLAHLDN(L)	100 %	NL	84.41	48.020622	45.36	2	665,3263	1 328,6380	2	0,001738	1,307	1 877,92	368923
(V)LGAFSDGLAHLDN(L)	100 %	NL	61.0	47.73494	47.0	2	665,3248	1 328,6351	2	-0,001202	-0,904	1 880,18	24 548,0
(V)LGAFSDGLAHLDNLK(G)	100 %	KG	121.37	48.136345	105.33	2	785,9168	1 569,8190	2	0,003678	2,341	1 915,69	78 040,0
(V)LGAFSDGLAHLDNLK(G)	100 %	KG	106.6	48.10387	86.39	2	785,9183	1 569,8220	2	0,006678	4,251	1 916,36	82 537,0
(V)LGAFSDGLAHLDNLKG(T)	100 %	GT	138.84	48.26522	116.13	2	814,4309	1 626,8472	2	0,01036	6,363	1 915,19	230911
(V)LGAFSDGLAHLDNLKG(T)	100 %	GT	122.18	48.39805	106.73	2	814,4304	1 626,8462	2	0,009358	5,749	1 914,11	151894
(V)LGAFSDGLAHLDNLKG(T)	100 %	GT	111.25	48.48423	97.75	2	814,432	1 626,8495	2	0,01266	7,776	1 916,44	46 651,0
(V)LGAFSDGLAHLDNLKG(T)	96 %	GT	46.89	48.30948	39.76	2	543,2859	1 626,8359	3	-0,0009379	-0,5762	1 915,35	15 148,0
(L)GAFSDGLAH(L)	99 %	HL	50.88	45.806396	32.28	2	874,4034	873,3961	1	-0,002036	-2,328	1 122,85	6 674,00
(L)GAFSDGLAH(L)	98 %	HL	50.07	46.204903	32.64	2	874,407	873,3997	1	0,001504	1,72	1 121,43	4 909,00
(L)GAFSDGLAH(L)	93 %	HL	40.6	44.889313	24.07	2	437,7033	873,3921	2	-0,006042	-6,91	1 118,02	19 664,0
(L)GAFSDGLAHLDN(L)	99 %	NL	54.86	45.74008	42.58	2	1 216,5549	1 215,5477	1	-0,004506	-3,704	1 730,97	14 581,0
(L)GAFSDGLAHLDN(L)	99 %	NL	51.27	46.003414	26.15	2	608,784	1 215,5534	2	0,001278	1,051	1 728,55	243187
(L)GAFSDGLAHLDN(L)	96 %	NL	45.23	45.999268	16.94	2	608,7836	1 215,5527	2	0,0005181	0,4258	1 726,22	43 167,0
(L)GAFSDGLAHLDNL(K)	98 %	LK	49.91	47.538357	26.34	2	665,3245	1 328,6345	2	-0,001782	-1,34	2 028,59	39 696,0
(L)GAFSDGLAHLDNL(K)	96 %	LK	45.91	47.697098	24.95	2	665,3247	1 328,6348	2	-0,001502	-1,13	2 029,51	86 592,0
(L)GAFSDGLAHLDNLKG(T)	100 %	GT	133.84	48.193398	111.89	2	757,8823	1 513,7501	2	-0,002662	-1,757	1 821,86	78 230,0
(L)GAFSDGLAHLDNLKG(T)	100 %	GT	130.13	48.144676	110.77	2	757,8825	1 513,7505	2	-0,002242	-1,48	1 851,10	54 640,0
(L)GAFSDGLAHLDNLKG(T)	100 %	GT	140.62	48.428837	124.14	2	757,8848	1 513,7550	2	0,002218	1,464	1 826,53	939276
(L)GAFSDGLAHLDNLKG(T)	100 %	GT	120.37	48.280987	102.79	2	757,8841	1 513,7536	2	0,0008581	0,5665	1 915,19	140353
(L)GAFSDGLAHLDNLKG(T)	100 %	GT	93.64	48.259064	78.9	2	757,8881	1 513,7617	2	0,008898	5,874	1 826,78	80 414,0
(L)GAFSDGLAHLDNLKG(T)	100 %	GT	89.95	48.20208	80.28	2	757,8845	1 513,7545	2	0,001718	1,134	1 988,71	34 345,0
(L)GAFSDGLAHLDNLKG(T)	100 %	GT	86.15	48.335995	72.84	2	757,8879	1 513,7613	2	0,008518	5,623	1 819,53	40 200,0
(L)GAFSDGLAHLDNLKG(T)	100 %	GT	79.3	48.274986	72.18	2	757,8823	1 513,7500	2	-0,002782	-1,837	1 904,92	37 930,0
(L)GAFSDGLAHLDNLKG(T)	100 %	GT	69.5	48.46263	47.19	2	757,8859	1 513,7572	2	0,004478	2,956	1 828,19	55 570,0
(L)GAFSDGLAHLDNLKG(T)	99 %	GT	58.68	48.14221	54.05	2	757,8827	1 513,7508	2	-0,001982	-1,308	1 819,53	15 487,0
(L)GAFSDGLAHLDNLKG(T)	91 %	GT	46.08	52.553715	35.08	2	757,8811	1 513,7477	2	-0,005042	-3,329	1 828,19	28 581,0
(G)AFSDGLAHLDN(L)	99 %	NL	50.45	45.94459	39.28	2	1 159,5343	1 158,5270	1	-0,003646	-3,144	1 672,54	7 687,00
(G)AFSDGLAHLDN(L)	98 %	NL	49.57	46.336197	20.8	2	580,2732	1 158,5318	2	0,001118	0,9642	1 666,72	218450
(G)AFSDGLAHLDN(L)	98 %	NL	49.41	46.221622	28.43	2	1 159,5327	1 158,5254	1	-0,005296	-4,567	1 667,88	24 948,0
(G)AFSDGLAHLDN(L)	97 %	NL	47.18	46.370792	26.28	2	580,2722	1 158,5297	2	-0,0009219	-0,7951	2 393,61	5 089,00
(G)AFSDGLAHLDN(L)	93 %	NL	41.53	45.99064	25.63	2	1 159,5348	1 158,5275	1	-0,003126	-2,696	1 666,80	19 443,0
(G)AFSDGLAHLDNLKG(T)	100 %	GT	112.83	48.31095	95.8	2	729,3734	1 456,7322	2	0,0009581	0,6572	1 778,75	914484
(G)AFSDGLAHLDNLKG(T)	100 %	GT	110.9	48.19564	75.74	2	729,3723	1 456,7300	2	-0,001282	-0,8794	1 775,26	452820
(G)AFSDGLAHLDNLKG(T)	100 %	GT	112.12	54.379677	89.02	2	729,3747	1 456,7348	2	0,003578	2,455	1 779,91	908109
(G)AFSDGLAHLDNLKG(T)	100 %	GT	101.73	48.197742	78.59	2	729,3719	1 456,7292	2	-0,002042	-1,401	1 775,34	64 568,0
(G)AFSDGLAHLDNLKG(T)	100 %	GT	87.81	48.199318	71.12	2	729,3714	1 456,7282	2	-0,003022	-2,073	1 852,44	30 733,0
(G)AFSDGLAHLDNLKG(T)	100 %	GT	69.57	48.19564	46.82	2	729,3722	1 456,7299	2	-0,001362	-0,9343	1 915,27	116919
(G)AFSDGLAHLDNLKG(T)	99 %	GT	57.44	48.3944	45.89	2	729,3726	1 456,7306	2	-0,0006619	-0,4541	1 747,40	15 104,0
(G)AFSDGLAHLDNLKG(T)	99 %	GT	53.32	48.19564	43.77	2	729,3724	1 456,7302	2	-0,001102	-0,7559	1 906,09	24 606,0

(G)AFSDGLAHLDNLKG(T)	98 %	GT	50.99	48.163277	34.28	2	729,371	1 456,7274	2	-0,003822	-2,622	1 880,68	24 392,0
(A)FSDGLAHLDN(L)	99 %	NL	53.14	46.00243	27.14	2	544,7535	1 087,4924	2	-0,001162	-1,067	1 505,74	118261
(A)FSDGLAHLDN(L)	98 %	NL	48.66	45.828587	28.2	2	544,7526	1 087,4906	2	-0,002962	-2,721	1 507,16	18 526,0
(A)FSDGLAHLDN(L)	97 %	NL	46.26	45.828472	24.38	2	544,7522	1 087,4898	2	-0,003722	-3,419	1 530,41	20 529,0
(A)FSDGLAHLDN(L)	95 %	NL	43.15	46.00265	20.81	2	544,7537	1 087,4928	2	-0,0007219	-0,6632	1 666,80	88 111,0
(A)FSDGLAHLDN(L)	93 %	NL	41.33	45.828587	14.83	2	544,7524	1 087,4903	2	-0,003242	-2,978	1 728,55	358280
(A)FSDGLAHLDN(L)	93 %	NL	40.83	45.852013	16.63	2	544,7529	1 087,4912	2	-0,002382	-2,188	1 727,38	92 203,0
(A)FSDGLAHLDN(L)	92 %	NL	40.43	45.85348	15.04	2	544,753	1 087,4914	2	-0,002202	-2,023	1 665,55	35 910,0
(A)FSDGLAHLDNLKGTFAT(L)	100 %	TL	69.65	49.193134	55.78	2	903,9544	1 805,8942	2	-0,0008419	-0,466	1 910,52	130568
(A)FSDGLAHLDNLKGTFAT(L)	94 %	TL	45.68	49.11094	33.25	2	602,9715	1 805,8927	3	-0,002368	-1,31	1 910,68	47 285,0
(F)SDGLAHLDN(L)	97 %	NL	44.93	45.368233	16.52	2	471,2182	940,4218	2	-0,003342	-3,55	932,469	20 751,0
(F)SDGLAHLDN(L)	96 %	NL	44.88	45.547195	16.27	2	471,2184	940,4221	2	-0,003022	-3,21	1 666,80	68 723,0
(F)SDGLAHLDN(L)	95 %	NL	43.47	45.75453	20.46	2	471,2198	940,4251	2	-0,0001019	-0,1083	965,062	128339
(F)SDGLAHLDN(L)	95 %	NL	42.75	45.756958	20.06	2	471,2191	940,4236	2	-0,001582	-1,68	975,539	92 075,0
(F)SDGLAHLDN(L)	92 %	NL	40.5	45.75453	15.8	2	471,2197	940,4249	2	-0,0002819	-0,2995	923,315	21 530,0
(F)SDGLAHLDN(L)	92 %	NL	40.48	45.75407	23.61	2	471,2199	940,4252	2	0,00001806	0,01918	961,572	119718
(F)SDGLAHLDN(L)	92 %	NL	39.93	45.75453	15.26	2	471,2198	940,4251	2	-0,0001019	-0,1083	959,244	13 265,0
(F)SDGLAHLDNLKG(T)	100 %	GT	99.26	53.88861	77.48	2	620,3212	1 238,6279	2	0,002158	1,741	1 378,85	1196720
(F)SDGLAHLDNLKG(T)	100 %	GT	92.62	47.621986	76.71	2	620,3194	1 238,6242	2	-0,001542	-1,244	1 826,53	513332
(F)SDGLAHLDNLKG(T)	100 %	GT	85.32	47.90883	66.15	2	620,3185	1 238,6224	2	-0,003362	-2,712	1 915,19	75 561,0
(F)SDGLAHLDNLKG(T)	100 %	GT	79.68	47.874107	60.31	2	620,3203	1 238,6261	2	0,0003581	0,2888	1 776,50	456419
(F)SDGLAHLDNLKG(T)	100 %	GT	79.57	47.870636	58.15	2	620,3201	1 238,6255	2	-0,0002219	-0,179	1 671,38	198648
(F)SDGLAHLDNLKG(T)	99 %	GT	56.39	47.61988	48.22	2	620,3197	1 238,6248	2	-0,0009219	-0,7437	1 668,05	86 725,0
(F)SDGLAHLDNLKG(T)	97 %	GT	48.65	47.58768	25.15	2	620,3212	1 238,6278	2	0,001998	1,612	1 379,85	32 469,0
(F)SDGLAHLDNLKGTFAT(L)	100 %	TL	106.27	54.77522	95.16	2	830,4216	1 658,8286	2	0,001938	1,168	1 810,29	1164300
(F)SDGLAHLDNLKGTFAT(L)	100 %	TL	88.74	48.899063	72.76	2	830,4192	1 658,8238	2	-0,002882	-1,736	1 977,13	67 495,0
(F)SDGLAHLDNLKGTFAT(L)	100 %	TL	93.58	54.74014	80.17	2	830,4199	1 658,8252	2	-0,001442	-0,8687	1 807,96	184888
(F)SDGLAHLDNLKGTFAT(L)	100 %	TL	82.71	48.7601	68.76	2	830,4185	1 658,8224	2	-0,004222	-2,544	1 808,05	103582
(F)SDGLAHLDNLKGTFAT(L)	100 %	TL	69.47	48.86767	53.33	2	830,419	1 658,8234	2	-0,003262	-1,965	1 978,22	119125
(F)SDGLAHLDNLKGTFAT(L)	100 %	TL	61.73	49.038277	46.93	2	830,4194	1 658,8243	2	-0,002402	-1,447	1 977,05	71 333,0
(S)DGLAHLDNLK(G)	100 %	KG	80.5	47.000114	50.98	2	548,292	1 094,5694	2	-0,002862	-2,612	1 776,50	71 869,0
(S)DGLAHLDNLK(G)	100 %	KG	77.73	46.885532	49.52	2	548,2939	1 094,5733	2	0,001018	0,9292	1 357,90	28 437,0
(S)DGLAHLDNLK(G)	100 %	KG	74.47	46.89016	49.18	2	548,2933	1 094,5721	2	-0,0001619	-0,1478	1 659,79	73 593,0
(S)DGLAHLDNLK(G)	100 %	KG	64.6	47.007988	36.41	2	548,2915	1 094,5684	2	-0,003862	-3,525	1 826,86	44 034,0
(S)DGLAHLDNLK(G)	96 %	KG	45.89	47.007298	32.34	2	548,2918	1 094,5690	2	-0,003222	-2,941	1 536,31	34 639,0
(S)DGLAHLDNLKG(T)	100 %	GT	73.22	47.6006	42.14	2	576,8032	1 151,5919	2	-0,001882	-1,633	1 455,77	25 910,0
(S)DGLAHLDNLKG(T)	100 %	GT	65.65	47.419315	42.15	2	576,8029	1 151,5913	2	-0,002462	-2,136	1 551,22	120196
(S)DGLAHLDNLKG(T)	100 %	GT	64.43	47.369698	39.51	2	576,8024	1 151,5903	2	-0,003462	-3,004	1 424,16	120195
(S)DGLAHLDNLKG(T)	100 %	GT	68.72	51.760826	36.78	2	576,8034	1 151,5923	2	-0,001462	-1,268	1 380,01	92 947,0
(S)DGLAHLDNLKG(T)	100 %	GT	63.4	47.422928	37.7	2	576,803	1 151,5914	2	-0,002342	-2,032	1 670,21	83 333,0
(S)DGLAHLDNLKG(T)	100 %	GT	67.62	51.698948	37.44	2	576,8025	1 151,5904	2	-0,003382	-2,934	1 424,24	305459
(S)DGLAHLDNLKG(T)	100 %	GT	60.3	47.6006	32.5	2	576,8033	1 151,5919	2	-0,001822	-1,581	1 826,70	163052
(S)DGLAHLDNLKG(T)	98 %	GT	55.61	51.626896	39.41	2	576,8038	1 151,5930	2	-0,0007619	-0,6611	1 551,55	27 404,0
(S)DGLAHLDNLKG(T)	98 %	GT	50.15	47.22889	35.85	2	576,8022	1 151,5898	2	-0,003962	-3,437	1 548,98	33 959,0
(S)DGLAHLDNLKGTFAT(L)	100 %	TL	70.56	48.717422	59.34	2	786,9078	1 571,8010	2	0,006338	4,03	1 977,30	48 417,0
(S)DGLAHLDNLKGTFAT(L)	100 %	TL	68.46	48.803963	56.48	2	786,9072	1 571,7998	2	0,005118	3,254	1 841,69	579455

(D)GLAHLDNLK(G)	100 %	KG	59.26	45.202606	29.79	2	490,7788	979,5429	2	-0,002322	-2,368	1 130,51	33 357,0
(D)GLAHLDNLK(G)	99 %	KG	54.36	45.219875	29.2	2	490,7805	979,5463	2	0,001078	1,099	1 358,82	93 593,0
(D)GLAHLDNLK(G)	99 %	KG	53.13	44.88198	28.84	2	490,778	979,5414	2	-0,003842	-3,918	1 776,50	63 853,0
(D)GLAHLDNLK(G)	98 %	KG	47.01	45.302383	26.41	2	490,7798	979,545	2	-0,0002619	-0,2671	1 660,21	38 406,0
(D)GLAHLDNLKG(T)	100 %	GT	57.93	45.933525	35.71	2	519,2893	1 036,5640	2	-0,002742	-2,643	1 379,76	230158
(D)GLAHLDNLKG(T)	100 %	GT	62.67	51.732708	42.91	2	519,2893	1 036,5641	2	-0,002702	-2,604	1 779,91	54 035,0
(D)GLAHLDNLKG(T)	98 %	GT	47.58	45.851673	30.72	2	519,2898	1 036,5651	2	-0,001662	-1,602	1 378,60	39 181,0
(D)GLAHLDNLKG(T)	94 %	GT	42.52	45.93574	29.31	2	519,2892	1 036,5638	2	-0,002962	-2,855	1 826,53	74 029,0
(D)GLAHLDNLKGTFAT(L)	100 %	TL	75.34	48.22273	57.89	2	729,3913	1 456,7680	2	0,0003581	0,2456	1 733,22	85 423,0
(D)GLAHLDNLKGTFAT(L)	100 %	TL	65.74	48.12599	53.69	2	729,392	1 456,7695	2	0,001838	1,261	1 809,38	89 894,0
(G)LAHLDNLKG(T)	99 %	GT	52.44	45.214256	28.42	2	490,7783	979,5421	2	-0,003182	-3,245	1 008,22	106808
(G)LAHLDNLKG(T)	97 %	GT	44.62	45.214256	23.89	2	490,7783	979,5421	2	-0,003182	-3,245	1 380,26	24 548,0
(G)LAHLDNLKG(T)	94 %	GT	40.9	44.900852	18.35	2	490,7774	979,5402	2	-0,005022	-5,122	1 001,24	439279
(G)LAHLDNLKG(T)	94 %	GT	40.6	44.87223	17.27	2	490,7778	979,541	2	-0,004262	-4,346	989,567	26 938,0
(G)LAHLDNLKGTFAT(L)	100 %	TL	69.24	47.827522	49.48	2	700,8805	1 399,7464	2	0,0002781	0,1985	1 660,87	473563
(G)LAHLDNLKGTFAT(L)	100 %	TL	59.62	47.82179	43.49	2	700,8805	1 399,7465	2	0,0003581	0,2556	1 659,87	72 852,0
(G)LAHLDNLKGTFAT(L)	99 %	TL	54.58	47.842896	41.59	2	700,8808	1 399,7471	2	0,0009381	0,6697	1 659,79	189231
(A)HLDNLKGTFAT(L)	99 %	TL	56.9	48.215263	26.62	2	608,819	1 215,6235	2	-0,001442	-1,185	1 661,12	58 317,0
(A)HLDNLKGTFAT(L)	96 %	TL	47.49	48.236176	28.65	2	608,8228	1 215,6311	2	0,006118	5,029	1 289,19	46 027,0
(C)DKLHVDPENFRLLG(N)	97 %	GN	49.36	48.27595	29.55	2	551,6279	1 651,8619	3	-0,006598	-3,992	1 900,01	32 909,0
(C)DKLHVDPENFRLLG(N)	97 %	GN	49.04	47.985195	23.1	2	826,9427	1 651,8708	2	0,002358	1,427	1 900,01	37 420,0
(D)KLHVDPENFR(L)	97 %	RL	48.33	47.45918	23.42	2	627,8325	1 253,6505	2	-0,001302	-1,038	1 586,34	67 271,0
(D)KLHVDPENFR(L)	91 %	RL	41.25	47.160202	16.86	2	627,8333	1 253,6521	2	0,0003581	0,2854	1 056,21	18 154,0
(H)VDPENFRLLG(N)	98 %	GN	49.93	48.250816	27.21	2	580,3074	1 158,6002	2	-0,003262	-2,813	1 952,53	124778
(H)VDPENFRLLG(N)	98 %	GN	49.82	48.24718	27.04	2	580,3074	1 158,6003	2	-0,003142	-2,709	1 951,45	46 675,0
(G)KEFTPPVQAAYQK(V)	100 %	KV	79.35	48.13561	59.98	2	753,9019	1 505,7891	2	0,001178	0,7818	1 214,34	192270
(G)KEFTPPVQAAYQK(V)	94 %	KV	44.12	48.338287	31.3	2	753,9009	1 505,7872	2	-0,0008019	-0,5322	1 210,85	43 990,0
(G)KEFTPPVQAAYQKVV(A)	100 %	VA	89.97	50.632282	76.28	2	852,9713	1 703,9281	2	0,003298	1,934	1 722,97	135622
(G)KEFTPPVQAAYQKVV(A)	100 %	VA	78.58	47.321846	63.33	2	852,9708	1 703,9269	2	0,002178	1,278	1 720,64	45 626,0
(G)KEFTPPVQAAYQKVVA(G)	100 %	AG	117.44	47.518024	105.23	2	888,4902	1 774,9659	2	0,004038	2,274	1 725,05	805019
(G)KEFTPPVQAAYQKVVA(G)	100 %	AG	92.53	47.52148	82.64	2	888,4897	1 774,9649	2	0,002998	1,688	1 719,22	157416
(G)KEFTPPVQAAYQKVVA(G)	93 %	AG	43.23	47.78325	36.84	2	888,4881	1 774,9616	2	-0,0002219	-0,125	1 714,63	33 933,0
(G)KEFTPPVQAAYQKVVAGVANA(L)	100 %	AL	92.32	48.49426	82.71	2	730,0631	2 187,1674	3	-0,001428	-0,6526	1 972,38	30 821,0
(G)KEFTPPVQAAYQKVVAGVANA(L)	93 %	AL	43.4	48.45222	36.76	2	730,0636	2 187,1689	3	0,00004206	0,01922	1 971,22	27 648,0
(F)TPPVQAAYQKVVAG(V)	100 %	GV	68.76	47.376217	56.93	2	714,896	1 427,7775	2	0,0001581	0,1106	1 677,20	193333
(F)TPPVQAAYQKVVAG(V)	100 %	GV	63.1	47.44434	47.7	2	714,8968	1 427,7790	2	0,001658	1,16	1 675,21	89 397,0
(T)PPVQAAYQKVVA(G)	100 %	AG	59.64	46.14739	40.25	2	635,8607	1 269,7068	2	-0,001342	-1,056	1 855,68	88 353,0
(T)PPVQAAYQKVVA(G)	96 %	AG	45.52	46.14739	27.67	2	635,8609	1 269,7072	2	-0,0009819	-0,7727	1 726,72	24 724,0
(T)PPVQAAYQKVVAG(V)	100 %	GV	69.67	51.88532	50.48	2	664,3703	1 326,7261	2	-0,003542	-2,668	1 888,67	140065
(T)PPVQAAYQKVVAG(V)	98 %	GV	48.96	47.235622	34.91	2	664,3679	1 326,7213	2	-0,008362	-6,298	1 888,34	130948
(V)QAAYQKVVAGVANALAHK(Y)	100 %	KY	82.73	46.220276	74.78	2	613,6805	1 838,0197	3	0,003462	1,883	1 876,00	47 992,0
(V)QAAYQKVVAGVANALAHK(Y)	100 %	KY	82.78	46.473827	70.32	2	613,6795	1 838,0167	3	0,0004021	0,2186	1 876,67	49 747,0
(Q)AAYQKVVAGVANALAH(K)	100 %	HK	128.24	46.871902	115.3	2	791,939	1 581,8635	2	0,0008181	0,5168	1 865,09	177415
(Q)AAYQKVVAGVANALAH(K)	100 %	HK	88.75	47.14363	77.53	2	791,9356	1 581,8567	2	-0,005962	-3,767	1 864,34	115790
(A)AYQKVVAGVANALAH(K)	100 %	HK	75.5	46.621155	56.81	2	756,4214	1 510,8282	2	0,002678	1,771	1 699,47	42 070,0
(A)AYQKVVAGVANALAH(K)	100 %	HK	60.4	46.806797	40.57	2	756,4173	1 510,8201	2	-0,005442	-3,6	1 617,96	24 435,0

(K)VVAGVANALAHK(Y)	100 %	KY	82.21	43.02504	59.21	2	575,3393	1 148,6640	2	-0,002562	-2,228	1 151,47	99 274,0
(K)VVAGVANALAHK(Y)	100 %	KY	81.96	43.02504	56.94	2	575,3393	1 148,6640	2	-0,002582	-2,246	1 149,15	47 476,0
(K)VVAGVANALAHK(Y)	97 %	KY	42.31	42.788906	24.39	2	575,3407	1 148,6669	2	0,0003181	0,2767	1 617,96	21 237,0
(K)VVAGVANALAHK(Y)	96 %	KY	41.29	43.02244	31.44	2	575,339	1 148,6635	2	-0,003102	-2,698	1 786,25	24 332,0
(V)VAGVANALAHK(Y)	100 %	KY	55.37	44.01004	29.36	2	525,8042	1 049,5938	2	-0,004322	-4,114	1 150,31	18 665,0
(V)VAGVANALAHK(Y)	95 %	KY	41.53	44.18235	23.94	2	525,8064	1 049,5982	2	-1,942E-06	-0,001848	927,806	20 280,0
(G)VANALAHK(Y)	99 %	KY	68.03	42.391243	38.81	2	412,2439	822,4733	2	0,002158	2,621	531,006	80 399,0
(G)VANALAHK(Y)	99 %	KY	67.77	43.728752	38.71	2	412,2421	822,4696	2	-0,001602	-1,945	506,592	24 531,0
(G)VANALAHK(Y)	99 %	KY	66.63	44.076797	39.07	2	412,2425	822,4705	2	-0,0006819	-0,8281	335,85	4 182,00
(G)VANALAHK(Y)	99 %	KY	51.56	44.076797	34.3	2	412,2429	822,4713	2	0,00009806	0,1191	674,109	9 015,00
(G)VANALAHK(Y)	99 %	KY	49.67	43.728752	30.29	2	412,2412	822,4678	2	-0,003342	-4,058	1 785,83	7 241,00
(G)VANALAHK(Y)	99 %	KY	49.63	44.076797	29.82	2	412,2423	822,4701	2	-0,001062	-1,29	333,857	2 211,00
(G)VANALAHK(Y)	98 %	KY	47.59	43.728752	31.02	2	412,2419	822,4692	2	-0,002002	-2,431	482,18	12 250,0
(G)VANALAHK(Y)	98 %	KY	47.35	44.076797	29.09	2	412,2423	822,4701	2	-0,001062	-1,29	384,514	9 159,00
(G)VANALAHK(Y)	98 %	KY	46.89	44.076797	29.21	2	412,2424	822,4702	2	-0,0009219	-1,12	457,76	7 066,00
(G)VANALAHK(Y)	98 %	KY	45.88	43.686214	31.17	2	412,2395	822,4645	2	-0,006662	-8,09	649,255	10 674,0
(G)VANALAHK(Y)	93 %	KY	39.27	43.728752	24.64	2	412,2415	822,4685	2	-0,002682	-3,257	1 619,38	8 329,00
