# Supplementary material for: Unveiling the Peptidase Network Orchestrating Hemoglobin Catabolism in Rhodnius prolixus
Source: Mol Cell Proteomics. 2024 Apr 23;23(6):100775. doi: 10.1016/j.mcpro.2024.100775 (PMC11135036; doi:10.1016/j.mcpro.2024.100775)
Supplement: Supplemental Table S9 [file mmc9.pdf]

Table S9: Relative expression intensity of A1 aspartic proteases quantified by proteomics

Uniprot Ids	0			6 h			24 h			48 h			7 d			14 d		
	Log intensity	somme intensity	Log relative intensity	Log intensity	somme intensity	Log relative intensity	Log intensity	somme intensity	Log relative intensity	Log intensity	somme intensity	Log relative intensity	Log intensity	somme intensity	Log relative intensity	Log intensity	somme intensity	Log relative intensity
R4G5J4	24,8727	51324,4365	0,00048462	31,8862	51324,4365	0,00062127	19,57033	49446,9707	0,00039578	17,3737	11422,6051	0,001520993	0	5446,95197	0	20,935	21507,1451	0,000973397
R4FPI4	22,97747	51324,4365	0,00044769	29,88957	51324,4365	0,00058237	22,07563	49446,9707	0,00044645	17,3737	11422,6051	0,001520993	0	5446,95197	0	20,935	21507,1451	0,000973397
R4G5J6	0	51324,4365	0	28,2682	51324,4365	0,00055077	22,07563	49446,9707	0,00044645	17,3737	11422,6051	0,001520993	0	5446,95197	0	20,935	21507,1451	0,000973397
T1IFK7	0	51324,4365	0	28,2682	51324,4365	0,00055077	22,07563	49446,9707	0,00044645	17,3737	11422,6051	0,001520993	0	5446,95197	0	20,935	21507,1451	0,000973397
R4G4V2	31,21937	51324,4365	0,00060827	32,49683	51324,4365	0,00063316	26,64073	49446,9707	0,00053877	19,5495	11422,6051	0,001711475	12,6276	5446,95197	0,00231829	20,6397	21507,1451	0,000959667
T1HPQ4	31,21937	51324,4365	0,00060827	32,49683	51324,4365	0,00063316	26,64073	49446,9707	0,00053877	19,5495	11422,6051	0,001711475	12,6276	5446,95197	0,00231829	20,6397	21507,1451	0,000959667
R4G3V2	0	51324,4365	0	0	51324,4365	0	18,3756	49446,9707	0,00037162	14,2518	11422,6051	0,001247684	0	5446,95197	0	0	21507,1451	0
R4FNG1	27,88603	51324,4365	0,00054333	32,12923	51324,4365	0,000626	18,5388	49446,9707	0,00037492	16,9258	11422,6051	0,001481781	11,103	5446,95197	0,00203839	23,4779	21507,1451	0,001091633
T1HY69	27,88603	51324,4365	0,00054333	32,12923	51324,4365	0,000626	0	49446,9707	0	16,9258	11422,6051	0,001481781	11,103	5446,95197	0,00203839	23,4779	21507,1451	0,001091633
T1IEM8	27,88603	51324,4365	0,00054333	32,12923	51324,4365	0,000626	0	49446,9707	0	16,9258	11422,6051	0,001481781	11,103	5446,95197	0,00203839	23,4779	21507,1451	0,001091633
T1I865	27,88603	51324,4365	0,00054333	32,12923	51324,4365	0,000626	0	49446,9707	0	16,9258	11422,6051	0,001481781	11,103	5446,95197	0,00203839	23,4779	21507,1451	0,001091633
T1I633	27,88603	51324,4365	0,00054333	32,12923	51324,4365	0,000626	0	49446,9707	0	16,9258	11422,6051	0,001481781	11,103	5446,95197	0,00203839	23,4779	21507,1451	0,001091633
T1I882	22,9668	51324,4365	0,00044748	23,97835	51324,4365	0,00046719	18,5388	49446,9707	0,00037492	0	11422,6051	0	0	5446,95197	0	16,3414	21507,1451	0,000759813
R4FKP9	27,1831	51324,4365	0,00052963	28,25047	51324,4365	0,00055043	22,3348	49446,9707	0,00045169	16,4009	11422,6051	0,001435828	0	5446,95197	0	18,0007	21507,1451	0,000836964
T1HJV8	27,1831	51324,4365	0,00052963	28,25047	51324,4365	0,00055043	22,3348	49446,9707	0,00045169	16,4009	11422,6051	0,001435828	0	5446,95197	0	18,0007	21507,1451	0,000836964
T1I3T5	27,1831	51324,4365	0,00052963	28,25047	51324,4365	0,00055043	22,3348	49446,9707	0,00045169	16,4009	11422,6051	0,001435828	0	5446,95197	0	18,0007	21507,1451	0,000836964
R4FJC3	24,199	51324,4365	0,00047149	26,78917	51324,4365	0,00052196	22,044	49446,9707	0,00044581	13,3741	11422,6051	0,001170845	0	5446,95197	0	15,9635	21507,1451	0,000742242
T1HRT9	24,199	51324,4365	0,00047149	26,78917	51324,4365	0,00052196	22,044	49446,9707	0,00044581	13,3741	11422,6051	0,001170845	0	5446,95197	0	15,9635	21507,1451	0,000742242
T1I914	28,0241	51324,4365	0,00054602	29,37557	51324,4365	0,00057235	24,95965	49446,9707	0,00050478	14,2822	11422,6051	0,001250345	0	5446,95197	0	16,2571	21507,1451	0,000755893
T1HQG6	31,21937	51324,4365	0,00060827	32,49683	51324,4365	0,00063316	26,64073	49446,9707	0,00053877	0	11422,6051	0	0	5446,95197	0	0	21507,1451	0
T1I913	26,9706	51324,4365	0,00052549	0	51324,4365	0	24,89075	49446,9707	0,00050338	0	11422,6051	0	0	5446,95197	0	0	21507,1451	0
R4FP07	26,9706	51324,4365	0,00052549	0	51324,4365	0	24,89075	49446,9707	0,00050338	0	11422,6051	0	0	5446,95197	0	0	21507,1451	0
R4FNN7	26,9706	51324,4365	0,00052549	0	51324,4365	0	24,89075	49446,9707	0,00050338	0	11422,6051	0	0	5446,95197	0	0	21507,1451	0
T1HEK6	0	51324,4365	0	0	51324,4365	0	18,3756	49446,9707	0,00037162	0	11422,6051	0	0	5446,95197	0	0	21507,1451	0
T1HEK7	0	51324,4365	0	0	51324,4365	0	18,3756	49446,9707	0,00037162	0	11422,6051	0	0	5446,95197	0	0	21507,1451	0
T1HJE8	0	51324,4365	0	27,0896	51324,4365	0,00052781	0	49446,9707	0	0	11422,6051	0	0	5446,95197	0	0	21507,1451	0
R4FP52	22,97747	51324,4365	0,00044769	29,88957	51324,4365	0,00058237	0	49446,9707	0	0	11422,6051	0	0	5446,95197	0	0	21507,1451	0
