# Supplementary material for: Unveiling the Peptidase Network Orchestrating Hemoglobin Catabolism in Rhodnius prolixus
Source: Mol Cell Proteomics. 2024 Apr 23;23(6):100775. doi: 10.1016/j.mcpro.2024.100775 (PMC11135036; doi:10.1016/j.mcpro.2024.100775)
Supplement: Supplemental Table S10 [file mmc10.pdf]

**Table S10:** ***In vivo*** **rabbit hemoglobin digest identified by mass spectrometry within the digestive content of the AM after 14 days post-feeding.**

| Sequence | Number of identification | Lenght (AA) | Prob | Mascot Ion score | Mascot Identi | Mascot Delta | NTT | Modifications | Observed | Actual Mass | Charge | Delta Da | Delta PPM | Retention Tim | TIC | Start | Stop |
|---|---|---|---|---|---|---|---|---|---|---|---|---|---|---|---|---|---|
| **Alpha-subunit** | | | | | | | | | | | | | | | | | |
| (M)VLSPADKTNIKTAWEKIGSHGGEYGAEAVERMF(L) | 15 | 33 | 100 % | 149.73 | 51.56622 | 146.34 | 2 | | 898,7061 | 3 590,7951 | 4 | 0,01203 | 3,348 | 1 878,22 | 369424 | 2 | 34 |
| (M)VLSPADKTNIKTAWEKIGSHGGEYGAEAVER(M) | 10 | 31 | 100 % | 204.31 | 51.398094 | 200.89 | 2 | | 663,5429 | 3 312,6783 | 5 | 0,00415 | 1,252 | 1 515,73 | 252193 | 2 | 32 |
| (M)VLSPADKTNIKTAWEKIGSHGGEYGAEAVERM(F) | 10 | 32 | 100 % | 138.02 | 51.466736 | 132.37 | 2 | | 861,9372 | 3 443,7197 | 4 | 0,005066 | 1,471 | 1 643,10 | 318400 | 2 | 33 |
| (L)SPADKTNIKTAWEKIGSHGGEY(G) | 7 | 22 | 100 % | 101.34 | 50.20974 | 97.6 | 2 | | 797,0651 | 2 388,1734 | 3 | 0,002142 | 0,8966 | 1 328,64 | 124001 | 4 | 25 |
| (L)SPADKTNIKTAWEKIG(S) | 13 | 16 | 100 % | 121.11 | 48.98731 | 104.26 | 2 | | 879,9747 | 1 757,9348 | 2 | 0,003318 | 1,886 | 1 496,93 | 427843 | 4 | 19 |
| (L)SPADKTNIKTAWEKI(G) | 11 | 15 | 100 % | 102.43 | 48.507076 | 80.67 | 2 | | 851,4643 | 1 700,9141 | 2 | 0,004138 | 2,431 | 1 520,32 | 519598 | 4 | 18 |
| (L)SPADKTNIKTAWE(K) | 8 | 13 | 100 % | 88.29 | 49.013657 | 63.82 | 2 | | 730,8721 | 1 459,7297 | 2 | -0,001142 | -0,7818 | 1 366,06 | 255468 | 4 | 16 |
| (L)SPADKTNIKTA(W) | 9 | 11 | 100 % | 74.12 | 48.789467 | 44.77 | 2 | | 573,3108 | 1 144,6071 | 2 | -0,001902 | -1,66 | 740,74 | 221383 | 4 | 14 |
| (L)SPADKTNIKT(A) | 11 | 10 | 100 % | 70.38 | 48.52474 | 21.07 | 2 | | 537,7928 | 1 073,5709 | 2 | -0,0009219 | -0,858 | 732,581 | 331214 | 4 | 13 |
| (F)LGFPTTKTYFPHFD(F) | 7 | 14 | 100 % | 90.28 | 48.85344 | 80.46 | 2 | | 835,9158 | 1 669,8171 | 2 | 0,002818 | 1,687 | 1 886,41 | 817594 | 35 | 48 |
| (P)TTKTYFPH(F) | 10 | 8 | 63.99 | 48.352276 | 35.77 | 2 | | 497,7509 | 993,4872 | 2 | -0,004882 | -4,909 | 618,883 | | 39 | 46 | 0 |
| (F)LGFPTTKTYFPHF(D) | 11 | 13 | 100 % | 112.17 | 48.943882 | 95.14 | 2 | | 519,2693 | 1 554,7861 | 3 | -0,001138 | -0,7314 | 1 937,86 | 184741 | 35 | 47 |
| (L)GFPTTKTYFPHF(D) | 7 | 12 | 100 % | 89.09 | 48.563423 | 73.18 | 2 | | 721,8594 | 1 441,7042 | 2 | 0,001018 | 0,7057 | 1 753,04 | 188285 | 36 | 47 |
| (L)GFPTTKTYFPH(F) | 7 | 11 | 100 % | 69.54 | 48.5808 | 54.98 | 2 | | 648,324 | 1 294,6335 | 2 | -0,001302 | -1,005 | 1 326,39 | 48 726,0 | 36 | 46 |
| (G)FPTTKTYFPHF(D) | 15 | 11 | 100 % | 80.64 | 48.38194 | 59.41 | 2 | | 693,3471 | 1 384,6797 | 2 | -0,001982 | -1,43 | 1 720,27 | 98 973,0 | 37 | 47 |
| (F)PTTKTYFPHFDF(T) | 15 | 12 | 100 % | 84.01 | 48.02897 | 70.42 | 2 | | 750,8618 | 1 499,7090 | 2 | 0,0003381 | 0,2253 | 1 839,61 | 890631 | 38 | 49 |
| (F)THGSEQIKAHGKKVSEALTKA(V) | 11 | 21 | 100 % | 160.58 | 48.042892 | 149.34 | 2 | | 740,7434 | 2 219,2083 | 3 | 0,005872 | 2,645 | 506,473 | 777899 | 50 | 70 |
| (T)HGSEQIKAHGKKVSEALTKA(V) | 9 | 20 | 100 % | 120.7 | 48.26897 | 109.22 | 2 | | 530,5454 | 2 118,1526 | 4 | -0,002194 | -1,035 | 466,826 | 336505 | 51 | 70 |
| (H)GSEQIKAHGKKVSEALTKA(V) | 8 | 19 | 100 % | 150.37 | 47.23497 | 139.44 | 2 | | 661,3724 | 1 981,0954 | 3 | -0,0004979 | -0,2512 | 500,635 | 422401 | 52 | 70 |
| (K)AHGKKVSEALTKA(V) | 7 | 13 | 100 % | 79.89 | 44.824593 | 65.03 | 2 | | 670,3882 | 1 338,7619 | 2 | -0,0001619 | -0,1209 | 504,221 | 137545 | 58 | 70 |
| (A)VGHLDDLPGALSTLSDLHAHKL(R) | 12 | 22 | 100 % | 145.47 | 49.583534 | 130.9 | 2 | | 770,4118 | 2 308,2134 | 3 | -0,004548 | -1,969 | 1 887,83 | 110873 | 71 | 92 |
| (H)LDDLPGALSTLSDLHAHKL(R) | 7 | 19 | 100 % | 89.5 | 49.421967 | 76.46 | 2 | | 672,6958 | 2 015,0654 | 3 | -0,003748 | -1,859 | 1 930,84 | 49 219,0 | 74 | 92 |
| (L)DDLPGALSTLSDLHAHKL(R) | 10 | 18 | 100 % | 125.11 | 49.68039 | 105.89 | 2 | | 951,9987 | 1 901,9828 | 2 | -0,002282 | -1,199 | 1 856,16 | 32 832,0 | 75 | 92 |
| (L)PGALSTLSDLHAHKL(R) | 9 | 15 | 100 % | 131.02 | 48.313385 | 111.19 | 2 | | 780,4288 | 1 558,8431 | 2 | -0,003902 | -2,501 | 1 866,60 | 63 531,0 | 78 | 92 |
| (A)LSTLSDLHAHKL(R) | 7 | 12 | 100 % | 93.75 | 46.737118 | 66.88 | 2 | | 667,8751 | 1 333,7357 | 2 | 0,0001381 | 0,1034 | 989,164 | 323584 | 81 | 92 |
| (L)STLSDLHAHKL(R) | 12 | 11 | 100 % | 88.03 | 47.924686 | 58.8 | 2 | | 611,3309 | 1 220,6473 | 2 | -0,004182 | -3,423 | 1 456,19 | 38 726,0 | 82 | 92 |
| (S)TLSDLHAHKL(R) | 8 | 10 | 100 % | 68.03 | 47.190407 | 44.57 | 2 | | 567,817 | 1 133,6194 | 2 | -0,0001019 | -0,08985 | 683,63 | 212127 | 83 | 92 |
| (T)LSDLHAHKL(R) | 15 | 9 | 100 % | 66.18 | 47.2574 | 40.26 | 2 | | 517,2927 | 1 032,5709 | 2 | -0,0008819 | -0,8533 | 496,052 | 143770 | 84 | 92 |
| (L)SDLHAHKL(R) | 16 | 8 | 98 % | 49.76 | 47.243744 | 14.51 | 2 | | 460,7492 | 919,4838 | 2 | -0,003822 | -4,152 | 1 456,02 | 42 160,0 | 85 | 92 |
| (L)RVDPVNFKLLSH(C) | 8 | 12 | 100 % | 79.94 | 45.902843 | 57.08 | 2 | | 712,9036 | 1 423,7926 | 2 | -0,001102 | -0,7734 | 1 479,48 | 119757 | 93 | 104 |
| (L)RVDPVNFK(L) | 10 | 8 | 99 % | 55.16 | 48.40746 | 31.13 | 2 | | 487,7737 | 973,5328 | 2 | -0,001782 | -1,828 | 718,597 | 124527 | 93 | 100 |
| (V)DPVNFKLL(S) | 15 | 8 | 97 % | 45.5 | 47.69333 | 19.13 | 2 | | 473,2723 | 944,5301 | 2 | -0,003162 | -3,344 | 1 897,10 | 17 148,0 | 95 | 102 |
| (L)LVTLANHHPSEFTPAVHASLDKF(L) | 14 | 23 | 100 % | 156.94 | 50.486164 | 147.01 | 2 | | 844,4411 | 2 530,3014 | 3 | 0,004442 | 1,755 | 1 591,74 | 519967 | 107 | 129 |
| (L)LVTLANHHPSEFTPAVHASLDK(F) | 7 | 22 | 100 % | 110.19 | 50.145794 | 97.6 | 2 | | 795,4169 | 2 383,2288 | 3 | 0,0002121 | 0,08894 | 1 390,60 | 247220 | 107 | 128 |
| (L)VTLANHHPSEFTPAVHASLDKF(L) | 19 | 22 | 100 % | 147.37 | 50.44806 | 137.92 | 2 | | 806,7446 | 2 417,2118 | 3 | -0,001048 | -0,4334 | 1 499,35 | 318627 | 108 | 129 |
| (V)TLANHHPSEFTPAVHASLDKF(L) | 17 | 21 | 100 % | 107.77 | 50.18097 | 98.1 | 2 | | 773,7226 | 2 318,1459 | 3 | 0,001382 | 0,5959 | 1 592,82 | 333252 | 109 | 129 |
| (T)LANHHPSEFTPAVHASLDKF(L) | 45 | 20 | 100 % | 128.9 | 49.983986 | 113.93 | 2 | | 740,0388 | 2 217,0944 | 3 | -0,002348 | -1,059 | 1 377,76 | 924631 | 110 | 129 |
| (L)ANHHPSEFTPAVHASLDKF(L) | 39 | 19 | 100 % | 108.44 | 49.790653 | 97.4 | 2 | | 1 053,0211 | 2 104,0276 | 2 | 0,01496 | 7,106 | 1 281,94 | 472318 | 111 | 129 |
| (A)NHHPSEFTPAVHASLDKF(L) | 30 | 18 | 100 % | 95.45 | 49.434547 | 77.43 | 2 | | 678,6673 | 2 032,9800 | 3 | 0,004472 | 2,199 | 1 308,78 | 353830 | 112 | 129 |
| (N)HHPSEFTPAVHASLDKF(L) | 21 | 17 | 100 % | 96.37 | 49.529617 | 82.6 | 2 | | 640,6515 | 1 918,9328 | 3 | 0,00009206 | 0,04795 | 1 335,66 | 92 240,0 | 113 | 129 |
| (H)HPSEFTPAVHASLDKF(L) | 12 | 16 | 100 % | 94.98 | 49.141315 | 80.14 | 2 | | 891,9427 | 1 781,8708 | 2 | -0,003002 | -1,684 | 1 592,91 | 61 007,0 | 114 | 129 |
| (H)PSEFTPAVHASLDKF(L) | 39 | 15 | 100 % | 116.88 | 49.12599 | 101.22 | 2 | | 823,4131 | 1 644,8116 | 2 | -0,003222 | -1,958 | 1 280,78 | 153390 | 115 | 129 |
| (H)HPSEFTPAVH(A) | 10 | 10 | 100 % | 68.26 | 48.306335 | 48.27 | 2 | | 561,271 | 1 120,5274 | 2 | -0,002782 | -2,48 | 572,933 | 36 150,0 | 114 | 123 |
| (H)PSEFTPAVH(A) | 17 | 9 | 100 % | 71.42 | 47.16087 | 38.01 | 2 | | 492,7415 | 983,4683 | 2 | -0,002922 | -2,968 | 583,5 | 46 818,0 | 115 | 123 |
| (F)LANVSTVLTSKYR(-) | 14 | 13 | 100 % | 101.05 | 46.153397 | 86.67 | 2 | | 484,6103 | 1 450,8092 | 3 | -0,005178 | -3,567 | 1 388,26 | 81 223,0 | 130 | 142 |
| (L)ANVSTVLTSKYR(-) | 10 | 12 | 100 % | 101.0 | 46.987267 | 79.79 | 2 | | 669,8719 | 1 337,7292 | 2 | -0,001102 | -0,8231 | 978,666 | 337369 | 131 | 142 |
| (A)NVSTVLTSKYR(-) | 14 | 11 | 100 % | 78.29 | 46.573524 | 53.74 | 2 | | 634,3545 | 1 266,6944 | 2 | 0,001218 | 0,9608 | 1 385,93 | 307233 | 132 | 142 |
| (N)VSTVLTSKYR(-) | 10 | 10 | 100 % | 78.77 | 45.24071 | 56.33 | 2 | | 577,3315 | 1 152,6485 | 2 | -0,001742 | -1,51 | 978,833 | 49 645,0 | 133 | 142 |
| (V)STVLTSKYR(-) | 16 | 9 | 100 % | 80.38 | 45.9479 | 46.65 | 2 | | 527,7968 | 1 053,5791 | 2 | -0,002782 | -2,638 | 980,999 | 124658 | 134 | 142 |
| (S)TVLTSKYR(-) | 8 | 8 | 99 % | 67.88 | 44.35255 | 36.33 | 2 | | 484,2821 | 966,5496 | 2 | -0,0002419 | -0,2501 | 497,417 | 220243 | 135 | 142 |

| | | | | | | | | | | | | | | | | | |
|---|---|---|---|---|---|---|---|---|---|---|---|---|---|---|---|---|---|
| **Beta-subunit** | | | | | | | | | | | | | | | | | |
| (M)VHLSSEEKSA(V) | 12 | 10 | 100 % | 65.93 | 47.47357 | 43.67 | 2 | | 543,7741 | 1 085,5337 | 2 | -0,001582 | -1,456 | 359,677 | 92 838,0 | 2 | 11 |
| (M)VHLSSEEKSAVT(A) | 17 | 11 | 100 % | 71.04 | 48.78125 | 55.68 | 2 | | 643,837 | 1 285,6593 | 2 | 0,007978 | 6,201 | 554,52 | 584624 | 2 | 13 |
| (M)VHLSSEEKSAVTA(L) | 28 | 12 | 100 % | 91.05 | 48.39836 | 76.79 | 2 | | 679,3539 | 1 356,6932 | 2 | 0,004758 | 3,504 | 636,116 | 1123210 | 2 | 14 |
| (M)VHLSSEEKSAVTAL(W) | 62 | 14 | 100 % | 109.16 | 48.40683 | 86.8 | 2 | | 735,8961 | 1 469,7777 | 2 | 0,005158 | 3,507 | 1 116,56 | 938039 | 2 | 15 |
| (M)VHLSSEEKSAVTALW(G) | 58 | 14 | 100 % | 118.14 | 49.22544 | 93.21 | 2 | | 828,9347 | 1 655,8549 | 2 | 0,003018 | 1,822 | 1 646,83 | 964024 | 2 | 16 |
| (M)VHLSSEEKSAVTALWGKVNVEEVG(G) | 7 | 24 | 100 % | 129.81 | 50.39755 | 112.35 | 2 | | 856,7816 | 2 567,3230 | 3 | -0,0001979 | -0,07707 | 1 815,27 | 138921 | 2 | 25 |
| (M)VHLSSEEKSAVTALWGKVNVEEVGGEAL(G) | 3 | 28 | 100 % | 121.05 | 50.902016 | 110.25 | 2 | | 980,1771 | 2 937,5094 | 3 | 0,0009721 | 0,3308 | 1 977,84 | 37 604,0 | 2 | 29 |
| (M)VHLSSEEKSAVTALWGKVNVEEVGGEALG(R) | 2 | 29 | 100 % | 86.94 | 50.989967 | 71.62 | 2 | | 999,1842 | 2 994,5308 | 3 | 0,0008621 | 0,2878 | 1 956,79 | 73 605,0 | 2 | 30 |
| (M)VHLSSEEKSAVTALWGKVNVEEVGGEALGRL(L) | 1 | 31 | 99 % | 59.21 | 50.572514 | 58.15 | 2 | | 1 088,9117 | 3 263,7134 | 3 | -0,001808 | -0,5538 | 2 032,82 | 147496 | 2 | 32 |
| (V)HLSSEEKSAVT(A) | 18 | 11 | 100 % | 68.49 | 48.236435 | 47.92 | 2 | | 594,2981 | 1 186,5817 | 2 | -0,001262 | -1,063 | 534,683 | 204672 | 3 | 13 |
| (V)HLSSEEKSAVTA(L) | 24 | 12 | 100 % | 78.51 | 48.15405 | 59.24 | 2 | | 629,8187 | 1 257,6227 | 2 | 0,002678 | 2,128 | 329,393 | 333530 | 3 | 14 |
| (V)HLSSEEKSAVTAL(W) | 11 | 14 | 100 % | 93.55 | 48.75177 | 52.78 | 2 | | 686,3604 | 1 370,7063 | 2 | 0,002118 | 1,544 | 882,202 | 75 317,0 | 3 | 15 |
| (V)HLSSEEKSAVTALW(G) | 10 | 14 | 100 % | 103.28 | 49.05007 | 81.33 | 2 | | 779,4007 | 1 556,7869 | 2 | 0,003418 | 2,194 | 1 540,44 | 364148 | 3 | 16 |
| (H)LSSEEKSAVT(A) | 5 | 10 | 91 % | 37.55 | 47.75188 | 18.75 | 2 | | 1 050,5299 | 1 049,5227 | 1 | -0,001406 | -1,338 | 542,842 | 3 888,00 | 4 | 13 |
| (H)LSSEEKSAVTA(L) | 3 | 11 | 100 % | 64.81 | 48.433197 | 35.34 | 2 | | 1 121,5690 | 1 120,5617 | 1 | 0,0005041 | 0,4494 | 622,291 | 6 283,00 | 4 | 14 |
| (H)LSSEEKSAVTAL(W) | 5 | 12 | 95 % | 42.3 | 48.575737 | 18.45 | 2 | | 1 234,6525 | 1 233,6452 | 1 | -0,00005594 | -0,04531 | 1 142,69 | 3 316,00 | 4 | 15 |
| (L)SSEEKSAVTAL(W) | 27 | 11 | 99 % | 51.49 | 48.30986 | 26.25 | 2 | | 1 121,5667 | 1 120,5594 | 1 | -0,001726 | -1,539 | 836,619 | 3 212,00 | 5 | 15 |
| (L)WGKVNVEEVGGEA(L) | 10 | 13 | 100 % | 97.34 | 48.317867 | 62.03 | 2 | | 687,3378 | 1 372,6610 | 2 | -0,001362 | -0,9915 | 1 323,11 | 140173 | 16 | 28 |
| (L)WGKVNVEEVGGEAL(G) | 6 | 14 | 100 % | 127.97 | 48.85033 | 97.45 | 2 | | 743,8818 | 1 485,7490 | 2 | 0,002518 | 1,694 | 1 651,51 | 545282 | 16 | 29 |
| (L)WGKVNVEEVGGEALG(R) | 6 | 15 | 100 % | 88.02 | 48.90074 | 66.59 | 2 | | 772,3896 | 1 542,7646 | 2 | -0,003402 | -2,204 | 1 645,75 | 45 055,0 | 16 | 30 |
| (L)WGKVNVEEVGGEALGR(L) | 3 | 16 | 100 % | 99.66 | 49.276527 | 79.57 | 2 | | 850,4418 | 1 698,8690 | 2 | -0,00008194 | -0,0482 | 1 506,52 | 456746 | 16 | 31 |
| (L)WGKVNVEEVGGEALGRL(L) | 7 | 17 | 100 % | 134.3 | 49.066315 | 95.17 | 2 | | 906,984 | 1 811,9534 | 2 | 0,0002381 | 0,1313 | 1 790,72 | 719262 | 16 | 32 |
| (L)WGKVNVEEVGGEALGRLL(V) | 8 | 18 | 100 % | 163.55 | 54.006382 | 142.38 | 2 | | 963,5244 | 1 925,0342 | 2 | -0,003022 | -1,569 | 2 039,84 | 752022 | 16 | 33 |
| (W)GKVNVEEVGGEAL(G) | 5 | 13 | 100 % | 67.57 | 48.56584 | 36.93 | 2 | | 650,8415 | 1 299,6684 | 2 | 0,001258 | 0,9672 | 1 300,86 | 37 374,0 | 17 | 29 |
| (W)GKVNVEEVGGEALG(R) | 7 | 14 | 100 % | 62.02 | 48.546337 | 46.2 | 2 | | 679,3514 | 1 356,6881 | 2 | -0,0005219 | -0,3844 | 1 240,42 | 54 385,0 | 17 | 30 |
| (W)GKVNVEEVGGEALGR(L) | 7 | 15 | 100 % | 113.04 | 48.700058 | 82.96 | 2 | | 757,4044 | 1 512,7942 | 2 | 0,004418 | 2,919 | 1 044,28 | 574174 | 17 | 31 |
| (W)GKVNVEEVGGEALGRL(L) | 7 | 16 | 100 % | 116.89 | 48.081097 | 89.09 | 2 | | 813,9472 | 1 625,8798 | 2 | 0,005958 | 3,662 | 1 590,74 | 786293 | 17 | 32 |
| (W)GKVNVEEVGGEALGRLL(V) | 18 | 17 | 100 % | 133.52 | 47.727398 | 106.94 | 2 | | 870,4801 | 1 738,9457 | 2 | -0,01226 | -7,047 | 1 900,67 | 1102320 | 17 | 33 |
| (W)GKVNVEEVGGEALGRLLV(V) | 6 | 18 | 100 % | 106.21 | 46.501423 | 94.49 | 2 | | 613,6822 | 1 838,0248 | 3 | -0,001598 | -0,8689 | 1 991,87 | 55 171,0 | 17 | 34 |
| (G)KVNVEEVGGEALGRL(L) | 4 | 15 | 100 % | 108.4 | 47.84218 | 87.0 | 2 | | 785,4343 | 1 568,8540 | 2 | 0,001678 | 1,069 | 1 589,57 | 446475 | 18 | 32 |
| (G)KVNVEEVGGEALGRLL(V) | 6 | 16 | 100 % | 101.92 | 46.648956 | 76.04 | 2 | | 841,9774 | 1 681,9402 | 2 | 0,003758 | 2,233 | 1 885,71 | 414171 | 18 | 33 |
| (K)VNVEEVGGEAL(G) | 3 | 11 | 99 % | 51.74 | 47.676678 | 17.6 | 2 | | 1 115,5561 | 1 114,5488 | 1 | -0,001856 | -1,664 | 1 585,13 | 9 239,00 | 19 | 29 |
| (K)VNVEEVGGEALGRL(L) | 5 | 14 | 100 % | 100.19 | 48.170696 | 73.86 | 2 | | 721,3826 | 1 440,7506 | 2 | -0,006782 | -4,704 | 1 804,74 | 77 259,0 | 19 | 32 |
| (K)VNVEEVGGEALGRLL(V) | 3 | 15 | 100 % | 60.92 | 47.911293 | 47.82 | 2 | | 777,9241 | 1 553,8336 | 2 | -0,007842 | -5,044 | 1 888,97 | 71 796,0 | 19 | 33 |
| (V)NVEEVGGEALG(R) | 5 | 11 | 99 % | 55.62 | 46.45206 | 33.3 | 2 | | 1 073,5032 | 1 072,4959 | 1 | -0,007856 | -7,318 | 1 192,80 | 7 995,00 | 20 | 30 |
| (V)NVEEVGGEALGRLL(V) | 15 | 14 | 100 % | 109.85 | 47.91157 | 82.9 | 2 | | 728,3951 | 1 454,7756 | 2 | 0,002498 | 1,716 | 1 976,67 | 410778 | 20 | 33 |
| (N)VEEVGGEAL(G) | 9 | 9 | 99 % | 57.19 | 47.939857 | 17.01 | 2 | | 902,445 | 901,4377 | 1 | -0,001626 | -1,802 | 1 223,91 | 6 045,00 | 21 | 29 |
| (N)VEEVGGEALGRL(L) | 6 | 12 | 100 % | 83.41 | 48.049908 | 47.44 | 2 | | 614,8293 | 1 227,6440 | 2 | -0,002022 | -1,646 | 1 538,11 | 50 309,0 | 21 | 32 |
| (N)VEEVGGEALGRLL(V) | 21 | 13 | 100 % | 97.36 | 47.253723 | 60.65 | 2 | | 671,3727 | 1 340,7307 | 2 | 0,0005781 | 0,4308 | 1 862,07 | 776792 | 21 | 33 |
| (N)VEEVGGEALGRLLV(V) | 5 | 14 | 100 % | 102.7 | 46.678356 | 71.51 | 2 | | 720,9048 | 1 439,7950 | 2 | -0,003522 | -2,444 | 2 001,23 | 101553 | 21 | 34 |
| (V)EEVGGEALGRL(L) | 6 | 11 | 100 % | 86.47 | 53.989864 | 57.27 | 2 | | 565,2951 | 1 128,5757 | 2 | -0,001982 | -1,755 | 1 413,04 | 139766 | 22 | 32 |
| (V)EEVGGEALGRLL(V) | 8 | 12 | 100 % | 80.22 | 48.26671 | 60.1 | 2 | | 621,8347 | 1 241,6549 | 2 | -0,006842 | -5,506 | 1 829,31 | 992675 | 22 | 33 |
| (E)EVGGEALGRLL(V) | 5 | 11 | 100 % | 70.94 | 46.510258 | 50.93 | 2 | | 557,3155 | 1 112,6164 | 2 | -0,002742 | -2,462 | 1 786,04 | 197792 | 23 | 33 |
| (E)VGGEALGRL(L) | 4 | 9 | 98 % | 45.54 | 46.49792 | 17.56 | 2 | | 436,2507 | 870,4868 | 2 | -0,005622 | -6,451 | 1 413,13 | 12 650,0 | 24 | 32 |
| (E)VGGEALGRLL(V) | 10 | 10 | 99 % | 48.3 | 44.76687 | 15.4 | 2 | | 492,7943 | 983,574 | 2 | -0,002582 | -2,622 | 1 674,91 | 203555 | 24 | 33 |
| (L)LVVYPWTQRF(F) | 7 | 10 | 99 % | 57.02 | 47.71477 | 32.97 | 2 | | 654,8578 | 1 307,7010 | 2 | -0,001702 | -1,3 | 2 003,57 | 157582 | 33 | 42 |
| (L)VVYPWTQRF(F) | 8 | 9 | 99 % | 53.56 | 47.920975 | 29.3 | 2 | | 598,3166 | 1 194,6186 | 2 | 0,00007806 | 0,06529 | 1 814,10 | 29 659,0 | 34 | 42 |
| (V)VYPWTQRF(F) | 5 | 8 | 95 % | 42.0 | 47.688232 | 26.37 | 2 | | 548,7796 | 1 095,5446 | 2 | -0,005542 | -5,054 | 1 670,57 | 16 283,0 | 35 | 42 |
| (V)YPWTQRF(F) | 19 | 7 | 90 % | 43.98 | 47.10566 | 19.96 | 2 | | 499,2459 | 996,4772 | 2 | -0,004522 | -4,533 | 1 993,21 | 27 713,0 | 36 | 42 |
| (F)FESFGDLSSA(N) | 6 | 10 | 99 % | 50.67 | 44.31444 | 29.66 | 2 | | 1 059,4605 | 1 058,4533 | 1 | -0,002416 | -2,28 | 1 718,34 | 8 137,00 | 43 | 52 |
| (F)FESFGDLSSAN(A) | 6 | 11 | 99 % | 47.04 | 44.157745 | 36.43 | 2 | | 1 173,5044 | 1 172,4971 | 1 | -0,001486 | -1,266 | 1 635,14 | 6 874,00 | 43 | 53 |
| (F)FESFGDLSSANA(V) | 4 | 12 | 94 % | 37.38 | 44.382416 | 22.36 | 2 | | 1 244,5415 | 1 243,5343 | 1 | -0,001416 | -1,138 | 1 689,02 | 7 495,00 | 43 | 54 |
| (F)FESFGDLSSANAVMNNPKVKAH(G) | 6 | 22 | 100 % | 100.84 | 49.789154 | 86.29 | 2 | | 788,3875 | 2 362,1406 | 3 | 0,002982 | 1,262 | 1 580,28 | 49 374,0 | 43 | 64 |

| | | | | | | | | | | | | | | | | | |
|---|---|---|---|---|---|---|---|---|---|---|---|---|---|---|---|---|---|
| (F)FESFGDLSSANAVMNNPKVKAHGK(K) | 6 | 23 | 100 % | 94.6 | 50.45139 | 84.36 | 2 | | 850,0929 | 2 547,2568 | 3 | 0,002742 | 1,076 | 1 453,94 | 92 702,0 | 43 | 66 |
| (F)FESFGDLSSANAVMNNPKVKAHGKK(V) | 6 | 25 | 100 % | 102.03 | 50.7912 | 91.21 | 2 | | 892,7896 | 2 675,3470 | 3 | -0,002038 | -0,7615 | 1 309,02 | 199676 | 43 | 67 |
| (F)FESFGDLSSANAVMNNPKVKAHGKKV(L) | 4 | 26 | 92 % | 40.83 | 50.842224 | 39.56 | 2 | | 925,8118 | 2 774,4135 | 3 | -0,003988 | -1,437 | 1 348,75 | 27 206,0 | 43 | 68 |
| (F)FESFGDLSSANAVMNNPKVKAHGKKVL(A) | 9 | 27 | 100 % | 106.27 | 50.596012 | 96.05 | 2 | | 963,508 | 2 887,5020 | 3 | 0,0004521 | 0,1565 | 1 486,65 | 206468 | 43 | 69 |
| (F)FESFGDLSSANAVMNNPKVKAHGKKVLA(A) | 5 | 28 | 100 % | 96.96 | 50.66311 | 94.44 | 2 | | 740,6428 | 2 958,5419 | 4 | 0,003226 | 1,09 | 1 478,48 | 182452 | 43 | 70 |
| (F)FESFGDLSSANAVMNNPKVKAHGKKVLAA(F) | 6 | 29 | 100 % | 74.88 | 50.813942 | 73.32 | 2 | | 758,4003 | 3 029,5721 | 4 | -0,003674 | -1,212 | 1 493,66 | 132855 | 43 | 71 |
| (F)GDLSSANAVMNNPKV(K) | 29 | 15 | 100 % | 125.4 | 49.033775 | 109.97 | 2 | | 758,8786 | 1 515,7427 | 2 | 0,007538 | 4,97 | 1 320,69 | 400260 | 47 | 61 |
| (V)MNNPKVKAH(G) | 6 | 9 | 100 % | 72.98 | 47.035923 | 58.91 | 2 | | 519,7792 | 1 037,5437 | 2 | -0,0003219 | -0,31 | 351,61 | 46 769,0 | 56 | 64 |
| (F)SEGLSHLDNL(K) | 14 | 10 | 95 % | 40.77 | 47.28808 | 21.42 | 2 | | 542,7656 | 1 083,5166 | 2 | -0,003202 | -2,952 | 1 415,38 | 332769 | 73 | 82 |
| (F)SEGLSHLDNLK(G) | 8 | 11 | 100 % | 77.45 | 47.83432 | 55.3 | 2 | | 606,8129 | 1 211,6112 | 2 | -0,003582 | -2,954 | 922,981 | 54 595,0 | 73 | 83 |
| (F)SEGLSHLDNLKG(T) | 10 | 12 | 100 % | 98.06 | 48.253094 | 81.8 | 2 | | 635,324 | 1 268,6334 | 2 | -0,002882 | -2,27 | 939,326 | 314267 | 73 | 84 |
| (F)SEGLSHLDNLKGTFAKL(S) | 9 | 17 | 100 % | 95.23 | 49.179043 | 85.05 | 2 | | 610,6622 | 1 828,9648 | 3 | -0,003768 | -2,059 | 1 677,25 | 251688 | 73 | 89 |
| (S)EGLSHLDNL(K) | 3 | 9 | 91 % | 36.48 | 47.233818 | 2.77 | 2 | | 997,4938 | 996,4866 | 1 | -0,001216 | -1,219 | 1 342,99 | 3 445,00 | 74 | 82 |
| (L)SELHCDKLHVDPENFRL(L) | 3 | 17 | 90 % | 38.05 | 49.37864 | 25.44 | 2 | | 684,6692 | 2 050,9858 | 3 | -0,003838 | -1,87 | 1 640,99 | 47 945,0 | 90 | 106 |
| (L)HCDKLHVDPENFRL(L) | 2 | 14 | 97 % | 47.47 | 49.04245 | 35.96 | 2 | | 574,9494 | 1 721,8265 | 3 | -0,004508 | -2,617 | 1 589,57 | 97 998,0 | 93 | 106 |
| (C)DKLHVDPENFRL(L) | 5 | 12 | 100 % | 69.57 | 48.765873 | 49.0 | 2 | | 494,9256 | 1 481,7549 | 3 | -0,008018 | -5,407 | 1 589,57 | 84 525,0 | 95 | 106 |
| (L)HVDPENF(R) | 3 | 7 | 74 % | 26.21 | 44.92928 | 4.75 | 2 | | 857,3761 | 856,3688 | 1 | -0,002746 | -3,203 | 724,034 | 2 608,00 | 98 | 104 |
| (L)HVDPENFR(L) | 4 | 8 | 98 % | 47.51 | 46.77525 | 24.62 | 2 | | 507,2432 | 1 012,4717 | 2 | -0,0009219 | -0,9097 | 612,794 | 215822 | 98 | 105 |
| (L)HVDPENFRL(L) | 4 | 9 | 98 % | 47.8 | 47.64572 | 16.56 | 2 | | 563,7846 | 1 125,5546 | 2 | -0,002202 | -1,955 | 1 337,06 | 862650 | 98 | 106 |
| (L)SHHFGKE(F) | 2 | 7 | 90 % | 35.88 | 44.656357 | 17.66 | 2 | | 421,1983 | 840,3821 | 2 | -0,005762 | -6,848 | 483,347 | 20 806,0 | 116 | 122 |
| (L)SHHFGKEF(T) | 3 | 8 | 95 % | 39.56 | 46.45304 | 21.82 | 2 | | 494,7342 | 987,4538 | 2 | -0,002502 | -2,531 | 481,012 | 91 582,0 | 116 | 123 |
| (L)SHHFGKEFTPQV(Q) | 3 | 12 | 100 % | 71.35 | 48.192673 | 57.32 | 2 | | 707,3484 | 1 412,6822 | 2 | -0,001562 | -1,105 | 862,28 | 46 890,0 | 116 | 127 |
| (L)SHHFGKEFTPQVQ(A) | 8 | 13 | 100 % | 71.46 | 48.588074 | 59.06 | 2 | | 771,3793 | 1 540,7441 | 2 | 0,001738 | 1,127 | 800,466 | 189715 | 116 | 128 |
| (L)SHHFGKEFTPQVQA(A) | 5 | 14 | 100 % | 73.13 | 48.841606 | 56.95 | 2 | | 806,8978 | 1 611,7810 | 2 | 0,001558 | 0,9661 | 863,447 | 207973 | 116 | 129 |
| (L)SHHFGKEFTPQVQAA(Y) | 4 | 15 | 100 % | 81.1 | 48.97583 | 72.47 | 2 | | 842,418 | 1 682,8214 | 2 | 0,004798 | 2,849 | 863,447 | 529462 | 116 | 130 |
| (L)SHHFGKEFTPQVQAAY(Q) | 4 | 16 | 100 % | 101.57 | 48.815784 | 90.57 | 2 | | 923,9467 | 1 845,8788 | 2 | -0,001022 | -0,5533 | 1 121,22 | 71 855,0 | 116 | 131 |
| (A)YQKVVAGVAN(A) | 6 | 10 | 96 % | 42.47 | 47.63308 | 25.18 | 2 | | 1 048,5766 | 1 047,5693 | 1 | -0,001986 | -1,894 | 680,906 | 1 912,00 | 131 | 140 |
| (A)YQKVVAGVANALA(H) | 5 | 13 | 100 % | 75.25 | 46.62191 | 47.26 | 2 | | 652,372 | 1 302,7293 | 2 | -0,0002219 | -0,1702 | 1 471,47 | 257560 | 131 | 143 |
| (A)YQKVVAGVANALAH(K) | 2 | 14 | 100 % | 66.01 | 47.317337 | 53.77 | 2 | | 720,8995 | 1 439,7844 | 2 | -0,004102 | -2,847 | 1 287,03 | 28 362,0 | 131 | 144 |
| (A)YQKVVAGVANALAHK(Y) | 6 | 15 | 100 % | 123.38 | 46.273865 | 103.86 | 2 | | 784,9468 | 1 567,8790 | 2 | -0,004502 | -2,87 | 1 059,45 | 107279 | 131 | 145 |
| (A)YQKVVAGVANALAHKY(-) | 3 | 16 | 100 % | 91.84 | 47.845604 | 78.14 | 2 | | 577,9897 | 1 730,9472 | 3 | 0,0004721 | 0,2726 | 1 431,74 | 59 044,0 | 131 | 146 |
| (A)YQKVVAGVANALAHKYH(-) | 6 | 17 | 100 % | 106.01 | 48.30582 | 96.16 | 2 | | 623,676 | 1 868,0061 | 3 | 0,0003821 | 0,2044 | 1 244,84 | 139398 | 131 | 147 |
