# Supplementary material for: Unveiling the Peptidase Network Orchestrating Hemoglobin Catabolism in Rhodnius prolixus
Source: Mol Cell Proteomics. 2024 Apr 23;23(6):100775. doi: 10.1016/j.mcpro.2024.100775 (PMC11135036; doi:10.1016/j.mcpro.2024.100775)
Supplement: Supplemental Table S8 [file mmc8.pdf]

Table S8: Position-Specific Scoring Matrix representing cleavage site preferences of *R. prolixus* major hemoglobinases

Amino acids (AA)		Hemoglobin AA number	Back. Freq	P4				P3				P2				P1				P1'				P2'				P3'				P4'			
				N°	Freq	F/BF	Log-odds	N°	Freq	F/BF	Log-odds	N°	Freq	F/BF	Log-odds	N°	Freq	F/BF	Log-odds	N°	Freq	F/BF	Log-odds	N°	Freq	F/BF	Log-odds	N°	Freq	F/BF	Log-odds	N°	Freq	F/BF	Log-odds
**A1 aspartic proteases**																																			
ALA	A	36	0,12456747	34	0,07	0,562	-0,832348	4	0,008	0,066	-3,9198	97	0,2	1,602	0,68010	40	0,082	0,661	-0,597	75	0,154	1,239	0,309006	177	0,36	2,92	1,5477	12	0,02	0,2	-2,334	2	0,004	0,033	-4,91981182254742
Arg	R	6	0,02076125	0	0,001	0,048	-4,375821	26	0,053	2,577	1,36559	58	0,119	5,748	2,52313	0	0,001	0,048	-4,375	12	0,025	1,189	0,250113	36	0,07	3,57	1,8350	4	0,01	0,4	-1,334	0	0,001	0,048	-4,37582110288256
Asn	N	10	0,03460208	4	0,008	0,238	-2,071814	2	0,004	0,119	-3,0718	57	0,117	3,39	1,76107	8	0,016	0,476	-1,071	0	0,001	0,029	-5,11278	0	0	0,03	-5,112	11	0,02	0,65	-0,612	0	0,001	0,029	-5,11278669704877
ASP	D	15	0,05190311	31	0,064	1,229	0,297418	152	0,313	6,026	2,5911	0	0,001	0,019	-5,6977	11	0,023	0,436	-1,197	2	0,004	0,079	-3,65677	3	0,01	0,12	-3,071	12	0,02	0,48	-1,071	0	0,001	0,019	-5,69774919776993
Asx	B	0	0,001	0	0,001	1	0	0	0,001	1	0	0	0,001	1	0	0	0,001	1	0	0	0,001	1	0	0	0	1	0	0	0	1	0	0	0,001	1	0
Cys	C	3	0,01038062	0	0,001	0,096	-3,375821	0	0,001	0,096	-3,3758	0	0,001	0,096	-3,3758	0	0,001	0,096	-3,375	0	0,001	0,096	-3,37582	2	0	0,4	-1,334	0	0	0,1	-3,375	0	0,001	0,096	-3,37582110288256
Gln	Q	4	0,01384083	0	0,001	0,072	-3,790858	23	0,047	3,419	1,77367	0	0,001	0,072	-3,7908	0	0,001	0,072	-3,790	0	0,001	0,072	-3,79085	21	0,04	3,12	1,6424	6	0,01	0,89	-0,164	0	0,001	0,072	-3,79085860216141
Glu	E	12	0,04152249	4	0,008	0,198	-2,33484	55	0,113	2,725	1,44651	6	0,012	0,297	-1,7498	0	0,001	0,024	-5,375	8	0,016	0,396	-1,33484	6	0,01	0,3	-1,749	2	0	0,02	-5,375	0	0,001	0,024	-5,37582110288256
Glx	Z	0	0,001	0	0,001	1	0	0	0,001	1	0	0	0,001	1	0	0	0,001	1	0	0	0,001	1	0	0	0	1	0	0	0	1	0	0	0,001	1	0
Gly	G	20	0,06920415	57	0,117	1,695	0,761075	71	0,146	2,111	1,07793	0	0,001	0,014	-6,1127	6	0,012	0,178	-2,486	32	0,066	0,951	-0,07181	35	0,07	1,04	0,0574	2	0	0,01	-6,112	0	0,001	0,014	-6,11278669704877
His	H	19	0,06574394	0	0,001	0,015	-6,038786	12	0,025	0,376	-1,4128	8	0,016	0,25	-1,9978	4	0,008	0,125	-2,997	0	0,001	0,015	-6,03878	10	0,02	0,31	-1,675	15	0,03	0,47	-1,090	23	0,047	0,72	-0,474252378491675
Ile	I	0	0,001	0	0,001	1	0	0	0,001	1	0	0	0,001	1	0	0	0,001	1	0	0	0,001	1	0	0	0	1	0	0	0	1	0	0	0,001	1	0
Leu	L	36	0,12456747	231	0,475	3,816	1,931937	4	0,008	0,066	-3,9198	19	0,039	0,314	-1,6718	184	0,379	3,039	1,6037	210	0,432	3,469	1,794433	47	0,1	0,78	-0,365	94	0,19	1,55	0,634	34	0,07	0,562	-0,832348981297075
Lys	K	22	0,07612457	23	0,047	0,622	-0,685756	35	0,072	0,946	-0,0800	158	0,325	4,271	2,09446	2	0,004	0,054	-4,209	33	0,068	0,892	-0,16492	0	0	0,01	-6,250	54	0,11	1,46	0,545	2	0,004	0,054	-4,2093184397424
Met	M	5	0,01730104	0	0,001	0,058	-4,112786	0	0,001	0,058	-4,1127	4	0,008	0,476	-1,0718	4	0,008	0,476	-1,071	0	0,001	0,058	-4,11278	0	0	0,06	-4,112	8	0,02	0,95	-0,071	4	0,008	0,476	-1,07181491599246
Phe	F	15	0,05190311	2	0,004	0,079	-3,656777	4	0,008	0,159	-2,6567	37	0,076	1,467	0,55267	215	0,442	8,523	3,0914	6	0,012	0,238	-2,07181	0	0	0,02	-5,697	6	0,01	0,24	-2,071	23	0,047	0,912	-0,133215460656608
Pro	P	14	0,04844291	42	0,086	1,784	0,835075	2	0,004	0,085	-3,5572	0	0,001	0,021	-5,5982	0	0,001	0,021	-5,598	13	0,027	0,552	-0,85680	0	0	0,02	-5,598	0	0	0,02	-5,598	31	0,064	1,317	0,396954567224169
Ser	S	16	0,05536332	6	0,012	0,223	-2,164924	11	0,023	0,409	-1,2904	6	0,012	0,223	-2,1649	2	0,004	0,074	-3,749	9	0,019	0,334	-1,57996	35	0,07	1,3	0,3793	154	0,32	5,72	2,516	96	0,198	3,568	1,83507567961605
Thr	T	16	0,05536332	2	0,004	0,074	-3,749886	44	0,091	1,635	0,70954	21	0,043	0,78	-0,3575	8	0,016	0,297	-1,749	2	0,004	0,074	-3,74988	30	0,06	1,11	0,1570	4	0,01	0,15	-2,749	2	0,004	0,074	-3,7498868211051
Trp	W	3	0,01038062	0	0,001	0,096	-3,375821	0	0,001	0,096	-3,3758	0	0,001	0,096	-3,3758	0	0,001	0,096	-3,375	29	0,06	5,748	2,523131	0	0	0,1	-3,375	0	0	0,1	-3,375	0	0,001	0,096	-3,37582110288256
Tyr	Y	6	0,02076125	0	0,001	0,048	-4,375821	0	0,001	0,048	-4,3758	0	0,001	0,048	-4,3758	2	0,004	0,198	-2,334	21	0,043	2,081	1,057468	0	0	0,05	-4,375	15	0,03	1,49	0,572	48	0,099	4,757	2,2501131788949
Val	V	31	0,10726644	50	0,103	0,959	-0,060226	41	0,084	0,786	-0,3465	15	0,031	0,288	-1,7971	0	0,001	0,009	-6,745	34	0,07	0,652	-0,61662	84	0,17	1,61	0,6882	87	0,18	1,67	0,738	221	0,455	4,239	2,08381942789945
Total				**486**	1			**486**	1			**486**	1			**486**	1			**486**	1			**486**	1			**486**	1			**486**	1		
**C1 cysteine proteases**																																			
ALA	A	36	0,12456747	230	0,12	0,965	-0,05113	161	0,084	0,676	-0,5657	128	0,067	0,537	-0,8966	196	0,102	0,823	-0,281	411	0,215	1,725	0,786374	165	0,09	0,69	-0,530	710	0,38	3,02	1,592	240	0,127	1,019	0,0277207575584501
Arg	R	6	0,02076125	12	0,006	0,302	-1,72669	0	0,001	0,05	-4,3116	0	0,001	0,048	-4,3758	80	0,042	2,014	1,0102	10	0,005	0,252	-1,98972	2	0	0,05	-4,375	8	0	0,05	-4,375	2	0,001	0,051	-4,29420733732891
Asn	N	10	0,03460208	116	0,061	1,752	0,809357	114	0,06	1,722	0,78426	49	0,026	0,74	-0,4339	143	0,075	2,16	1,1112	4	0,002	0,06	-4,04862	18	0,01	0,27	-1,878	29	0,02	0,44	-1,173	24	0,013	0,367	-1,44621043077396
ASP	D	15	0,05190311	106	0,055	1,068	0,094334	20	0,01	0,201	-2,3116	446	0,233	4,492	2,16731	28	0,015	0,282	-1,826	20	0,01	0,201	-2,31165	108	0,06	1,09	0,1213	74	0,04	0,75	-0,406	180	0,095	1,835	0,8757176641134
Asx	B	0	0,001	0	0,001	1	0	0	0,001	1,045	0,06416	0	0,001	1	0	0	0,001	1	0	0	0,001	1	0	0	0	1	0	0	0	1	0	0	0,001	1	0
Cys	C	3	0,01038062	0	0,001	0,096	-3,375821	0	0,001	0,101	-3,3116	0	0,001	0,096	-3,3758	0	0,001	0,096	-3,375	13	0,007	0,655	-0,61121	0	0	0,1	-3,375	5	0	0,1	-3,375	0	0,001	0,096	-3,37582110288256
Gln	Q	4	0,01384083	2	0,001	0,076	-3,72669	0	0,001	0,076	-3,7266	2	0,001	0,076	-3,7266	14	0,007	0,529	-0,919	4	0,002	0,151	-2,72669	0	0	0,07	-3,790	0	0	0,07	-3,790	2	0,001	0,076	-3,70924483660776
Glu	E	12	0,04152249	16	0,008	0,201	-2,311657	62	0,032	0,781	-0,3574	44	0,023	0,554	-0,8522	101	0,053	1,272	0,3465	39	0,02	0,491	-1,02625	65	0,03	0,82	-0,289	26	0,01	0,33	-1,593	111	0,059	1,414	0,500208529021194
Glx	Z	0	0,001	0	0,001	1	0	0	0,001	1,045	0,06416	0	0,001	1	0	0	0,001	1	0	0	0,001	1	0	0	0	1	0	0	0	1	0	0	0,001	1	0
Gly	G	20	0,06920415	172	0,09	1,299	0,377641	113	0,059	0,854	-0,2284	144	0,075	1,088	0,12130	346	0,181	2,614	1,3860	78	0,041	0,589	-0,76322	197	0,1	1,49	0,5734	338	0,18	2,58	1,369	85	0,045	0,65	-0,621781995357417
His	H	19	0,06574394	272	0,142	2,163	1,112839	39	0,02	0,31	-1,6892	82	0,043	0,652	-0,6170	74	0,039	0,588	-0,765	29	0,015	0,231	-2,11664	151	0,08	1,2	0,2637	8	0	0,02	-6,038	106	0,056	0,853	-0,229251895488142
Ile	I	0	0,001	0	0,001	1	0	0	0,001	1,045	0,06416	0	0,001	1	0	0	0,001	1	0	0	0,001	1	0	0	0	1	0	0	0	1	0	0	0,001	1	0
Leu	L	36	0,12456747	52	0,027	0,218	-2,19618	534	0,279	2,241	1,16407	268	0,14	1,125	0,16946	97	0,051	0,407	-1,296	225	0,118	0,944	-0,08283	630	0,33	2,64	1,4025	176	0,09	0,75	-0,419	231	0,122	0,981	-0,027420796634011
Lys	K	22	0,07612457	218	0,114	1,497	0,582057	259	0,135	1,779	0,83068	217	0,113	1,49	0,57542	703	0,367	4,827	2,2712	46	0,024	0,316	-1,66256	111	0,06	0,76	-0,391	22	0,01	0,15	-2,709	143	0,076	0,994	-0,00880511846666394
Met	M	5	0,01730104	36	0,019	1,088	0,121301	36	0,019	1,088	0,12130	0	0,001	0,058	-4,1127	1	5E-04	0,03	-5,048	35	0,018	1,058	0,080659	17	0,01	0,51	-0,961	0	0	0,06	-4,112	3	0,002	0,092	-3,44621043077396
Phe	F	15	0,05190311	33	0,017	0,332	-1,589191	44	0,023	0,443	-1,1741	43	0,022	0,433	-1,2073	0	0,001	0,019	-5,697	444	0,232	4,472	2,160829	229	0,12	2,31	1,2056	82	0,04	0,84	-0,258	61	0,032	0,622	-0,685398094653388
Pro	P	14	0,04844291	122	0,064	1,316	0,396686	142	0,074	1,532	0,61569	87	0,045	0,939	-0,0911	0	0,001	0,021	-5,598	1	5E-04	0,011	-6,53405	15	0,01	0,16	-2,627	58	0,03	0,63	-0,658	5	0,003	0,055	-4,194671663778
Ser	S	16	0,05536332	361	0,189	3,409	1,769159	18	0,009	0,17	-2,5567	47	0,025	0,444	-1,1721	29	0,015	0,274	-1,868	104	0,054	0,982	-0,02625	72	0,04	0,68	-0,556	258	0,14	2,47	1,301	493	0,261	4,712	2,23619899977016
Thr	T	16	0,05536332	79	0,041	0,746	-0,422914	20	0,01	0,189	-2,4047	25	0,013	0,236	-2,0828	20	0,01	0,189	-2,404	58	0,03	0,548	-0,86871	24	0,01	0,23	-2,141	2	0	0,02	-5,790	103	0,054	0,984	-0,0227443094245379
Trp	W	3	0,01038062	0	0,001	0,096	-3,375821	58	0,03	2,921	1,54632	30	0,016	1,511	0,59523	0	0,001	0,096	-3,375	0	0,001	0,096	-3,37582	14	0,01	0,71	-0,504	31	0,02	1,58	0,659	5	0,003	0,255	-1,97227924244155
Tyr	Y	6	0,02076125	37	0,019	0,932	-0,102204	10	0,005	0,252	-1,9897	46	0,024	1,158	0,21190	0	0,001	0,048	-4,375	85	0,044	2,14	1,097732	45	0,02	1,13	0,1801	14	0,01	0,36	-1,486	0	0,001	0,048	-4,37582110288256
Val	V	31	0,10726644	49	0,026	0,239	-2,066181	283	0,148	1,379	0,46376	255	0,133	1,243	0,31346	81	0,042	0,395	-1,341	307	0,16	1,496	0,581203	50	0,03	0,24	-2,037	49	0,03	0,24	-2,048	96	0,051	0,474	-1,07847864627348
Total				**1913**	1,005			**1913**	1			**1913**	1			**1913**	1			**1913**	1			**1913**	1			**1913**	1,01			**1913**	1,005		
